# Supplementary material for: Tspan8-Tumor Extracellular Vesicle-Induced Endothelial Cell and Fibroblast Remodeling Relies on the Target Cell-Selective Response
Source: Cells. 2020 Jan 29;9(2):319. doi: 10.3390/cells9020319 (PMC7072212; doi:10.3390/cells9020319)
Supplement: Supplementary file 1 [file cells-09-00319-s001.pdf]

Supplement

Tspan8-tumor extracellular vesicle-induced endothelial cell and fibroblast remodeling relies on the target cell-selective response

Wei Mu[1,2], Jan Provaznik[3], Thilo Hackert[2], Margot Zöller[2]

[1] School of Public Health, Shanghai Jiao Tong University School of Medicine, Shanghai, China, [2] Department of General, Visceral and Transplantation Surgery, Pancreas Section, University of Heidelberg, Germany, [3] EMBL Genomics Core Facility, Heidelberg, Germany

Supplementary Tables

Table S1 primers and antibodies
Table S2 most abundant RNA in endothelial cells, fibroblasts, AS-Tspan8-TEX
Table S3 Significant differences in mRNA signal strength between endothelial cells, fibroblasts and AS-Tspan8-TEX
Table S4 miRNA recovery in cells and TEX
Table S5 The impact of Tspan8 on TEX uptake and potential targets
Table S6 TEX coculture-induced distinct mRNA recovery in fibroblasts and endothelial cells
Table S7 Altered miRNA recovery in TEX-treated endothelial cells
Table S8 Noncoding RNA in rat endothelial cells and fibroblasts
Table S9 Annotation of noncoding RNA that expression is altered in TEX-treated rat fibroblasts and endothelial cells and human non-metastasizing tumor cells
Table S10 Full names of symbols (protein coding) (alphabetic)

Supplementary Figures

Figure S1 Functional assignment of mRNA in endothelial cells, fibroblasts and AS-Tspan8-TEX
Figure S2 Distinct miRNA recovery in AS, AS-Tspan8-, ASML- and ASML-Tspan8kd-TEX and cells
Figure S3 Distinct mRNA recovery in fibroblasts and endothelial cells cocultured with AS-Tspan8-TEX
Figure S4 Overview on molecular functions of fibroblast and endothelial cell mRNA after coculture with AS-Tspan8-TEX
Figure S5 AS- and AS-TEX-promoted fibroblast modulation
Figure S6 Angiogenesis-related mRNA modulation and associated signaling activation in TEX-treated endothelial cells
Figure S7 Recovery and functional assignment of predicted target mRNA of downregulated miRNA in TEX-treated endothelial cells
Figure S8 Pseudogenes and lncRNA in fibroblasts, endothelial cells and TEX

Table S1

Primers and antibodies

Table S1A

Primers

IL-1α	Forward	CAGGTCTCCTCATGGCTTTGC
	Reverse	CTTCCGAAAAGAAGGCTGTCC
IL-1β	Forward	AAGGCTGGGTGAAGACCCTTA
	Reverse	TGAATGGCCGTTTCTGGAAGT
IL-6	Forward	CGGTTAGCACACACTCCTTTG
	Reverse	CTTCGACGTGACAGACGCT
TNFα	Forward	ACAAACTGGGTAAAGGTGATGG
	Reverse	CAAGTTATCTGTGTCCCCAAAGC
VEGF	Forward	CTCTCCCCCGCAAAAGAAACG
	Reverse	CGGAACATCTCGAAGCGTTTA
TIMP1	Forward	ATCCACGGCATACTATCAACATC
	Reverse	CAAGGCTCACCATCATCGTAG
GAPDH	Forward	AGAGGGAAATCGTGCGTGAC
	Reverse	CAATAGTGATGACCTGGCCGT

Table S1B

Antibodies

**Name**	**Origin**	**Supplier**
Adam15	rabbit	Santa Cruz, HD, G
alpha 5 integrin	hamster	Becton Dickinson, HD, G
α6β4 (B5.5)	mouse IgG	ref. 1
Areg	rabbit	Santa Cruz, HD, G
beta1 integrin	hamster IgG	Becton Dickinson, HD, G
c-jun	mouse	Becton Dickinson, HD, G
CCR-2	rabbit	Cell Signaling, HD, G
CCR-7	rabbit	Cell Signaling, HD, G
CD163	mouse	Cell Signaling, HD, G
CD36	rabbit	Cell Signaling, HD, G
CD44	mouse	Cell Signaling, HD, G
CD86	mouse	Santa Cruz, HD, G
Cofilin	mouse	Santa Cruz, HD, G
Collagen IV	rabbit	Rockland, Gilbertsville, USA
CXCL1	rabbit	Cell Signaling, HD, G
CXCR2	rabbit	Cell Signaling, HD, G
CXCR4	rabbit	Cell Signaling, HD, G
CXCR5	rabbit	Cell Signaling, HD, G
E-cadherin	rabbit	Santa Cruz, HD, G
eLF1	rabbit	Cell Signaling, HD, G
Fos	rabbit	Cell Signaling, HD, G
Foxo3	rabbit	Santa Cruz, HD, G
HIF1α	mouse IgG	Becton Dickinson, HD, G
JNK	rabbit	Santa Cruz, HD, G
Keap	rabbit	Cell Signaling, HD, G
MMP-9	mouse	Dianova, Hamburg, G
NFκB p65	mouse	Becton Dickinson, HD, G
NOX1	rabbit	Cell Signaling, HD, G
NOX4	rabbit	Cell Signaling, HD, G
Nrf2	mouse	Cell Signaling, HD, G
p38 MAPK	rabbit	Cell Signaling, HD, G
Paxillin	rabbit	Santa Cruz, HD, G, G
pERK1/2	mouse	Becton Dickinson, HD, G
PI3K-p85	mouse	Santa Cruz, HD, G
PKCA	mouse	Becton Dickinson, HD, G
PPARγ	rabbit	Cell Signaling, HD, G
RhoB	rabbit	Cell Signaling, HD, G
SMAD4	rabbit	Santa Cruz, HD, G
Tspan5	rabbit	Cell Signaling, HD, G
VEGF	goat	Abcam, Cambridge, UK
VEGFR2	rabbit	Abcam, Cambridge, UK
Vimentin	rabbit	Santa Cruz, HD, G
Vinculin	rabbit	Cell Signaling, HD, G
secondary, dye-labeled antibodies		Dianova, Hamburg, G

reference 1

Matzku S, Wenzel A, Liu S, Zöller M. Antigenic differences between metastatic and nonmetastatic BSp73 rat tumor variants characterized by monoclonal antibodies. Cancer Res. 1989;49:1294-9.

Table S1C

miRNA inhibitors

miR-181a inhibitor: 5'-GTATCGCCTATACGTGTA-3'

miR-146b inhibitor: 5'-GTCACTCGTCTCCGAGA-3'

negative control inhibitor: 5′-GTGTAACACTATACGCCCA-3′

Table S2

Most abundant RNA in endothelial cells, fibroblasts and AS-Tspan8-TEX

Table S2A

Endothelial cells: 50 most abundant RNA

GeneSymbol	EC	GeneName
Actg1	258535	actin, gamma 1
Adm	104998	adrenomedullin
Anxa1	100721	annexin A1
Anxa2	162491	annexin A2
Atp5g2	106464	ATP synthase, H+ transporting, subunit C2
Bgn	324982	Biglycan
Cdhr1	191901	cadherin-related family member 1
Ceacam9	144429	carcinoembryonic antigen-related cell adhesion molecule 9
Col1a2	146445	collagen, type I, alpha 2
Ctsd	113317	cathepsin D
Eef1a1	174154	eukaryotic translation elongation factor 1 alpha 1
Eef1g	165905	eukaryotic translation elongation factor 1 gamma
Eno1	174154	enolase 1
Fau	246290	Finkel-Biskis-Reilly murine sarcoma virus (FBR-MuSV) ubiquit. expressed
Fth1	305328	ferritin, heavy polypeptide 1
Ftl	286863	ferritin, light polypeptide
Gnas	100721	GNAS complex locus
Hspa8	154795	heat shock protein 8
Ints7	160254	integrator complex subunit 7
Ldha	155872	lactate dehydrogenase A
Lgals1	132902	lectin, galactoside-binding, soluble, 1
LOC100364138	233005	ferritin light chain 1-like
LOC290595	100025	hypothetical gene supported by AF152002
LOC303448	162491	similar to glyceraldehyde-3-phosphate dehydrogenase
LOC310926	248003	hypothetical protein LOC310926
LOC687270	217401	similar to glyceraldehyde-3-phosphate dehydrogenase
Mapk3	100025	mitogen activated protein kinase 3
Mif	139509	macrophage migration inhibitory factor
Myl6	148489	myosin, light chain 6, smooth muscle and non-muscle
Myl6l	226633	myosin, light polypeptide 6, smooth muscle and non-muscle-like
Pabpc1	120611	poly(A) binding protein, cytoplasmic 1
Pcolce	198668	procollagen C-endopeptidase enhancer
Peo1	125733	progressive external ophthalmoplegia 1
Pgam1	194579	phosphoglycerate mutase 1
Pgk1	137588	phosphoglycerate kinase 1
Ppia	163621	peptidylprolyl isomerase A (cyclophilin A)
RGD1309537	104998	similar to Myosin regulatory light chain 2-A, smooth muscle isoform
RGD1559682	135694	similar to peptidylprolyl isomerase A (cyclophilin A)
RGD1562953	215899	similar to Rpl7a protein
Rps2	221969	ribosomal protein S2
S100a4	244589	S100 calcium-binding protein A4
S100a6	343512	S100 calcium binding protein A6
Sparc	118129	secreted protein, acidic, cysteine-rich (osteonectin)
Stfa2	338783	stefin A2
Timp2	187951	TIMP metallopeptidase inhibitor 2
Tmsb4x	117313	thymosin beta 4, X-linked
Tpt1	221969	tumor protein, translationally-controlled 1
Tubb4b	126607	tubulin, beta 4B class IVb
Ubb	147464	ubiquitin B
Ubc	154795	ubiquitin C

Table S2B

Lung fibroblasts: 50 most abundant RNA

Symbol	Fb	GeneName
Actg1	226633	actin, gamma 1
Anxa1	139509	annexin A1
Anxa2	149522	annexin A2
Atp5g2	109457	ATP synthase, H+ transporting, subunit C2 (subunit 9)
Bgn	345901	Biglycan
Cdhr1	106464	cadherin-related family member 1
Ceacam9	194579	carcinoembryonic antigen-related cell adhesion molecule 9
Col1a2	172951	collagen, type I, alpha 2
Cox4i1	99334	cytochrome c oxidase subunit IV isoform 1
Cox6a1	98648	cytochrome c oxidase, subunit VIa, polypeptide 1
Ctsb	130167	cathepsin B
Cx3cl1	128375	chemokine (C-X3-C motif) ligand 1
Eef1a1	177813	eukaryotic translation elongation factor 1 alpha 1
Eef1g	150562	eukaryotic translation elongation factor 1 gamma
Eno1	129267	enolase 1, (alpha)
Fau	265803	Finkel-Biskis-Reilly murine sarcoma virus (FBR-MuSV) ubiquit. expressed
Fth1	322737	ferritin, heavy polypeptide 1
Ftl	296979	ferritin, light polypeptide
Gnas	108701	GNAS complex locus
Hspa8	156956	heat shock protein 8
Ifi27	110985	interferon, alpha-inducible protein 27
Ints7	152664	integrator complex subunit 7
Lgals1	104998	lectin, galactoside-binding, soluble, 1
LOC100364138	233005	ferritin light chain 1-like
LOC303448	124864	similar to glyceraldehyde-3-phosphate dehydrogenase
LOC310926	301124	hypothetical protein LOC310926
LOC687270	187951	similar to glyceraldehyde-3-phosphate dehydrogenase
Mapk3	113317	mitogen activated protein kinase 3
Mt2A	100721	metallothionein 2A
Myl6	152664	myosin, light chain 6, alkali, smooth muscle and non-muscle
Myl6l	218913	myosin, light polypeptide 6, alkali, smooth muscle and non-muscle-like
Pabpc1	164759	poly(A) binding protein, cytoplasmic 1
Pcolce	198668	procollagen C-endopeptidase enhancer
Peo1	175365	progressive external ophthalmoplegia 1
Ppia	180295	peptidylprolyl isomerase A (cyclophilin A)
RGD1559682	133826	similar to peptidylprolyl isomerase A (cyclophilin A)
RGD1562953	184083	similar to ribosomal protein L7a
RGD1564839	107204	similar to ribosomal protein L31
Rps2	226633	ribosomal protein S2
S100a4	269514	S100 calcium-binding protein A4
S100a6	269514	S100 calcium binding protein A6
Sparc	150562	secreted protein, acidic, cysteine-rich (osteonectin)
Stfa2	358099	stefin A2
Timp2	220436	TIMP metallopeptidase inhibitor 2
Tkt	97966	Transketolase
Tmsb4x	127488	thymosin beta 4, X-linked
Tpt1	214408	tumor protein, translationally-controlled 1
Ubb	127488	ubiquitin B
Ubc	115698	ubiquitin C
Vim	110218	vimentin

Table S2C

AS-Tspan8-TEX: 50 most abundant RNA

Symbol	Tspan8-TEX	GeneName
Actg1	242993	actin, gamma 1
Atp5a1	179088	ATP synthase, H+ transporting, alpha subunit 1
Ceacam9	244642	carcinoembryonic antigen-related cell adhesion molecule 9
Chchd2	149580	coiled-coil-helix-coiled-coil-helix domain containing 2
Cox6a1	184088	cytochrome c oxidase, subunit VIa, polypeptide 1
Cxcl1	168855	chemokine (C-X-C motif) ligand 1 (melanoma growth stimulating activity, alpha)
Dbi	152144	diazepam binding inhibitor (GABA receptor modulator, acyl-CoA binding protein)
Eef1a1	174154	eukaryotic translation elongation factor 1 alpha 1
Eno1	240804	enolase 1, (alpha)
Fau	171760	Finkel-Biskis-Reilly murine sarcoma virus (FBR-MuSV) ubiquitously expressed
Fth1	327274	ferritin, heavy polypeptide 1
Ftl	304420	ferritin, light polypeptide
Gapdh	285319	glyceraldehyde-3-phosphate dehydrogenase
Hint1	112558	histidine triad nucleotide binding protein 1
Hnrnpab	132930	heterogeneous nuclear ribonucleoprotein A/B
Hspa8	156624	heat shock protein 8
Ifi27	131987	interferon, alpha-inducible protein 27
Ints7	113341	integrator complex subunit 7
Lcn2	372071	lipocalin 2
Ldha	266442	lactate dehydrogenase A
LOC100364138	264373	ferritin light chain 1-like
LOC299558	119789	similar to Zinc finger protein 44 (Zinc finger protein KOX7)
LOC303448	309598	similar to glyceraldehyde-3-phosphate dehydrogenase
LOC310926	282221	hypothetical protein LOC310926
LOC687270	321715	similar to glyceraldehyde-3-phosphate dehydrogenase
Mapk3	116528	mitogen activated protein kinase 3
Mif	265963	macrophage migration inhibitory factor
Myl6	140968	myosin, light chain 6, alkali, smooth muscle and non-muscle
Myl6l	222017	myosin, light polypeptide 6, alkali, smooth muscle and non-muscle-like
Pcolce	124439	procollagen C-endopeptidase enhancer
Pebp1	203646	phosphatidylethanolamine binding protein 1
Peo1	153859	progressive external ophthalmoplegia 1
Pgam1	269590	phosphoglycerate mutase 1
Pgk1	247396	phosphoglycerate kinase 1
Ppia	227531	peptidylprolyl isomerase A (cyclophilin A)
RGD1559682	259585	similar to peptidylprolyl isomerase A (cyclophilin A)
RGD1562953	201461	similar to ribosomal protein L7a
Rps2	240423	ribosomal protein S2
RT1-A1	165332	RT1 class Ia, locus A1
Stfa2	377264	stefin A2
Tkt	229847	Transketolase
Tmsb10	120200	thymosin, beta 10
Tpi1	175369	triosephosphate isomerase 1
Tpt1	118540	tumor protein, translationally-controlled 1
Tuba1b	149580	tubulin, alpha 1B
Tubb2a	120637	tubulin, beta 2A class IIa
Tubb4b	264735	tubulin, beta 4B class Ivb
Txn1	111424	thioredoxin 1
Ubb	243045	ubiquitin B
Ubc	188741	ubiquitin C

Table S3

Significant differences in mRNA signal strength between endothelial cells, fibroblasts and AS-Tspan8-TEX

Table S3A

Higher signal strength in endothelial cells than lung fibrobasts

Symbol	EC	Fb	EC:Fb	GeneName
Agtr1a	4240	331	12.82	angiotensin II receptor, type 1a
Aldoc	23494	2435	9.65	aldolase C, fructose-bisphosphate
Anpep	3397	478	7.11	alanyl (membrane) aminopeptidase
Aqp1	2628	461	5.70	aquaporin 1
Areg	2336	89	26.17	amphiregulin
Asns	1833	78	23.43	asparagine synthetase
Camk2n1	2210	261	8.46	calcium/calmodulin-dependent protein kinase II inhibitor 1
Cd248	8841	108	82.14	CD248 molecule, endosialin
Cda	1885	186	10.13	cytidine deaminase
Col14a1	6937	484	14.32	collagen, type XIV, alpha 1
Crabp1	4545	83	54.57	cellular retinoic acid binding protein 1
Ctla2a	1113	138	8.06	cytotoxic T lymphocyte-associated protein 2 alpha
Cyp3a9	3327	572	5.82	cytochrome P450, family 3, subfamily a, polypeptide 9
Dusp5	6841	989	6.92	dual specificity phosphatase 5
Egr1	18179	1965	9.25	early growth response 1
Egr2	1675	197	8.51	early growth response 2
Emp2	2241	286	7.84	epithelial membrane protein 2
Fabp5	4738	104	45.57	fatty acid binding protein 5, epidermal
Fam26e	1361	241	5.66	family with sequence similarity 26, member E
Fos	7858	91	86.82	FBJ osteosarcoma oncogene
Gdf15	1585	236	6.73	growth differentiation factor 15
Gprc5a	5078	910	5.58	G protein-coupled receptor, family C, group 5, member A
Grem2	1938	92	20.97	gremlin 2
Il13ra2	2665	117	22.78	interleukin 13 receptor, alpha 2
Il17re	3444	164	20.97	interleukin 17 receptor E
Il1r2	5078	133	38.32	interleukin 1 receptor, type II
Il1rl1	16271	1418	11.47	interleukin 1 receptor-like 1
Itgb1	7033	91	77.71	integrin, beta 1
LOC100361389	2610	252	10.34	hypothetical protein LOC100361389
LOC290595	100025	3468	28.84	hypothetical gene supported by AF152002
Lrit1	1618	99	16.34	leucine-rich repeat, immunoglob.-like and transmembr. domains 1
Mfap5	26987	2684	10.06	microfibrillar associated protein 5
Mgst1	2610	331	7.89	microsomal glutathione S-transferase 1
Msln	42055	4738	8.88	mesothelin
Nppb	1323	80	16.45	natriuretic peptide B
Nr4a1	10960	657	16.68	nuclear receptor subfamily 4, group A, member 1
Nxph3	1629	111	14.72	neurexophilin 3
Pard3	3281	92	35.51	par-3 (partitioning defective 3) homolog
Pla2g2a	8780	719	12.21	phospholipase A2, group IIA
Ptgs1	7181	425	16.91	prostaglandin-endoperoxide synthase 1
Rgs2	2180	388	5.62	regulator of G-protein signaling 2
Sepp1	1710	118	14.52	selenoprotein P, plasma, 1
Slc22a18	3350	217	15.45	solute carrier family 22, member 18
Slc6a4	1872	120	15.56	solute carrier family 6 (neurotransmitter transp., serotonin), memb.4
Slpi	1287	125	10.27	secretory leukocyte peptidase inhibitor
Sncg	6383	152	41.93	synuclein, gamma (breast cancer-specific protein 1)
Steap4	1898	263	7.21	STEAP family member 4
Sulf2	1531	141	10.85	sulfatase 2
Thbs2	7804	519	15.03	thrombospondin 2
Tmem45a	3984	739	5.39	transmembrane protein 45A

Table S3B

Higher signal strength in lung fibroblasts than endothelial cells

Symbol	EC	Fb	Fb:EC	GeneName
Acta2	1795	11747	6.54	smooth muscle alpha-actin
Adarb1	309	1771	5.74	adenosine deaminase, RNA-specific, B1
Aoc3	904	5634	6.23	amine oxidase, copper containing 3 (vascular adhesion protein 1)
Aox1	2452	9742	3.97	aldehyde oxidase 1
C1ql3	258	1479	5.74	complement component 1, q subcomponent-like 3
C1qtnf7	396	1585	4.00	C1q and tumor necrosis factor related protein 7
Ccdc37	3083	15608	5.06	coiled-coil domain containing 37
Ccl19	326	1771	5.43	chemokine (C-C motif) ligand 19
Cd24	278	1479	5.31	CD24 molecule
Cdc42bpg	385	1510	3.92	CDC42 binding protein kinase gamma (DMPK-like)
Ces1a	1226	7804	6.36	carboxylesterase 1A
Ces1d	191	3875	20.25	carboxylesterase 1D
Cfh	284	1641	5.78	complement factor H
Clk1	2957	10514	3.56	CDC-like kinase 1
Cx3cl1	17805	128375	7.21	chemokine (C-X3-C motif) ligand 1
Dock8	211	1046	4.96	dedicator of cytokinesis 8
Enpep	491	4939	10.06	glutamyl aminopeptidase
Fam46a	1820	6383	3.51	family with sequence similarity 46, member A
Fhl1	3281	19893	6.06	four and a half LIM domains 1
Fxyd1	241	1885	7.84	FXYD domain-containing ion transport regulator 1
Gna14	734	3259	4.44	guanine nucleotide binding protein, alpha 14
Gpm6b	820	3040	3.71	glycoprotein m6b
Lcat	309	1209	3.92	lecithin cholesterol acyltransferase
LOC100363229	1448	4939	3.41	hypothetical protein LOC100363229
LOC100364218	2336	10735	4.59	hypothetical protein LOC100364218
LOC100365120	2165	9476	4.38	hypothetical protein LOC100365120
LOC100366054	2539	11191	4.41	Da1-10-like
LOC499330	468	1924	4.11	similar to Nicotinamide riboside kinase 1
LOC500846	1399	4673	3.34	hypothetical protein LOC500846
LOC690463	501	1675	3.34	hypothetical protein LOC690463
Mrvi1	396	6608	16.68	murine retrovirus integration site 1 homolog
Myh1	1771	7591	4.29	myosin, heavy polypeptide 1, skeletal muscle, adult
Myh10	855	2856	3.34	myosin, heavy chain 10, non-muscle
Myh8	2020	9027	4.47	myosin, heavy chain 8, skeletal muscle
Myl9	537	2272	4.23	myosin, light chain 9, regulatory
Nacc2	1520	5185	3.41	nucleus accumbens assoc.2, BEN and BTB (POZ) domain contain.
Nme4	613	2336	3.81	non-metastatic cells 4, protein expressed in
Npff	317	1448	4.56	neuropeptide FF-amide peptide precursor
Pcp4l1	347	2135	6.15	Purkinje cell protein 4-like 1
Pear1	329	1235	3.76	platelet endothelial aggregation receptor 1
Pik3r1	471	2210	4.69	phosphoinositide-3-kinase, regulatory subunit 1 (alpha)
Ptn	194	1136	5.86	Pleiotrophin
Reep6	380	1399	3.68	receptor accessory protein 6
RGD1359529	6165	25006	4.06	similar to chromosome 1 open reading frame 63
Sfxn4	592	2353	3.97	sideroflexin 4
Sirpa	239	2020	8.46	signal-regulatory protein alpha
Spint2	2336	8599	3.68	serine peptidase inhibitor, Kunitz type, 2
Tcf7l2	760	3040	4.00	transcription factor 7-like 2 (T-cell specific, HMG-box)
Tm4sf1	6747	38166	5.66	transmembrane 4 L six family member 1
Tnfrsf11b	564	2487	4.41	tumor necrosis factor receptor superfamily, member 11b

Table S3C

Higher mRNA signal strength in AS-Tspan8-TEX than endothelial cells

Symbol	EC	Tspan8-TEX	Tspan8-TEX:EC[a]	GeneName
A2m	69	35879	519.46	alpha-2-macroglobulin
Adamts9	82	10663	129.81	a disintegrin-like and metalloprotease with thrombospondin type 1 motif, 9
Adora1	91	1098	12.13	adenosine A1 receptor
Afp	70	2576	36.78	alpha-fetoprotein
Akr7a3	84	1700	20.13	aldo-keto reductase family 7, member A3 (aflatoxin aldehyde reductase)
Aldh1a1	78	13034	166.58	aldehyde dehydrogenase 1 family, member A1
Anxa3	91	2750	30.38	annexin A3
Apoc4	92	2684	29.04	apolipoprotein C-IV
Apoe	228	23334	102.55	apolipoprotein E
Arg2	91	5009	55.34	arginase type II
Arhgap8	307	3605	11.76	Rho GTPase activating protein 8
Atf3	478	10300	21.56	activating transcription factor 3
Atp6v0a4	95	1177	12.39	ATPase, H+ transporting, lysosomal V0 subunit A4
B3gnt7	98	1500	15.35	UDP-GlcNAc:betaGal beta-1,3-N-acetylglucosaminyltransferase 7
Bambi	223	3304	14.83	BMP and activin membrane-bound inhibitor
Bex1	88	9418	106.98	brain expressed, X-linked 1
Bpifb6	66	1052	16.00	BPI fold containing family B, member 6
C2cd4d	68	4624	67.88	C2 calcium-dependent domain containing 4D
C3	4482	91461	20.41	complement component 3
Cacna1h	90	4013	44.65	calcium channel, voltage-dependent, T type, alpha 1H subunit
Ccdc11	77	2403	31.35	coiled-coil domain containing 11
Ccl20	148	9253	62.50	chemokine (C-C motif) ligand 20
Cd1d1	239	2558	10.71	CD1d1 molecule
Cd59	194	2345	12.09	CD59 molecule, complement regulatory protein
Cd99l2	80	3094	38.46	CD99 molecule-like 2
Cecr5	383	5096	13.32	cat eye syndrome chromosome region, candidate 5 homolog
Cfi	134	23171	172.45	complement factor I
Chchd10	242	31253	129.04	coiled-coil-helix-coiled-coil-helix domain containing 10
Chdh	94	11192	118.61	choline dehydrogenase
Cldn2	69	4612	66.78	claudin 2
Clec10a	73	2372	32.71	C-type lectin domain family 10, member A
Clec12a	181	2656	14.67	C-type lectin domain family 12, member A
Cmbl	78	1747	22.32	carboxymethylenebutenolidase homolog
Cpz	484	17880	36.91	carboxypeptidase Z
Crabp2	458	9378	20.46	cellular retinoic acid binding protein 2
Crlf1	207	4940	23.92	cytokine receptor-like factor 1
Cryba4	78	2369	30.49	crystallin, beta A4
Csrnp3	79	3819	48.13	cysteine-serine-rich nuclear protein 3
Csrp2	1652	66953	40.53	cysteine and glycine-rich protein 2
Ctsc	100	5349	53.63	cathepsin C
Cxcl2	108	3327	30.70	chemokine (C-X-C motif) ligand 2
Dclk2	86	1311	15.20	doublecortin-like kinase 2
Ddit4l	150	2173	14.47	DNA-damage-inducible transcript 4-like
Defa10	115	1205	10.45	defensin alpha 10
Defa7	181	4452	24.59	defensin alpha 7
Dhh	72	1140	15.84	desert hedgehog
Efhc2	81	2419	29.86	EF-hand domain (C-terminal) containing 2
Egln3	362	5914	16.34	EGL nine homolog 3
Eid2	80	2329	28.95	EP300 interacting inhibitor of differentiation 2
Elf3	160	1614	10.10	E74-like factor 3
Emid1	78	1181	15.09	EMI domain containing 1
Enpp2	78	3959	50.59	ectonucleotide pyrophosphatase/phosphodiesterase 2
Enpp4	180	1892	10.52	ectonucleotide pyrophosphatase/phosphodiesterase 4
Entpd2	99	3493	35.27	ectonucleoside triphosphate diphosphohydrolase 2
Epha4	129	1324	10.27	Eph receptor A4
Eps8l2	105	1231	11.68	EPS8-like 2
Etnk2	98	2172	22.09	ethanolamine kinase 2
F5	77	4186	54.62	coagulation factor V (proaccelerin, labile factor)

Table S3C continued

Symbol	EC	Tspan8-TEX	Tspan8-TEX:EC[a]	GeneName
Fabp12	62	1777	28.74	fatty acid binding protein 12
Fndc1	119	3837	32.35	fibronectin type III domain containing 1
Gadd45g	1585	31108	19.63	growth arrest and DNA-damage-inducible, gamma
Gch1	197	8482	43.12	GTP cyclohydrolase 1
Gckr	108	1172	10.89	glucokinase (hexokinase 4) regulator
Gdf15	1585	33931	21.41	growth differentiation factor 15
Gli1	84	1380	16.45	GLI family zinc finger 1
Glod5	152	13682	89.89	glyoxalase domain containing 5
Gprc5c	101	2020	19.98	G protein-coupled receptor, family C, group 5, member C
Gramd1b	523	6897	13.19	GRAM domain containing 1B
Gstt1	182	3051	16.74	glutathione S-transferase theta 1
Hap1	143	1931	13.50	huntingtin-associated protein 1
Hist1h2an	229	2423	10.58	histone cluster 1, H2an
Hist2h3c	1201	13639	11.36	histone cluster 2, H3c
Hopx	530	9509	17.94	HOP homeobox
Hoxb13	92	1410	15.36	homeo box B13
Hoxd9	73	1855	25.41	homeo box D9
Hpx	115	4197	36.63	Hemopexin
Htra3	93	1513	16.26	HtrA serine peptidase 3
Id2	372	16103	43.26	inhibitor of DNA binding 2
Igf2	247	4923	19.91	insulin-like growth factor 2
Igfbp2	77	1049	13.60	insulin-like growth factor binding protein 2
Igfbp5	88	3579	40.66	insulin-like growth factor binding protein 5
Il17rb	82	1073	13.06	interleukin 17 receptor B
Il6	357	5673	15.89	interleukin 6
Isg15	1541	17381	11.28	ISG15 ubiquitin-like modifier
Itga8	292	3104	10.63	integrin, alpha 8
Itm2a	159	2980	18.78	integral membrane protein 2A
Itpka	170	2495	14.67	inositol-trisphosphate 3-kinase A
Klhdc8a	71	7835	110.33	kelch domain containing 8A
Krt19	74	12164	165.46	keratin 19
Lcn2	4939	372071	75.33	lipocalin 2
Lgi3	87	1846	21.27	leucine-rich repeat LGI family, member 3
LOC100359930	512	12595	24.60	Cyp2s1 protein-like
LOC100364559	84	3679	43.56	gene model 691, (NCBI)-like
LOC100364854	74	1253	16.93	similar to RIKEN cDNA A430107P09 gene-like
LOC498829	402	7779	19.37	Ab2-143
LOC678766	148	26744	180.64	similar to ras homolog gene family, member U
LOC679126	236	3692	15.67	similar to SMAD-interacting zinc finger protein 2
LOC689756	201	2091	10.41	hypothetical protein LOC689756
LOC691293	103	1369	13.35	similar to reproductive homeobox on chromosome X, 7
Lphn1	592	7119	12.02	latrophilin 1
Lypd2	644	24664	38.32	Ly6/Plaur domain containing 2
Mark1	68	2378	35.15	MAP/microtubule affinity-regulating kinase 1
Mat1a	79	1443	18.32	methionine adenosyltransferase I, alpha
Mcam	83	3226	38.74	melanoma cell adhesion molecule
Mfsd6	452	10121	22.39	major facilitator superfamily domain containing 6
Mgst1	2610	35632	13.65	microsomal glutathione S-transferase 1
Mmd2	70	1142	16.42	monocyte to macrophage differentiation-associated 2
Mrap	84	12246	146.02	melanocortin 2 receptor accessory protein
Myh3	125	3320	26.48	myosin, heavy chain 3, skeletal muscle, embryonic
Npl	71	1168	16.57	N-acetylneuraminate pyruvate lyase
Npr1	169	2233	13.22	natriuretic peptide rec.A/guanylate cyclase A (atrionatriuretic peptide receptor A)
Nr0b2	80	1231	15.41	nuclear receptor subfamily 0, group B, member 2
Nrip3	132	4012	30.49	nuclear receptor interacting protein 3
Nrp1	76	1329	17.46	neuropilin 1
Nxph1	74	6544	89.02	neurexophilin 1
Obsl1	193	1927	10.00	obscurin-like 1
Olfml2a	95	6430	67.68	olfactomedin-like 2A

Table S3C continued

Symbol	EC	Tspan8-TEX	Tspan8-TEX:EC[a]	GeneName
Osgin1	296	3327	11.24	oxidative stress induced growth inhibitor 1
P2ry6	252	3194	12.65	pyrimidinergic receptor P2Y, G-protein coupled, 6
Pcbd1	181	3517	19.43	pterin-4α-carbinolamine dehydrat./dimeriz. cofactor of hepatoc.nuclear factor1α
Pcsk9	101	8055	79.66	proprotein convertase subtilisin/kexin type 9
Pde9a	69	1456	21.22	phosphodiesterase 9A
Pdpn	194	35861	184.84	Podoplanin
Pitx1	505	19860	39.33	paired-like homeodomain 1
Plagl1	75	3398	45.26	pleiomorphic adenoma gene-like 1
Plat	488	6563	13.45	plasminogen activator, tissue
Plch1	98	1557	15.83	phospholipase C, eta 1
Plekhb1	101	1777	17.57	pleckstrin homology domain containing, family B (evectins) member 1
Plxnd1	218	5331	24.43	plexin D1
Pnkd	209	9982	47.67	paroxysmal nonkinesiogenic dyskinesia
Podxl	84	7012	83.04	podocalyxin-like
Prkar1b	383	4722	12.34	protein kinase, cAMP dependent regulatory, type I, beta
Prkch	138	3051	22.09	protein kinase C, eta
Prtg	76	1310	17.33	Protogenin
Ptges	170	2241	13.18	prostaglandin E synthase
Ptn	194	12247	63.12	Pleiotrophin
Qrich2	97	1958	20.18	glutamine rich 2
Rac2	72	5616	78.53	ras-rel. C3 botulinum toxin substrate 2 (rho fam., small GTP bind. protein Rac2)
Rap2ip	231	4436	19.23	Rap2 interacting protein
Raph1	117	1759	15.03	Ras association (RalGDS/AF-6) and pleckstrin homology domains 1
Rbp4	79	2329	29.36	retinol binding protein 4, plasma
Rbpms	66	1193	18.01	RNA binding protein with multiple splicing
Rerg	82	2436	29.65	RAS-like, estrogen-regulated, growth-inhibitor
RGD1304580	76	1399	18.51	similar to Hypothetical protein MGC38513
RGD1308305	82	1464	17.82	similar to RIKEN cDNA 5430400H23
RGD1309676	139	4469	32.13	similar to RIKEN cDNA 5730469M10
RGD1560394	97	5753	59.31	RGD1560394
RGD1561113	434	7063	16.29	similar to Hypothetical UPF0184 protein C9orf16 homolog
RGD1564380	67	12247	183.56	similar to BC049730 protein
RGD1565166	102	2233	21.93	similar to MGC45438 protein
Rgn	71	1537	21.64	regucalcin (senescence marker protein-30)
Rhbdl2	76	2253	29.81	rhomboid, veinlet-like 2
Ril	1808	23508	13.00	reversion induced LIM gene
Rln1	74	4991	67.43	relaxin 1
Ropn1l	108	1244	11.47	rhophilin associated tail protein 1-like
Rps6ka1	120	5293	44.02	ribosomal protein S6 kinase polypeptide 1
Scarb1	734	14664	19.97	scavenger receptor class B, member 1
Scd1	443	12161	27.47	stearoyl-Coenzyme A desaturase 1
Scgb1c1	145	1620	11.18	secretoglobin, family 1C, member 1
Sec14l2	523	5279	10.10	SEC14-like 2
Selp	272	3330	12.22	selectin P
Serinc5	101	2427	24.00	serine incorporator 5
Slc29a4	187	57292	305.72	solute carrier family 29 (nucleoside transporters), member 4
Slc2a5	195	1980	10.14	solute carrier family 2 (facilitated glucose/fructose transporter), member 5
Slc39a8	72	3641	50.92	solute carrier family 39 (zinc transporter), member 8
Slc4a3	676	11273	16.69	solute carrier family 4 (anion exchanger), member 3
Slc7a11	498	5296	10.63	solute carrier family 7 (anionic amino acid transporter light chain), member 11
Snph	84	1474	17.57	Syntaphilin
Sostdc1	78	69288	891.64	sclerostin domain containing 1
Sox7	103	1372	13.38	SRY (sex determining region Y)-box 7
Spon1	83	4524	54.32	spondin 1, extracellular matrix protein
Srd5a1	165	6208	37.53	steroid-5α-reduct., α polypeptide 1 (3-oxo-5 α -steroid δ4-dehydrogenase α1)
Stbd1	212	3281	15.46	starch binding domain 1
Stc1	78	1892	24.18	stanniocalcin 1
Tead2	474	5842	12.31	TEA domain family member 2
Tesc	73	2470	33.83	Tescalcin

Table S3C continued

Symbol	EC	Tspan8-TEX	Tspan8-TEX:EC[a]	GeneName
Tfr2	78	1664	21.26	transferrin receptor 2
Tfrc	1541	54038	35.06	transferrin receptor
Timd2	282	3409	12.09	T-cell immunoglobulin and mucin domain containing 2
Tjp3	98	3051	31.24	tight junction protein 3
Tmem176a	69	4112	59.54	transmembrane protein 176A
Tmem176b	87	22699	259.63	transmembrane protein 176B
Tnfrsf21	80	8570	106.53	tumor necrosis factor receptor superfamily, member 21
Tnnt2	290	5277	18.19	troponin T type 2
Tppp3	100	4657	46.70	tubulin polymerization-promoting protein family member 3
Trpv4	102	2218	21.78	transient receptor potential cation channel, subfamily V, member 4
Tspan12	64	3849	60.56	tetraspanin 12
Tspan13	239	2858	11.96	tetraspanin 13
Tspan8	81	1194	14.74	tetraspanin 8
Tst	443	5067	11.45	thiosulfate sulfurtransferase
Tubb2b	66	13465	203.23	tubulin, beta 2B class Iib
Tubb3	191	2071	10.82	tubulin, beta 3 class III
Tubg2	87	2651	30.32	tubulin, gamma 2
Uchl1	83	18372	222.12	ubiquitin carboxyl-terminal esterase L1 (ubiquitin thiolesterase)
Vnn1	92	2135	23.10	vanin 1
Vtcn1	93	2647	28.44	V-set domain containing T cell activation inhibitor 1
Wif1	95	1090	11.47	Wnt inhibitory factor 1
Wnt4	223	2490	11.17	wingless-type MMTV integration site family, member 4
Zwint	2837	33399	11.77	ZW10 interactor

[a] AS-Tspan8-TEX mRNA signal ≥10-fold higher than in EC

Table S3D

Higher mRNA signal strength in AS-Tspan8-TEX than lung fibroblasts

Symbol	Fb	Tspan8-TEX	Tspan8-TEX:Fb[b]	GeneName
A2m	68	35879	526.71	alpha-2-macroglobulin
Adamts9	66	10663	162.05	a disintegrin-like and metalloprotease with thrombospondin type 1 motif, 9
Adora1	100	1098	11.01	adenosine A1 receptor
Adora2b	413	8690	21.04	adenosine A2B receptor
Afp	66	2576	38.88	alpha-fetoprotein
Ak4	84	2820	33.40	adenylate kinase 4
Akr7a3	91	1700	18.78	aldo-keto reductase family 7, member A3 (aflatoxin aldehyde reductase)
Aldh1a1	100	13034	129.79	aldehyde dehydrogenase 1 family, member A1
Aldh1l1	98	2289	23.27	aldehyde dehydrogenase 1 family, member L1
Aldoc	2435	41922	17.21	aldolase C, fructose-bisphosphate
Anxa3	239	2750	11.51	annexin A3
Apoc4	96	2684	27.86	apolipoprotein C-IV
Apoe	391	23334	59.72	apolipoprotein E
Areg	89	1314	14.72	Amphiregulin
Arg2	91	5009	55.34	arginase type II
Arhgap8	102	3605	35.40	Rho GTPase activating protein 8
Atf3	161	10300	64.01	activating transcription factor 3
Atp6v0a4	96	1177	12.30	ATPase, H+ transporting, lysosomal V0 subunit A4
B3gnt7	92	1500	16.34	UDP-GlcNAc:betaGal beta-1,3-N-acetylglucosaminyltransferase 7
Bambi	145	3304	22.79	BMP and activin membrane-bound inhibitor, homolog
Bex1	79	9418	119.53	brain expressed, X-linked 1
Bpifb6	63	1052	16.67	BPI fold containing family B, member 6
Btg2	1152	25183	21.86	BTG family, member 2
C2cd4d	77	4624	59.92	C2 calcium-dependent domain containing 4D
C3	4640	91461	19.71	complement component 3
Cacna1h	83	4013	48.19	calcium channel, voltage-dependent, T type, alpha 1H subunit
Ccdc11	76	2403	31.79	coiled-coil domain containing 11
Ccdc91	96	1161	12.05	coiled-coil domain containing 91
Ccl20	82	9253	113.43	chemokine (C-C motif) ligand 20
Cd59	66	2345	35.39	CD59 molecule, complement regulatory protein
Cd99l2	79	3094	39.27	CD99 molecule-like 2
Ceacam10	209	4909	23.45	carcinoembryonic antigen-related cell adhesion molecule 10
Cfi	175	23171	132.52	complement factor I
Chchd10	224	31253	139.27	coiled-coil-helix-coiled-coil-helix domain containing 10
Chdh	94	11192	118.61	choline dehydrogenase
Cldn2	72	4612	64.50	claudin 2
Clec10a	70	2372	33.86	C-type lectin domain family 10, member A
Clec12a	98	2656	27.00	C-type lectin domain family 12, member A
Cmbl	79	1747	22.17	carboxymethylenebutenolidase homolog
Col15a1	360	11585	32.22	collagen, type XV, alpha 1
Cpz	133	17880	134.93	carboxypeptidase Z
Crabp2	596	9378	15.73	cellular retinoic acid binding protein 2
Crlf1	124	4940	39.68	cytokine receptor-like factor 1
Cryba4	88	2369	26.91	crystallin, beta A4
Csf3	282	3238	11.48	colony stimulating factor 3 (granulocyte)
Csrnp3	76	3819	50.52	cysteine-serine-rich nuclear protein 3
Csrp2	685	66953	97.74	cysteine and glycine-rich protein 2
Ctsc	70	5349	76.38	cathepsin C
Cxcl2	94	3327	35.51	chemokine (C-X-C motif) ligand 2
Dclk2	105	1311	12.43	doublecortin-like kinase 2
Ddit4l	146	2173	14.88	DNA-damage-inducible transcript 4-like
Defa10	118	1205	10.23	defensin alpha 10
Defa7	172	4452	25.82	defensin alpha 7
Dhh	72	1140	15.84	desert hedgehog
Dnajc12	84	1073	12.79	DnaJ (Hsp40) homolog, subfamily C, member 12
Dusp22	111	1133	10.17	dual specificity phosphatase 22
Efhc2	64	2419	37.79	EF-hand domain (C-terminal) containing 2
Egln3	370	5914	16.00	EGL nine homolog 3

Table S3D continued

Symbol	Fb	Tspan8-TEX	Tspan8-TEX:Fb[b]	GeneName
Egr1	1965	35000	17.82	early growth response 1
Eid2	79	2329	29.56	EP300 interacting inhibitor of differentiation 2
Emid1	75	1181	15.84	EMI domain containing 1
Emp2	286	17562	61.40	epithelial membrane protein 2
Enc1	572	10775	18.84	ectodermal-neural cortex 1
Enpp2	143	3959	27.68	ectonucleotide pyrophosphatase/phosphodiesterase 2
Entpd2	87	3493	39.96	Ectonucleoside triphosphate diphosphohydrolase 2
Entpd6	136	1777	13.04	Ectonucleoside triphosphate diphosphohydrolase 6
Eps8l2	94	1231	13.04	EPS8-like 2
Etnk2	119	2172	18.32	ethanolamine kinase 2
F5	84	4186	49.57	coagulation factor V (proaccelerin, labile factor)
Fabp12	66	1777	26.82	fatty acid binding protein 12
Fabp5	104	39375	378.73	fatty acid binding protein 5, epidermal
Fam173a	96	1003	10.48	family with sequence similarity 173, member A
Fndc1	67	3837	57.51	fibronectin type III domain containing 1
Fos	91	6274	69.31	FBJ osteosarcoma oncogene
Gadd45g	505	31108	61.61	growth arrest and DNA-damage-inducible, gamma
Gch1	81	8482	104.70	GTP cyclohydrolase 1
Gckr	74	1172	15.94	glucokinase (hexokinase 4) regulator
Gdf15	236	33931	144.04	growth differentiation factor 15
Gli1	85	1380	16.22	GLI family zinc finger 1
Gli2	77	1056	13.69	GLI family zinc finger 2
Glod5	85	13682	160.90	glyoxalase domain containing 5
Gngt2	98	3746	38.09	guanine nucleotide binding protein, gamma transduc.activ.polypept.2
Gprc5c	93	2020	21.71	G protein-coupled receptor, family C, group 5, member C
Gramd1b	523	6897	13.19	GRAM domain containing 1B
Hap1	177	1931	10.89	huntingtin-associated protein 1
Hes1	133	2974	22.44	hairy and enhancer of split 1
Hopx	592	9509	16.06	HOP homeobox
Hoxb13	74	1410	19.18	homeo box B13
Hoxd9	64	1855	29.19	homeo box D9
Hpx	177	4197	23.67	Hemopexin
Hspa4l	108	1380	12.82	heat shock protein 4-like
Htra3	82	1513	18.55	HtrA serine peptidase 3
Id1	267	2939	11.01	inhibitor of DANN binding 1
Id2	622	16103	25.90	inhibitor of DANN binding 2
Igf2	142	4923	34.67	insulin-like growth factor 2
Igfbp2	68	1049	15.51	insulin-like growth factor binding protein 2
Igfbp5	80	3579	44.49	insulin-like growth factor binding protein 5
Il17rb	82	1073	13.15	interleukin 17 receptor B
Il33	108	2337	21.71	interleukin 33
Il6	135	5673	41.93	interleukin 6
Itgb1	91	5185	57.28	integrin, beta 1
Itm2a	79	2980	37.55	integral membrane protein 2A
Itpka	223	2495	11.20	inositol-trisphosphate 3-kinase A
Klhdc8a	71	7835	111.10	kelch domain containing 8A
Krt19	68	12164	178.57	keratin 19
Lcn2	2937	372071	126.70	lipocalin 2
Lgi3	86	1846	21.42	leucine-rich repeat LGI family, member 3
LOC100359930	92	12595	137.24	Cyp2s1 protein-like
LOC100361389	252	4156	16.46	hypothetical protein LOC100361389
LOC100364559	74	3679	49.69	gene model 691, (NCBI)-like
LOC100364854	68	1253	18.40	similar to RIKEN cDNA A430107P09 gene-like
LOC498829	360	7779	21.64	Ab2-143
LOC500959	4482	59889	13.36	triosephosphate isomerase
LOC678766	71	26744	376.62	similar to ras homolog gene family, member U
LOC679126	202	3692	18.25	similar to SMAD-interacting zinc finger protein 2
LOC689756	107	2091	19.57	hypothetical protein LOC689756
LOC691293	98	1369	13.92	similar to reproductive homeobox on chromosome X, 7

Table S3D continued

Symbol	Fb	Tspan8-TEX	Tspan8-TEX:Fb[b]	GeneName
Lphn1	680	7119	10.47	latrophilin 1
Ly6e	106	1319	12.43	lymphocyte antigen 6 complex, locus E
Lypd2	910	24664	27.10	Ly6/Plaur domain containing 2
Mark1	77	2378	30.81	MAP/microtubule affinity-regulating kinase 1
Mat1a	64	1443	22.55	methionine adenosyltransferase I, alpha
Mbp	70	1097	15.77	myelin basic protein
Mcam	91	3226	35.40	melanoma cell adhesion molecule
Mfsd6	69	10121	147.54	major facilitator superfamily domain containing 6
Mgst1	331	35632	107.70	microsomal glutathione S-transferase 1
Mmd2	73	1142	15.75	monocyte to macrophage differentiation-associated 2
Mrap	84	12246	146.02	melanocortin 2 receptor accessory protein
Myh3	121	3320	27.42	myosin, heavy chain 3, skeletal muscle, embryonic
Npl	72	1168	16.34	N-acetylneuraminate pyruvate lyase
Nppb	80	10056	124.99	natriuretic peptide B
Npr1	199	2233	11.20	natriuretic peptide rec.A/guanylate cyclase A (atrionatriuretic peptide receptor A)
Nr0b2	83	1231	14.78	nuclear receptor subfamily 0, group B, member 2
Nr4a1	657	7132	10.85	nuclear receptor subfamily 4, group A, member 1
Nrarp	147	1496	10.17	Notch-regulated ankyrin repeat protein
Nrip3	133	4012	30.27	nuclear receptor interacting protein 3
Nrp1	79	1329	16.75	neuropilin 1
Nxph1	70	6544	94.10	neurexophilin 1
Olfml2a	163	6430	39.41	olfactomedin-like 2A
Osap	1965	21545	10.97	ovary-specific acidic protein
Osgin1	324	3327	10.27	oxidative stress induced growth inhibitor 1
P2ry6	142	3194	22.49	pyrimidinergic receptor P2Y, G-protein coupled, 6
Pappa	143	1564	10.93	pregnancy-associated plasma protein A
Pard3	92	3225	34.90	par-3 (partitioning defective 3) homolog
Pcbd1	108	3517	32.68	pterin-4α-carbinolamine dehydrat/dimeriz. cofactor of hepatoc. nuclear factor1α
Pcsk9	78	8055	103.66	proprotein convertase subtilisin/kexin type 9
Pde9a	77	1456	18.86	phosphodiesterase 9A
Pdpn	86	35861	415.91	Podoplanin
Pdzrn3	600	11126	18.53	PDZ domain containing RING finger 3
Pitx1	787	19860	25.24	paired-like homeodomain 1
Pla2g16	1136	16103	14.17	phospholipase A2, group XVI
Plagl1	78	3398	43.72	pleiomorphic adenoma gene-like 1
Plch1	87	1557	17.94	phospholipase C, eta 1
Plxnd1	443	5331	12.04	plexin D1
Pnkd	695	9982	14.37	paroxysmal nonkinesiogenic dyskinesia
Podxl	74	7012	95.39	podocalyxin-like
Ppp1r36	143	1641	11.47	protein phosphatase 1, regulatory subunit 36
Prkar1b	458	4722	10.30	protein kinase, cAMP dependent regulatory, type I, beta
Prkch	134	3051	22.71	protein kinase C, eta
Prtg	73	1310	17.94	Protogenin
Ptges	105	2241	21.26	prostaglandin E synthase
Ptn	1136	12247	10.78	Pleiotrophin
Qrich2	131	1958	14.98	glutamine rich 2
Rab15	87	1094	12.60	RAB15, member RAS oncogene family
Rac2	74	5616	75.86	ras-rel. C3 botulinum toxin substrate 2 (rho family, small GTP binding pr. Rac2)
Rap2ip	77	4436	57.48	Rap2 interacting protein
Raph1	146	1759	12.04	Ras association (RalGDS/AF-6) and pleckstrin homology domains 1
Rbp4	71	2329	33.03	retinol binding protein 4, plasma
Rbpms	68	1193	17.64	RNA binding protein with multiple splicing
Rerg	70	2436	35.02	RAS-like, estrogen-regulated, growth-inhibitor
RGD1304580	99	1399	14.13	similar to Hypothetical protein MGC38513
RGD1304982	180	1802	10.02	similar to RIKEN cDNA 2810025M15
RGD1308305	101	1464	14.47	similar to RIKEN cDNA 5430400H23
RGD1309676	156	4469	28.56	similar to RIKEN cDNA 5730469M10
RGD1560394	74	5753	78.26	RGD1560394
RGD1561113	465	7063	15.20	similar to Hypothetical UPF0184 protein C9orf16 homolog

Table S3D continued

Symbol	Fb	Tspan8-TEX	Tspan8-TEX:Fb[b]	GeneName
RGD1564380	67	12247	183.56	similar to BC049730 protein
RGD1565166	68	2233	32.79	similar to MGC45438 protein
Rgn	75	1537	20.47	regucalcin (senescence marker protein-30)
Rgs2	388	8780	22.63	regulator of G-protein signaling 2
Rhbdl2	75	2253	30.01	rhomboid, veinlet-like 2
Rhob	331	4692	14.18	ras homolog gene family, member B
Ril	1296	23508	18.14	reversion induced LIM gene
Rln1	74	4991	67.43	relaxin 1
Rps6ka1	90	5293	58.89	ribosomal protein S6 kinase polypeptide 1
Scarb1	676	14664	21.71	scavenger receptor class B, member 1
Scd1	244	12161	49.87	stearoyl-Coenzyme A desaturase 1
Scgb1c1	141	1620	11.49	secretoglobin, family 1C, member 1
Selp	73	3330	45.93	selectin P
Serinc5	72	2427	33.71	serine incorporator 5
Sh3tc1	97	1319	13.60	SH3 domain and tetratricopeptide repeats 1
Slc29a4	120	57292	476.41	solute carrier family 29 (nucleoside transporters), member 4
Slc2a5	78	1980	25.48	solute carrier family 2 (facilitated glucose/fructose transporter), member 5
Slc39a8	80	3641	45.57	solute carrier family 39 (zinc transporter), member 8
Slc7a11	154	5296	34.31	solute carrier family 7 (anionic amino acid transporter light chain), member 11
Slpi	125	1885	15.03	secretory leukocyte peptidase inhibitor
Slurp1	124	2106	17.03	secreted Ly6/Plaur domain containing 1
Snph	77	1474	19.10	Syntaphilin
Sostdc1	82	69288	843.54	sclerostin domain containing 1
Sox13	81	1241	15.32	SRY (sex determining region Y)-box 13
Sox7	111	1372	12.40	SRY (sex determining region Y)-box 7
Spon1	94	4524	48.28	spondin 1, extracellular matrix protein
Srd5a1	148	6208	41.93	steroid-5α-reductase, α polypeptide 1 (3-oxo-5α -steroid δ 4-dehydrog.α1)
Stau2	82	1292	15.73	staufen, RNA binding protein, homolog 2
Stc1	83	1892	22.88	stanniocalcin 1
Tesc	78	2470	31.78	Tescalcin
Tfr2	77	1664	21.56	transferrin receptor 2
Tfrc	1885	54038	28.67	transferrin receptor
Thnsl2	84	1283	15.19	threonine synthase-like 2
Tjp3	108	3051	28.35	tight junction protein 3
Tmem176a	69	4112	59.95	Transmembrane protein 176A
Tmem176b	101	22699	224.46	Transmembrane protein 176B
Tnfrsf21	76	8570	113.38	tumor necrosis factor receptor superfamily, member 21
Tnnt2	201	5277	26.27	troponin T type 2 (cardiac)
Tppp3	117	4657	39.81	tubulin polymerization-promoting protein family member 3
Trib3	194	2639	13.60	tribbles homolog 3
Trpv4	99	2218	22.39	transient receptor potential cation channel, subfamily V, member 4
Tspan12	72	3849	53.46	tetraspanin 12
Tspan13	111	2858	25.64	tetraspanin 13
Tspan8	100	1194	11.89	tetraspanin 8
Tubb2b	65	13465	206.06	tubulin, beta 2B class IIb
Tubb3	96	2071	21.50	tubulin, beta 3 class III
Tubg2	76	2651	35.07	tubulin, gamma 2
Uchl1	78	18372	234.79	ubiquitin carboxyl-terminal esterase L1 (ubiquitin thiolesterase)
Vnn1	72	2135	29.86	vanin 1
Vtcn1	77	2647	34.30	V-set domain containing T cell activation inhibitor 1
Wnt4	97	2490	25.66	wingless-type MMTV integration site family, member 4
Zdhhc2	83	4000	48.36	zinc finger, DHHC-type containing 2
Zrsr1	74	1156	15.73	zinc finger (CCCH type), RNA binding motif and serine/arginine rich 1
Zwint	3259	33399	10.25	ZW10 interactor

[b] AS-Tspan8-TEX mRNA signal ≥10-fold higher than in Fb

Table S4

miRNA recovery in cells and TEX

Table S4A

Top miRNA in AS-Tspan8-TEX versus cells

name	AS-Tspan8-TEX[a]	name	AS-Tspan8-Cells[a]
let-7a	11501	let-7a	21915
let-7b	12220	let-7b	15247
let-7c	16054	let-7c	22129
let-7d	2728	let-7d	5248
let-7e	1445	let-7e	3349
let-7f	4337	let-7f	16370
let-7i	1131	let-7i	5160
miR-15b	4603	miR-10b	2105
miR-16	2404	miR-15b	6033
miR-21	4204	miR-16	13231
miR-22	3134	miR-21	69126
miR-23a	3015	miR-22	13479
miR-23b	1483	miR-23a	6814
miR-24	1372	miR-23b	2605
miR-25	1225	miR-24	3756
miR-26a	1680	miR-25	2107
miR-27a	749	miR-26a	6199
miR-27b	525	miR-26b	3714
miR-29a	5641	miR-27a	4491
miR-29c	741	miR-27b	1961
miR-30c	1570	miR-29a	19376
miR-32	1123	miR-29b	10663
miR-34a	1061	miR-29c	9494
miR-34c	947	miR-30a	1766
miR-93	995	miR-30b-5p	1452
miR-99a	1177	miR-30c	3170
miR-99b	767	miR-30e	1527
miR-103	912	miR-34a	6911
miR-107	1148	miR-34b	3144
miR-125a-5p	1626	miR-34c	3666
miR-125b-5p	9329	miR-93	1355
miR-130a	3464	miR-96	4485
miR-146a	722	miR-99a	3704
miR-182	615	miR-103	2196
miR-183	586	miR-106b	1533
miR-195	575	miR-107	2899
miR-196c	476	miR-125a-5p	2292
miR-204	645	miR-125b-5p	14845
miR-210	1590	miR-130a	8911
miR-221	2219	miR-142-3p	2960
miR-324-3p	747	miR-146a	2072
miR-346	504	miR-182	1261
miR-466b	3159	miR-183	1227
miR-466c	2793	miR-195	4560
miR-494	1905	miR-210	2384
miR-497	1182	miR-221	2125
miR-652	584	miR-450a	1357
miR-672	805	miR-494	1231
miR-764	754	miR-497	4695
miR-1224	1324	miR-1224	1628

[a] red: only in the top 50 in either TEX or cells

Table S4B

Significant differences in the miRNA recovery in AS-Tspan8-TEX versus cells

name	AS-Tspan8-TEX	AS-Tspan8-cells	AS-Tspan8-TEX : cells
miR-32	1123	148	7.608
miR-206	213	32	6.727
miR-207	414	64	6.498
miR-298	331	38	8.639
miR-328a	426	101	4.240
miR-328b-3p	357	37	9.783
miR-346	504	35	14.561
miR-465	277	23	11.894
miR-466b	3159	495	6.383
miR-466c	2793	367	7.613
miR-466d	247	34	7.239
miR-485	461	69	6.641
miR-504	314	27	11.753
miR-511	294	25	11.546
miR-672	805	109	7.419
miR-760-5p	311	108	2.879
miR-764	754	63	11.897
miR-1188-3p	247	30	8.168
miR-3591	216	26	8.162

Table S4B continued

name	AS-Tspan8-TEX	AS-Tspan8 cells	AS-Tspan8-cells : TEX
let-7e	1445	3349	2.317
let-7f	4337	16370	3.774
let-7i	1131	5160	4.562
miR-10a-5p	305	963	3.161
miR-10b	464	2105	4.536
miR-16	2404	13231	5.505
miR-21	4204	69126	16.441
miR-22	3134	13479	4.301
miR-23a	3015	6814	2.260
miR-24	1372	3756	2.737
miR-26a	1680	6199	3.689
miR-26b	417	3714	8.904
miR-27a	749	4491	5.992
miR-27b	525	1961	3.732
miR-29a	5641	19376	3.435
miR-29b	424	10663	25.167
miR-29c	741	9494	12.816
miR-30a	240	1766	7.354
miR-30b-5p	325	1452	4.466
miR-30c	1570	3170	2.019
miR-30e	148	1527	10.342
miR-34a	1061	6911	6.513
miR-34b	426	3144	7.380
miR-34c	947	3666	3.871
miR-96	364	4485	12.310
miR-98	181	901	4.973
miR-99a	1177	3704	3.148
miR-100	324	778	2.402
miR-101a	10	282	28.223
miR-101b	36	879	24.174
miR-103	912	2196	2.408
miR-106b	244	1533	6.280
miR-107	1148	2899	2.524
miR-128	139	430	3.099
miR-130a	3464	8911	2.572
miR-130b	348	917	2.639
miR-142-3p	94	2960	31.368
miR-146a	722	2072	2.871
miR-148b-3p	125	322	2.586
miR-151	307	793	2.582
miR-152	67	461	6.856
miR-182	615	1261	2.051
miR-183	586	1227	2.094
miR-185	99	404	4.091
miR-186	64	261	4.092
miR-195	575	4560	7.929
miR-212	60	257	4.292
miR-301a	18	391	21.994
miR-322	176	367	2.082
miR-324-5p	82	299	3.629
miR-344a-3p	58	279	4.817
miR-425	164	360	2.193
miR-450a	120	1357	11.294
miR-497	1182	4695	3.971
miR-503	36	316	8.719
miR-542-3p	28	542	19.378
miR-542-5p	172	532	3.100
miR-872	109	358	3.275
miR-1949	288	771	2.673
miR-3584-5p	156	533	3.419

Table S4C

The impact of Tspan8 on miRNA recovery in TEX: the top 50 miRNA in AS- and AS-Tspan8-TEX

name	AS-TEX[a]	name	AS-Tspan8-TEX[a]
let-7a	4914	let-7a	11501
let-7b	5214	let-7b	12220
let-7c	7569	let-7c	16054
let-7d	1213	let-7d	2728
let-7e	421	let-7e	1445
let-7f	3520	let-7f	4337
let-7i	957	let-7i	1131
miR-15b	951	miR-15b	4603
miR-16	2939	miR-16	2404
miR-21	10114	miR-21	4204
miR-22	743	miR-22	3134
miR-23a	2195	miR-23a	3015
miR-24	514	miR-23b	1483
miR-25	489	miR-24	1372
miR-26a	1504	miR-25	1225
miR-26b	984	miR-26a	1680
miR-29a	2898	miR-27a	749
miR-29c	670	miR-27b	525
miR-32	473	miR-29a	5641
miR-34a	1057	miR-29c	741
miR-34c	443	miR-30c	1570
miR-93	465	miR-32	1123
miR-99a	585	miR-34a	1061
miR-107	644	miR-34c	947
miR-125a-3p	510	miR-93	995
miR-125b-5p	4503	miR-99a	1177
miR-130a	774	miR-99b	767
miR-134	1690	miR-103	912
miR-150	1039	miR-107	1148
miR-188	4230	miR-125a-5p	1626
miR-195	1116	miR-125b-5p	9329
miR-196c	500	miR-130a	3464
miR-204	482	miR-146a	722
miR-211	4318	miR-182	615
miR-290	472	miR-183	586
miR-327	8758	miR-195	575
miR-328a	1313	miR-196c	476
miR-451	1291	miR-204	645
miR-466b	842	miR-210	1590
miR-483	8872	miR-221	2219
miR-494	14649	miR-324-3p	747
miR-497	1434	miR-346	504
miR-652	1723	miR-466b	3159
miR-672	491	miR-466c	2793
miR-874	659	miR-494	1905
miR-1224	12255	miR-497	1182
miR-3562	474	miR-652	584
miR-3564	957	miR-672	805
miR-3584-5p	1829	miR-764	754
miR-3593-3p	1846	miR-1224	1324

[a] red: only in the top 50 AS- or AS-Tspan8-TEX

Table S4D

Significant differences in miRNA recovery in ASML- versus ASML-Tspan8kd-TEX

name	**ASML-TEX**	**ASML-Tspan8kd-TEX**	**ASML : ASML-Tspan8kd-TEX**
let-7d	1148	377	3.042
miR-21	11462	5333	2.149
miR-24-1	35571	6978	5.097
miR-29c	1118	533	2.099
miR-30c-1	5715	1870	3.057
miR-103	1468	637	2.304
miR-124	2332	1163	2.005
miR-125a-5p	9079	2077	4.371
miR-125b-3p	29549	5541	5.333
miR-127	1209	356	3.400
miR-148b-3p	1924	528	3.641
miR-214	9826	3482	2.822
miR-291a-5p	5534	627	8.833
miR-296	33359	9235	3.612
miR-300-5p	65490	7287	8.987
miR-351	3707	1558	2.380
miR-363	1692	181	9.335
miR-382	18070	3630	4.978
miR-423	4548	1448	3.141
miR-494	31123	14605	2.131
miR-500	1225	548	2.235
miR-542-3p	14225	2133	6.668
miR-874	3096	1075	2.880

**name**	**ASML-TEX**	**ASML-Tspan8kd-TEX**	**ASML-Tspan8kd-TEX : ASML-TEX**
miR-7a	569	5188	9.122
miR-26b	7022	26350	3.752
miR-30b-3p	2348	5402	2.300
miR-34a	650	5420	8.339
miR-99b	1359	2793	2.054
miR-142-5p	541	1490	2.757
miR-185	655	1510	2.305
miR-325-5p	523	1617	3.095
miR-330	285	1181	4.147
miR-331	585	4471	7.650
miR-409-5p	375	1801	4.805
miR-471	432	1101	2.547

Table S4E

Top miRNA in AS-Tspan8-cells versus EC

name	AS-Tspan8-cells[a]	Name	EC[a]
let-7a	21915	let-7a	23209
let-7b	15247	let-7b	18017
let-7c	22129	let-7c	22736
let-7d	5248	let-7d	7196
let-7e	3349	let-7e	2981
let-7f	16370	let-7f	18605
let-7i	5160	let-7i	9778
miR-10b	2105	miR-10a-5p	2192
miR-15b	6033	miR-15b	4600
miR-16	13231	miR-16	14665
miR-21	69126	miR-19b	2054
miR-22	13479	miR-21	97312
miR-23a	6814	miR-22	10964
miR-23b	2605	miR-23a	23505
miR-24	3756	miR-23b	18313
miR-25	2107	miR-24	22666
miR-26a	6199	miR-26a	10907
miR-26b	3714	miR-26b	3772
miR-27a	4491	miR-27a	11889
miR-27b	1961	miR-27b	11648
miR-29a	19376	miR-29a	32837
miR-29b	10663	miR-29b	17774
miR-29c	9494	miR-29c	2631
miR-30a	1766	miR-30b-5p	1342
miR-30b-5p	1452	miR-30c	1602
miR-30c	3170	miR-31	7286
miR-30e	1527	miR-34a	3843
miR-34a	6911	miR-34b	1579
miR-34b	3144	miR-34c	1667
miR-34c	3666	miR-100	1587
miR-93	1355	miR-101a	1310
miR-96	4485	miR-103	1728
miR-99a	3704	miR-106b	1940
miR-103	2196	miR-107	1830
miR-106b	1533	miR-125b-5p	63999
miR-107	2899	miR-129	2316
miR-125a-5p	2292	miR-130a	8209
miR-125b-5p	14845	miR-140	4503
miR-130a	8911	miR-143	4197
miR-142-3p	2960	miR-146a	2959
miR-146a	2072	miR-199a-3p	20198
miR-182	1261	miR-199a-5p	6623
miR-183	1227	miR-204	1540
miR-195	4560	miR-210	1471
miR-210	2384	miR-214	4859
miR-221	2125	miR-221	7013
miR-450a	1357	miR-365	1686
miR-494	1231	miR-494	1732
miR-497	4695	miR-1224	1383
miR-1224	1628	miR-3584-5p	2519

[a] red: only in the top 50 AS-Tspan8-cells or EC

Table S5

The impact of Tspan8 on TEX uptake and potential targets

**TEX**	**Target**	**TEX uptake**	**TEX blockade**	**Target blockade**
AS-TEX	EC	+	CD9	CD151
			CD49c	CD54
				CD106
	Fb	+	CD9	CD9
			CD49c	CD151
				CD106
AS-Tspan8-TEX	EC	**+++**	Tspan8	CD151
			CD49d	CD54
			CD49e	CD106
	Fb	**++**	Tspan8	CD151
			CD49c	CD106

Table S6

TEX coculture-induced distinct mRNA recovery in fibroblasts and endothelial cells

Table S6A

AS-Tspan8-TEX coculture-induced Fb mRNA upregulation

**Symbol[a]**	**Fb**	**Fb+AS-Tsp8-TEX**	**Fb+AS-Tsp8-TEX : Fb**	**GeneName**
Aars	7383	39787	5.389	alanyl-tRNA synthetase
Aatf	1641	4421	2.694	apoptosis antagonizing transcription factor
Acsl1	1607	4096	2.549	acyl-CoA synthetase long-chain family member 1
Adora2b	413	1305	3.160	adenosine A2B receptor
Adrbk1	898	2419	2.694	adrenergic, beta, receptor kinase 1
Aen	6295	13777	2.189	apoptosis enhancing nuclease
Agfg2	2062	8306	4.028	ArfGAP with FG repeats 2
Ahi1	792	1722	2.173	Abelson helper integration site 1
Aig1	3641	9090	2.497	androgen-induced 1
Akap2	338	1017	3.010	A kinase (PRKA) anchor protein 2
Akna	1951	8192	4.199	AT-hook transcription factor
Alkbh1	592	1168	1.972	alkB, alkylation repair homolog
Alkbh3	1097	2257	2.056	alkB, alkylation repair homolog 3
Angel1	413	849	2.056	angel homolog 1
Anks3	4182	8719	2.085	ankyrin repeat and sterile alpha motif domain containing 3
Anks6	929	3281	3.531	ankyrin repeat and sterile alpha motif domain containing 6
Antxr2	4451	9742	2.189	similar to Antxr2 protein
Anxa3	239	1121	4.691	annexin A3
Ap2a2	4545	9541	2.099	adaptor-related protein complex 2, alpha 2 subunit
Apex1	4771	10297	2.158	APEX nuclease (multifunctional DNA repair enzyme) 1
Arhgef2	1698	3641	2.144	rho/rac guanine nucleotide exchange factor (GEF) 2
Arl15	367	760	2.071	ADP-ribosylation factor-like 15
Arl8b	9410	22227	2.362	ADP-ribosylation factor-like 8B
Arpc5l	12161	35610	2.928	actin related protein 2/3 complex, subunit 5-like
Artn	1618	9608	5.938	artemin
Asl	2937	6889	2.346	argininosuccinate lyase
Atf3	161	1872	11.632	activating transcription factor 3
Atf4	20882	73732	3.531	activating transcription factor 4 (tax-responsive enhancer B67)
Atf6	1046	5518	5.278	activating transcription factor 6
Atg16l2	820	1833	2.235	ATG16 autophagy related 16-like 2
Atp6v0b	3169	10297	3.249	ATPase, H+ transporting, lysosomal V0 subunit B
Atp6v1a	2647	5480	2.071	ATPase, H+ transporting, lysosomal V1 subunit A
Atp6v1c1	2288	4939	2.158	ATPase, H+ transporting, lysosomal V1 subunit C1
Atp6v1d	1885	3984	2.114	ATPase, H+ transporting, lysosomal V1 subunit D
Azin1	471	1075	2.282	antizyme inhibitor 1
Bbc3	3929	11666	2.969	Bcl-2 binding component 3
Bcar1	14263	27939	1.959	breast cancer anti-estrogen resistance 1
Bcat1	4871	16845	3.458	branched chain amino acid transaminase 1, cytosolic
Bcl2l1	10514	25355	2.412	Bcl2-like 1
Bf	635	2165	3.411	complement factor B
Bhlhe40	307	2006	6.543	basic helix-loop-helix family, member e40
Bnip3	7804	15826	2.028	BCL2/adenovirus E1B interacting protein 3
Bod1	724	2180	3.010	biorientation of chromosomes in cell division 1
Bola3	1652	3421	2.071	bolA homolog 3 (E. coli)
Bop1	471	1031	2.189	block of proliferation 1
Btg1	9608	26616	2.770	B-cell translocation gene 1, anti-proliferative
Btg3	340	962	2.828	BTG family, member 3
C4b	2006	6562	3.272	complement component 4B (Chido blood group)
Cars	760	3616	4.757	cysteinyl-tRNA synthetase
Casp12	962	2504	2.603	caspase 12
Casp4	1978	14462	7.311	caspase 4, apoptosis-related cysteine peptidase
Casp7	1820	3929	2.158	caspase 7
Catsper2	260	537	2.071	cation channel, sperm associated 2
Cby3	605	1380	2.282	chibby homolog 3 (Drosophila)
Ccdc101	3169	6985	2.204	coiled-coil domain containing 101
Ccnc	461	1082	2.346	cyclin C

Table S6A continued

Symbol	Fb	Fb+AS-Tsp8-TEX	Fb+AS-Tsp8-TEX : Fb	GeneName
Ccrn4l	158	576	3.655	CCR4 carbon catabolite repression 4-like
Ccser2	962	1924	2.000	granule cell antiserum positive 14
Cct8	588	1235	2.099	chaperonin containing Tcp1, subunit 8 (theta)
Cd68	274	622	2.266	Cd68 molecule
Cda	186	530	2.848	cytidine deaminase
Cdc42se2	263	657	2.497	CDC42 small effector 2
Cdpf1	1287	4096	3.182	similar to 2210021J22Rik protein
Cebpb	12161	24834	2.042	CCAAT/enhancer binding protein (C/EBP), beta
Cebpg	1409	2856	2.028	CCAAT/enhancer binding protein (C/EBP), gamma
Cers1	1458	6165	4.228	ceramide synthase 1
Chchd6	1510	5149	3.411	coiled-coil-helix-coiled-coil-helix domain containing 6
Chka	838	2759	3.294	choline kinase alpha
Ciart	117	1305	11.158	circadian associated repressor of transcription
Cisd1	12503	26616	2.129	CDGSH iron sulfur domain 1
Ciz1	1201	2539	2.114	CDKN1A interacting zinc finger protein 1
Clpp	592	1399	2.362	ClpP caseinolytic peptidase, ATP-dep., proteolytic subunit homol.
Cnfn	917	1938	2.114	cornifelin
Cnn3	11347	36107	3.182	calponin 3, acidic
Comtd1	8719	21619	2.479	catechol-O-methyltransferase domain containing 1
Cops7a	3468	7538	2.173	COP9 constitutive photomorphogenic homolog subunit 7A
Coq8b	1448	3169	2.189	aarF domain containing kinase 4
Cox6a2	96	1924	20.112	cytochrome c oxidase subunit VIa polypeptide 2
Cpeb1	1176	2504	2.129	cytoplasmic polyadenylation element binding protein 1
Cpeb4	315	942	2.990	cytoplasmic polyadenylation element binding protein 4
Creb3l1	1652	7383	4.469	cAMP responsive element binding protein 3-like 1
Crim1	19216	38699	2.014	cysteine rich transmembrane BMP regulator 1 (chordin like)
Cry1	263	1323	5.028	cryptochrome 1 (photolyase-like)
Csda	7538	15393	2.042	cold shock domain protein A
Ctps1	9027	22227	2.462	CTP synthase
Cuta	458	1067	2.329	cutA divalent cation tolerance homolog
Cyp3a9	572	1629	2.848	cytochrome P450, family 3, subfamily a, polypeptide 9
Dbp	9947	28329	2.848	D site of albumin promoter (albumin D-box) binding protein
Dbt	471	1342	2.848	dihydrolipoamide branched chain transacylase E2
Ddah1	7538	28725	3.811	dimethylarginine dimethylaminohydrolase 1
Ddit3	6794	56658	8.340	DNA-damage inducible transcript 3
Ddx59	541	1305	2.412	DEAD (Asp-Glu-Ala-Asp) box polypeptide 59
Dgka	519	1031	1.986	diacylglycerol kinase, alpha
Dhx58	232	588	2.532	DEXH (Asp-Glu-X-His) box polypeptide 58
Dmtf1	3566	7082	1.986	cyclin D binding myb-like transcription factor 1
Dnaja3	256	553	2.158	DnaJ (Hsp40) homolog, subfamily A, member 3
Dnajc2	10156	24154	2.378	DnaJ (Hsp40) homolog, subfamily C, member 2
Dnajc5	468	1218	2.603	DnaJ (Hsp40) homolog, subfamily C, member 5
Dnajc6	452	904	2.000	DnaJ (Hsp40) homolog, subfamily C, member 6
Dohh	10587	22073	2.085	deoxyhypusine hydroxylase/monooxygenase
Dok5	653	1710	2.621	docking protein 5
Dph5	2272	7231	3.182	DPH5 homolog
Dqx1	198	560	2.828	DEAQ box RNA-dependent ATPase 1
Dus2l	2504	6841	2.732	dihydrouridine synthase 2-like, SMM1 homolog
Dusp5	989	2165	2.189	dual specificity phosphatase 5
Dusp8	249	666	2.676	dual specificity phosphatase 8
Eef2kmt	271	534	1.972	family with sequence similarity 86, member A
Eefsec	792	1833	2.313	eukaryotic elongation factor, selenocysteine-tRNA-specific
Eepd1	512	1269	2.479	endonuclease/exonuclease/phosphatase family domain contain.1
Efna1	3191	12590	3.945	ephrin A1
Eif1	10587	27746	2.621	eukaryotic translation initiation factor 1
Eif1b	1235	4837	3.918	eukaryotic translation initiation factor 1B
Eif2ak2	391	982	2.514	eukaryotic translation initiation factor 2-alpha kinase 2
Eif2b3	699	4771	6.821	eukaryotic translation initiation factor 2B, subunit 3
Eif2s2	1160	2916	2.514	eukaryotic translation initiation factor 2, subunit 2 beta

Table S6A continued

Symbol	Fb	Fb+AS-Tsp8-TEX	Fb+AS-Tsp8-TEX : Fb	GeneName
Eif4ebp1	25532	69273	2.713	eukaryotic translation initiation factor 4E binding protein 1
Elmsan1	292	1428	4.891	similar to transcriptional regulating protein 132
Emp2	286	1075	3.758	epithelial membrane protein 2
Enc1	572	1563	2.732	ectodermal-neural cortex 1
Eno2	3616	9345	2.585	enolase 2, gamma, neuronal
Eprs	4513	14165	3.138	glutamyl-prolyl-tRNA synthetase
Eps8	843	3126	3.706	epidermal growth factor receptor pathway substrate 8
Esrra	7697	16845	2.189	estrogen related receptor, alpha
Ets2	1687	6339	3.758	v-ets erythroblastosis virus E26 oncogene homolog 2
Etv3	1607	3214	2.000	ets variant 3
Exoc3l4	269	1121	4.170	exocyst complex component 3-like 4
Ezr	3743	9947	2.657	ezrin
Faap20	437	917	2.099	FA core complex associated protein 20
Faf1	3083	7858	2.549	Fas (TNFRSF6) associated factor 1
Fam110a	523	1176	2.250	family with sequence similarity 110, member A
FAM120C	904	1833	2.028	family with sequence similarity 120C
Fam129a	969	2916	3.010	family with sequence similarity 129, member A
Fam195b	8135	22694	2.789	family with sequence similarity 195, member B
Fam50a	2241	4804	2.144	family with sequence similarity 50, member A
Fam53a	360	709	1.972	family with sequence similarity 53, member A
Fam65b	11347	32542	2.868	family with sequence similarity 65, member B
Fam71f1	94	699	7.413	family with sequence similarity 71, member F1
Farsb	2957	8780	2.969	phenylalanyl-tRNA synthetase, beta subunit
Fbxl4	1361	2937	2.158	F-box and leucine-rich repeat protein 4
Fbxo4	832	1911	2.297	F-box protein 4
Fkbp5	1468	3566	2.428	FK506 binding protein 5
Fosl1	1235	3373	2.732	fos-like antigen 1
Foxk1	553	2195	3.972	forkhead box K1
Foxs1	2452	4939	2.014	forkhead box S1
Gadd45a	2469	19083	7.727	growth arrest and DNA-damage-inducible, alpha
Gadd45b	4905	20738	4.228	growth arrest and DNA-damage-inducible, beta
Gadd45g	505	8249	16.336	growth arrest and DNA-damage-inducible, gamma
Gart	304	605	1.986	phosphoribosylglycinamide formyltransferase
Gas5	68794	161369	2.346	growth arrest specific 5
Gbp5	815	1734	2.129	guanylate binding protein 5
Gcat	760	3191	4.199	glycine C-acetyltransferase
Gcsh	760	2106	2.770	glycine cleavage system protein H (aminomethyl carrier)
Gdf15	236	5078	21.556	growth differentiation factor 15
Gga1	3397	6794	2.000	golgi associated, gamma adaptin ear cont., ARF binding protein 1
Ghitm	2978	11268	3.784	growth hormone inducible transmembrane protein
Gmeb1	260	516	1.986	glucocorticoid modulatory element binding protein 1
Gmfb	313	657	2.099	glia maturation factor, beta
Gng5	3350	8841	2.639	guanine nucleotide binding protein (G protein), gamma 5
Gnl3	5914	11994	2.028	guanine nucleotide binding protein-like 3 (nucleolar)
Gns	309	709	2.297	glucosamine (N-acetyl)-6-sulfatase
Got1	1629	3875	2.378	glutamic-oxaloacetic transaminase 1, (aspartate aminotransferase 1)
Gpatch11	898	2020	2.250	coiled-coil domain containing 75
Gpcpd1	251	1269	5.063	glycerophosphocholine phosphodiesterase GDE1 homolog
Gpr162	194	639	3.294	G protein-coupled receptor 162
Gpx1	8249	18306	2.219	glutathione peroxidase 1
Gramd1b	523	1342	2.567	GRAM domain containing 1B
Gtf2e1	564	1261	2.235	general transcription factor IIE, polypeptide 1 (alpha subunit)
Gtpbp2	481	1113	2.313	GTP binding protein 2
Gzmc	231	695	3.010	granzyme C
H1f0	32996	102127	3.095	H1 histone family, member 0
H2afj	20311	51063	2.514	H2A histone family, member J
H3 histone	7181	21619	3.010	histone cluster 1, H2ai-like
H3 histone	1218	3327	2.732	histone cluster 2, H3c2
H3f3b	23332	55492	2.378	H3 histone, family 3B

Table S6A continued

Symbol	Fb	Fb+AS-Tsp8-TEX	Fb+AS-Tsp8-TEX : Fb	GeneName
Hadhb	942	2574	2.732	hydroxyacyl-CoA dehydrog./3-ketoacyl-CoA thiolase/enoyl-CoA hydrat. β
Hars2	1031	2106	2.042	histidyl-tRNA synthetase 2-like
Haus8	803	1574	1.959	HAUS augmin-like complex, subunit 8
Hax1	815	1663	2.042	HCLS1 associated protein X-1
Hbegf	468	1361	2.908	heparin-binding EGF-like growth factor
Hbs1l	530	1243	2.346	Hbs1-like (S. cerevisiae)
Hebp1	2180	4451	2.042	heme binding protein 1
Herpud1	7383	18951	2.567	homocysteine- / ER stress-inducible, ubiquitin-like domain member 1
Hexb	15393	34397	2.235	hexosaminidase B
Hif1a	388	867	2.235	hypoxia-inducible factor 1, alpha subunit (transcription factor)
Hip1r	6123	12246	2.000	huntingtin interacting protein 1 related
Hist1h1c	13216	71220	5.389	similar to Histone H1.2 (H1 VAR.1) (H1c)
Hist1h1d	2435	9216	3.784	histone cluster 1, H1d
Hist1h2af	16271	47644	2.928	histone cluster 1, H2af
Hist1h2ak	21619	60725	2.809	histone cluster 1, H2ak
Hist1h2bd	1278	4451	3.482	histone cluster 1, H2bm
Hist1h2bh	1585	4608	2.908	histone cluster 1, H2bh
Hist1h2bk	3822	12766	3.340	histone cluster 1, H2bc
Hist1h2bl	2856	10016	3.506	histone cluster 1, H2bl
Hist2h2ac	7332	27746	3.784	histone cluster 2, H2ac
Hist2h2be	377	826	2.189	histone cluster 2, H2be
Hist3h3	21028	49667	2.362	similar to histone 1, H2ai
Hist4h4	3350	7332	2.189	histone cluster 2, H4
Hmga1	402	929	2.313	high mobility group AT-hook 1
Hps4	362	923	2.549	Hermansky-Pudlak syndrome 4 homolog
Hrasls	904	2876	3.182	HRAS-like suppressor
Hsd17b8	1965	6252	3.182	hydroxysteroid (17-beta) dehydrogenase 8
Hspa1b	1629	6123	3.758	heat shock 70kD protein 1B
Hspa5	22227	50711	2.282	heat shock protein 5
Hspa9	1184	7383	6.233	heat shock protein 9
Htatip2	2077	5833	2.809	HIV-1 tat interactive protein 2, homolog
Iars	2665	7181	2.694	isoleucyl-tRNA synthetase
Id1	267	662	2.479	inhibitor of DNA binding 1
Ier5	4451	22073	4.959	immediate early response 5
Ier5l	8540	19349	2.266	immediate early response 5-like
Ifi27l2b	6654	88906	13.361	interferon, alpha-inducible protein 27 like 2B
Ifit2	910	2504	2.751	interferon-induced protein with tetratricopeptide repeats 2
Ifit3	425	1060	2.497	interferon-induced protein with tetratricopeptide repeats 3
Ifngr2	3259	9810	3.010	interferon gamma receptor 2
Ifrd1	4545	13682	3.010	interferon-related developmental regulator 1
Igf2bp2	7486	15076	2.014	insulin-like growth factor 2 mRNA binding protein 2
Il23a	194	648	3.340	Interleukin 23, alpha subunit p19
Ilf3	942	1846	1.959	interleukin enhancer binding factor 3
Inip	904	1938	2.144	similar to HSPC043 protein
Insig1	3350	12161	3.630	insulin induced gene 1
Ip6k1	333	820	2.462	inositol hexakisphosphate kinase 1
Ipo5	5405	11666	2.158	importin 5
Irak3	3743	10735	2.868	interleukin-1 receptor-associated kinase 3
Irf7	2150	6383	2.969	interferon regulatory factor 7
Irx2	296	855	2.888	iroquois homeobox 2
Isg15	2665	5367	2.014	ISG15 ubiquitin-like modifier
Isg20	1510	3566	2.362	interferon stimulated exonuclease gene 20
Isx	4482	12678	2.828	similar to Retinal homeobox protein Rx (DRx1) (DRx)
Itpkc	311	879	2.828	inositol-trisphosphate 3-kinase C
Jade1	1342	3373	2.514	PHD finger protein 17
Junb	20032	56267	2.809	jun B proto-oncogene
Kcmf1	2150	4905	2.282	potassium channel modulatory factor 1
Kcne2	437	1152	2.639	potassium voltage-gated channel, Isk-related family, member 2
Kctd13	317	781	2.462	potassium channel tetramerisation domain containing 13

Table S6A continued

Symbol	Fb	Fb+AS-Tsp8-TEX	Fb+AS-Tsp8-TEX : Fb	GeneName
Kdm6b	560	1563	2.789	lysine (K)-specific demethylase 6B
Kifc3	8192	20738	2.532	kinesin family member C3
Klc1	2304	4973	2.158	kinesin light chain 1
Klf10	1252	4545	3.630	Kruppel-like factor 10
Klf2	182	755	4.141	Kruppel-like factor 2 (lung)
Klf9	3104	8249	2.657	Kruppel-like factor 9
Klhl21	996	3541	3.555	kelch-like 21 (Drosophila)
Kpna1	484	1235	2.549	karyopherin alpha 1
Lars	832	2320	2.789	leucyl-tRNA synthetase
Lcn2	2937	5793	1.972	lipocalin 2
Ldlr	226	1038	4.595	low density lipoprotein receptor
Letm1	1226	2778	2.266	leucine zipper-EF-hand containing transmembrane protein 1
Lhfpl2	564	1448	2.567	lipoma HMGIC fusion partner-like 2
Lims2	333	724	2.173	LIM and senescent cell antigen like domains 2
Lmbrd1	635	1428	2.250	LMBR1 domain containing 1
LOC102552870	13401	26432	1.972	similar to 60S ribosomal protein L23a
LOC103690178	815	1652	2.028	similar to Nucleolar GTP-bind.protein 1 (Chronic renal failure gene)
LOC103691261	272	734	2.694	similar to Sperm flagellar protein 1
LOC301725	7033	14766	2.099	similar to 60S ribosomal protein L35
LOC366632	5113	11113	2.173	similar to 40S ribosomal protein S6
LOC498154	2304	5997	2.603	hypothetical protein LOC498154
LOC498675	446	949	2.129	hypothetical LOC498675
LOC500028	1038	2385	2.297	hypothetical protein LOC500028
LOC678708	347	719	2.071	similar to histone 1, H2ai
LOC680322	27939	67378	2.412	similar to Histone H2A type 1
LOC680635	2778	7332	2.639	similar to 40S ribosomal protein S10
LOC689065	131	942	7.210	hypothetical protein LOC689065
LOC690386	832	2062	2.479	hypothetical protein LOC690386
Lonp1	5634	26432	4.691	lon peptidase 1, mitochondrial
Lrrc8b	324	781	2.412	leucine rich repeat containing 8 family, member B
Luc7l	1342	2837	2.114	LUC7-like
Lvrn	3169	6517	2.056	laeverin
Lyrm9	580	1448	2.497	RGD1562012
Lysmd2	1333	2740	2.056	LysM, putative peptidoglycan-binding, domain containing 2
Mafk	278	576	2.071	v-maf musculoaponeurotic fibrosarcoma oncogene homolog K
Map2k1	2435	5149	2.114	mitogen activated protein kinase kinase 1
Map6	744	2288	3.074	microtubule-associated protein 6
Mapkapk3	4837	11037	2.282	mitogen-activated protein kinase-activated protein kinase 3
Mars	3373	14362	4.257	methionine-tRNA synthetase
Mat2b	5293	10735	2.028	methionine adenosyltransferase II, beta
Mccrip2	923	3769	4.084	family with sequence similarity 195, member A
Mcoln1	3061	6339	2.071	mucolipin 1
Mcub	843	1820	2.158	coiled-coil domain containing 109B
Mdm2	2504	6654	2.657	Mdm2 p53 binding protein homolog (mouse)
MDR1a	88	734	8.340	ATP-binding cassette, sub-family B (MDR/TAP), member 1A
Mecp2	329	855	2.603	methyl CpG binding protein 2
Mef2b	229	1370	5.979	myocyte enhancer factor 2B
Metrnl	10226	29126	2.848	meteorin, glial cell differentiation regulator-like
Mettl11a	410	815	1.986	methyltransferase like 11A
Mettl22	1965	5113	2.603	methyltransferase like 22
Mettl3	2684	5442	2.028	methyltransferase-like 3
MGC105649	537	1771	3.294	hypothetical LOC302884
Mgea5	534	1053	1.972	meningioma expressed antigen 5 (hyaluronidase)
Micu1	5753	14263	2.479	mitochondrial calcium uptake 1
Mid1ip1	5293	14067	2.657	MID1 interacting protein 1 (gastrulation specific G12 homolog)
Mmaa	530	1323	2.497	methylmalonic aciduria (cobalamin deficiency) cblA type
Mnt	6841	23332	3.411	max binding protein
Mok	340	787	2.313	MOK protein kinase
Mospd1	315	639	2.028	motile sperm domain containing 1

Table S6A continued

Symbol	Fb	Fb+AS-Tsp8-TEX	Fb+AS-Tsp8-TEX : Fb	GeneName
Mpp5	605	1351	2.235	membrane protein, palmitoylated 5 (MAGUK p55 subfamily member 5)
Mpv17l2	9608	24154	2.514	MPV17 mitochondrial membrane protein-like 2
Mri1	2385	5293	2.219	methylthioribose-1-phosphate isomerase homolog
Mrnip	609	1351	2.219	MRN complex interacting protein
Mrpl17	11426	22694	1.986	mitochondrial ribosomal protein L17
Mrpl38	488	1060	2.173	mitochondrial ribosomal protein L38
Mrpl55	4012	8481	2.114	mitochondrial ribosomal protein L55
Mrps18b	516	1226	2.378	mitochondrial ribosomal protein S18B
Mt1a	45703	116502	2.549	metallothionein 1a
Mthfd1	929	3083	3.317	methylenetetrahydrofolate dehydrogenase 1, -cyclohydrolase
Mthfd2	1380	13588	9.849	methylenetetrahydrofolate dehydrogenase 2, -cyclohydrolase
Mtpap	923	1911	2.071	mitochondrial poly(A) polymerase
Mus81	534	1389	2.603	MUS81 endonuclease homolog
Mxd1	362	776	2.144	max dimerization protein 1
Myo5a	1510	3083	2.042	myosin VA
N4bp2l1	269	826	3.074	NEDD4 binding protein 2-like 1
Naglu	3902	20882	5.352	N-acetyl-alpha-glucosaminidase
Nampt	452	1193	2.639	nicotinamide phosphoribosyltransferase
Nars	671	2336	3.482	asparaginyl-tRNA synthetase
Nckipsd	1097	3191	2.908	NCK interacting protein with SH3 domain
Ndufaf6	891	1746	1.959	NADH:ubiquinone oxidoreductase complex assembly factor 6
Nek6	1121	2385	2.129	NIMA (never in mitosis gene a)-related kinase 6
Neu1	617	1428	2.313	sialidase 1 (lysosomal sialidase)
Nfe2l1	1038	3984	3.837	nuclear factor, erythroid derived 2,-like 1
Nfe2l2	13034	27554	2.114	nuclear factor, erythroid derived 2, like 2
Nfu1	242	739	3.053	NFU1 iron-sulfur cluster scaffold homolog
Nfyb	1168	2504	2.144	nuclear transcription factor-Y beta
Ngdn	2978	7033	2.362	neuroguidin, EIF4E binding protein
Ninj1	1783	3492	1.959	ninjurin 1
Nkain1	820	1771	2.158	Na+/K+ transporting ATPase interacting 1
Nkx6-3	867	1783	2.056	NK6 homeobox 3
Nmnat3	1278	3061	2.395	nicotinamide nucleotide adenylyltransferase 3
Nol8	936	1846	1.972	nucleolar protein 8
Nop14	4068	8719	2.144	NOP14 nucleolar protein homolog
Nop58	3019	6747	2.235	nucleolar protein NOP58
Npap60	3848	12766	3.317	nuclear pore associated protein
Npas2	247	519	2.099	neuronal PAS domain protein 2
Npc1	3666	7858	2.144	Cdig2 protein
Npepl1	568	1176	2.071	aminopeptidase-like 1
Nphp1	2539	10735	4.228	nephronophthisis 1 (juvenile) homolog
Nr1d1	288	1924	6.681	nuclear receptor subfamily 1, group D, member 1
Nrf1	471	1017	2.158	nuclear respiratory factor 1
Nrg1	242	622	2.567	neuregulin 1
Nsd1	861	1771	2.056	nuclear receptor binding SET domain protein 1
Nsd2	434	861	1.986	Wolf-Hirschhorn syndrome candidate 1
Nudt6	256	695	2.713	nudix (nucleoside diphosphate linked moiety X)-type motif 6
Nudt9	1911	4451	2.329	nudix (nucleoside diphosphate linked moiety X)-type motif 9
Nup54	370	861	2.329	nucleoporin 54
Odc1	1783	6252	3.506	ornithine decarboxylase 1
Optn	4482	9541	2.129	optineurin
Oser1	3875	10809	2.789	oxidative stress responsive gene
Otub2	2034	4738	2.329	OTU domain, ubiquitin aldehyde binding 2
P2ry2	391	1160	2.969	purinergic receptor P2Y, G-protein coupled, 2
Pak1ip1	431	1552	3.605	PAK1 interacting protein 1
Paox	4939	10016	2.028	polyamine oxidase (exo-N4-amino)
Pappa1	143	501	3.506	pregnancy-associated plasma protein A
Parvb	261	644	2.462	parvin, beta
Pck2	4837	10661	2.204	phosphoenolpyruvate carboxykinase 2 (mitochondrial)
Pcp4l1	2135	4182	1.959	Purkinje cell protein 4-like 1

Table S6A continued

Symbol	Fb	Fb+AS-Tsp8-TEX	Fb+AS-Tsp8-TEX : Fb	GeneName
Pcsk4	3517	7033	2.000	proprotein convertase subtilisin/kexin type 4
Pdcd6	9476	19216	2.028	programmed cell death 6
Pdrg1	1687	3984	2.362	p53 and DNA damage regulated 1
Pfkp	4182	11037	2.639	phosphofructokinase, platelet
Phgdh	9476	38166	4.028	phosphoglycerate dehydrogenase
Pigp	8903	17805	2.000	phosphatidylinositol glycan anchor biosynthesis, class P
Pim1	125	803	6.409	pim-1 oncogene
Pim3	4608	13401	2.908	pim-3 oncogene
Pknox1	237	744	3.138	PBX/knotted 1 homeobox 1
Pkp1	5518	29328	5.315	plakophilin 1
Pkp2	855	2876	3.364	plakophilin 2
Plekha4	2504	8719	3.482	pleckstrin homology domain containing, family A member 4
Plekhg5	307	653	2.129	pleckstrin homology domain containing, family G member 5
Pnpla2	910	2487	2.732	patatin-like phospholipase domain containing 2
Polg2	461	910	1.972	polymerase (DNA directed), gamma 2, accessory subunit
Ppat	284	592	2.085	phosphoribosyl pyrophosphate amidotransferase
Ppp1r15a	2469	39512	16.000	protein phosphatase 1, regulatory subunit 15A
Ppp1r32	639	1323	2.071	protein phosphatase 1, regulatory subunit 32
Prag1	226	592	2.621	pragma of Rnd2
Prdx5	36866	87077	2.362	peroxiredoxin 5
Prepl	468	1370	2.928	prolyl endopeptidase-like
Prmt1	15393	32317	2.099	protein arginine methyltransferase 1
Prmt5	724	1448	2.000	protein arginine methyltransferase 5
Prmt7	744	1758	2.362	protein arginine methyltransferase 7
Proser2	296	1010	3.411	hypothetical protein LOC683460
Prpf40b	729	1783	2.445	pre-mRNA processing factor 40 homolog B
Prr7	2077	5257	2.532	proline rich 7 (synaptic)
Psat1	5914	12161	2.056	phosphoserine aminotransferase 1
Psph	530	1618	3.053	phosphoserine phosphatase
Ptcd2	508	1193	2.346	pentatricopeptide repeat domain 2
Ptges2	288	885	3.074	prostaglandin E synthase 2
Ptp4a1	6252	25006	4.000	protein tyrosine phosphatase type IVA, member 1
Ptpdc1	307	826	2.694	protein tyrosine phosphatase domain containing 1
Ptprm	278	826	2.969	protein tyrosine phosphatase, receptor type, M
Qsox2	413	1003	2.428	quiescin Q6 sulfhydryl oxidase 2
R3hdm1	2876	13777	4.790	R3H domain containing 1
Rab11fip5	8364	21921	2.621	similar to RAB11 family interacting protein 5 (class I) isoform 1
Rab32	576	1499	2.603	RAB32, member RAS oncogene family
Rab3il1	300	676	2.250	RAB3A interacting protein (rabin3)-like 1
Rabggtb	7968	18179	2.282	Rab geranylgeranyltransferase, beta subunit
Rad23a	657	1389	2.114	RAD23 homolog A
Rad9b	639	1618	2.532	RAD9 homolog B
Rae1	1563	3104	1.986	RAE1 RNA export 1 homolog
Rai14	5557	21921	3.945	retinoic acid induced 14
Rala	6383	15393	2.412	v-ral simian leukemia viral oncogene homolog A (ras related)
Rangrf	263	576	2.189	RAN guanine nucleotide release factor
Rars2	1898	4096	2.158	arginyl-tRNA synthetase 2, mitochondrial
Rasl12	399	2419	6.063	RAS-like, family 12
Rassf5	286	596	2.085	Ras association (RalGDS/AF-6) domain family member 5
Rbm28	832	1663	2.000	RNA binding motif protein 28
Rbm38	709	1938	2.732	RNA binding motif protein 38
Rcc2	898	2304	2.567	regulator of chromosome condensation 2
Rell2	873	6562	7.516	RELT-like 2
Renbp	898	2020	2.250	renin binding protein
RGD1304624	265	1024	3.864	similar to RIKEN cDNA 2700097O09
RGD1311739	3214	7281	2.266	similar to RIKEN cDNA 1700037H04
RGD1559892	4012	8023	2.000	ribosomal protein L29 pseudogene
RGD1560073	20311	40342	1.986	similar to ribosomal protein S10
RGD1561520	49667	104996	2.114	similar to Ab2-162

Table S6A continued

Symbol	Fb	Fb+AS-Tsp8-TEX	Fb+AS-Tsp8-TEX : Fb	GeneName
RGD1561871	23332	46663	2.000	similar to ribosomal protein S10
RGD1562114	7643	19083	2.497	RGD1562114
RGD1562378	534	1201	2.250	histone H4 variant H4-v.1
RGD1563378	11585	34636	2.990	similar to ferritin heavy polypeptide-like 17
RGD1564482	714	1499	2.099	RGD1564482
RGD1565033	666	2106	3.160	similar to hypothetical protein LOC284018 isoform b
RGD1565170	4545	9345	2.056	similar to 60S ribosomal protein L23a
RGD1566033	300	750	2.497	similar to BC003940 protein
Rgs3	8192	21028	2.567	regulator of G-protein signaling 3
Rhbdf2	425	873	2.056	rhomboid 5 homolog 2 (Drosophila)
Rhob	331	648	1.959	ras homolog gene family, member B
Riok3	3492	19484	5.579	RIO kinase 3 (yeast)
Rnf34	1758	3517	2.000	ring finger protein 34
RP4	1885	6608	3.506	RGD1559532
Rpe	657	1296	1.972	ribulose-5-phosphate-3-epimerase
Rpf2	452	885	1.959	ribosome production factor 2 homolog (S. cerevisiae)
Rpl10l	1097	2336	2.129	ribosomal protein L10-like
Rragd	365	2150	5.897	Ras-related GTP binding D
Rrp12	609	1520	2.497	similar to Protein KIAA0690
Rrp9	1418	3769	2.657	ribosomal RNA process.9, small subunit (SSU) processome comp.
Rsad1	407	1351	3.317	similar to radical S-adenosyl methionine domain containing 1
Rsad2	331	2435	7.362	radical S-adenosyl methionine domain containing 2
Runx1	5955	19484	3.272	runt-related transcription factor 1
Rwdd1	6252	14263	2.282	RWD domain containing 1
Sacm1l	680	1552	2.282	SAC1 suppressor of actin mutations 1-like
Samd4a	929	2721	2.928	sterile alpha motif domain containing 4A
Sars	1758	6081	3.458	seryl-tRNA synthetase
Sdad1	478	982	2.056	SDA1 domain containing 1
Sdcbp2	168	765	4.563	syndecan binding protein (syntenin) 2
Sdf2l1	2180	7281	3.340	stromal cell-derived factor 2-like 1
Sdr39u1	734	1618	2.204	short chain dehydrogenase/reductase family 39U, member 1
Selenow	1218	2487	2.042	selenoprotein W, 1
Sema3b	2106	4608	2.189	sema domain, Ig & short basic domain, secreted, (semaphorin) 3B
Serac1	416	1128	2.713	serine active site containing 1
Serpine1	4871	15393	3.160	serpin peptidase inhibitor, clade E, member 1
Sesn2	152	519	3.411	sestrin 2
Sfxn1	1010	3397	3.364	sideroflexin 1
Sh3bp2	4153	10156	2.445	SH3-domain binding protein 2
Shc2	580	1641	2.828	SHC (Src homology 2 domain containing) transforming protein 2
Shisa4	410	1144	2.789	shisa homolog 4
Shisa5	3444	6841	1.986	shisa homolog 5
Shmt1	734	2048	2.789	serine hydroxymethyltransferase 1 (soluble)
Shmt2	1333	5595	4.199	serine hydroxymethyltransferase 2 (mitochondrial)
Sik1	309	630	2.042	salt-inducible kinase 1
Sirt3	478	949	1.986	sirtuin 3
Skil	191	553	2.888	SKI-like oncogene
Slc16a1	329	1105	3.364	solute carrier family 16, member 1 (monocarbox.acid transporter 1)
Slc17a5	1783	3591	2.014	solute carrier family 17 (anion/sugar transporter), member 5
Slc1a5	1152	3692	3.204	solute carrier family 1 (neutral amino acid transporter), member 5
Slc20a1	3591	12161	3.387	solute carrier family 20 (phosphate transporter), member 1
Slc20a2	9027	19756	2.189	solute carrier family 20 (phosphate transporter), member 2
Slc25a12	3984	7968	2.000	solute carrier family 25 (mitochondrial carrier, Aralar), member 12
Slc25a25	1652	4738	2.868	solute carrier family 25 (mitochondrial carrier, phosph.), member 25
Slc25a30	1428	3104	2.173	solute carrier family 25, member 30
Slc25a33	260	1531	5.897	solute carrier family 25, member 33
Slc25a37	197	776	3.945	solute carrier family 25, member 37
Slc25a38	1075	2740	2.549	solute carrier family 25, member 38
Slc2a1	3717	11994	3.227	solute carrier family 2 (facilitated glucose transporter), member 1
Slc33a1	416	815	1.959	solute carrier family 33 (acetyl-CoA transporter), member 1

Table S6A continued

Symbol	Fb	Fb+AS-Tsp8-TEX	Fb+AS-Tsp8-TEX : Fb	GeneName
Slc3a2	3795	29532	7.781	solute carrier family 3 (activat.dibasic & neutral AA transp.), memb. 2
Slc6a9	331	1428	4.317	solute carrier family 6 (neurotransmitter transp., glycine), member 9
Slc7a1	556	1607	2.888	solute carrier family 7 (cationic amino acid transporter), member 1
Slc7a5	249	1698	6.821	solute carrier family 7 (amino acid transporter light chain), member 5
Slfn2	750	2210	2.949	schlafen 2
Smad1	1269	3083	2.428	SMAD family member 1
Smg6	437	1176	2.694	Smg-6 homolog, nonsense mediated mRNA decay factor
Smg9	553	1105	2.000	smg-9 homolog, nonsense mediated mRNA decay factor
Smim29	4837	10885	2.250	small integral membrane protein 29
Snx12	393	867	2.204	sorting nexin 12
Snx8	169	534	3.160	sorting nexin 8
Sp2	333	709	2.129	Sp2 transcription factor
Specc1l	553	1097	1.986	sperm antigen with calponin homol. and coiled-coil domains 1-like
Spef1	1046	2385	2.282	sperm flagellar 1
Spout1	690	1820	2.639	similar to LOC495800 protein
Sqstm1	13970	28924	2.071	sequestosome 1
Srebf1	4240	10661	2.514	sterol regulatory element binding transcription factor 1
Srfbp1	622	1380	2.219	serum response factor binding protein 1
Srgap2	4608	10809	2.346	SLIT-ROBO Rho GTPase activating protein 2
Srm	3169	8364	2.639	spermidine synthase
Srp68	5753	11268	1.959	signal recognition particle 68
Srrd	704	1389	1.972	SRR1 domain containing
Ssu72	6747	14462	2.144	SSU72 RNA polymerase II CTD phosphatase homolog
St7l	3517	7332	2.085	suppression of tumorigenicity 7-like
Stambpl1	2876	6123	2.129	similar to AMSH-family protein
Stat2	4012	9027	2.250	signal transducer and activator of transcription 2
Stbd1	338	1924	5.696	starch binding domain 1
Steap3	530	1531	2.888	STEAP family member 3, metalloreductase
Stk11ip	304	750	2.462	serine/threonine kinase 11 interacting protein
Strbp	1323	2665	2.014	spermatid perinuclear RNA binding protein
Stxbp5	333	704	2.114	syntaxin binding protein 5 (tomosyn)
Styxl1	626	1252	2.000	serine/threonine/tyrosine interacting-like 1
Surf1	3492	6937	1.986	surfeit 1
Surf2	4068	9281	2.282	surfeit 2
Taf1d	2385	9090	3.811	TATA box bind.protein (Tbp)-associated factor, RNA polymerase I, D
Tap1	657	1820	2.770	transporter 1, ATP-binding cassette, sub-family B (MDR/TAP)
Tap2	4068	8023	1.972	transporter 2, ATP-binding cassette, sub-family B (MDR/TAP)
Tapbp	1269	2610	2.056	TAP binding protein
Tardbp	3019	6165	2.042	TAR DNA binding protein
Tars	5914	18179	3.074	threonyl-tRNA synthetase
Tars2	4300	9742	2.266	threonyl-tRNA synthetase 2, mitochondrial (putative)
Tbc1d20	541	1520	2.809	TBC1 domain family, member 20
Tbccd1	380	744	1.959	TBCC domain containing 1
Tcea2	402	820	2.042	transcription elongation factor A (SII), 2
Tfap4	609	1965	3.227	transcription factor AP-4
Tfpt	383	755	1.972	TCF3 (E2A) fusion partner
Tgfbr2	685	1951	2.848	transforming growth factor, beta receptor II
Timm8a1	1287	2957	2.297	translocase of inner mitochondrial membrane 8 homolog a1
Tjap1	5634	11347	2.014	tight junction associated protein 1
Tjp3	108	501	4.659	tight junction protein 3
Tlcd1	1168	4482	3.837	TLC domain containing 1
Tma16	1053	3517	3.340	similar to RIKEN cDNA 1810029B16
Tmco4	1176	2937	2.497	transmembrane and coiled-coil domains 4
Tmem116	135	781	5.776	transmembrane protein 116
Tmem140	1846	6252	3.387	transmembrane protein 140
Tmem189	622	1479	2.378	transmembrane protein 189
Tmem192	1010	2048	2.028	transmembrane protein 192
Tmem199	261	530	2.028	transmembrane protein 199
Tmem250	474	1269	2.676	RGD1564379

Table S6A continued

Symbol	Fb	Fb+AS-Tsp8-TEX	Fb+AS-Tsp8-TEX : Fb	GeneName
Tmem256	14165	32542	2.297	transmembrane protein 256
Tmem268	2165	7033	3.249	similar to RIKEN cDNA 6330416G13 gene
Tmem55b	449	1128	2.514	transmembrane protein 55B
Tmem62	343	1361	3.972	transmembrane protein 62
Tnip2	1082	2504	2.313	TNFAIP3 interacting protein 2
Tollip	4451	8903	2.000	toll interacting protein
Tom1	14362	36866	2.567	target of myb1 homolog
Tp53inp1	370	1563	4.228	tumor protein p53 inducible nuclear protein 1
Traf2	917	2647	2.888	Tnf receptor-associated factor 2
Trib3	194	3397	17.509	tribbles homolog 3
Trim37	377	815	2.158	tripartite motif-containing 37
Trim8	1261	2521	2.000	tripartite motif-containing 8
Trit1	393	885	2.250	tRNA isopentenyltransferase 1
Trpt1	613	1361	2.219	tRNA phosphotransferase 1
Tsc22d3	2006	4300	2.144	TSC22 domain family, member 3
Tspan5	861	2226	2.585	tetraspanin 5
Tssc4	7591	15936	2.099	tumor suppressing subtransferable candidate 4
Ttc39c	7968	22851	2.868	hypothetical protein LOC686179
Tyw5	1010	1978	1.959	tRNA-yW synthesizing protein 5
Uap1l1	13401	41190	3.074	UDP-N-acteylglucosamine pyrophosphorylase 1-like 1
Ubald2	13034	32317	2.479	family with sequence similarity 100, member B
Ube4b	714	1438	2.014	ubiquitination factor E4B
Ubqln1	3666	7858	2.144	ubiquilin 1
Uchl5	917	1911	2.085	ubiquitin carboxyl-terminal hydrolase L5
Uck2	4576	17929	3.918	uridine-cytidine kinase 2
Unc5b	286	704	2.462	unc-5 homolog B
Upp1	3281	6747	2.056	uridine phosphorylase 1
Uprt	431	1458	3.387	uracil phosphoribosyltransferase (FUR1) homolog
Uqcc2	3795	7434	1.959	similar to RIKEN cDNA 2900010M23
Uri1	2998	6608	2.204	URI1, prefoldin-like chaperone
Usp14	2452	5557	2.266	ubiquitin specific peptidase 14
Usp18	242	1734	7.160	ubiquitin specific peptidase 18
Vapa	9476	20311	2.144	VAMP (vesicle-associated membrane protein)-associated protein A
Vegfa	2304	7858	3.411	vascular endothelial growth factor A
Vhl	4673	10226	2.189	von Hippel-Lindau tumor suppressor
Wars	5149	43841	8.515	tryptophanyl-tRNA synthetase
Wdcp	685	1698	2.479	WD repeat and coiled coil containing
Wdfy1	2288	6747	2.949	WD repeat and FYVE domain containing 1
Wdr12	347	709	2.042	WD repeat domain 12
Wdr25	867	2048	2.362	WD repeat domain 25-like
Wdr70	942	2048	2.173	WD repeat domain 70
Wdr77	1585	3126	1.972	WD repeat domain 77
Wdr89	272	556	2.042	WD repeat domain 89
Whamm	413	815	1.972	WAS protein homolog associated with actin, golgi and microtubules
Wwc2	1992	4211	2.114	WW and C2 domain containing 2
Xpo1	1252	3421	2.732	exportin 1, CRM1 homolog
Xpot	1510	3591	2.378	exportin, tRNA (nuclear export receptor for tRNAs)
Yae1d1	1235	2957	2.395	Yae1 domain containing 1
Yars	613	2336	3.811	tyrosyl-tRNA synthetase
Ypel5	4871	17929	3.681	yippee-like 5
Zbtb21	534	1333	2.497	zinc finger protein 295
Zbtb38	425	955	2.250	zinc finger and BTB domain containing 38
Zbtb7b	553	2521	4.563	zinc finger and BTB domain containing 7B
Zc3h8	1105	5595	5.063	zinc finger CCCH type containing 8
Zfand2a	2419	7281	3.010	zinc finger, AN1-type domain 2A
Zfp280c	936	1924	2.056	suppressor of hairy wing homolog 3
Zfp775	377	873	2.313	zinc finger protein 775
Zfr	7033	19756	2.809	zinc finger RNA binding protein
Zmynd15	428	949	2.219	zinc finger, MYND-type containing 15

Table S6B

AS-Tspan8-TEX coculture-induced Fb mRNA downregulation

Symbol[a]	Fb	Fb+AS-Tspan8-TEX	Fb+AS-Tsp8-TEX : Fb	GeneName
Aamdc	1370	657	0.480	adipogenesis associated, Mth938 domain containing
Abt1	622	286	0.460	activator of basal transcription 1
Acat2	9027	2369	0.262	acetyl-Coenzyme A acetyltransferase 3
Acpl2	508	246	0.483	acid phosphatase-like 2
Acss2	690	249	0.361	acyl-CoA synthetase short-chain family member 2
Acy3	4360	1898	0.435	aspartoacylase (aminocyclase) 3
Adam10	949	335	0.354	ADAM metallopeptidase domain 10
Adam15	1641	676	0.412	a disintegrin and metallopeptidase domain 15 (metargidin)
Adam9	588	282	0.480	ADAM metallopeptidase domain 9
Adamts15	1489	671	0.451	ADAM metallopeptidase with thrombospondin type 1 motif, 15
Adamts2	3875	982	0.253	ADAM metallopeptidase with thrombospondin type 1 motif, 2
Adarb1	1771	690	0.390	adenosine deaminase, RNA-specific, B1
Adcy9	474	195	0.412	adenylate cyclase 9
Akap17b	422	211	0.500	A kinase (PRKA) anchor protein 17B
Akr1c13	605	195	0.323	aldo-keto reductase family 1, member C13
Akr1c19	488	191	0.392	aldo-keto reductase family 1, member C19
Akr1e2	1641	505	0.308	aldo-keto reductase family 1, member E2
Aldh3a1	20882	1287	0.062	aldehyde dehydrogenase 3 family, member A1
Aldh5a1	1038	315	0.304	aldehyde dehydrogenase 5 family, member A1
Ankrd37	609	144	0.237	ankyrin repeat domain 37
Ankrd50	1209	605	0.500	ankyrin repeat domain 50
Anpep	478	174	0.363	alanyl (membrane) aminopeptidase
Anxa1	139509	61147	0.438	annexin A1
Anxa6	11037	5405	0.490	annexin A6
Aoc3	5634	596	0.106	amine oxidase, copper containing 3 (vascular adhesion protein 1)
Aox1	9742	1951	0.200	aldehyde oxidase 1
Ap2s1	22851	9090	0.398	adaptor-related protein complex 2, sigma 1 subunit
Aqp1	461	151	0.328	aquaporin 1
Arg1	6889	1859	0.270	arginase, liver
Arglu1	11113	5257	0.473	arginine and glutamate rich 1
Arhgap11a	505	212	0.420	Rho GTPase activating protein 11A
Arhgap18	501	195	0.390	Rho GTPase activating protein 18
Arhgap28	2006	630	0.314	Rho GTPase activating protein 28
Arl2bp	3566	1687	0.473	ADP-ribosylation factor-like 2 binding protein
Arnt2	1314	541	0.412	aryl hydrocarbon receptor nuclear translocator 2
Arpc1a	4182	2077	0.497	actin related protein 2/3 complex, subunit 1A
Arpc5	26616	11037	0.415	actin related protein 2/3 complex, subunit 5
Arsi	1992	553	0.277	arylsulfatase family, member I
Asf1b	630	274	0.435	ASF1 anti-silencing function 1 homolog B
Aspm	2916	1428	0.490	asp (abnormal spindle) homolog, microcephaly associated
Aspn	5221	272	0.052	asporin
Atp2c1	1783	826	0.463	ATPase, Ca++ transporting, type 2C, member 1
Atp5j2	55492	23657	0.426	ATP synthase, H+ transporting, mitochondrial Fo complex, subunit F2
Aurka	1652	662	0.401	aurora kinase A
Bhmt	596	241	0.403	betaine-homocysteine S-methyltransferase
Birc5	2369	826	0.349	baculoviral IAP repeat-containing 5
Bloc1s1	11666	4240	0.363	biogenesis of lysosomal organelles complex-1, subunit 1
Bmp4	12417	2721	0.219	bone morphogenetic protein 4
Bnip3l	22073	10016	0.454	BCL2/adenovirus E1B interacting protein 3-like
Borcs7	1269	576	0.454	BLOC-1 related complex subunit 7
Borcs8	1820	596	0.328	BLOC-1 related complex subunit 8
Brip1	617	234	0.379	BRCA1 interacting protein C-terminal helicase 1
Bst1	6889	3236	0.470	bone marrow stromal cell antigen 1
Btd	8719	1965	0.225	biotinidase
C1qtnf6	4182	1924	0.460	C1q and tumor necrosis factor related protein 6
C1r	65083	28526	0.438	complement component 1, r subcomponent
Calhm2	1652	820	0.497	calcium homeostasis modulator 2
Calml4	734	142	0.193	calmodulin-like 4

Table S6B continued

Symbol	Fb	Fb+AS-Tspan8-TEX	Fb+AS-Tsp8-TEX : Fb	GeneName
Casq1	1168	362	0.310	calsequestrin 1 (fast-twitch, skeletal muscle)
Cav1	63744	18433	0.289	caveolin 1, caveolae protein
Ccdc167	501	242	0.483	coiled-coil domain containing 167
Ccdc28b	1663	622	0.374	coiled coil domain containing 28B
Ccdc99	695	324	0.467	coiled-coil domain containing 99
Ccl19	1771	820	0.463	chemokine (C-C motif) ligand 19
Ccl2	36358	11268	0.310	chemokine (C-C motif) ligand 2
Ccna2	2610	826	0.316	cyclin A2
Ccnb1	1342	455	0.339	cyclin B1
Ccnb2	1269	402	0.316	cyclin B2
Ccne1	609	208	0.342	cyclin E1
Ccnyl1	1184	422	0.356	cyclin Y-like 1
Cd1d1	617	307	0.497	CD1d1 molecule
Cd302	6339	2539	0.401	CD302 molecule
Cd36	1209	239	0.198	CD36 molecule (thrombospondin receptor)
Cd3eap	2226	1010	0.454	CD3e molecule, epsilon associated protein
Cd48	622	309	0.497	Cd48 molecule
Cdc20	2435	1144	0.470	cell division cycle 20 homolog
Cdc42bpg	1510	704	0.467	CDC42 binding protein kinase gamma (DMPK-like)
Cdc42ep5	5043	2504	0.497	CDC42 effector protein (Rho GTPase binding) 5
Cdc45	1641	523	0.319	cell division cycle 45 homolog
Cdca3	5078	1296	0.255	cell division cycle associated 3
Cdk2	724	331	0.457	cyclin dependent kinase 2
Cdkn2c	1771	820	0.463	cyclin-dependent kinase inhibitor 2C (p18, inhibits CDK4)
Cebpd	42348	12077	0.285	CCAAT/enhancer binding protein (C/EBP), delta
Cenpf	739	229	0.310	centromere protein F
Cenpj	1499	714	0.476	centromere protein J
Cenpq	592	254	0.429	centromere protein Q
Cenpw	1734	609	0.351	centromere protein W
Cep55	2369	584	0.247	centrosomal protein 55
Ces1a	7804	1226	0.157	carboxylesterase 1A
Ces1d	3875	146	0.038	carboxylesterase 1D
Cfap100	15608	7538	0.483	cilia and flagella associated protein 100
Cfh	1641	724	0.441	complement factor H
Cisd2	1510	744	0.493	CDGSH iron sulfur domain 2
Ckap4	2557	1218	0.476	cytoskeleton-associated protein 4
Cklf	1795	461	0.257	chemokine-like factor
Clec11a	617	147	0.238	C-type lectin domain family 11, member A
Clmp	680	311	0.457	CXADR-like membrane protein
Cog8	1574	760	0.483	component of oligomeric golgi complex 8
Col12a1	3083	1468	0.476	collagen, type XII, alpha 1
Col14a1	484	108	0.224	collagen, type XIV, alpha 1
Col1a1	26068	5442	0.209	collagen, type I, alpha 1
Col1a2	172951	72214	0.418	collagen, type I, alpha 2
Col3a1	57849	4738	0.082	collagen, type III, alpha 1
Col5a1	6794	2778	0.409	collagen, type V, alpha 1
Col6a1	6654	3040	0.457	collagen, type VI, alpha 1
Col6a2	22381	9608	0.429	collagen, type VI, alpha 2
Commd1	12766	6165	0.483	copper metabolism (Murr1) domain containing 1
Cotl1	3902	1176	0.301	coactosin-like 1
Cox6b1	14563	6295	0.432	cytochrome c oxidase subunit 6B1
Cox7a2	22073	10441	0.473	cytochrome c oxidase subunit VIIa polypeptide 2
Cox7b	15181	6841	0.451	cytochrome c oxidase subunit VIIb
Cr1l	21321	9947	0.467	complement component (3b/4b) receptor 1-like
Crip2	55878	11585	0.207	cysteine-rich protein 2
Crtap	1168	556	0.476	cartilage associated protein
Cryl1	1046	388	0.371	crystallin, lambda 1
Csgalnact1	709	237	0.334	chondroitin sulfate N-acetylgalactosaminyltransferase 1
Csrp2	685	274	0.401	cysteine and glycine-rich protein 2

Table S6B continued

Symbol	Fb	Fb+AS-Tspan8-TEX	Fb+AS-Tsp8-TEX : Fb	GeneName
Ctf1	592	278	0.470	cardiotrophin 1
Ctsh	2684	1342	0.500	cathepsin H
Ctu1	1734	803	0.463	cytosolic thiouridylase subunit 1 homolog
Ctxn1	508	211	0.415	cortexin 1
Cx3cl1	128375	25355	0.198	chemokine (C-X3-C motif) ligand 1
Cxcl1	44453	11505	0.259	chemokine (C-X-C motif) ligand 1 (melanoma growth stim. activity, α)
Cxcl12	74245	24322	0.328	chemokine (C-X-C motif) ligand 12
Cxcl3	5634	2721	0.483	chemokine (C-X-C motif) ligand 3
Cxcr7	3169	1152	0.363	chemokine (C-X-C motif) receptor 7
Cyb5r3	11505	5634	0.490	cytochrome b5 reductase 3
Cybasc3	2210	955	0.432	cytochrome b, ascorbate dependent 3
Cyhr1	4068	1278	0.314	cysteine and histidine rich 1
Cyp4a8	27746	10297	0.371	cytochrome P450, family 4, subfamily a, polypeptide 8
Cyth3	8719	3126	0.358	cytohesin 3
Da1-10	11191	4124	0.369	Da1-10-like
Daam1	2180	1010	0.463	dishevelled associated activator of morphogenesis 1
Dcn	26616	11666	0.438	decorin
Ddx46	898	419	0.467	DEAD (Asp-Glu-Ala-Asp) box polypeptide 46
Ddx52	1370	653	0.476	DEAD (Asp-Glu-Ala-Asp) box polypeptide 52
Depp1	2353	776	0.330	DEPP1, autophagy regulator
Dexi	2937	1243	0.423	dexamethasone-induced transcript
Dhodh	1380	605	0.438	dihydroorotate dehydrogenase (quinone)
Dhrs1	7858	2369	0.301	dehydrogenase/reductase (SDR family) member 1
Dhx9	2469	787	0.319	DEAH (Asp-Glu-Ala-His) box polypeptide 9
Dlgap5	2120	1038	0.490	discs, large (Drosophila) homolog-associated protein 5
Dnajc10	3616	1734	0.480	DnaJ (Hsp40) homolog, subfamily C, member 10
Dnase1l1	6608	3126	0.473	deoxyribonuclease 1-like 1
Dpep1	14766	7082	0.480	dipeptidase 1 (renal)
Dpep2	512	146	0.285	dipeptidase 2
Dpp8	5293	2521	0.476	dipeptidylpeptidase 8
Dse	929	440	0.473	dermatan sulfate epimerase
Dsel	588	292	0.497	dermatan sulfate epimerase-like
Dusp6	13401	6295	0.470	dual specificity phosphatase 6
Dut	1924	719	0.374	deoxyuridine triphosphatase
E2f1	2150	942	0.438	E2F transcription factor 1
Ech1	6427	2665	0.415	enoyl CoA hydratase 1, peroxisomal
Eci2	4360	1820	0.418	enoyl-Coenzyme A delta isomerase 2
Edem2	455	208	0.457	ER degradation enhancer, mannosidase alpha-like 2
Edn1	898	218	0.243	endothelin 1
Ehd2	3769	1795	0.476	EH-domain containing 2
Eln	3902	449	0.115	elastin
Elovl6	2226	1082	0.486	ELOVL family member 6, elongation of long chain fatty acids (yeast)
Emilin1	23822	9476	0.398	elastin microfibril interfacer 1
Emx2	2702	1201	0.444	empty spiracles homeobox 2
Ephb6	1820	765	0.420	Eph receptor B6
Epn3	1218	280	0.230	epsin 3
Epyc	11037	449	0.041	epiphycan
Ereg	1184	584	0.493	epiregulin
Erp29	4421	1898	0.429	endoplasmic reticulum protein 29
Espl1	765	267	0.349	extra spindle pole bodies homolog 1
Extl2	478	204	0.426	exostoses (multiple)-like 2
Fam180a	523	90	0.172	family with sequence similarity 180, member A
Fam25a	1176	431	0.366	family with sequence similarity 25, member A
Fam43a	2062	760	0.369	family with sequence similarity 43, member A
Fam57b	1305	648	0.497	family with sequence similarity 57, member B
Fam83d	630	201	0.319	family with sequence similarity 83, member D
Fasn	6700	2628	0.392	fatty acid synthase
Fat4	2288	771	0.337	FAT tumor suppressor homolog 4
Faxdc2	2353	1067	0.454	fatty acid hydroxylase domain containing 2

Table S6B continued

Symbol	Fb	Fb+AS-Tspan8-TEX	Fb+AS-Tsp8-TEX : Fb	GeneName
Fbln5	3214	695	0.216	fibulin 5
Fbxo21	2048	1017	0.497	F-box protein 21
Fbxo23	2721	719	0.264	F-box only protein 23
Fbxw9	680	340	0.500	F-box and WD repeat domain containing 9
Fcgbpl1	484	236	0.486	Fc fragment of IgG binding protein-like 1
Fdps	12417	5557	0.448	farnesyl diphosphate synthase
Fibin	12944	3397	0.262	fin bud initiation factor homolog
Fkbp7	10016	3641	0.363	FK506 binding protein 7
Fkrp	1038	410	0.395	fukutin related protein
Fmo3	724	181	0.250	flavin containing monooxygenase 3
Foxf1	553	198	0.358	forkhead box F1
Foxm1	1003	455	0.454	forkhead box M1
Foxo4	1003	471	0.470	forkhead box O4
Fut8	910	347	0.382	fucosyltransferase 8 (alpha (1,6) fucosyltransferase)
Fxyd1	1885	549	0.291	FXYD domain-containing ion transport regulator 1
Fzd1	4068	1722	0.423	frizzled family receptor 1
Gale	605	296	0.490	UDP-galactose-4-epimerase
Galnt11	820	271	0.330	UDP-N-acetyl-α-D-galactos.:polypeptide N-acetylgalactos.transfer.11
Galnt16	1333	622	0.467	polypeptide N-acetylgalactosaminyltransferase 16
Galnt2	2876	996	0.346	UDP-N-acetyl-α-D-galactos.:polypeptide N-acetylgalactos.transfer. 2
Gas1	49667	4973	0.100	growth arrest-specific 1
Gas6	30786	11268	0.366	growth arrest specific 6
Gchfr	14362	4673	0.325	GTP cyclohydrolase I feedback regulator
Gemin4	653	294	0.451	gem (nuclear organelle) associated protein 4
Ggh	4240	1663	0.392	gamma-glutamyl hydrolase (folylpolygammaglutamyl hydrolase)
Gins1	1458	377	0.259	GINS complex subunit 1 (Psf1 homolog)
Gja4	760	350	0.460	gap junction protein, alpha 4
Glipr1	18433	6937	0.376	GLI pathogenesis-related 1
Glrx1	7033	3191	0.454	glutaredoxin 1
Gls2	2077	1003	0.483	glutaminase 2 (liver, mitochondrial)
Glt8d1	2721	1017	0.374	glycosyltransferase 8 domain containing 1
Glt8d2	1003	360	0.358	glycosyltransferase 8 domain containing 2
Gltp	5293	2521	0.476	glycolipid transfer protein
Glul	1090	461	0.423	glutamate-ammonia ligase
Gna14	3259	246	0.075	guanine nucleotide binding protein, alpha 14
Gpr89b	2048	962	0.470	G protein-coupled receptor 89
Gpsm1	8481	2452	0.289	G-protein signaling modulator 1
Gpsm2	2916	1278	0.438	G-protein signaling modulator 2
Gpx7	7697	2006	0.261	glutathione peroxidase 7
Grem1	65536	26616	0.406	gremlin 1
Gsn	26987	12161	0.451	gelsolin
Gsta3	63744	24662	0.387	glutathione S-transferase A3
Gsta4	19756	8079	0.409	glutathione S-transferase alpha 4
Gsta5	25006	8964	0.358	glutathione S-transferase Yc2 subunit
Gsta5	2647	989	0.374	glutathione S-transferase alpha 5
Gsta6	1060	355	0.334	glutathione S-transferase alpha 6
Gstm1	3350	1663	0.497	glutathione S-transferase mu 1
Gstm2	6747	1687	0.250	glutathione S-transferase mu 2
Gstm3	709	300	0.423	glutathione S-transferase, mu 7
Gstm5	2610	1184	0.454	glutathione S-transferase, mu 5
Gusb	2091	553	0.264	glucuronidase, beta
H2afx	534	261	0.490	H2A histone family, member X
Hadh	11037	3083	0.279	hydroxyacyl-CoA dehydrogenase
Hapln3	855	405	0.473	hyaluronan and proteoglycan link protein 3
Hexim1	5793	2369	0.409	hexamethylene bis-acetamide inducible 1
Hmgb3	592	226	0.382	high mobility group box 3
Hmgcr	600	220	0.366	3-hydroxy-3-methylglutaryl-CoA reductase
Hmgcs1	12503	4270	0.342	3-hydroxy-3-methylglutaryl-CoA synthase 1 (soluble)
Hopx	592	194	0.328	HOP homeobox

Table S6B continued

Symbol	Fb	Fb+AS-Tspan8-TEX	Fb+AS-Tsp8-TEX : Fb	GeneName
Hp	2740	771	0.281	haptoglobin
Hs6st1	425	174	0.409	heparan sulfate 6-O-sulfotransferase 1
Hsbp1	12678	5557	0.438	heat shock factor binding protein 1
Hsdl2	584	278	0.476	hydroxysteroid dehydrogenase like 2
Hspa8	156956	73732	0.470	heat shock protein 8
Hyal2	6794	3061	0.451	hyaluronoglucosaminidase 2
Idi1	2759	1090	0.395	isopentenyl-diphosphate delta isomerase 1
Ids	5367	2353	0.438	iduronate 2-sulfatase
Igf2r	2628	898	0.342	insulin-like growth factor 2 receptor
Il11ra1	15076	4421	0.293	interleukin 11 receptor, alpha chain 1
Il34	1351	217	0.160	interleukin 34
Incenp	1992	676	0.339	inner centromere protein
Isy1	1552	765	0.493	ISY1 splicing factor homolog
Isyna1	1846	609	0.330	inositol-3-phosphate synthase 1
Itga1	9153	2978	0.325	integrin, alpha 1
Itga10	526	263	0.500	integrin, alpha 10
Itga8	676	258	0.382	integrin, alpha 8
Itgb5	13777	4360	0.316	integrin, beta 5
Itgbl1	22381	5008	0.224	integrin, beta-like 1
Izumo4	809	377	0.467	IZUMO family member 4
Jpt1	14766	6383	0.432	Jupiter microtubule associated homolog 1
Jup	729	307	0.420	junction plakoglobin
Kazald1	40063	5518	0.138	Kazal-type serine peptidase inhibitor domain 1
Kcnk2	5914	2435	0.412	potassium channel, subfamily K, member 2
Kdelc2	9345	4640	0.497	KDEL (Lys-Asp-Glu-Leu) containing 2
Kdelr3	942	440	0.467	KDEL (Lys-Asp-Glu-Leu) endopl.reticulum protein retention receptor 3
Kif11	1380	680	0.493	kinesin family member 11
Kif20a	4040	1017	0.252	kinesin family member 20A
Kif23	1641	648	0.395	kinesin family member 23
Kifc1	1370	592	0.432	kinesin family member C1
KIFC2	1024	383	0.374	kinesin family member C2
Kitlg	9345	2837	0.304	KIT ligand
Klhl36	666	290	0.435	kelch-like 36
Kpna2	10441	2702	0.259	karyopherin alpha 2
Kprp	815	372	0.457	keratinocyte proline-rich protein
Krtap16-5	734	300	0.409	keratin associated protein 16-5
Krtap21-2	657	300	0.457	keratin associated protein 21-2
Laptm4a	9410	4330	0.460	lysosomal protein transmembrane 4 alpha
Laptm4b	2798	1314	0.470	lysosomal protein transmembrane 4 beta
Lbh	1144	505	0.441	limb bud and heart development
Lc17	218913	107204	0.490	myosin, light polypeptide 6, alkali, smooth and non-muscle-like
Leprel4	6339	2684	0.423	leprecan-like 4
Lgals1	104998	34160	0.325	lectin, galactoside-binding, soluble, 1
LOC100125364	2487	1144	0.460	hypothetical protein LOC100125364
LOC287274	3717	1808	0.486	sedlin-like
LOC497848	1031	452	0.438	hypothetical LOC497848
LOC499219	3956	1607	0.406	hypothetical protein LOC499219
LOC500227	592	249	0.420	hypothetical gene supported by BC079424
LOC500846	4673	2272	0.486	hypothetical protein LOC500846
LOC500959	6081	2665	0.438	triosephosphate isomerase
LOC688459	3717	1795	0.483	hypothetical protein LOC688459
LOC689574	26432	12331	0.467	hypothetical protein LOC689574
Lox	7281	3083	0.423	lysyl oxidase
Loxl1	7181	2195	0.306	lysyl oxidase-like 1
Lpar6	1060	458	0.432	lysophosphatidic acid receptor 6
Lrif1	2837	1389	0.490	ligand dependent nuclear receptor interacting factor 1
Lrp11	2557	1017	0.398	low density lipoprotein receptor-related protein 11
Lum	7697	576	0.075	lumican
Lurap1l	434	168	0.387	leucine rich adaptor protein 1-like

Table S6B continued

Symbol	Fb	Fb+AS-Tspan8-TEX	Fb+AS-Tsp8-TEX : Fb	GeneName
Lypla2	9878	4211	0.426	lysophospholipase 2
Lztfl1	471	208	0.441	leucine zipper transcription factor-like 1
Maged2	5833	2226	0.382	melanoma antigen, family D, 2
Manba	1965	771	0.392	mannosidase, beta A, lysosomal
March2	16613	7697	0.463	membrane-associated ring finger (C3HC4) 2
Marcksl1	5673	2837	0.500	MARCKS-like 1
Mbtps1	14869	6985	0.470	membrane-bound transcription factor peptidase, site 1
Mcm3	1209	508	0.420	minichromosome maintenance complex component 3
Mcm3	4608	1710	0.371	minichromosome maintenance complex component 3
Mcm5	792	317	0.401	minichromosome maintenance complex component 5
Mcm6	596	251	0.420	minichromosome maintenance complex component 6
Me1	4068	1209	0.297	malic enzyme 1, NADP(+)-dependent, cytosolic
Med11	2452	1184	0.483	mediator complex subunit 11
Med18	1399	609	0.435	mediator complex subunit 18
Med23	849	410	0.483	mediator complex subunit 23
Medag	1808	809	0.448	mesenteric estrogen-dependent adipogenesis
Melk	617	251	0.406	maternal embryonic leucine zipper kinase
Meox2	8841	1924	0.218	mesenchyme homeobox 2
Mesdc2	3743	1846	0.493	mesoderm development candidate 2
Mex3b	413	194	0.470	mex3 homolog B
Mgarp	1965	724	0.369	mitochondria-localized glutamic acid-rich protein
Mgat1	5634	2592	0.460	mannosyl (α-1,3-)-glycoprotein β-1,2-N-acetylglucosaminyltransferase
Mgst3	2210	313	0.142	microsomal glutathione S-transferase 3
Mif4gd	5713	2385	0.418	MIF4G domain containing
Mms22l	929	416	0.448	MMS22-like, DNA repair protein
Mogat2	443	159	0.358	monoacylglycerol O-acyltransferase 2
Mon1b	719	338	0.470	MON1 homolog b
Mphosph6	2257	1017	0.451	M phase phosphoprotein 6
Mrc2	739	254	0.344	mannose receptor, C type 2
mrpl11	8364	3444	0.412	mitochondrial ribosomal protein L11
Mrps12	5914	2817	0.476	mitochondrial ribosomal protein S12
Mrps17	4576	1458	0.319	mitochondrial ribosomal protein S17
Mrps21	11037	4300	0.390	mitochondrial ribosomal protein S21
Mrps21l	2020	898	0.444	mitochondrial ribosomal protein S21-like
Mrvi1	6608	1017	0.154	murine retrovirus integration site 1 homolog
Ms4a7	481	154	0.321	membrane-spanning 4-domains, subfamily A, member 7
Mtbp	1675	648	0.387	MDM2 binding protein
Mvd	4482	1951	0.435	mevalonate (diphospho) decarboxylase
Mxd4	2304	936	0.406	Max dimerization protein 4
Mybl2	516	165	0.321	myeloblastosis oncogene-like 2
Mydgf	10587	5113	0.483	myeloid-derived growth factor
Myh8	9027	2504	0.277	myosin, heavy chain 8, skeletal muscle, perinatal
Myhc	7591	1978	0.261	myosin, heavy polypeptide 1, skeletal muscle, adult
Myl6	152664	73223	0.480	myosin, light chain 6, alkali, smooth muscle and non-muscle
Mylk	3956	1641	0.415	myosin light chain kinase
Mylk3	501	236	0.470	myosin light chain kinase 3
Mzf1	917	428	0.467	myeloid zinc finger 1
Narf	2647	1296	0.490	nuclear prelamin A recognition factor
Ncaph	1859	798	0.429	non-SMC condensin I complex, subunit H
Nck2	1992	729	0.366	NCK adaptor protein 2
Ndc80	3104	1105	0.356	NDC80 homolog, kinetochore complex component
Nde1	3616	1574	0.435	nudE nuclear distribution gene E homolog 1
Ndrg2	2978	1136	0.382	N-myc downstream regulated gene 2
Ndrg4	5673	1596	0.281	N-myc downstream regulated gene 4
Ndufa4	17682	7281	0.412	NADH dehydrogenase (ubiquinone) 1 alpha subcomplex, 4
Nelf	12417	5634	0.454	nasal embryonic LHRH factor
Nfkbiz	560	234	0.418	nuclear factor κ light polypeptide gene enhancer in B-cells inhibitor, ζ
Nid2	4421	838	0.189	nidogen 2
Nmrk1,Nrk1	1924	478	0.248	nicotinamide riboside kinase 1

Table S6B continued

Symbol	Fb	Fb+AS-Tspan8-TEX	Fb+AS-Tsp8-TEX : Fb	GeneName
Nnat	1261	214	0.170	neuronatin
Nr2f1	530	211	0.398	nuclear receptor subfamily 2, group F, member 1
Nr2f2	4270	1783	0.418	nuclear receptor subfamily 2, group F, member 2
Ns5atp9	1060	292	0.275	NS5A (hepatitis C virus) transactivated protein 9
Nsun4	1168	564	0.483	NOP2/Sun domain family, member 4
Nudt1	949	208	0.219	nudix (nucleoside diphosphate linked moiety X)-type motif 1
Nudt12	861	410	0.476	nudix (nucleoside diphosphate linked moiety X)-type motif 12
Nudt14	1795	771	0.429	nudix (nucleoside diphosphate linked moiety X)-type motif 14
Nudt15	1992	572	0.287	nudix (nucleoside diphosphate linked moiety X)-type motif 15
Nuf2	1024	491	0.480	NUF2, NDC80 kinetochore complex component, homolog
Nxn	407	153	0.376	nucleoredoxin
Oat	24834	10960	0.441	ornithine aminotransferase
Oaz2	27939	11829	0.423	ornithine decarboxylase antizyme 2
Obfc1	4640	2304	0.497	oligonucleotide/oligosaccharide-binding fold containing 1
Ogn	29944	5367	0.179	osteoglycin
Ogt	2077	666	0.321	O-linked N-acetylglucosamine transferase
Olfml1	2048	388	0.189	olfactomedin-like 1
Olfml3	42939	19484	0.454	olfactomedin-like 3
Omd	11191	215	0.019	osteomodulin
Osr2	695	296	0.426	odd-skipped related 2
P3h1	11268	5008	0.444	prolyl 3-hydroxylase 1
Papss2	929	422	0.454	3\'-phosphoadenosine 5\'-phosphosulfate synthase 2
Patz1	1924	929	0.483	POZ (BTB) and AT hook containing zinc finger 1
Pcdh19	1859	724	0.390	protocadherin 19
Pcdhac2	2062	549	0.266	protocadherin alpha subfamily C, 2
Pcdhb19	1563	274	0.176	protocadherin beta 19
Pcdhga7	20311	10156	0.500	protocadherin gamma subfamily A, 7
Pcolce	198668	96618	0.486	procollagen C-endopeptidase enhancer
Pdgfra	32094	6985	0.218	platelet derived growth factor receptor, alpha polypeptide
Pdgfrl	1563	402	0.257	platelet-derived growth factor receptor-like
Pdk2	2062	936	0.454	pyruvate dehydrogenase kinase, isozyme 2
Pdp1	3397	1082	0.319	pyruvate dehyrogenase phosphatase catalytic subunit 1
Pfkm	4012	1468	0.366	phosphofructokinase, muscle
Phf19	2006	699	0.349	PHD finger protein 19
Pik3r1	2210	820	0.371	phosphoinositide-3-kinase, regulatory subunit 1 (alpha)
Pimreg	2896	1184	0.409	PICALM interacting mitotic regulator
Pip5k1c	3468	1399	0.403	phosphatidylinositol-4-phosphate 5-kinase, type I, gamma
Pla2g16	1136	413	0.363	phospholipase A2, group XVI
Pla2g2a	719	146	0.203	phospholipase A2, group IIA (platelets, synovial fluid)
Plk1	4300	1596	0.371	polo-like kinase 1
Pls3	3591	1641	0.457	plastin 3
Pnn	1783	855	0.480	pinin, desmosome associated protein
Pnrc1	1758	671	0.382	proline-rich nuclear receptor coactivator 1
Podnl1	576	126	0.219	podocan-like 1
Pofut2	3795	1531	0.403	protein O-fucosyltransferase 2
Polr3gl	2937	1399	0.476	polymerase (RNA) III (DNA directed) polypeptide G-like
Pomgnt2	592	276	0.467	protein O-linked mannose N-acetylglucosaminyltransfer.2 (beta 1,4-)
Pon2	5221	1808	0.346	paraoxonase 2
Ppa2	7486	3717	0.497	pyrophosphatase (inorganic) 2
Ppap2b	2574	820	0.319	phosphatidic acid phosphatase type 2B
Ppih	917	391	0.426	peptidylprolyl isomerase H (cyclophilin H)
Ppp2r3b	3566	1783	0.500	protein phosphatase 2, regulatory subunit B", beta
Ppt1	10226	4905	0.480	palmitoyl-protein thioesterase 1
Prdx4	6937	3304	0.476	peroxiredoxin 4
Prelp	6517	3083	0.473	proline/arginine-rich end leucine-rich repeat protein
Prpf4b	1428	653	0.457	PRP4 pre-mRNA processing factor 4 homolog B
Prss23	13970	4451	0.319	protease, serine, 23
Psen2	5257	2226	0.423	presenilin 2
Psmb10	17805	7132	0.401	proteasome (prosome, macropain) subunit, beta type 10

Table S6B continued

Symbol	Fb	Fb+AS-Tspan8-TEX	Fb+AS-Tsp8-TEX : Fb	GeneName
Psme4	2288	1003	0.438	proteasome (prosome, macropain) activator subunit 4
Ptn	1136	130	0.114	pleiotrophin
Ptprf	18690	8422	0.451	protein tyrosine phosphatase, receptor type, F
Ptprs	1278	592	0.463	protein tyrosine phosphatase, receptor type, S
Ptrhd1	2684	942	0.351	peptidyl-tRNA hydrolase domain containing 1
Pttg1	12246	4451	0.363	pituitary tumor-transforming 1
Ptx3	949	335	0.354	pentraxin 3, long
Purb	3397	1342	0.395	purine rich element binding protein B
Rab26	1698	605	0.356	RAB26, member RAS oncogene family
Rad54l	580	242	0.418	RAD54 like
Rbfox1	1885	410	0.218	RNA binding protein, fox-1 homolog 1
Rbm12	1938	468	0.241	RNA binding motif protein 12
Rbp1	43238	16962	0.392	retinol binding protein 1, cellular
Rcn3	2702	1252	0.463	reticulocalbin 3, EF-hand calcium binding domain
Reck	5480	2077	0.379	reversion-inducing-cysteine-rich protein with kazal motifs
Rfc3	3019	1458	0.483	replication factor C (activator 1) 3
RGD1306227	639	282	0.441	similar to 4833420G17Rik protein
RGD1307929	18433	8481	0.460	similar to CG14967-PA
RGD1309534	455	226	0.497	similar to RIKEN cDNA 4931406C07
RGD1311946	4153	1287	0.310	similar to RIKEN cDNA 1810055G02
RGD1561381	2062	294	0.143	similar to microsomal glutathione S-transferase 3
Rgma	9742	2876	0.295	RGM domain family, member A
Romo1	41765	16613	0.398	reactive oxygen species modulator 1
Rpa3	1924	644	0.334	replication protein A3
Rrm1-ps1	729	362	0.497	ribonucleotide reductase M1, pseudogene 1
Rrm2	17560	5997	0.342	ribonucleotide reductase M2
Rspo1	3421	1370	0.401	R-spondin 1
Rsu1	9742	4837	0.497	Ras suppressor protein 1
Rtl8b	5753	2856	0.497	retrotransposon Gag like 8B
S100a1	4330	1438	0.332	S100 calcium binding protein A1
S100a10	75805	36107	0.476	S100 calcium binding protein A10
S100a13	5008	2419	0.483	S100 calcium binding protein A13
S100a4	269514	72214	0.268	S100 calcium-binding protein A4
Sap25	630	274	0.435	Sin3A-associated protein 25
Sapcd2	3616	1607	0.444	suppressor APC domain containing 2
Scara3	8780	2150	0.245	scavenger receptor class A, member 3
Scara5	13216	5185	0.392	scavenger receptor class A, member 5 (putative)
Scarf2	4771	1520	0.319	scavenger receptor class F, member 2
Scd	20032	8306	0.415	stearoyl-CoA desaturase (delta-9-desaturase)
Scfd2	1278	488	0.382	sec1 family domain containing 2
Scp2	30362	7383	0.243	sterol carrier protein 2
Sdf2	11585	4421	0.382	stromal cell derived factor 2
Selenom	14067	6383	0.454	selenoprotein M
Sept4	410	202	0.493	septin 4
Sertad3	820	292	0.356	SERTA domain containing 3
Sertad4	17199	3769	0.219	SERTA domain containing 4
Sesn3	771	236	0.306	sestrin 3
Sfrp2	792	169	0.213	secreted frizzled-related protein 2
Sgcb	1820	855	0.470	sarcoglycan, beta (dystrophin-associated glycoprotein)
Sgk196	2320	815	0.351	protein kinase-like protein SgK196
Shcbp1	704	317	0.451	SHC binding and spindle associated 1
Sipa1	1872	781	0.418	signal-induced proliferation-associated 1
Ska1	867	355	0.409	spindle and kinetochore associated complex subunit 1
Skp1	7858	3666	0.467	S-phase kinase-associated protein 1
Slc16a3	5873	2241	0.382	solute carrier family 16, member 3 (monocarboxylic acid transporter 4)
Slc22a17	3541	1541	0.435	solute carrier family 22, member 17
Slc35a2	1951	832	0.426	solute carrier family 35 (UDP-galactose transporter), member A2
Slc35f5	861	380	0.441	solute carrier family 35, member F5
Slc44a1	962	365	0.379	solute carrier family 44, member 1

Table S6B continued

Symbol	Fb	Fb+AS-Tspan8-TEX	Fb+AS-Tsp8-TEX : Fb	GeneName
Slc6a6	1226	209	0.171	solute carrier family 6 (neurotransmitter transporter, taurine), member 6
Slirp	6608	1938	0.293	SRA stem-loop interacting RNA binding protein
Smarcc2	1872	440	0.235	SWI/SNF rel., matrix assoc., actin dep.reg.chromat., subfam.c, memb.2
Smim7	9216	3191	0.346	small integral membrane protein 7
Snn	3517	1641	0.467	stannin
Snx14	3641	1687	0.463	sorting nexin 14
Sod3	40905	15393	0.376	superoxide dismutase 3, extracellular
Sox4	13308	6608	0.497	SRY (sex determining region Y)-box 4
Spag5	2304	1067	0.463	sperm associated antigen 5
Sparc	150562	65083	0.432	secreted protein, acidic, cysteine-rich (osteonectin)
Spc25	1641	755	0.460	SPC25, NDC80 kinetochore complex component, homolog
Spint2	8599	4124	0.480	serine peptidase inhibitor, Kunitz type, 2
Sptbn1	2062	734	0.356	spectrin, beta, non-erythrocytic 1
Srgn	1652	560	0.339	serglycin
Srp14	10514	5078	0.483	signal recognition particle 14
Srpx	17080	4124	0.241	sushi-repeat-containing protein, X-linked
Srpx2	36107	8422	0.233	sushi-repeat-containing protein, X-linked 2
Srrt	1031	443	0.429	serrate, RNA effector molecule
Srsf5	9608	4390	0.457	serine/arginine-rich splicing factor 5
Srsf6	4240	1911	0.451	serine/arginine-rich splicing factor 6
Ssc5d	4211	1370	0.325	scavenger receptor cysteine rich domain containing (5 domains)
St14	724	267	0.369	suppression of tumorigenicity 14 (colon carcinoma)
St3gal5	2369	843	0.356	ST3 beta-galactoside alpha-2,3-sialyltransferase 5
Stard4	410	124	0.301	StAR-related lipid transfer (START) domain containing 4
Stard9	2402	1193	0.497	StAR-related lipid transfer domain containing 9
Sub1	10735	5149	0.480	SUB1 homolog (S. cerevisiae)
Sult1a1	671	105	0.157	sulfotransferase family, cytosolic, 1A, phenol-preferring, member 1
Sumf2	1323	365	0.275	sulfatase modifying factor 2
Suox	556	224	0.403	sulfite oxidase
Surf4	12331	6165	0.500	surfeit 4
Tacc3	6985	2320	0.332	transforming, acidic coiled-coil containing protein 3
Tax1bp3	25355	8964	0.354	Tax1 (human T-cell leukemia virus type I) binding protein 3
Tcaf1	765	360	0.470	TRPM8 channel-associated factor 1
Tcf19	10885	3517	0.323	transcription factor 19
Tcp11l2	955	234	0.245	t-complex 11 (mouse) like 2
Tect2	1820	666	0.366	tectonic 2
Tex19	3083	1361	0.441	testis expressed 19
Thbs2	519	145	0.279	thrombospondin 2
Tk1	4390	1168	0.266	thymidine kinase 1, soluble
Tlr2	6889	2288	0.332	toll-like receptor 2
Tm4sf1	38166	3421	0.090	transmembrane 4 L six family member 1
Tmem123	1978	976	0.493	transmembrane protein 123
Tmem2	437	185	0.423	transmembrane protein 2
Tmem204	923	355	0.384	transmembrane protein 204
Tmem216	449	201	0.448	transmembrane protein 216
Tmem50b	1323	413	0.312	transmembrane protein 50B
Tnfrsf11b	2487	556	0.224	tumor necrosis factor receptor superfamily, member 11b
Tnfrsf9	455	162	0.356	tumor necrosis factor receptor superfamily, member 9
Tnn	1468	505	0.344	tenascin N
Top2a	1978	584	0.295	topoisomerase (DNA) II alpha
Topbp1	787	375	0.476	topoisomerase (DNA) II binding protein 1
Tpx2	580	290	0.500	TPX2, microtubule-associated, homolog
Tradd	7132	3566	0.500	TNFRSF1A-associated via death domain
Traf4af1	7281	1489	0.204	TRAF4 associated factor 1
Trak2	4124	1687	0.409	trafficking protein, kinesin binding 2
Trib2	1097	191	0.174	tribbles homolog 2
Trim25	4096	1820	0.444	tripartite motif-containing 25
Tspan2	1128	523	0.463	tetraspanin 2
Tspan7	5480	2320	0.423	tetraspanin 7

Table S6B continued

Symbol	Fb	Fb+AS-Tspan8-TEX	Fb+AS-Tsp8-TEX : Fb	GeneName
Ttc30b	1734	685	0.395	tetratricopeptide repeat domain 30B
Ttyh3	2020	617	0.306	tweety homolog 3
Tuba1a	38431	7804	0.203	tubulin, alpha 1A
Tubb4b	74245	25888	0.349	tubulin, beta 4B class Ivb
Tubb5	70728	34877	0.493	tubulin, beta 5 class I
Txnip	3214	714	0.222	thioredoxin interacting protein
Ube2c	1911	803	0.420	ubiquitin-conjugating enzyme E2C
Ubl5	1924	891	0.463	ubiquitin-like 5
Ufc1	5518	2721	0.493	ubiquitin-fold modifier conjugating enzyme 1
Uqcrh	31216	7913	0.253	ubiquinol-cytochrome c reductase hinge protein
Vamp1	1144	498	0.435	vesicle-associated membrane protein 1
Vcam1	37381	4012	0.107	vascular cell adhesion molecule 1
Vkorc1	28526	13873	0.486	vitamin K epoxide reductase complex, subunit 1
Vof16	744	338	0.454	ischemia related factor vof-16
Vwa1	1746	809	0.463	von Willebrand factor A domain containing 1
Wdr53	3259	1629	0.500	WD repeat domain 53
Xdh	3083	1333	0.432	xanthine dehydrogenase
Yipf2	1380	534	0.387	Yip1 domain family, member 2
Ylpm1	630	307	0.486	YLP motif containing 1
Yrdc	4330	1872	0.432	yrdC domain containing
Zbtb8os	505	249	0.493	zinc finger and BTB dom.containing 8 opposite strand, pseudogene 1
Zc3h11a	4360	1992	0.457	zinc finger CCCH-type containing 11A
Zdhhc16	13682	6794	0.497	zinc finger, DHHC-type containing 16
Zfp846	468	234	0.500	zinc finger protein 846
Znf24	1629	639	0.392	zinc finger protein 191
Zscan2	739	367	0.497	zinc finger and SCAN domain containing 2
Zscan21	4124	1859	0.451	zinc finger and SCAN domain containing 21

Table S6C

AS-Tspan8-TEX coculture-induced EC mRNA upregulation

Symbol[a]	EC	EC+Tspan8-TEX	EC+Tspan8-TEX : EC	GeneName
Aarsd1	179	457	2.561	alanyl-tRNA synthetase domain containing 1
Abca16	172	807	4.680	ATP-binding cassette, subfamily A (ABC1), member 16
Adap1	357	898	2.516	ArfGAP with dual PH domains 1
Add3	885	2361	2.667	adducin 3 (gamma)
Agps	1607	4716	2.935	alkylglycerone phosphate synthase
Ahdc1	1060	2198	2.073	AT hook, DNA binding motif, containing 1
Ahi1	609	1548	2.543	Abelson helper integration site 1
Akap17b	207	423	2.047	A kinase (PRKA) anchor protein 17B
Apol9a	2195	4889	2.227	apolipoprotein L 9a
Arhgap28	622	1687	2.713	Rho GTPase activating protein 28
Arid3a	530	2548	4.807	AT rich interactive domain 3A (Bright like)
Atp8b2	317	835	2.631	Atpase, class I, type 8B, member 2
Bcl11a	377	1746	4.627	B-cell CLL/lymphoma 11A (zinc finger protein)
Bf	1226	3920	3.196	complement factor B
Bicc1	488	1113	2.282	bicaudal C homolog 1
Bmp2	1046	2136	2.043	bone morphogenetic protein 2
Bpifb6	150	428	2.849	BPI fold containing family B, member 6
C2cd4b	195	471	2.412	C2 calcium-dependent domain containing 4B
C4b	6700	13732	2.049	complement component 4B (Chido blood group)
Cables1	1687	3568	2.115	Cdk5 and Abl enzyme substrate 1
Caly	153	434	2.832	calcyon neuron-specific vesicular protein
Ccdc69	177	592	3.341	coiled-coil domain containing 69
Ccl19	326	662	2.028	chemokine (C-C motif) ligand 19
Cd24	278	1000	3.593	CD24 molecule
CD244	150	447	2.980	similar to transmembrane NK cell receptor 2B4
Cd99l2	1218	6548	5.378	similar to MIC2 like 1
Cdkn1a	11829	32094	2.713	cyclin-dependent kinase inhibitor 1A
Cep250	6208	20455	3.295	centrosomal protein 250
Ces1a	1226	2897	2.363	carboxylesterase 1A
Chd2	685	1511	2.205	chromodomain helicase DNA binding protein 2
Chm	333	1185	3.556	choroideremia (Rab escort protein 1)
Clec12a	181	479	2.648	C-type lectin domain family 12, member A
Clrn2	217	555	2.558	clarin 2
Cotl1	3421	7281	2.129	coactosin-like 1 (Dictyostelium)
Cox6b2	3717	9126	2.455	cytochrome c oxidase subunit VIb polypeptide 2
Cplx2	622	1287	2.071	complexin 2
Cpne5	254	524	2.060	copine V
Csn1s1	223	455	2.042	casein alpha s1
Ctnnal1	1938	10886	5.618	catenin (cadherin associated protein), alpha-like 1
Cx3cl1	17805	41621	2.338	chemokine (C-X3-C motif) ligand 1
Cx3cr1	1128	2423	2.147	chemokine (C-X3-C motif) receptor 1
Cxcl10	2647	7860	2.970	chemokine (C-X-C motif) ligand 10
Dcaf6	530	1176	2.219	DDB1 and CUL4 associated factor 6
Dcun1d1	3875	9027	2.330	DCN1, defective in cullin neddylation 1, domain containing 1
Dgkd	205	425	2.071	diacylglycerol kinase, delta
Duox1	286	676	2.362	dual oxidase 1
Dusp16	242	496	2.049	dual specificity phosphatase 16
Eef1a2	750	1655	2.207	eukaryotic translation elongation factor 1 alpha 2
Eln	5043	12767	2.532	elastin
Emx1	278	747	2.685	empty spiracles homeobox 1
Eral1	484	1141	2.355	Era (G-protein)-like
Ermard	498	1072	2.153	ER membrane-assocciated RNA degradation
Etv3	879	3541	4.028	ets variant 3
Fam131b	1113	2312	2.078	family with sequence similarity 131, member B
Fam13a	221	532	2.403	family with sequence similarity 13, member A
Flad1	393	847	2.152	flavin adenine dinucleotide synthetase
Fosb	714	2548	3.568	FBJ osteosarcoma oncogene B
Foxd4	205	458	2.235	forkhead box D4

Table S6C continued

Symbol	EC	EC+Tspan8-TEX	EC+Tspan8-TEX : EC	GeneName
Foxe1	4513	16787	3.719	forkhead box E1 (thyroid transcription factor 2)
Fzd6	271	787	2.908	frizzled family receptor 6
Gal3st4	405	1399	3.458	galactose-3-O-sulfotransferase 4
Galp	170	400	2.354	galanin-like peptide
Gfra2	177	712	4.014	GDNF family receptor alpha 2
Grin2d	22073	57451	2.603	glutamate receptor, ionotropic, N-methyl D-aspartate 2D
H2afj	21321	60939	2.858	H2A histone family, member J
Hdc	320	942	2.949	histidine decarboxylase
Hes7	21028	66451	3.160	hairy and enhancer of split 7
Hist1h1a	347	1283	3.695	histone cluster 1, H1a
Hist1h1b	2837	11465	4.042	histone cluster 1, H1b
Hist1h1d	3743	14416	3.852	histone cluster 1, H1d
Hist1h2af	21174	64414	3.042	histone cluster 1, H2af
Hist1h2ai	260	683	2.630	histone cluster 1, H2ai
Hist1h2ail1	13970	34173	2.446	histone cluster 1, H2ai-like
Hist1h2ak	24492	82953	3.387	histone cluster 1, H2ak
Hist1h2an	229	748	3.264	histone cluster 1, H2an
Hist1h2bd	1184	2713	2.290	histone cluster 1, H2bm
Hist1h3c	1201	2788	2.322	histone cluster 2, H3c
Hist1h4b	2469	5386	2.181	histone cluster 1, H4b
Hist2h2ac	9947	30684	3.085	histone cluster 2, H2ac
Hist2h3c2	2195	6540	2.979	histone cluster 2, H3c2
Hist4h4	2759	7513	2.723	histone cluster 2, H4
Hmx1	36358	95624	2.630	H6 family homeobox 1
Homer2	419	1168	2.790	homer homolog 2 (Drosophila)
Hoxa11-as/nc[b]	545	1479	2.713	similar to hypothetical gene supported by BC025338
Hpn	910	1997	2.194	hepsin
Ifi35	2048	4285	2.092	interferon-induced protein 35
Ifit3	186	752	4.042	interferon-induced protein with tetratricopeptide repeats 3
Il1f10	662	1705	2.577	interleukin 1 family, member 10
Il34	1305	2731	2.092	interleukin 34
Iqgap1	1428	2999	2.100	IQ motif containing GTPase activating protein 1
Irf7	1380	4514	3.272	interferon regulatory factor 7
Isg15	1541	9027	5.856	ISG15 ubiquitin-like modifier
Jsrp1	1176	3456	2.938	junctional sarcoplasmic reticulum protein 1
Kcnma1	149	497	3.331	potassium large conductance calcium-activat.channel, subf.Mα1
Kcp	568	1671	2.941	kielin/chordin-like protein
Krtap1-1	182	609	3.343	keratin associated protein 1-3-like
Lbp	560	1230	2.196	lipopolysaccharide binding protein
LOC100366216	201	437	2.175	nuclear antigen Sp100-like
LOC298139	269	939	3.496	similar to RIKEN cDNA 2310003M01
LOC301748	228	556	2.446	similar to RIKEN cDNA 1700001E04
LOC678708	505	1641	3.250	similar to histone 1, H2ai
LOC680322	28329	93329	3.294	similar to Histone H2A type 1
LOC680578	324	1031	3.181	similar to C56C10.7a
LOC684998	252	568	2.248	hypothetical protein LOC684998
LOC689727	523	1065	2.036	hypothetical protein LOC689727
LOC690415	471	1218	2.586	hypothetical protein LOC690415
LOC690435	165	449	2.714	hypothetical protein LOC690435
Loxl2	1499	3226	2.152	lysyl oxidase-like 2
Ly6g5c	671	2675	3.987	lymphocyte antigen 6 complex, locus G5C
Mag	234	648	2.770	myelin-associated glycoprotein
Map3k20	413	830	2.010	sterile alpha motif and leucine zipper containing kinase AZK
Medag	1060	3405	3.212	similar to RIKEN cDNA 6330406I15
Mmp15	15393	52876	3.435	matrix metallopeptidase 15
Mmp28	352	848	2.407	matrix metallopeptidase 28
Mmtag2	2048	4127	2.015	similar to RIKEN cDNA 2310033P09
Mras	1144	2283	1.996	muscle RAS oncogene homolog
Mx1	304	2236	7.343	myxovirus (influenza virus) resistance 1

Table S6C continued

Symbol	EC	EC+Tspan8-TEX	EC+Tspan8-TEX : EC	GeneName
Napsa	549	1305	2.379	napsin A aspartic peptidase
Nes	223	512	2.298	nestin
Nr2f2	2385	4956	2.078	nuclear receptor subfamily 2, group F, member 2
Nrg4	150	441	2.938	neuregulin 4
Nsd2	338	907	2.686	Wolf-Hirschhorn syndrome candidate 1
Oas1a	129	424	3.290	2\'-5\' oligoadenylate synthetase 1A
Oasl2	280	917	3.272	2\'-5\' oligoadenylate synthetase-like 2
Olr143	161	490	3.046	olfactory receptor 143
Otx1	2916	10553	3.619	orthodenticle homeobox 1
Parp14	3281	7206	2.196	poly (ADP-ribose) polymerase family, member 14
Pcdhgc5	338	702	2.078	protocadherin gamma c5
Pdlim5	3492	9092	2.603	PDZ and LIM domain 5
Pdx1	284	923	3.250	pancreatic and duodenal homeobox 1
Pdxp	2180	4940	2.266	pyridoxal (pyridoxine, vitamin B6) phosphatase
Pgbd5	202	468	2.313	piggyBac transposable element derived 5
Pgpep1	3281	6941	2.115	pyroglutamyl-peptidase I
Pitx1	505	1014	2.008	paired-like homeodomain 1
Pla2g2a	8780	23582	2.686	phospholipase A2, group IIA (platelets, synovial fluid)
Pla2g2c	185	534	2.889	phospholipase A2, group IIC
Plk2	1144	2435	2.129	polo-like kinase 2
Polm	7231	27678	3.828	polymerase (DNA directed), mu
Polr1a	220	748	3.401	polymerase (RNA) I polypeptide A
Ppp1r14a	568	1351	2.379	protein phosphatase 1, regulatory (inhibitor) subunit 14A
Prkacb	215	603	2.799	protein kinase, cAMP dependent, catalytic, beta
Psd	5713	23905	4.184	pleckstrin and Sec7 domain containing
Psmb10	14766	30049	2.035	proteasome (prosome, macropain) subunit, beta type 10
Pth2	265	562	2.121	parathyroid hormone 2
Ptms	23010	50186	2.181	parathymosin
Rai12	5518	12374	2.242	retinoic acid induced 12
Rara	2020	4082	2.021	retinoic acid receptor, alpha
Rassf2	2288	5077	2.219	Ras association (RalGDS/AF-6) domain family member 2
Rbmx	1342	3328	2.480	RNA binding motif protein, X-linked
Rbp1	19893	40909	2.056	retinol binding protein 1, cellular
Rbp2	1846	3836	2.078	retinol binding protein 2, cellular
Rc3h2	755	1538	2.037	ring finger and CCCH-type domains 2
Rftn1	709	1464	2.064	raftlin lipid raft linker 1
RGD1308117	1128	2369	2.100	similar to 9930012K11Rik protein
RGD1310081	184	402	2.192	similar to hypothetical protein FLJ13231
RGD1561671	537	1829	3.403	similar to RIKEN cDNA 2900010M23
RGD1563034	347	1266	3.644	similar to ETS domain transcript.factor ERF (Ets2 repr.factor)
Rnpepl1	1160	2320	2.000	similar to arginyl aminopeptidase (aminopeptidase B)-like 1
Rpph1	1243	4405	3.543	RNA component of mitoch.RNA processing endoribonuclease
Rsad2	309	2402	7.781	radical S-adenosyl methionine domain containing 2
Rsph6a	184	495	2.696	radial spoke head 6 homolog A
Rtp4	704	2027	2.878	receptor (chemosensory) transporter protein 4
Sema5a	596	1269	2.129	sema dom., 7 thrombosp.rep., short cytopl.dom., semaphorin5A
Sema6c	22694	61586	2.714	sema dom., TM, cytopl. dom., semaphorin6C
Serpinb7	898	2470	2.751	serpin peptidase inhibitor, clade B (ovalbumin), member 7
Sertad3	1046	2093	2.002	SERTA domain containing 3
Sesn3	288	588	2.040	sestrin 3
Sez6	164	440	2.676	seizure related 6 homolog
Sfrp2	630	2203	3.494	secreted frizzled-related protein 2
Shbg	8719	17936	2.057	sex hormone binding globulin
Shisa8	474	1624	3.423	shisa homolog 8
Sin3a	292	832	2.848	SIN3 homolog A, transcription regulator
Sirpa	239	503	2.107	signal-regulatory protein alpha
Slc19a1	452	2183	4.831	solute carrier family 19 (folate transporter), member 1
Slc25a28	4973	10333	2.078	solute carrier family 25, member 28
Slc25a30	739	1515	2.049	solute carrier family 25, member 30

Table S6C continued

Symbol	EC	EC+Tspan8-TEX	EC+Tspan8-TEX : EC	GeneName
Slc38a7	391	938	2.401	solute carrier family 38, member 7
Slc4a2	3929	8780	2.235	solute carrier family 4 (anion exchanger), member 2
Snhg11	2592	6040	2.330	small nucleolar RNA host gene 11
Sobp	247	704	2.848	sine oculis-binding protein homolog
Spsb4	6383	19620	3.074	splA/ryanodine receptor domain and SOCS box containing 4
Stat2	3083	7282	2.362	signal transducer and activator of transcription 2
Ston1	246	746	3.036	stonin 1
Taok3	335	926	2.761	TAO kinase 3
Tbx21	27939	73551	2.633	T-box 21
Tcp11x2	205	596	2.907	similar to t-complex 11 protein
Tgm2	362	1485	4.101	transglutaminase 2, C polypeptide
Tlx3	187	424	2.260	T-cell leukemia, homeobox 3
Tmem121	1075	2488	2.315	transmembrane protein 121
Tmem229b	744	2238	3.007	transmembrane protein 229B
Tmem47	6841	14811	2.165	transmembrane protein 47
Tpbgl	12161	34043	2.799	similar to trophoblast glycoprotein
Tprn	491	1024	2.085	taperin
Trim42	201	934	4.652	tripartite motif-containing 42
Tsen54	1499	3541	2.362	tRNA splicing endonuclease 54 homolog
Tubb4a	3984	11547	2.898	tubulin, beta 4A class Iva
Uba7	372	1170	3.143	ubiquitin-like modifier activating enzyme 7
Uqcr11	18054	41485	2.298	ubiquinol-cytochrome c reductase, complex III subunit XI
Usp18	329	867	2.639	ubiquitin specific peptidase 18
Usp2	1771	5257	2.969	ubiquitin specific peptidase 2
Usp42	843	1754	2.080	ubiquitin specific peptidase 42
Utrn	215	557	2.585	utrophin
Vom1r57	288	740	2.568	vomeronasal 1 receptor 57
Vom1r61	405	823	2.035	vomeronasal 1 receptor 61
Wdr37	254	532	2.092	WD repeat domain 37
Wfdc1	568	1999	3.519	WAP four-disulfide core domain 1
Wfdc21	580	1305	2.251	WDNM1 homolog
withdrawn	431	873	2.028	similar to hypothetical protein MGC13138
Wnt7a	170	425	2.497	wingless-type MMTV integration site family, member 7A
Zbtb38	267	607	2.275	zinc finger and BTB domain containing 38
Zbtb7c	214	450	2.107	zinc finger and BTB domain containing 7C
Zbtb8b	666	2558	3.839	zinc finger and BTB domain containing 8b
Zdhhc14	284	707	2.488	zinc finger, DHHC-type containing 14
Zeb2	443	1136	2.567	zinc finger E-box binding homeobox 2
Zfp592	5914	15342	2.594	zinc finger protein 592
Zfp787	205	646	3.152	zinc finger protein 787
Znrf4	195	848	4.341	zinc and ring finger 4

Table S6D

AS-Tspan8-TEX coculture-induced EC mRNA downregulation

**Symbol**[a]	**EC**	**EC+Tspan8-TEX**	**EC+Tspan8-TEX : EC**	**GeneName**
Adam9	709	355	0.500	ADAM metallopeptidase domain 9
Adamts1	29738	8690	0.292	ADAM metallopeptidase with thrombospondin type 1 motif, 1
Ag2	605	301	0.497	similar to LOC387763 protein
Ankrd1	657	246	0.374	ankyrin repeat domain 1
Arc	699	183	0.262	activity-regulated cytoskeleton-associated protein
Areg	2336	547	0.234	amphiregulin
Arf4	8364	4098	0.490	ADP-ribosylation factor 4
Arrdc4	458	176	0.385	arrestin domain containing 4
Atf3	478	234	0.490	activating transcription factor 3
Atf4	38431	16271	0.423	activating transcript. factor 4 (tax-responsive enhancer element B67)
Atg12	505	248	0.491	ATG12 autophagy related 12 homolog
Bmper	530	203	0.383	BMP-binding endothelial regulator
Btg2	6039	719	0.119	BTG family, member 2
Btg3	744	358	0.481	BTG family, member 3
Cacybp	9281	4127	0.445	calcyclin binding protein
Ccdc112	584	281	0.481	coiled-coil domain containing 112
Ccl27	1978	952	0.481	chemokine (C-C motif) ligand 27
Ccnl1	2120	1043	0.492	cyclin L1
Cda	1885	885	0.470	cytidine deaminase
Cdkn2aip	617	288	0.467	CDKN2A interacting protein
Ceacam4	929	377	0.406	carcinoembryonic antigen-related cell adhesion molecule 10
Csrnp1	942	336	0.357	cysteine-serine-rich nuclear protein 1
Cst6	695	199	0.286	cystatin E/M
Ctxn3	2557	927	0.362	cortexin 3
Cyp3a9	3327	1218	0.366	cytochrome P450, family 3, subfamily a, polypeptide 9
Dars	5330	2470	0.463	aspartyl-tRNA synthetase
Diras1	1898	903	0.476	DIRAS family, GTP-binding RAS-like 1
Dusp1	1218	195	0.160	dual specificity phosphatase 1
Dusp22	630	205	0.325	dual specificity phosphatase 22
Dusp5	6841	2020	0.295	dual specificity phosphatase 5
Egr1	18179	7107	0.391	early growth response 1
Egr2	1675	283	0.169	early growth response 2
Eif2s2	2521	1075	0.426	eukaryotic translation initiation factor 2, subunit 2 beta
Eif3e	4738	1939	0.409	eukaryotic translation initiation factor 3, subunit E
Eif4e	1687	844	0.500	eukaryotic translation initiation factor 4E
Eif4e3	1017	493	0.485	eukaryotic translation initiation factor 4E family member 3
Ero1l	9281	4467	0.481	ERO1-like
Errfi1	5713	2531	0.443	ERBB receptor feedback inhibitor 1
Fem1b	867	431	0.497	fem-1 homolog b
Fos	7858	179	0.023	FBJ osteosarcoma oncogene
Fosl1	6039	2798	0.463	fos-like antigen 1
Gbe1	452	222	0.490	glucan (1,4-alpha-), branching enzyme 1
Gdf15	1585	609	0.384	growth differentiation factor 15
Gpr88	695	347	0.499	G-protein coupled receptor 88
Hif1a	826	213	0.258	hypoxia-inducible factor 1, alpha subunit (transcription factor)
Higd1a	787	389	0.495	HIG1 hypoxia inducible domain family, member 1A
Iars	5673	2639	0.465	isoleucyl-tRNA synthetase
Id1	910	422	0.463	inhibitor of DNA binding 1
Ier2	28725	12810	0.446	immediate early response 2
Il13ra2	2665	1101	0.413	interleukin 13 receptor, alpha 2
Il1rl1	16271	5853	0.360	interleukin 1 receptor-like 1
Insig1	8023	3796	0.473	insulin induced gene 1
Klf10	6295	2675	0.425	Kruppel-like factor 10
Klf4	16728	8030	0.480	Kruppel-like factor 4 (gut)
Kpna4	491	235	0.478	karyopherin alpha 4 (importin alpha 3)
Lnpk	873	430	0.493	limb and neural patterns
LOC100359930	512	195	0.381	Cyp2s1 protein-like
LOC303590	488	201	0.412	similar to cactin CG1676-PA

Table S6D continued

Symbol	EC	EC+Tspan8-TEX	EC+Tspan8-TEX : EC	GeneName
LOC689959	648	306	0.471	hypothetical protein LOC689959
Met	431	168	0.390	met proto-oncogene
Morf4l2	8135	4054	0.498	mortality factor 4 like 2
Mthfd2	4153	2077	0.500	methylenetetrahydrofolate dehydrog.2, -cyclohydrolase
Myc	4096	1965	0.480	myelocytomatosis oncogene
Nedd4	32768	16103	0.491	neural precursor cell expressed, developmentally down-regulated 4
Nfkbiz	1105	454	0.411	nuclear factor of κ light polypept. gene enhanc. in B-cells inhibitor, ζ
Nox1	1184	521	0.440	NADPH oxidase 1
Nr4a1	10960	1591	0.145	nuclear receptor subfamily 4, group A, member 1
Nr4a2	1235	350	0.284	nuclear receptor subfamily 4, group A, member 2
Nr4a3	501	145	0.289	nuclear receptor subfamily 4, group A, member 3
Nxph3	1629	644	0.395	neurexophilin 3
Oas1e	695	307	0.443	2\'-5\' oligoadenylate synthetase 1E
Ovol1	505	215	0.426	ovo-like 1(Drosophila)
Pdcd10	4837	2206	0.456	programmed cell death 10
Pdk1	1218	582	0.478	pyruvate dehydrogenase kinase, isozyme 1
Phlda1	750	324	0.433	pleckstrin homology-like domain, family A, member 1
Pls3	2702	1328	0.491	plastin 3
Plscr2	982	446	0.454	phospholipid scramblase 2
Pnrc1	1992	974	0.489	proline-rich nuclear receptor coactivator 1
Pnrc2	2628	1053	0.401	proline-rich nuclear receptor coactivator 2
Ppp1cb	5955	2812	0.472	protein phosphatase 1, catalytic subunit, beta isozyme
Psat1	17929	8339	0.465	phosphoserine aminotransferase 1
Psip1	867	422	0.487	PC4 and SFRS1 interacting protein 1
Psph	1082	540	0.499	phosphoserine phosphatase
Ptpn12	1296	557	0.429	protein tyrosine phosphatase, non-receptor type 12
Ptx3	4576	1878	0.410	pentraxin 3, long
Rap1a	7132	3304	0.463	RAP1A, member of RAS oncogene family
Rbm3	14165	7085	0.500	RNA binding motif (RNP1, RRM) protein 3
Rcor1	760	363	0.478	REST corepressor 1
Rgs2	2180	429	0.197	regulator of G-protein signaling 2
Rsl24d1	1075	491	0.457	ribosomal L24 domain containing 1
Sacm1l	879	374	0.425	SAC1 suppressor of actin mutations 1-like
Sept7	7132	3252	0.456	septin 7
Serp1	4182	1687	0.403	stress-associated endoplasmic reticulum protein 1
Slc12a2	3769	1885	0.500	solute carrier family 12 (sodium/potassium/chloride, member 2
Slc30a4	873	334	0.382	solute carrier family 30 (zinc transporter), member 4
Slc6a4	1872	549	0.293	solute carrier family 6 (neurotransmitter, serotonin), member 4
Slc7a11	498	185	0.371	solute carrier family 7 (anionic AA transporter light chain), memb.11
Slc7a5	776	375	0.483	solute carrier family 7 (amino acid transporter light chain), member 5
Snx7	2957	1454	0.492	sorting nexin 7
Spcs3	5595	2666	0.476	signal peptidase complex subunit 3 homolog
Spp1	27364	11952	0.437	secreted phosphoprotein 1
Sprr1a	572	271	0.474	small proline-rich protein 1A
Srxn1	8964	4436	0.495	sulfiredoxin 1
Tceal8	5257	2530	0.481	transcription elongation factor A (SII)-like 8
Tmem45a	3984	1872	0.470	transmembrane protein 45A
Trmt6	568	276	0.486	tRNA methyltransferase 6 homolog
Vldlr	1418	555	0.391	very low density lipoprotein receptor
Vps35	3591	1520	0.423	vacuolar protein sorting 35 homolog
Vsnl1	1243	570	0.459	visinin-like 1
Zdhhc2	832	402	0.484	zinc finger, DHHC-type containing 2
Zfp319	491	190	0.388	zinc finger protein 319
Zfp36	425	203	0.478	zinc finger protein 36

[a] red symbol: Fb and EC mRNA accordingly regulated by AS-TEX or AS-Tspan8-TEX, [b] nc: noncoding

Table S7

Altered miRNA recovery in TEX treated EC

name[a]	EC	EC+AS-TEX	AS-TEX:EC[b]
miR-7a	233	419	1.80
miR-125a-3p	290	577	1.99
miR-128	138	244	1.76
miR-146a	4669	7287	1.56
miR-146b	1437	3906	2.72
miR-150	344	528	1.53
miR-202	117	286	2.43
miR-210	2321	3700	1.59
miR-211	347	569	1.64
miR-322	1472	2278	1.55
miR-345-3p	51	289	5.62
miR-450a	136	247	1.82
miR-542-3p	81	207	2.55

name[a]	EC	EC+AS-Tspan8-TEX	AS-Tspan8-TEX:EC[b]
let-7i	15429	23790	1.54
miR-1188-3p	62	111	1.80
miR-146b	1437	3261	2.27
miR-181a	364	545	1.50
miR-207	66	300	4.55
miR-485	57	327	5.70
miR-760-5p	165	570	3.46
miR-764	245	520	2.12

name	EC	EC+AS-TEX	EC:AS-TEX
miR-32	601	117	5.14
miR-34c	2630	1470	1.79
miR-101a	2067	1341	1.54
miR-129	3654	1563	2.34
miR-140	7105	3591	1.98
miR-362	249	153	1.63
miR-374	313	138	2.26
miR-466b	1715	720	2.38
miR-466c	1052	463	2.27
miR-494	2733	1800	1.52
miR-500	596	360	1.66
miR-672	384	86	4.48
miR-1249	271	171	1.58

name	EC	EC+AS-Tspan8-TEX	EC:AS-Tspan8-TEX
miR-18a	302	168	1.80
miR-19a	428	210	2.04
miR-29b	28045	16235	1.73
miR-34b	2492	1189	2.10
miR-34c	2630	1640	1.60
miR-101a	2067	1332	1.55
miR-134	462	119	3.87
miR-140	7105	4181	1.70
miR-188	393	197	2.00
miR-193	1042	429	2.43
miR-211	347	199	1.74
miR-212	390	102	3.83
miR-214	7667	3831	2.00
miR-222	1694	1047	1.62
miR-301a	1143	557	2.05
miR-362	249	163	1.52
miR-494	2733	1576	1.73
miR-1224	2183	1360	1.60
miR-1249	271	98	2.76
miR-3584-5p	3975	1102	3.61

[a] corresponding changes in miRNA expression in AS- and AS-Tspan8-TEX-treated EC are indicated in red.
[b] 1.5-fold changes in miRNA signal strength were accepted as significant.

Table S8

Noncoding RNA in rat endothelial cells, fibroblasts and AS-Tspan8-TEX

symbol	classif.[1]	GeneName
AA926063	nc	AA926063gene
Abhd11-as1	nc	hypothetical protein LOC686120
Aff1-as1	as,coding	similar to hypothetical protein MGC26744
Aoc2-ps1	pseudo	amine oxidase, copper containing 2 (retina-specific), pseudogene 1
Atp5hl1	pseudo	ATP synthase, H+ transporting, mitochondrial Fo complex, subunit d-like 1
CB741658	nc	CB741658 gene
Cfl1	pseudo	similar to Cofilin-1 (Cofilin, non-muscle isoform)
Clec6a-ps1	pseudo	C-type lectin domain family 6, member A, pseudogene 1
Cox6c-ps1	pseudo	cytochrome c oxidase subunit VIc, pseudogene
Cyp3a85-ps	pseudo	cytochrome P450, family 3, subfamily a, polypeptide 85, pseudogene
Defb16-ps	pseudo	defensin beta 16 pseudogene
Dnmt3a-ps2	pseudo	pink-eyed dilution-like
Dpp3l	pseudo	dipeptidylpeptidase 3-like
Dzip1-ps1	pseudo	DAZ interacting protein 1, pseudogene 1
E230034O05Rik	nc	E230034O05Rik gene
Fgfr1-ps1	pseudo	fibroblast growth factor receptor 1, pseudogene 1
Fsip2-ps1	pseudo	fibrous sheath-interacting protein 2, pseudogene 1
Gapdh-ps1	pseudo	glyceraldehyde-3-phosphate dehydrogenase, pseudogene 1
Gas5	nc	growth arrest specific 5
H19	nc	H19, imprinted maternally expressed transcript (non-protein coding)
Hmgb1-ps2	pseudo	high mobility group box 1, pseudogene 2
Hmox2-ps1	pseudo	heme oxygenase (decycling) 2, pseudogene 1
Hoxa11-as	nc	similar to hypothetical gene supported by BC025338
Hspd1-ps2	pseudo	heat shock protein 1, pseudogene 2
Hspd1-ps3	pseudo	heat shock protein 1, pseudogene 3
Ifi203-ps1	pseudo	interferon activated gene 203, pseudogene 1
LOC100359554	pseudo	Cytochrome P450 2C26-like
LOC100361049	pseudo	hypothetical protein LOC100361049
LOC100362263	pseudo	similar to Heat shock transcription factor, Y-linked
LOC100363290	nc	hypothetical protein LOC100363290
LOC100363736	pseudo	ribosomal protein L19-like
LOC100364138	pseudo	ferritin light chain 1-like
LOC100364957	pseudo	RGD1560755 protein-like
LOC100365858	pseudo	zinc finger CCCH type, antiviral 1-like
LOC100910558	nc	uncharacterized LOC100910558
LOC100912673	pseudo	similar to High mobility group protein 1-like 10 (HMG-1L10)
LOC102547637	nc	uncharacterized LOC102547637
LOC102548134	nc	hypothetical protein LOC690347
LOC102550668	pseudo	60S ribosomal protein L21-like
LOC102551195	pseudo	60S ribosomal protein L5-like
LOC102552129	pseudo	similar to Tubulin alpha-2 chain (Alpha-tubulin 2)
LOC102552394	pseudo	Nucleoside 2-deoxyribosyltransferase domain containing protein RGD620382
LOC102552870	pseudo	60S ribosomal protein L23a-like
LOC102554402	pseudo	similar to High mobility group protein 1 (HMG-1) (Heparin-binding protein p30)
LOC102554715	pseudo	similar to RIKEN cDNA 5031410I06
LOC102554992	pseudo	60S ribosomal protein L29-like
LOC102555366	pseudo	similar to Cofilin, non-muscle isoform (Cofilin-1)
LOC102556209	nc	uncharacterized LOC102556209
LOC103690331	pseudo	similar to ribosomal protein L13
LOC103690592	nc	hypothetical protein LOC685569
LOC103690796	pseudo	60S ribosomal protein L9-like
LOC103691298	pseudo	similar to lysophospholipase I
LOC103691301	pseudo	similar to olfactory receptor 1463
LOC103691425	pseudo	60S ribosomal protein L7a pseudogene
LOC103691563	pseudo	similar to ribosomal protein L31
LOC103691840	pseudo	similar to DNA polymerase epsilon subunit 4
LOC103692072	pseudo	similar to mKIAA2005 protein
LOC103692519	pseudo	60S ribosomal protein L9 pseudogene
LOC103693457	pseudo	similar to ATPase, H+ transporting, V1 subunit F
LOC103694291	pseudo	similar to small nuclear ribonucleoprotein D3

Table S8 continued

symbol	classif.[1]	GeneName
LOC287004	nc	Mg1 protein
LOC301725	pseudo	similar to 60S ribosomal protein L35
LOC301772	pseudo	similar to Y-linked testis-specific protein
LOC301772	pseudo	similar to Y-linked testis-specific protein
LOC303341	pseudo	hypothetical LOC303341
LOC303590	pseudo	similar to cactin CG1676-PA
LOC304027	pseudo	similar to NACHT, leucine rich repeat and PYD containing 4A
LOC310487	pseudo	similar to purinergic receptor P2Y, G-protein coupled, 4
LOC361963	pseudo	similar to phosphoglycerate mutase (EC 5.4.2.1) B chain - rat
LOC362473	pseudo	similar to Elongation factor 1-gamma (EF-1-gamma) (eEF-1B gamma)
LOC365444	pseudo	similar to CGI-09 protein
LOC366449	pseudo	hypothetical LOC366449
LOC366632	pseudo	similar to 40S ribosomal protein S6
LOC366709	pseudo	similar to prohibitin
LOC366979	pseudo	hypothetical LOC366979
LOC367050	pseudo	similar to 60S ribosomal protein L35
LOC497848	pseudo	hypothetical LOC497848
LOC497952	pseudo	similar to Ubiquitin-associated protein 2-like
LOC498601	nc	similar to cyclin B2
LOC499023	pseudo	similar to keratin complex 1, acidic, gene 18
LOC500213	pseudo	similar to T-box transcription factor TBX15 (T-box protein 15) (MmTBx8)
LOC500265	pseudo	nucleoporin 50-like
LOC501810	pseudo	similar to Leukosialin precursor (Sialophorin, Ly-48) (B cell different. antigen LP-3, CD43)
LOC502504	nc	similar to CG11883-PB, isoform B
LOC502876	pseudo	similar to protein phosphatase 1, regulatory subunit 15B
LOC679583	pseudo	similar to UPF0197 protein C11orf10 homolog
LOC679586	pseudo	similar to ATP synthase, H+ transporting, mitochondrial Fo complex, subunit G
LOC679899	pseudo	similar to 40S ribosomal protein S20
LOC680224	pseudo	similar to Aspartate aminotransferase, mitochondrial precursor (Transaminase A)
LOC680590	nc	hypothetical protein LOC680590
LOC680635	pseudo	similar to 40S ribosomal protein S10
LOC680802	pseudo	similar to Zinc finger protein 45 (BRC1744)
LOC680967	pseudo	RAB1A-like protein-like
LOC681958	pseudo	similar to Polycomb group RING finger protein 2 (DNA-binding Mel-18) (Zfp-144)
LOC683581	nc	similar to ADP-ribosylation factor 7
LOC683746	pseudo	similar to thyroid autoantigen
LOC684399	pseudo	similar to 60S ribosomal protein L29 (P23)
LOC685003	pseudo	similar to Leydig cell tumor 10 kDa protein
LOC685176	pseudo	similar to Spindlin-like protein 2 (SPIN-2)
LOC685262	pseudo	similar to paired immunoglobin-like type 2 receptor beta
LOC686096	pseudo	hypothetical protein LOC686096
LOC686151	pseudo	similar to cell division cycle associated 5
LOC687746	pseudo	similar to Mitochondrial import inner membrane translocase subunit Tim17-B
LOC688539	pseudo	similar to Fructose-bisphosphate aldolase A (Muscle-type aldolase)
LOC688562	pseudo	similar to U1 small nuclear ribonucleoprotein C (U1 snRNP protein C) (U1C protein) (U1-C)
LOC688570	pseudo	similar to butyrate-induced transcript 1
LOC688874	pseudo	similar to NEDD4-binding protein 1 (N4BP1)
LOC688948	pseudo	similar to ribosomal protein S26
LOC689316	nc	hypothetical protein LOC689316
LOC689435	pseudo	similar to vomeronasal 2, receptor, 1
LOC689459	pseudo	similar to Cytochrome c, somatic
LOC689961	pseudo	similar to ATP synthase lipid-binding protein, mitochondrial precursor
LOC690120	nc	hypothetical protein LOC690120
LOC690138	pseudo	similar to UPF0197 protein C11orf10 homolog
LOC690269	pseudo	hypothetical protein LOC690269
LOC690350	pseudo	similar to LSM7 homolog, U6 small nuclear RNA associated
LOC690840	pseudo	similar to ribosomal protein L37
LOC691797	pseudo	similar to developmental pluripotency-associated 3
Nutf2-ps1	pseudo	similar to Nuclear transport factor 2 (NTF-2)
Olr1276-ps	pseudo	olfactory receptor Olfr936-like
Olr1740-ps	pseudo	olfactory receptor 10-like

Table S8 continued

symbol	classif.[1]	GeneName
Olr366-ps	pseudo	thioredoxin-like 4-like
Pcdhgb2	pseudo	protocadherin gamma subfamily B, 2
Phb-ps1	pseudo	similar to prohibitin
Pira2	nc	paired-Ig-like receptor A2
pramef20l	pseudo	similar to PRAME family member 8
Prrc2c	pseudo	proline-rich coiled-coil 2C
Rexo1l1-ps1	pseudo,cod.	similar to Transcription elongation factor B polypeptide 3 binding protein 1
Rf11	pseudo	similar to RING finger protein 11 (NEDD4 WW domain-binding protein 2) (Sid 1669)
RGD1306704	nc	hypothetical LOC295483
RGD1559513	pseudo	similar to DEAD (Asp-Glu-Ala-Asp) box polypeptide 41
RGD1559677	pseudo	similar to cell surface receptor FDFACT
RGD1559743	pseudo	similar to 40S ribosomal protein S16
RGD1559747	pseudo	similar to Zinc finger and SCAN domain containing protein 2 (Zinc finger protein 29)
RGD1559772	pseudo	similar to Luc7 homolog (S. cerevisiae)-like
RGD1559859	nc	RGD1559859
RGD1559892	pseudo	ribosomal protein L29 pseudogene
RGD1560208	pseudo	similar to Farnesyl pyrophosphate synthetase (FPP synthetase)
RGD1560350	pseudo	similar to proteasome subunit iota
RGD1560412	pseudo	similar to Leukosialin precursor (Leucocyte sialoglycoprotein)
RGD1560648	pseudo	similar to DJ-1 protein
RGD1560789	pseudo	similar to ribosomal protein S2
RGD1560815	pseudo	similar to acidic ribosomal phosphoprotein P1
RGD1560936	pseudo	similar to 60S ribosomal protein L13
RGD1561232	pseudo	keratin 8-like
RGD1561381	pseudo	similar to microsomal glutathione S-transferase 3
RGD1561582	pseudo	similar to Fus1 protein
RGD1561620	pseudo	similar to isopentenyl diphosphate delta-isomerase type 2
RGD1561627	pseudo	similar to hypothetical protein 4930474N05
RGD1561766	pseudo	similar to basic transcription factor 3
RGD1561843	pseudo	ribosomal protein L23a pseudogene
RGD1561890	pseudo	similar to craniofacial development protein 1
RGD1562143	pseudo	similar to Ctps protein
RGD1562146	nc	RGD1562146
RGD1562404	pseudo	similar to ribosomal protein S18
RGD1562542	pseudo	similar to ribosomal protein S12
RGD1562890	nc	RGD1562890
RGD1562937	pseudo	similar to ribosomal protein S24
RGD1563049	nc	RGD1563049
RGD1563100	pseudo	similar to KIAA0089
RGD1563375	pseudo	similar to small nuclear ribonucleoparticle-associated protein
RGD1563725	pseudo	similar to C1GALT1-specific chaperone 1
RGD1564031	pseudo	similar to transcription elongation factor B (SIII), polypeptide 2
RGD1564167	pseudo	similar to basic transcription factor 3
RGD1564268	pseudo	similar to ribosomal protein L36
RGD1564386	pseudo	similar to TDPOZ3
RGD1564482	nc	RGD1564482
RGD1564534	nc	similar to CHCHD4 protein
RGD1564613	pseudo	similar to MGC40405 protein
RGD1564814	pseudo	similar to CDNA sequence BC061212
RGD1565054	pseudo	similar to 60S acidic ribosomal protein P1
RGD1565183	pseudo	similar to ribosomal protein L28
RGD1565495	pseudo	similar to retinoblastoma binding protein 7
RGD1565534	pseudo	similar to RING finger protein 33
RGD1565648	pseudo	similar to Chain A, Solution Structure Of Rabbit Apo-S100a11 (19 Models)
RGD1565661	pseudo	similar to RIKEN cDNA 3110001I22
RGD1565689	nc	RGD1565689
RGD1565900	pseudo	similar to ribosomal protein L27
RGD1566033	pseudo	similar to BC003940 protein
RGD1566136	pseudo	similar to 40S ribosomal protein S9
RGD1566197	pseudo	similar to prohibitin
RGD1566247	pseudo	similar to 40S ribosomal protein S2

Table S8 continued

symbol	classif.[1]	GeneName
RGD1566355	pseudo	similar to cell division cycle 2-like 1
Rhno1	nc	similar to 5930416I19Rik protein
Rpl27-ps1	pseudo	ribosomal protein L27, pseudogene 1
Rpl31l1	pseudo	similar to ribosomal protein L31
Rpl34l1	pseudo	ribosomal protein L34-like1
Rpl5l1	pseudo	ribosomal protein L5-like 1
Rpph1	nc	RNA component of mitochondrial RNA processing endoribonuclease
Rps15al1	pseudo	ribosomal protein S15A-like 1
Rps15-ps2	pseudo	ribosomal protein S15, pseudogene 2
Rps17l	pseudo	ribosomal protein S17-like
Rps27a-ps5	pseudo	similar to putative protein kinase
Rps2-ps7	pseudo	ribosomal protein S2, pseudogene 7
Rrm1-ps1	pseudo	ribonucleotide reductase M1, pseudogene 1
Sdccag1-ps1	pseudo	serologically defined colon cancer antigen 1
Sec61gl	pseudo	SEC61 gamma subunit-like
Snrpgl2	pseudo	small nuclear ribonucleoprotein polypeptide G-like 2
Terc	nc	telomerase RNA component
Tnxa-ps1	pseudo	tenascin XA, pseudogene 1
Ubbp4	pseudo	similar to ribosomal protein S27a
Vhll	pseudo	similar to von Hippel-Lindau syndrome protein homolog
Vof16	nc	ischemia related factor vof-16
Voom1r-ps20	pseudo	similar to vomeronasal 1 receptor, f2
Zbtb8os	os,coding	zinc finger and BTB domain containing 8 opposite strand, pseudogene 1
Znrd1as	as,coding	hypothetical protein LOC685722

1 as: antisense, nc: noncoding, os: opposite strand, pseudo: pseudogene, red: coding

Table S9

Distinct recovery of noncoding RNA in endothelial cells, fibroblasts and AS-Tspan8-TEX

Table S9A

Distinct recovery of noncoding RNA in AS-Tspan8-TEX versus endothelial cells and fibroblasts

**symbol**	**classif.**	**EC**	**Fb**	**AS-Tspan8-TEX**
Gas5	nc	37122	68794	**21573**
RGD1564482	nc	1031	714	**523**
Terc	nc	158	190	**714**
LOC689316	nc	70	62	**570**
RGD1559859	nc	184	147	**302**
Atp5hl1	pseudo	28329	38699	**48988**
Cfl1	pseudo	11037	9216	**5596**
Cox6c-ps1	pseudo	2817	4240	**4815**
Gapdh-ps1	pseudo	1585	1342	**11323**
Hspd1-ps2	pseudo	7913	6339	**16848**
Hspd1-ps3	pseudo	1428	1097	**3074**
LOC100362689	pseudo	969	1783	**367**
LOC102552129	pseudo	46021	42055	**83265**
LOC102552394	pseudo	2272	1574	**5149**
LOC102555366	pseudo	440	413	**237**
LOC103690331	pseudo	260	294	**136**
LOC103691563	pseudo	3769	4153	**2498**
LOC103692072	pseudo	1046	2798	**1333**
LOC103693457	pseudo	3902	4012	**7049**
LOC361963	pseudo	12503	4012	**14122**
LOC362473	pseudo	76332	70240	**33927**
LOC365444	pseudo	553	501	**880**
LOC366709	pseudo	4513	4451	**11116**
LOC497952	pseudo	288	300	**177**
LOC679583	pseudo	10735	15076	**5773**
LOC680802	pseudo	304	93	**982**
LOC687746	pseudo	639	600	**1121**
LOC688539	pseudo	3397	2048	**6302**
LOC689459	pseudo	355	251	**2338**
LOC689961	pseudo	15500	15608	**35372**
LOC690138	pseudo	5008	7033	**3169**
Phb-ps1	pseudo	1859	1924	**4199**
Prrc2c	pseudo	843	1722	**993**
RGD1559513	pseudo	719	776	**2165**
RGD1559892	pseudo	4068	4012	**2896**
RGD1560648	pseudo	5043	3984	**8935**
RGD1561381	pseudo	1607	2062	**212**
RGD1561843	pseudo	2876	2557	**1403**
RGD1562143	pseudo	2034	1722	**553**
RGD1562404	pseudo	215899	176585	**19409**
RGD1562937	pseudo	23657	28329	**14978**
RGD1564613	pseudo	1389	2006	**946**
RGD1565183	pseudo	26249	27746	**45864**
RGD1565495	pseudo	1992	1596	**3214**
RGD1566033	pseudo	501	300	**82**
RGD1566197	pseudo	1168	1075	**293**
RGD1566355	pseudo	1499	1585	**166**
Rps27a-ps5	pseudo	125	91	**20976**
Rrm1-ps1	pseudo	1218	729	**2006**
Sec61gl	pseudo	22537	47315	**24154**
Tnxa-ps1	pseudo	690	613	**210**
Vhll	pseudo	66	128	**226**

Table S9B

Distinct recovery of noncoding RNA in endothelial cells versus fibroblasts

**symbol**	**classif.**	**EC**	**Fb**
Gas5	nc	37122	**68794**
LOC690120	nc	254	**556**
Rpph1	nc	1243	**2574**
Vof16	nc	190	**744**
LOC100362689	pseudo	969	**1783**
LOC103692072	pseudo	1046	**2798**
LOC361963	pseudo	**12503**	4012
LOC688539	pseudo	**3397**	2048
Pcdhgb2	pseudo	**452**	146
Prrc2c	pseudo	843	**1722**
Rrm1-ps1	pseudo	**1218**	729
Sec61gl	pseudo	22537	**47315**

Table S9C

Distinct recovery of noncoding RNA in AS-TEX treated endothelial cells or fibroblasts

**symbol**	**classif.**	**EC**	**EC+AS-TEX**	**Fb**	**Fb+AS-TEX**
RGD1562890	nc	180	215	380	**174**
Vof16	nc	190	258	744	**478**
LOC103692072	pseudo	1046	1448	2798	**1859**
LOC303590	pseudo	488	**209**	174	228
LOC680802	pseudo	304	**101**	93	97
LOC688562	pseudo	3373	3984	4673	**7033**
LOC689459	pseudo	355	292	251	**383**
Prrc2c	pseudo	843	**1296**	1722	2034
Tnxa-ps1	pseudo	690	**1361**	613	468
Vhll	pseudo	66	68	128	**77**

Table S9D

Distinct recovery of noncoding RNA in AS-Tspan8-TEX treated endothelial cells or fibroblasts

**symbol**	**classif.**	**EC**	**EC+AS-Tspan8-TEX**	**Fb**	**Fb+AS-Tspan8-TEX**
Gas5	nc	37122	37516	68794	**161369**
Hoxa11-as	nc	545	**1479**	600	690
RGD1564482	nc	1031	939	714	**1499**
Rpph1	nc	1243	**4405**	2574	2876
Terc	nc	158	**346**	190	108
Vof16	nc	190	**285**	744	**338**
Cfl1	pseudo	11037	9316	9216	**5833**
Cox6c-ps1	pseudo	2817	3170	4240	**2288**
Hspd1-ps2	pseudo	7913	6608	6339	**9878**
Hspd1-ps3	pseudo	1428	1091	1097	**1734**
LOC100362689	pseudo	969	885	1783	**3397**
LOC100363736	pseudo	30786	29559	34877	**53232**
LOC102552129	pseudo	46021	51959	42055	**25355**
LOC102552394	pseudo	2272	3019	1574	**2740**
LOC102552870	pseudo	15076	9299	13401	**26432**
LOC103690796	pseudo	6654	4755	4905	**7697**
LOC103691563	pseudo	3769	3373	4153	**8023**
LOC103693457	pseudo	3902	4406	4012	**6841**
LOC301725	pseudo	6747	6252	7033	**14766**
LOC365444	pseudo	553	**365**	501	**855**
LOC366632	pseudo	5955	4330	5113	**11113**
LOC366709	pseudo	4513	5918	4451	**2684**
LOC367050	pseudo	3040	**1710**	3641	**6562**
LOC680635	pseudo	3373	4168	2778	**7332**
Pcdhgb2	pseudo	452	**248**	146	131
Phb-ps1	pseudo	1859	**2724**	1924	1380
RGD1559892	pseudo	4068	3566	4012	**8023**
RGD1560208	pseudo	3517	3460	3169	**1629**
RGD1561381	pseudo	1607	**2445**	2062	**294**
RGD1561766	pseudo	2062	**1400**	1585	2091
RGD1561843	pseudo	2876	2120	2557	**4939**
RGD1562143	pseudo	2034	**3248**	1722	**1003**
RGD1564268	pseudo	3214	3972	3444	**6208**
RGD1564613	pseudo	1389	1747	2006	**1314**
RGD1565183	pseudo	26249	24920	27746	**42939**
RGD1566033	pseudo	501	497	300	**750**
Rrm1-ps1	pseudo	1218	1112	729	**362**
Sec61gl	pseudo	22537	**41136**	47315	46988
Tnxa-ps1	pseudo	690	1205	613	**347**
Ubbp4	pseudo	25355	20383	24154	**42055**

Table S9E

Annotation of noncoding RNA with altered expression in TEX-treated endothelial cells

upregulated by AS-TEX	
Prrc2c (ps)	possibly engaged in histone gene regulation regulation of the oncogenic splice factor SRSF1 (serine/arginine-rich splicing factor 1) in lung cancer and ribonucleoprotein complex formation (1,2)
Tnxa-ps1 (nc)	possibly engaged in ischemic shock (glucose metabolism), might act as a competing endogenous RNA to affect dual specificity phosphatase 1 (Dusp1) expression, regulating Schwann cell migration (3,4)
upregulated by AS-Tspan8-TEX	
Hoxa11-as (nc)	regulation of transcription by RNA polymerase II (5-22)
Phb-ps1 (ps)	upregulated in esphageal squamous cell carcinoma, forms RNA-RNA hybrids with PHB (prohibitin) increasing PHB expression at the mRNA and protein level with accelerated G1-G0 progression (23)
RGD1561381 (ps)	nuclear function in preconditioning neuroprotection in the neonate (24)
RGD1562143 (ps)	unknown
Rpph1 (nc)	aggravates hypoxia-induced tissue damage by targeting miR-206 and upregulation of ATG3 (autophagy related 3); downstream signaling may involve the PI3K/Akt/mTOR pathway. Overexpressed in early stage of Alzheimer, binds miR326-3p/miR-330-5p with release of Cdc42 (cell division cycle 42) and increased dendritic spine density. Targets miR-122 with release of ADAM10, PKM (pyruvate kinase M), NOD2 (nucleotide binding oligomerization domain containing 2) and IGF1R, promoting breast cancer progression (25-27)
Sec61gl (ps)	unknown
Terc (nc)	Terc regulates telomere length and is engaged in gene transcription; associated with progression of cervical intraepithelial to invasive cancer (28-32)
Vof16 (nc)	ischemia related factor, involved in attention deficit/hyperactivity symptoms (33)
downregulated by AS-TEX	
LOC303590 (ps)	unknown
downregulated by AS-Tspan8-TEX	
LOC102552870 (ps)	unknown
LOC365444 (ps)	similar to TRMT6 (tRNA methyltransferase 6), which accounts for N-methyladenosine epitranscriptomic regulation (34)
LOC367050 (ps)	unknown
Pcdhgb2 (ps)	unknown
RGD1561766 (ps)	similar to transcription factor 3 that activates transcription by binding regulatory E-box sequences (35)

Table S9F

Annotation of noncoding RNA with altered expression in TEX-treated fibroblasts

upregulated by AS-TEX	
LOC688562 (ps)	the U1 small nuclear RNA is one of the most abundant ncRNA, which is engaged in pre-messenger RNA splicing, it may contribute to gastric cancer progression via its downstream effector NGF (nerve growth factor) (36,37)
LOC689459 (ps)	unknown
upregulated by AS-Tspan8-TEX	
Gas5 (nc)	Gas5 is a tumor suppressor that mostly acts by miRNA sponging affecting multiple signaling pathways; it also is invovled in innate immune cell regulation and angiogenesis (38-54)
Hspd1-ps2 (ps)	may be engaged in impaired stress response; connects several cellular networks including ribosome biogenesis (55)
Hspd1-ps3 (ps)	see ref. (55)
LOC100362689 (ps)	unknown
LOC100363736 (ps)	unknown
LOC102552394 (ps)	unknown
LOC102552870 (ps)	unknown
LOC103690796 (ps)	unknown
LOC103691563 (ps)	unknown
LOC103693457 (ps)	V-type proton ATPase subunit F pseudogene, may be engaged in protein export (56)
LOC301725 (ps)	unknown
LOC365444 (ps)	unknown
LOC366632 (ps)	unknown
LOC367050 (ps)	unknown
LOC680635 (ps)	unknown
RGD1559892 (ps)	unknown
RGD1561843 (ps)	unknown
RGD1564268 (ps)	may be engaged in a cell cycle regulatory network comprising nuclear lncRNAPLAC2/ STAT1 binding to the RPL36 promoter; however cytosolic PLAC2 bound STAT1 becomes hampered in nuclear transfer (57)

RGD1564482 (nc)	unknown
RGD1565183 (ps)	similar to ribosomal protein L28 (cytosolic large ribosomal subunit) (58,59)
RGD1566033 (ps)	unknown
Ubbp4 (ps)	Belongs to the E3 ubiquitin ligase family, oncogenic, multiple activities,engaged in protein complex formation (60-62)
downregulated by AS-TEX	
LOC103692072 (ps)	unknown
RGD1562890 (nc)	unknown
Vhll (ps)	predisposes to develop tumors in specific organs, including the pancreas by triggering tranformation, metabolism imbalance and ECM remodelling. May be engaged in pluripotency maintenance, associated with increased VEGF, PDGFβ, TGFα, erythropoietin and glucose metabolism via increased HIF1α expression and stabilization (63-66)
Vof16 (nc)	see ref. (33)
downregulated by AS-Tspan-TEX	
Cfl1 (LOC103694804) (ps)	Similar to cofilin 1. High cofilin 1 expression correlates with non-small cell lung cancer patients survival and low Twist1 expression; in triple negative breast cancer miR-200b-3p and miR-429-5p arrest the cell cycle by suppressing cyclin dependent kinases; in EC, it contributes to actin remodeling and migration upon VEGF stimulation and account for the actin cortex thickness and tension that inversely correlates with cell cycle progression; it also is invovled in nuclear integrity, transcriptional regulation, apoptosis and lipid metabolism (67-72)
Cox6c-ps1 (ps)	unknown
LOC102552129 (ps)	unknown
LOC366709 (ps)	unknown
Phb-ps1 (ps)	see ref. (23)
RGD1560208 (ps)	unknown
RGD1561381 (ps)	similar to MGST3 (microsomal glutathione-S-transferase 3); engaged in oxidative stress protection; mutations might be associated with inflammation and risk of developing esophageal cancer (73,74)
RGD1562143 (ps)	unknown
RGD1564613 (ps)	unknown
Rrm1-ps1 (ps)	unknown
Terc (nc)	see ref. (28-32)
Tnxa-ps1 (ps)	see ref. (3,4)

References

1. de Miguel FJ, Sharma RD, Pajares MJ, Montuenga LM, Rubio A, Pio R. Identification of alternative splicing events regulated by the oncogenic factor SRSF1 in lung cancer. Cancer Res. 2014 Feb 15;74(4):1105-15. doi: 10.1158/0008-5472.CAN-13-1481
2. Youn JY, Dunham WH, Hong SJ, Knight JDR, Bashkurov M, Chen GI, Bagci H, Rathod B, MacLeod G, Eng SWM, Angers S, Morris Q, Fabian M, Côté JF, Gingras AC. High-Density Proximity Mapping Reveals the Subcellular Organization of mRNA-Associated Granules and Bodies. Mol Cell. 2018 Feb 1;69(3):517-532.e11. doi: 10.1016/j.molcel.2017.12.020.
3. Yao C, Wang Y, Zhang H, Feng W, Wang Q, Shen D, Qian T, Liu F, Mao S, Gu X, Yu B. lncRNA TNXA-PS1 Modulates Schwann Cells by Functioning As a Competing Endogenous RNA Following Nerve Injury. J Neurosci. 2018 Jul 18;38(29):6574-6585. doi: 10.1523/JNEUROSCI.3790-16.2018.
4. Li H, Wu Y, Suo G, Shen F, Zhen Y, Chen X, Lv H. Profiling neuron-autonomous lncRNA changes upon ischemia/reperfusion injury. Biochem Biophys Res Commun. 2018 Jan 1;495(1):104-109. doi: 10.1016/j.bbrc.2017.10.157.
5. Liu Z, Chen Z, Fan R, Jiang B, Chen X, Chen Q, Nie F, Lu K, Sun M. Over-expressed long noncoding RNA HOXA11-AS promotes cell cycle progression and metastasis in gastric cancer. Mol Cancer. 2017 Apr 26;16(1):82. doi: 10.1186/s12943-017-0651-6.
6. Li W, Jia G, Qu Y, Du Q, Liu B, Liu B. Long Non-Coding RNA (LncRNA) HOXA11-AS Promotes Breast Cancer Invasion and Metastasis by Regulating Epithelial-Mesenchymal Transition. Med Sci Monit. 2017 Jul 13;23:3393-3403.
7. Xu C, He T, Li Z, Liu H, Ding B. Regulation of HOXA11-AS/miR-214-3p/EZH2 axis on the growth, migration and invasion of glioma cells. Biomed Pharmacother. 2017 Nov;95:1504-1513. doi: 10.1016/j.biopha.2017.08.097.
8. Sun M, Nie F, Wang Y, Zhang Z, Hou J, He D, Xie M, Xu L, De W, Wang Z, Wang J. LncRNA HOXA11-AS Promotes Proliferation and Invasion of Gastric Cancer by Scaffolding the Chromatin Modification Factors PRC2, LSD1, and DNMT1. Cancer Res. 2016 Nov 1;76(21):6299-6310.
9. Lu Q, Zhao N, Zha G, Wang H, Tong Q, Xin S. LncRNA HOXA11-AS Exerts Oncogenic Functions by Repressing p21 and miR-124 in Uveal Melanoma. DNA Cell Biol. 2017 Oct;36(10):837-844. doi: 10.1089/dna.2017.3808.
10. Yu J, Hong JF, Kang J, Liao LH, Li CD. Promotion of LncRNA HOXA11-AS on the proliferation of hepatocellular carcinoma by regulating the expression of LATS1. Eur Rev Med Pharmacol Sci. 2017 Aug;21(15):3402-3411.
11. Chen D, Sun Q, Zhang L, Zhou X, Cheng X, Zhou D, Ye F, Lin J, Wang W. The lncRNA HOXA11-AS functions as a competing endogenous RNA to regulate PADI2 expression by sponging miR-125a-5p in liver metastasis of colorectal cancer. Oncotarget. 2017 Aug 3;8(41):70642-70652. doi: 10.18632/oncotarget.19956.
12. Cui Y, Yi L, Zhao JZ, Jiang YG. Long Noncoding RNA HOXA11-AS Functions as miRNA Sponge to Promote the Glioma Tumorigenesis Through Targeting miR-140-5p. DNA Cell Biol. 2017 Oct;36(10):822-828. doi: 10.1089/dna.2017.3805.
13. Cui M, Wang J, Li Q, Zhang J, Jia J, Zhan X. Long non-coding RNA HOXA11-AS functions as a competing endogenous RNA to regulate ROCK1 expression by sponging miR-124-3p in osteosarcoma. Biomed Pharmacother. 2017 Aug;92:437-444. doi: 10.1016/j.biopha.2017.05.081.
14. Chen JH, Zhou LY, Xu S, Zheng YL, Wan YF, Hu CP. Overexpression of lncRNA HOXA11-AS promotes cell epithelial-mesenchymal transition by repressing miR-200b in non-small cell lung cancer. Cancer Cell Int. 2017 Jun 12;17:64. doi: 10.1186/s12935-017-0433-7.

15. Yu W, Peng W, Jiang H, Sha H, Li J. LncRNA HOXA11-AS promotes proliferation and invasion by targeting miR-124 in human non-small cell lung cancer cells. Tumour Biol. 2017 Oct;39(10):1010428317721440. doi: 10.1177/1010428317721440.
16. Zhan M, He K, Xiao J, Liu F, Wang H, Xia Z, Duan X, Huang R, Li Y, He X, Yin H, Xiang G, Lu L. LncRNA HOXA11-AS promotes hepatocellular carcinoma progression by repressing miR-214-3p. J Cell Mol Med. 2018 May 15. doi: 10.1111/jcmm.13633. [Epub ahead of print]
17. Yang FQ, Zhang JQ, Jin JJ, Yang CY, Zhang WJ, Zhang HM, Zheng JH, Weng ZM. HOXA11-AS promotes the growth and invasion of renal cancer by sponging miR-146b-5p to upregulate MMP16 expression. J Cell Physiol. 2018 Dec;233(12):9611-9619. doi: 10.1002/jcp.26864.
18. Zhang Y, He RQ, Dang YW, Zhang XL, Wang X, Huang SN, Huang WT, Jiang MT, Gan XN, Xie Y, Li P, Luo DZ, Chen G, Gan TQ. Comprehensive analysis of the long noncoding RNA HOXA11-AS gene interaction regulatory network in NSCLC cells. Cancer Cell Int. 2016 Dec 1;16:89. doi: 10.1186/s12935-016-0366-6.
19. Li B, Wang W, Miao S, Li G, Lv Y, Xiang C, Pei R. HOXA11-AS promotes the progression of oral squamous cell carcinoma by targeting the miR-518a-3p/PDK1 axis. Cancer Cell Int. 2019 May 21;19:140. doi: 10.1186/s12935-019-0838-6.
20. Xu CH, Xiao LM, Liu Y, Chen LK, Zheng SY, Zeng EM, Li DH. The lncRNA HOXA11-AS promotes glioma cell growth and metastasis by targeting miR-130a-5p/HMGB2. Eur Rev Med Pharmacol Sci. 2019 Jan;23(1):241-252. doi: 10.26355/eurrev_201901_16770.
21. Liu Y, Zhang YM, Ma FB, Pan SR, Liu BZ. Long noncoding RNA HOXA11-AS promotes gastric cancer cell proliferation and invasion *via* SRSF1 and functions as a biomarker in gastric cancer. World J Gastroenterol. 2019 Jun 14;25(22):2763-2775. doi: 10.3748/wjg.v25.i22.2763.
22. Wang S, Zhang S, He Y, Huang X, Hui Y, Tang Y. HOXA11-AS regulates JAK-STAT pathway by miR-15a-3p/STAT3 axis to promote the growth and metastasis in liver cancer. J Cell Biochem. 2019 Sep;120(9):15941-15951. doi: 10.1002/jcb.28871.
23. Feng F, Qiu B, Zang R, Song P, Gao S. Pseudogene PHBP1 promotes esophageal squamous cell carcinoma proliferation by increasing its cognate gene PHB expression. Oncotarget. 2017 Apr 25;8(17):29091-29100. doi: 10.18632/oncotarget.16196.
24. Kimberly EM Cox-Limpens, Johan SH Vles, Daniel LA van den Hove, Luc JI Zimmermann, Antonio WD Gavilanes. Fetal asphyctic preconditioning alters the transcriptional response to perinatal asphyxia. BMC Neurosci. 2014; 15: 67. doi: 10.1186/1471-2202-15-67
25. Kong F, Jin J, Lv X, Han Y, Liang X, Gao Y, Duan X. Long noncoding RNA RMRP upregulation aggravates myocardial ischemia-reperfusion injury by sponging miR-206 to target ATG3 expression. Biomed Pharmacother. 2019 Jan;109:716-725. doi: 10.1016/j.biopha.2018.10.079.
26. Cai Y, Sun Z, Jia H, Luo H, Ye X, Wu Q, Xiong Y, Zhang W, Wan J. *Rpph1* Upregulates CDC42 Expression and Promotes Hippocampal Neuron Dendritic Spine Formation by Competing with miR-330-5p. Front Mol Neurosci. 2017 Feb 7;10:27. doi: 10.3389/fnmol.2017.00027.
27. Zhang Y, Tang L. Inhibition of breast cancer cell proliferation and tumorigenesis by long non-coding RNA *RPPH1* down-regulation of miR-122 expression. Cancer Cell Int. 2017 Nov 21;17:109. doi: 10.1186/s12935-017-0480-0.
28. Zhou J, Ding D, Wang M, Cong YS. Telomerase reverse transcriptase in the regulation of gene expression. BMB Rep. 2014 Jan;47(1):8-14.
29. Majumder M, House R, Palanisamy N, Qie S, Day TA, Neskey D, Diehl JA, Palanisamy V. RNA-Binding Protein FXR1 Regulates p21 and TERC RNA to Bypass p53-Mediated Cellular Senescence in OSCC. PLoS Genet. 2016 Sep 8;12(9):e1006306. doi: 10.1371/journal.pgen.1006306.
30. Laudadio I, Orso F, Azzalin G, Calabrò C, Berardinelli F, Coluzzi E, Gioiosa S, Taverna D, Sgura A, Carissimi C, Fulci V. AGO2 promotes telomerase activity and interaction between the telomerase components TERT and TERC. EMBO Rep. 2019 Feb;20(2). pii: e45969. doi: 10.15252/embr.201845969
31. Chu C, Qu K, Zhong FL, Artandi SE, Chang HY. Genomic maps of long noncoding RNA occupancy reveal principles of RNA-chromatin interactions. Mol Cell. 2011 Nov 18;44(4):667-78. doi: 10.1016/j.molcel.2011.08.027.
32. Fish L, Zhang S, Yu JX, Culbertson B, Zhou AY, Goga A, Goodarzi H. Cancer cells exploit an orphan RNA to drive metastatic progression. Nat Med. 2018 Nov;24(11):1743-1751. doi: 10.1038/s41591-018-0230-4.
33. Dela Peña I, Bang M, Lee J, de la Peña JB, Kim BN, Han DH, Noh M, Shin CY, Cheong JH. Common prefrontal cortical gene expression profiles between adolescent SHR/NCrl and WKY/NCrl rats which showed inattention behavior. Behav Brain Res. 2015 Sep 15;291:268-276. doi: 10.1016/j.bbr.2015.05.012.
34. Li X, Xiong X, Zhang M, Wang K, Chen Y, Zhou J, Mao Y, Lv J, Yi D, Chen XW, Wang C, Qian SB, Yi C. Base-Resolution Mapping Reveals Distinct m_1A Methylome in Nuclear- and Mitochondrial-Encoded Transcripts. Mol Cell. 2017 Dec 7;68(5):993-1005.e9. doi: 10.1016/j.molcel.2017.10.019.
35. Moorman AV. New and emerging prognostic and predictive genetic biomarkers in B-cell precursor acute lymphoblastic leukemia. Haematologica. 2016 Apr;101(4):407-16. doi: 10.3324/haematol.2015.141101.
36. Guiro J, O'Reilly D. Insights into the U1 small nuclear ribonucleoprotein complex superfamily. Wiley Interdiscip Rev RNA. 2015 Jan-Feb;6(1):79-92. doi: 10.1002/wrna.1257.
37. Dou N, Yang D, Yu S, Wu B, Gao Y, Li Y. SNRPA enhances tumour cell growth in gastric cancer through modulating NGF expression. Cell Prolif. 2018 Oct;51(5):e12484. doi: 10.1111/cpr.12484.
38. Li BG, Wu WJ, Zheng HC, Yang HF, Zuo YX, Cui XP. Long noncoding RNA GAS5 silencing inhibits the expression of KCNQ3 by sponging miR-135a-5p to prevent the progression of epilepsy. Kaohsiung J Med Sci. 2019 Aug 2. doi: 10.1002/kjm2.12102.
39. Ma C, Wang W, Li P. LncRNA GAS5 overexpression downregulates IL-18 and induces the apoptosis of fibroblast-like synoviocytes. Clin Rheumatol. 2019 Aug 1. doi: 10.1007/s10067-019-04691-2.
40. Zhang X, Du K, Lou Z, Ding K, Zhang F, Zhu J, Chang Z. The CtBP1-HDAC1/2-IRF1 transcriptional complex represses the expression of the long noncoding RNA GAS5 in human osteosarcoma cells. Int J Biol Sci. 2019 Jun 2;15(7):1460-1471. doi: 10.7150/ijbs.33529
41. Li B, Hu X, Yang Y, Zhu M, Zhang J, Wang Y, Pei X, Zhou H, Wu J. GAS5/miR-21 Axis as a Potential Target to Rescue ZCL-082-Induced Autophagy of Female Germline Stem Cells In Vitro. Mol Ther Nucleic Acids. 2019 Jun 28;17:436-447. doi: 10.1016/j.omtn.2019.06.012.
42. Guo LL, Wang SF. Downregulated Long Noncoding RNA GAS5 Fails to Function as Decoy of CEBPB, Resulting in Increased GDF15 Expression and Rapid Ovarian Cancer Cell Proliferation. Cancer Biother Radiopharm. 2019 Jul 16. doi: 10.1089/cbr.2019.2889.
43. Li J, Li L, Yuan H, Huang XW, Xiang T, Dai S. Up-regulated lncRNA GAS5 promotes chemosensitivity and apoptosis of triple-negative breast cancer cells. Cell Cycle. 2019 Aug;18(16):1965-1975. doi: 10.1080/15384101.2019.1635870.
44. Yang W, Xu X, Hong L, Wang Q, Huang J, Jiang L. Upregulation of lncRNA GAS5 inhibits the growth and metastasis of cervical cancer cells. J Cell Physiol. 2019 Jun 17. doi: 10.1002/jcp.28926.

45. Zhang F, Yang C, Xing Z, Liu P, Zhang B, Ma X, Huang L, Zhuang L. LncRNA GAS5-mediated miR-1323 promotes tumor progression by targeting TP53INP1 in hepatocellular carcinoma. Onco Targets Ther. 2019 May 21;12:4013-4023. doi: 10.2147/OTT.S209439.
46. Cheng Y, Dai X, Yang T, Zhang N, Liu Z, Jiang Y. Low Long Noncoding RNA Growth Arrest-Specific Transcript 5 Expression in the Exosomes of Lung Cancer Cells Promotes Tumor Angiogenesis. J Oncol. 2019 May 2;2019:2476175. doi: 10.1155/2019/2476175.
47. Dong S, Zhang X, Liu D. Overexpression of long noncoding RNA GAS5 suppresses tumorigenesis and development of gastric cancer by sponging miR-106a-5p through the Akt/mTOR pathway. Biol Open. 2019 Jun 26;8(6). pii: bio041343. doi: 10.1242/bio.041343.
48. Tang R, Mei X, Wang YC, Cui XB, Zhang G, Li W, Chen SY. LncRNA GAS5 regulates vascular smooth muscle cell cycle arrest and apoptosis via p53 pathway. Biochim Biophys Acta Mol Basis Dis. 2019 Sep 1;1865(9):2516-2525. doi: 10.1016/j.bbadis.2019.05.022.
49. Liu K, Liu C, Zhang Z. lncRNA GAS5 acts as a ceRNA for miR-21 in suppressing PDGF-bb-induced proliferation and migration in vascular smooth muscle cells. J Cell Biochem. 2019 Sep;120(9):15233-15240. doi: 10.1002/jcb.28789.
50. Yao J, Shi Z, Ma X, Xu D, Ming G. lncRNA GAS5/miR-223/NAMPT axis modulates the cell proliferation and senescence of endothelial progenitor cells through PI3K/AKT signaling. J Cell Biochem. 2019 Sep;120(9):14518-14530. doi: 10.1002/jcb.28713.
51. Song J, Shu H, Zhang L, Xiong J. Long noncoding RNA GAS5 inhibits angiogenesis and metastasis of colorectal cancer through the Wnt/β-catenin signaling pathway. J Cell Biochem. 2019 Jan 22. doi: 10.1002/jcb.27743.
52. Tao H, Zhang JG, Qin RH, Dai C, Shi P, Yang JJ, Deng ZY, Shi KH. LncRNA GAS5 controls cardiac fibroblast activation and fibrosis by targeting miR-21 via PTEN/MMP-2 signaling pathway. Toxicology. 2017 Jul 1;386:11-18. doi: 10.1016/j.tox.2017.05.007.
53. Pickard MR, Williams GT. Molecular and Cellular Mechanisms of Action of Tumour Suppressor GAS5 LncRNA. Genes (Basel). 2015 Jul 7;6(3):484-99. doi: 10.3390/genes6030484.
54. Goustin AS, Thepsuwan P, Kosir MA, Lipovich L. The Growth-Arrest-Specific (*GAS*)-5 Long Non-Coding RNA: A Fascinating lncRNA Widely Expressed in Cancers. Noncoding RNA. 2019 Sep 17;5(3). pii: E46. doi: 10.3390/ncrna5030046.
55. Amit M, Weisberg SJ, Nadler-Holly M, McCormack EA, Feldmesser E, Kaganovich D, Willison KR, Horovitz A. Equivalent mutations in the eight subunits of the chaperonin CCT produce dramatically different cellular and gene expression phenotypes. J Mol Biol. 2010 Aug 20;401(3):532-43. doi: 10.1016/j.jmb.2010.06.037.
56. Ibuki T, Imada K, Minamino T, Kato T, Miyata T, Namba K. Common architecture of the flagellar type III protein export apparatus and F- and V-type ATPases. Nat Struct Mol Biol. 2011 Mar;18(3):277-82. doi: 10.1038/nsmb.1977.
57. Hu YW, Kang CM, Zhao JJ, Nie Y, Zheng L, Li HX, Li X, Wang Q, Qiu YR. LncRNA PLAC2 down-regulates RPL36 expression and blocks cell cycle progression in glioma through a mechanism involving STAT1. J Cell Mol Med. 2018 Jan;22(1):497-510. doi: 10.1111/jcmm.13338.
58. Burke PS, Lium E, Lin CS, Wolgemuth DJ. Sequence and expression of a cDNA encoding the mouse homologue of the rat ribosomal protein L28. Gene. 1994 May 16;142(2):315-6.
59. Kim YJ, Ahn K, Gim JA, Oh MH, Han K, Kim HS. Gene structure variation in segmental duplication block C of human chromosome 7q 11.23 during primate evolution. Gene. 2015 Dec 1;573(2):285-95. doi: 10.1016/j.gene.2015.07.060.
60. Weon JL, Yang SW, Potts PR. Cytosolic Iron-Sulfur Assembly Is Evolutionarily Tuned by a Cancer-Amplified Ubiquitin Ligase. Mol Cell. 2018 Jan 4;69(1):113-125.e6. doi: 10.1016/j.molcel.2017.11.010.
61. Hua Z, Early MJ. Closing target trimming and CTTdocker programs for discovering hidden superfamily loci in genomes. PLoS One. 2019 Jul 2;14(7):e0209468. doi: 10.1371/journal.pone.0209468.
62. Zheng N, Shabek N. Ubiquitin Ligases: Structure, Function, and Regulation. Annu Rev Biochem. 2017 Jun 20;86:129-157. doi: 10.1146/annurev-biochem-060815-014922.
63. Tabaro F, Minervini G, Sundus F, Quaglia F, Leonardi E, Piovesan D, Tosatto SC. VHLdb: A database of von Hippel-Lindau protein interactors and mutations. Sci Rep. 2016 Aug 11;6:31128. doi: 10.1038/srep31128.
64. Minervini G, Quaglia F, Tabaro F, Tosatto SCE. Genotype-phenotype relations of the von Hippel-Lindau tumor suppressor inferred from a large-scale analysis of disease mutations and interactors. PLoS Comput Biol. 2019 Apr 3;15(4):e1006478. doi: 10.1371/journal.pcbi.1006478.
65. Schuster J, Fatima A, Schwarz F, Klar J, Laan L, Dahl N. Generation of human induced pluripotent stem cell (iPSC) lines from three patients with von Hippel-Lindau syndrome carrying distinct VHL gene mutations. Stem Cell Res. 2019 Jul;38:101474. doi: 10.1016/j.scr.2019.101474.
66. Chittiboina P, Lonser RR. Von Hippel-Lindau disease. Handb Clin Neurol. 2015;132:139-56. doi: 10.1016/B978-0-444-62702-5.00010-X.
67. Tsai CH, Lin LT, Wang CY, Chiu YW, Chou YT, Chiu SJ, Wang HE, Liu RS, Wu CY, Chan PC, Yang MH, Chiou SH, Liao MJ, Lee YJ. Over-expression of cofilin-1 suppressed growth and invasion of cancer cells is associated with up-regulation of let-7 microRNA. Biochim Biophys Acta. 2015 May;1852(5):851-61. doi: 10.1016/j.bbadis.2015.01.007.
68. Li D, Wang H, Song H, Xu H, Zhao B, Wu C, Hu J, Wu T, Xie D, Zhao J, Shen Q, Fang L. The microRNAs miR-200b-3p and miR-429-5p target the LIMK1/CFL1 pathway to inhibit growth and motility of breast cancer cells. Oncotarget. 2017 Jul 12;8(49):85276-85289. doi: 10.18632/oncotarget.19205.
69. Zhang HH, Wang W, Feng L, Yang Y, Zheng J, Huang L, Chen DB. S-nitrosylation of Cofilin-1 Serves as a Novel Pathway for VEGF-Stimulated Endothelial Cell Migration. J Cell Physiol. 2015 Feb;230(2):406-17. doi: 10.1002/jcp.24724.
70. Wioland H, Guichard B, Senju Y, Myram S, Lappalainen P, Jégou A, Romet-Lemonne G. ADF/Cofilin Accelerates Actin Dynamics by Severing Filaments and Promoting Their Depolymerization at Both Ends. Curr Biol. 2017 Jul 10;27(13):1956-1967.e7. doi: 10.1016/j.cub.2017.05.048.
71. Chugh P, Clark AG, Smith MB, Cassani DAD, Dierkes K, Ragab A, Roux PP, Charras G, Salbreux G, Paluch EK. Actin cortex architecture regulates cell surface tension. Nat Cell Biol. 2017 Jun;19(6):689-697. doi: 10.1038/ncb3525.
72. Kanellos G, Frame MC. Cellular functions of the ADF/cofilin family at a glance. J Cell Sci. 2016 Sep 1;129(17):3211-8. doi: 10.1242/jcs.187849.
73. Morgenstern R, Zhang J, Johansson K. Microsomal glutathione transferase 1: mechanism and functional roles. Drug Metab Rev. 2011 May;43(2):300-6. doi: 10.3109/03602532.2011.558511.
74. Buas MF, He Q, Johnson LG, Onstad L, Levine DM, Thrift AP, Gharahkhani P, Palles C, Lagergren J, Fitzgerald RC, Ye W, Caldas C, Bird NC, Shaheen NJ, Bernstein L, Gammon MD, Wu AH, Hardie LJ, Pharoah PD, Liu G, Iyer P, Corley DA, Risch HA, Chow WH, Prenen H, Chegwidden L, Love S, Attwood S, Moayyedi P, MacDonald D, Harrison R, Watson P, Barr H, deCaestecker J, Tomlinson I, Jankowski J, Whiteman DC, MacGregor S, Vaughan TL, Madeleine MM. Germline variation in inflammation-related pathways and risk of Barrett's oesophagus and oesophageal adenocarcinoma. Gut. 2017 Oct;66(10):1739-1747. doi: 10.1136/gutjnl-2016-311622.

Table S9G

Noncoding RNA in human PaCa A818.4 TEX versus A818.4 Tspan8kd cells

Symbol	wt TEX	Tspan8kd cells	noncoding RNA	Gene name	reference
ADIRF-AS1	330	940	lnc	ADIRF antisense RNA 1	
AFAP1-AS1	2892	3317	lnc	AFAP1 antisense RNA 1	1-10
AFG3L1P	563	604	pseudo	AFG3 like matrix AAA peptidase subunit 1, pseudogene	11,12
APCDD1L-AS1	688	11	lnc	APCDD1L antisense RNA 1 (head to head)	13
ARRDC1-AS1	447	610	lnc	ARRDC1 antisense RNA 1	14-16
BAIAP2-AS1	1439	3212	lnc	BAIAP2 antisense RNA 1 (head to head)	17-20
CD24P4	2391	2207	pseudo	CD24 molecule pseudogene 4	
CENPBD1P1	1616	1072	pseudo	CENPB DNA-binding domains containing 1 pseudogene 1	21
CLUHP3	203	465	pseudo	clustered mitochondria homolog pseudogene 3	
CROCCP2	249	173	pseudo	ciliary rootlet coiled-coil, rootletin pseudogene 2	
CRYBB2P1	1058	495	pseudo	crystallin beta B2 pseudogene 1	22,23
CTBP1-AS2	617	495	lnc	CTBP1 antisense RNA 2 (head to head)	24,25
CUTALP	725	1436	lnc	PSMD5 antisense RNA 1 (head to head)	
Cytor	780	365	lnc	cytoskeleton regulator RNA	26-32
DBH-AS1	223	965	lnc	DBH antisense RNA 1	33-35
DHRS4-AS1	469	666	lnc	DHRS4 antisense RNA 1	36-38
DLGAP1-AS1	610	453	lnc	DLGAP1 antisense RNA 1	
DLGAP1-AS2	1326	982	lnc	DLGAP1 antisense RNA 2	
EEF1A1P1	584	239	pseudo	eukaryotic translation elongation factor 1 alpha 1 pseudogene 1	
EEF1A1P11	961	226	pseudo	eukaryotic translation elongation factor 1 alpha 1 pseudogene 11	
EEF1A1P13	572	252	pseudo	eukaryotic translation elongation factor 1 alpha 1 pseudogene 13	
EEF1A1P5	740	328	pseudo	eukaryotic translation elongation factor 1 alpha 1 pseudogene 5	39
EEF1GP1	575	182	pseudo	eukaryotic translation elongation factor 1 gamma pseudogene 1	
EIF3J-AS1	612	532	lnc	EIF3J antisense RNA 1 (head to head)	
ELFN1-AS1	782	936	lnc	ELFN1 antisense RNA 1	40
EPB41L4A-AS1	4712	1156	lnc	EPB41L4A antisense RNA 1	41
FAM83H-AS1	1277	3745	lnc	FAM83H antisense RNA 1 (head to head)	42-45
FBXL19-AS1	933	151	lnc	FBXL19 antisense RNA 1 (head to head)	46-51
FER1L4	1319	5334	lnc	fer-1 like family member 4, pseudogene	52-56
FGD5-AS1	2875	4288	lnc	FGD5 antisense RNA 1	57,58
FIRRE	221	566	lnc	firre intergenic repeating RNA element	59-63
FKBP9P1	120	204	pseudo	FK506 binding protein 9 pseudogene 1	
FOXD2-AS1	278	545	lnc	FOXD2 antisense RNA 1 (head to head)	64-74
GATA2-AS1	214	1911	lnc	GATA2 antisense RNA 1	75
GOLGA2P5	250	1343	lnc	golgin A2 pseudogene 5	76
HAGLR	2666	500	lnc	HOXD antisense growth-associated long non-coding RNA	77-83
HLA-H	278	803	pseudo	major histocompatibility complex, class I, H (pseudogene)	84
HLA-L	193	541	pseudo	major histocompatibility complex, class I, L (pseudogene)	
HOTAIRM1	372	344	lnc	HOXA transcript antisense RNA, myeloid-specific 1	85-88
HOTTIP	377	3111	lnc	HOXA distal transcript antisense RNA	89-92
HOXA11-AS	1689	1577	lnc	HOXA11 antisense RNA	93-96
HOXA-AS2	382	608	lnc	HOXA cluster antisense RNA 2	97-100
HOXA-AS3	101	197	lnc	HOXA cluster antisense RNA 3	101-104
HOXB-AS3	1196	1361	lnc	HOXB cluster antisense RNA 3	105,106
HSPD1P1	448	362	pseudo	heat shock protein family D (Hsp60) member 1 pseudogene 1	107
ILF3-AS1	1065	510	lnc	ILF3 antisense RNA 1 (head to head)	108-110
IPO5P1	452	1272	pseudo	importin 5 pseudogene 1	
JHDM1D-AS1	122	11	lnc	JHDM1D antisense RNA 1 (head to head)	111,112
KCNQ1OT1	66	777	lnc	KCNQ1 opposite strand/antisense transcript 1 (non-protein coding)	113-119
KRT8P12	507	391	lnc	keratin 8 pseudogene 12	120
LBX2-AS1	640	238	lnc	LBX2 antisense RNA 1	121,122
LDHAP7	381	410	pseudo	lactate dehydrogenase A pseudogene 7	
LHFPL3-AS2	542	174	lnc	LHFPL3 antisense RNA 2	
LINC00239	381	183	lnc	long intergenic non-protein coding RNA 239	123
LINC00261	2927	58	lnc	long intergenic non-protein coding RNA 261	124-134
LINC00467	728	497	lnc	long intergenic non-protein coding RNA 467	135-138
LINC00511	1105	1234	lnc	long intergenic non-protein coding RNA 511	139-146
LINC00649	170	138		long intergenic non-protein coding RNA 649	
LINC00659	1057	267	lnc	long intergenic non-protein coding RNA 659	147
LINC00662	1027	393	lnc	long intergenic non-protein coding RNA 662	148-150

Table S9G continued

Symbol	wt TEX	Tspan8kd cells	noncoding RNA	Gene name	reference
LINC00665	3624	2734	lnc	long intergenic non-protein coding RNA 665	151-154
LINC00667	914	1400	lnc	long intergenic non-protein coding RNA 667	155-158
LINC00847	673	540	lnc	long intergenic non-protein coding RNA 847	
LINC00868	120	91	lnc	long intergenic non-protein coding RNA 868	
LINC00896	123	575	lnc	long intergenic non-protein coding RNA 896	
LINC00920	1307	531	lnc	long intergenic non-protein coding RNA 920	159
LINC00941	2789	374	lnc	long intergenic non-protein coding RNA 941	160-162
LINC00963	2772	3788	lnc	long intergenic non-protein coding RNA 963	163-167
LINC01124	163	345	lnc	long intergenic non-protein coding RNA 1124	
LINC01137	527	274	lnc	long intergenic non-protein coding RNA 1137	168
LINC01184	1934	1518	lnc	long intergenic non-protein coding RNA 1184	169
LINC01278	1373	932	lnc	long intergenic non-protein coding RNA 1278	
LINC01578	3573	1100	lnc	long intergenic non-protein coding RNA 1578	
LNCAROD	509	102	lnc	lncRNA activating regulator of DKK1	170
LINC-PINT	120	105	lnc	long intergenic non-protein coding RNA, p53 induced transcript	171-178
LOC100288175	141	113	lnc	uncharacterized LOC100288175	
LOC100506098	186	50	lnc	uncharacterized LOC100506098	
LOC100506688	140	193	lnc	uncharacterized LOC100506688	
LOC103344931	229	572	lnc	uncharacterized LOC103344931	
LOC150776	377	454	pseudo	sphingomyelin phosphodiesterase 4, neutral membrane pseudogene	179
LOC220729	1531	1703	pseudo	succinate dehydrogenase complex flavoprotein subunit A pseudogene	
LOC648987	554	295	lnc	uncharacterized LOC648987	
LOC728554	349	355	lnc	THO complex 3 pseudogene	
LOC93622	1300	942	pseudo	Morf4 family associated protein 1 like 1 pseudogene	180
LRRC37A16P	2630	2680	lnc	leucine rich repeat containing 37 member A16, pseudogene	181
LRRC75A-AS1	47550	14621	lnc	LRRC75A antisense RNA 1	182,183
LSP1P5	1191	331	pseudo	lymphocyte-specific protein 1 pseudogene	184
MAP4K3-DT	570	259	lnc	MAP4K3-DT divergent transcript	185
MCM3AP-AS1	76	278	lnc	MCM3AP antisense RNA 1	186-190
MIR4435-2HG	514	340	lnc	MIR4435-2 host gene	191-197
MIR4458HG	808	569	lnc	MIR4458 host gene	191-193
MIR4712	322	402	lnc	microRNA 4712	191-193
MIR600HG	122	786	lnc	MIR600 host gene	191-193,198
MNX1-AS1	558	450	lnc	MNX1 antisense RNA 1 (head to head)	199-206
MSL3P1	1449	1174	pseudo	male-specific lethal 3 homolog (Drosophila) pseudogene 1	207,208
MTATP6P1	401	13496	pseudo	mitochondrially encoded ATP synthase 6 pseudogene 1	
MTND2P28	167	4129	pseudo	mitoch. encoded NADH:ubiqu. oxidoreduct. core sub.2 pseudogene 28	
MTND4P12	323	16380	pseudo	mitoch. encoded NADH:ubiqu. oxidoreduct. core sub.4 pseudogene 12	
NACA3P	1130	325	pseudo	NACA family member 3 pseudogene	
NCBP2-AS2	2395	1017	lnc	NCBP2 antisense RNA 2 (head to head)	209-212
NNT-AS1	395	916	lnc	NNT antisense RNA 1	213-219
NOP14-AS1	956	398	lnc	NOP14 antisense RNA 1	220
NORAD	4399	13471	lnc	non-coding RNA activated by DNA damage	221-231
NPM1P27	742	478	pseudo	nucleophosmin 1 pseudogene 27	
NUDT16P1	228	826	pseudo	nudix hydrolase 16 pseudogene 1	232
NUTM2A-AS1	622	385	lnc	NUTM2A antisense RNA 1	
OIP5-AS1	2951	5025	lnc	OIP5 antisense RNA 1	233-240
OLMALINC	1966	1692	lnc	oligodendrocyte maturation-associated long intergenic non-coding RNA	241
OTUD6B-AS1	1881	1068	lnc	OTUD6B antisense RNA 1 (head to head)	242,243
PAX8-AS1	761	1584	lnc	PAX8 antisense RNA 1	244-247
PAXIP1-AS1	351	372	lnc	PAXIP1 antisense RNA 1 (head to head)	
PCBP1-AS1	527	349	lnc	PCBP1 antisense RNA 1	38,248
PIK3CD-AS2	878	284	lnc	PIK3CD antisense RNA 2	
PLEKHM1P1	2247	2745	pseudo	pleckstrin homology and RUN domain containing M1 pseudogene 1	
PPP1R14BP3	593	137	pseudo	protein phosphatase 1 regulatory inhibitor subunit 14B pseudogene 3	249
PPP1R26-AS1	85	212	lnc	PPP1R26 antisense RNA 1	250
PROX1-AS1	179	71	lnc	PROX1 antisense RNA 1	251,252
PRR34-AS1	619	119	lnc	PRR34 antisense RNA 1	
PRSS3P1	3007	11	lnc	protease, serine 3 pseudogene 1	
PSMA3-AS1	695	1060	lnc	PSMA3 antisense RNA 1	253
PTOV1-AS1	454	545	lnc	PTOV1 antisense RNA 1	

Table S9G continued

Symbol	wt TEX	Tspan8kd cells	noncoding RNA	Gene name	reference
PVT1	1879	1361	lnc	Pvt1 oncogene (non-protein coding)	254-266
RNASEH1-AS1	905	379	lnc	RNASEH1 antisense RNA 1	199
RNF157-AS1	308	650	lnc	RNF157 antisense RNA 1	
RNF216P1	358	304	pseudo	ring finger protein 216 pseudogene 1	267,268
RP9P	289	160	lnc	retinitis pigmentosa 9 pseudogene	
RPARP-AS1	770	495	lnc	RPARP antisense RNA 1	269
RPL10P3	15300	2971	lnc	ribosomal protein L10 pseudogene 3	
RPL13AP5	1345	581	lnc	ribosomal protein L13a pseudogene 5	
RPL13P12	29649	7251	lnc	ribosomal protein L13 pseudogene 12	270,271
RPL14P1	725	266	lnc	ribosomal protein L14 pseudogene 1	272
RPL18AP3	2941	686	lnc	ribosomal protein L18a pseudogene 3	249
RPL32P3	191	706	lnc	ribosomal protein L32 pseudogene 3	273
RPL3P4	539	128	lnc	ribosomal protein L3 pseudogene 4	272
RPL4P4	502	172	lnc	ribosomal protein L4 pseudogene 4	272
RPLP0P6	1818	447	pseudo	ribosomal protein lateral stalk subunit P0 pseudogene 6	272,274
RPS13P2	791	404	lnc	ribosomal protein S13 pseudogene 2	272
RPS23P8	860	775	lnc	ribosomal protein S23 pseudogene 8	272
RPS28P7	5522	1603	lnc	ribosomal protein S28 pseudogene 7	272
RPS4XP22	3723	1275	lnc	ribosomal protein S4X pseudogene 22	272
SBDSP1	2086	627	pseudo	Shwachman-Bodian-Diamond syndrome pseudogene 1	275-280
SCAND2P	504	553	pseudo	SCAN domain containing 2 pseudogene	281
SDHAP2	160	319	pseudo	succinate dehydrog. complex flavoprotein subunit A pseudogene 2	
SLC25A25-AS1	205	876	lnc	SLC25A25 antisense RNA 1	282
SLC2A1-AS1	268	219	lnc	SLC2A1 antisense RNA 1	
SNHG1	11994	4144	lnc	small nucleolar RNA host gene 1	283-293
SNHG10	136	207	lnc	small nucleolar RNA host gene 10	294,295
SNHG12	1194	493	lnc	small nucleolar RNA host gene 12	296-303
SNHG15	2656	2150	lnc	small nucleolar RNA host gene 15	304-313
SNHG16	2079	1083	lnc	small nucleolar RNA host gene 16	314-321
SNHG17	1755	637	lnc	small nucleolar RNA host gene 17	322-324
SNHG19	2211	617	lnc	small nucleolar RNA host gene 19	325
SNHG3	2044	2281	lnc	small nucleolar RNA host gene 3	326-331
SNHG5	4461	634	lnc	small nucleolar RNA host gene 5	331-339
SNHG6	5536	2650	lnc	small nucleolar RNA host gene 6	340-348
SNHG7	5245	2054	lnc	small nucleolar RNA host gene 7	349-353
SNHG8	19650	2674	lnc	small nucleolar RNA host gene 8	354-357
SNHG9	525	160	lnc	small nucleolar RNA host gene 9	358,359
STAG3L4	584	309	pseudo	stromal antigen 3-like 4 (pseudogene)	
SVIL-AS1	556	288	lnc	SVIL antisense RNA 1	360
THAP9-AS1	2607	2016	lnc	THAP9 antisense RNA 1	
THUMPD3-AS1	488	624	lnc	THUMPD3 antisense RNA 1	361
TMEM147-AS1	174	671	lnc	TMEM147 antisense RNA 1	
TMSB10P1	1613	247	pseudo	thymosin beta 10 pseudogene 1	
TNRC6C-AS1	214	703	lnc	TNRC6C antisense RNA 1	362,362
TPT1P9	1258	513	pseudo	tumor protein, translationally-controlled 1 pseudogene 9	
TRIM52-AS1	396	193	lnc	TRIM52 antisense RNA 1 (head to head)	364
TRPM2-AS	105	90		TRPM2 antisense RNA	365-370
TUBBP6	9	2	pseudo	tubulin beta class I pseudogene 6	
UBA6-AS1	511	247	lnc	UBA6 antisense RNA 1 (head to head)	
VPS9D1-AS1	594	446	lnc	VPS9D1 antisense RNA 1	371
WAC-AS1	487	583	lnc	WAC antisense RNA 1 (head to head)	372,373
WASH3P	596	317	pseudo	WAS protein family homolog 3 pseudogene	374-377
WASH8P	570	325	pseudo	WAS protein family homolog 1 pseudogene	
ZBED5-AS1	485	826	lnc	ZBED5 antisense RNA 1	
ZDHHC8P1	634	549	pseudo	zinc finger DHHC-type containing 8 pseudogene 1	
ZEB1-AS1	184	284	lnc	ZEB1 antisense RNA 1	378-384
ZFAS1	29226	5139	lnc	ZNFX1 antisense RNA 1	385-401
ZNF204P	469	639	pseudo	zinc finger protein 204, pseudogene	
ZNF252P	1469	2058	pseudo	zinc finger protein 252, pseudogene	
ZNF271P	1309	1280	pseudo	zinc finger protein 271, pseudogene	
ZNF37BP	447	1244	pseudo	zinc finger protein 37B, pseudogene	

Table S9G continued

Symbol	wt TEX	Tspan8kd cells	noncoding RNA	Gene name	reference
ZNF670-ZNF695	169	397	lnc	ZNF670-ZNF695 readthrough (NMD candidate)	402,403
ZNF702P	436	812	lnc	zinc finger protein 702, pseudogene	281
ZNF767P	193	493	pseudo	zinc finger family member 767, pseudogene	273

Table S9H
Impact of human PaCa-TEX on human Tspan8kd PaCa cell lncRNA / nc pseudogenes

**Symbol**	**Tspan8kd cells**	**Tspan8kd cells+wt-TEX**	**Tspan8kd cells+ wt TEX: Tspan8kd cells**	**reference[a]**
**upregulated**				
ADIRF-AS1	940	1495	1.59	
AFG3L1P	604	1125	1.86	11,12
APCDD1L-AS1	108	13037	120.72	13
ARRDC1-AS1	610	4427	7.26	14-16
BAIAP2-AS1	3212	5547	1.73	17-20
CD24P4	2207	16742	7.58	
CYTOR	365	1135	3.11	26-32
DBH-AS1	965	2711	2.81	33-35
DLGAP1-AS1	453	1599	3.53	
DLGAP1-AS2	982	10433	10.62	
EPB41L4A-AS1	1156	2066	1.79	41
FGD5-AS1	4288	8376	1.95	57,58
FKBP9P1	204	1262	6.19	
GOLGA2P5	1343	2939	2.19	76
HOXA11-AS	1577	4887	3.10	93-96
HOXB-AS3	1361	3610	2.65	105,106
ILF3-AS1	510	1190	2.33	108-110
IPO5P1	1272	4387	3.45	
JHDM1D-AS1	110	2095	19.04	111,112
KCNQ1OT1	777	1420	1.83	113-119
LDHAP7	410	827	2.02	
LINC00649	138	3021	21.89	
LINC00662	393	613	1.56	148-150
LINC00667	1400	43851	31.32	157,158
LINC00847	540	863	1.60	
LINC00868	91	423	4.65	
LINC00941	374	1881	5.02	160-162
LINC00963	3788	11066	2.92	163-167
LINC01184	1518	19319	12.73	169
LINC01578	1100	4844	4.40	
LNCAROD	102	447	4.38	170
LOC100506688	193	1282	6.64	
MIR4435-2HG	340	2322	6.84	191-197
NCBP2-AS2	1017	1683	1.66	209-212
OTUD6B-AS1	1068	3801	3.56	242,243
PAX8-AS1	1584	2511	1.59	244-247
PPP1R14BP3	137	261	1.91	249
PROX1-AS1	71	1218	17.24	251,252
PSMA3-AS1	1060	21329	20.11	253
PSMD5-AS1	1436	3494	2.43	
PVT1	1361	7057	5.19	254-266
RPL10P3	2971	36460	12.27	
RPL32P3	706	1057	1.50	273
RPS28P7	1603	11522	7.19	272
RPS4XP22	1275	9244	7.25	272
SLC25A25-AS1	876	1859	2.12	282
SNHG1	4144	6966	1.68	283-293
SNHG12	493	749	1.52	296-303
SNHG16	1083	5107	4.72	314-321
SNHG17	637	1826	2.87	322-324

SNHG3	2281	3910	1.71	326-331
SNHG5	634	6140	9.68	331-339
TUBBP6	141	1418	10.06	
ZNF670-ZNF695	397	1926	4.85	402,403
**downregulated**				
ELFN1-AS1	936	615	0.66	40
GATA2-AS1	1911	1127	0.59	75
LINC00239	183	110	0.60	123
LINC00665	2734	1761	0.64	151-154
LINC01124	345	106	0.31	
LOC220729	1703	1125	0.66	
LRRC75A-AS1	14621	3298	0.23	182,183
MTATP6P1	13496	4151	0.31	
MTND2P28	4129	2619	0.63	
MTND4P12	16380	3701	0.23	
NORAD	13471	2558	0.19	221-231
OIP5-AS1	5025	1948	0.39	233-240
OLMALINC	1692	731	0.43	241
PIK3CD-AS2	284	138	0.49	
PLEKHM1P1	2745	1172	0.43	
RPL13AP5	581	353	0.61	
RPL13P12	7251	3389	0.47	270,271
RPS13P2	404	175	0.43	272
SNHG15	2150	1026	0.48	304-313
SNHG8	2674	1567	0.59	354-357
TNRC6C-AS1	703	395	0.56	362,363
ZBED5-AS1	826	396	0.48	

Table S9I

Confirmed and predicted functional annotations

**Symbol*,***	**Functional annotation**
AFAP1-AS1	expression is tightly regulated by DNA methylation; GAS8-AS may downregulate AFAP1-AS1; represses HMG box-containing protein 1 (HBP1) expression by recruiting LSD1 to the HBP1 promoter; promotes malignant phenotype through binding with lysine-specific demethylase 1, represses miR-4695-5p with release of TCF4 and downstream β-catenin activation, competes with miR-384 releasing activin receptor A type I (ACVR1); binds RAP1B repressing miR-181a, activates interferon regulatory factor (IRF)7, the retinoid-inducible protein (RIG)-I-like receptor signaling pathway and Bcl-2; recruits EZH2 to the p21 promoter with epigenetic repression; sequesters miR-181a with upregulation of RAP1B; also affects the actin cytokeratin signaling pathway (1-10)
AFG3L1P	belongs to ATP-dependent proteolytic machines in the inner membrane of mitochondria, crucial for the maintenance of mitochondrial activities. They act as processing enzymes in vivo and might be of relevance for the pathogenesis of neurodegenerative disorders like spastic paraplegia (11,12)
APCDD1L-AS1	associated with poor prognosis in lung, its association with semaphorin5A, 6D, ADAMTS like1, slit guidance ligand3 and tenascinC may provide hints towards the underlying mechanism; also linked to a set of essential spliceosome and RNA binding protein (RBP) genes, including heterogeneous nuclear ribonucleoprotein L (HNRNPL), these RBP-RNA interactions regulate alternative splicing including ncRNA (13)
ARRDC1-AS1	binds phosphatidylserine and phosphoinositol and is engaged in pyroptosis, in breast cancer this arrestin domain containing protein is associated with recurrence (14-16)
BAIAP2-AS1	coexpressed with *IL1* and *IL18* families might function in the clearance process after RSV infection via cytokine-cytokine receptor interaction and TLR signaling; upregulated in hepatitis B virus-related HCC and of prognostic value in cervical squamous cell carcinoma (17-20)
CENPBD1P1	this palmitoyl acyltransferase shares interactors with huntingtin and might play a role in the pathogenesis of Huntington's disease (21)
CRYBB2P1	crystallins are heterogeneous proteins sharing significant homology with small heat shock proteins and having chaperone-like properties, which protect retinal ganglion cells, mutations being linked to cataract. , including the ability to bind and prevent the precipitation of denatured proteins and to increase cellular resistance to stress-induced apoptosis; ncRNA linked to cataract (22,23)
CTBP1-AS2	Sp1-induced lncRNA, stabilizes TLR4 mRNA by recruiting FUS linked to cardiomyocyte hypertrophy; also upregulated in papillary thyroid cancer (24,25)
CYTOR	binds enhancer of zeste homolog 2, silencing of tumor suppressor genes, also binds NCL and Sam68, the heterotrimeric complex activation the NFκB pathway; binding to cytoplasmic β-catenin impeded casein kinase 1 (CK1)-induced β-catenin phosphorylation that enabled it to accumulate and translocate to the nucleus; reciprocally, the β-catenin/TCF complex enhances the transcription activity of CYTOR in the nucleus, thus forming a positive feed-forward circuit, it sponges miR-16, -103a, -199-5p, -138 (26-32)
DBH-AS1	by targeting miR-138 the FAK/Src/ERK pathway becomes activated, also linked to PI3K/Akt pathway activation, involved in HCC and osteosarcoma promotes activation of the PI3K/Akt pathway; competes with miR-138 promoting FAK/Src/ERK pathway activation; induced by hepatitis B virus x protein (HBx), promotes CDK6, CCND1, CCNE1 upregulation and p16, p21 and p27 downregulation (33-35)

DHRS4-AS1	controls 3 DHRS4 genes; where DNA looping and H3 modifications promote DHRS4-A1 expression, it is related to autophagy, downregulation may promote enhancer physically interacts with promoter glioma and RCC progression (36-38)
EEF1A1P5	interferes with translation / translation elongation (39)
ELFN1-AS1	Myclo-2, myc-regulated lncRNA, regulates myc target genes (CDKN1A, CDKN2B), RNA binding proteins HuR and hbRNPK interact with MYclo; CoCa, PC, transformation and tumorigenesis (40)
EPB41L4A-AS1	colocalizes with HDAC2 and NPMI in the nucleolus, upon low EPB41L4A-AS1 released HDAC2 plays a major role in metabolic reprogramming, important in glycolysis and glutaminolysis, frequently downregulated in cancer (41)
FAM83H-AS1	regulates proliferation and invasion through NOTCH, MET/EGFR signaling in CoCa, LungCa, Breast-Ca; (42-45)
FBXL19-AS1	possibly binding miR-203; targets miR-431-5p promoting release of the RAF axis; also sponges miR-718, and miR-346, and promotes WD repeat domain 66 (WDR66) expression, high expression in several cancer, (46-51)
FER1L4	acts as tumor suppressor in several cancer, competes with miR106-5p, interacts with miR-18a-5p that affects Pten expression (52-56)
FGD5-AS1	promotes CoCa through CDCA7 upregulation by sponging miR-302e, regulates miR-142-3p/SOCS6/NFkb pathway (periodontitis) (57,58)
FIRRE	regulates inflammatory genes in macrophages and intestinal epithelial cells by interacting with hnrU, which stabilizes mRNA of selected inflammatory genes; it also functions as a ribonucleic nuclear retention signal that retains otherwise cytoplasmic mRNA in the nucleus, whereas intronic sequences can function as a DNA enhancer element; it becomes activated via Myc and is engaged in Wnt/β-catenin signaling promoting β-catenin nuclear translocation (59-63)
FOXD2-AS1	upregulated in several cancer; binds the enhancer of zeste homolog 2 (EZH2) and lysine (K)-specific demethylase 1A (LSD1) proteins, which mediate EphB3 downregulation; it also negatively regulates the expression of Tribbles pseudokinase 3 (TRIB3), a negative regulator of Akt forming an RNA-DNA complex with the promoter of TRIB3, which leads to Akt activation increasing the expression of the transcription factor E2F1, E2F1 also binds to the FOXD2-AS1 promoter region creating a FOXD2-AS1/Akt/E2F1 feedback loop; CREB1 also induces FOXD2-AS1 transcription, which via competing with miR-185 promotes Akt upregulation; by competing with miR-363-5p, the S100A1 pathway becomes activated (64-74)
GATA2-AS1	frequent altered in GaCa (75)
GOLGA2P5	may be important for TERT regulation in p53-deficient cancer (76)
HAGLR / HOXD-AS1	transcription is promoted by STAT3, it is engaged in many cancer progression, mostly via capturing miRNA, it competes with miR-130a-3p with liberation of SOX4 supporting EZH2 and MMP2 expression; HOXD-AS1 recruited WDR5 to directly regulate the expression of target genes by mediating histone H3 lysine 4 tri-methylation (H3K4me3), targeting miR-130a-3p also releases zinc finger E-box homeobox 1 (ZEB1) from repression as well as E2F8; by targeting miR-133a-3p it may contribute to activation of Wnt/β-catenin signaling, by competing with miR-608 it contributes to frizzled receptor 4 (FZD4) activation; upregulation of Rho GTPase activating protein 11A (ARHGAP11A) may be due to competitively binding to miR-19a; it also targets miR-147a and others (77-83)
HLA-H	impact on HLA response (84)
HOTAIRM1	PU.1 may activate HOTAIRM1 expression through binding to its regulatory region; HOTAIRM1 mediated demethylation of histone H3K9 and H3K27 and reduced DNA methylation levels by sequester epigenetic modifiers G9a and EZH2, which are H3K9me and H3K27me specific histone methyltransferases, and DNA methyltransferases (DnmTs) away from the transcription start site of HOXA1 gene (example of transcriptional control over the chromatin state of gene by contributing to physical dissociation of chromatin loops at the cluster proximal end) (85-88)
HOTTIP	sponges miR-4301, TGFBR1 and -2 become upregulated by sponging miR-148a, regulates insulin-like growth factor2 (IGF2) via miR-615, HOTTIP could bind to enhancer of zeste homolog 2 (EZH2) and lysine specific demethylase 1 (LSD1), thereby repressing LATS2 expression, also sponges miR-216a-5p at 3'-UTR, miR-637 and miR-216a and is engaged in autophagy via the PI3K/Akt/Atg13 pathway, it mediates chromatin organization by associating with the CCCTC-binding factor (CTCF) that serves as an insulator by organizing HOXA cluster (89-92)
HOXA11-AS	recruits EZH2 along with the histone demethylase LSD1 or DNMT1, which function as a scaffold, HOXA11-AS also functions as a sponge for miR-1297, antagonizing its ability to repress EZH2 protein translation, functions as ceRNA for miR-214-3p, which in turn positively regulates EZH2 expression, interacts with WDR5 and promotes β-catenin transcription, represses P21 transcription, and induce KLF2 mRNA degradation via interacting with STAU1, via sponging miR-200b several EMT genes become upregulated; also targets miR-124-3p and miR-506-3p that regulates NIMA-related kinase 6(NEK6), sponges let-7i, which represses ABCC10 (93-96)
HOXA-AS2	could interact with EZH2 (enhancer of zeste homolog 2), LSD1 (lysine specific demethylase 1) and recruit them to p21 (CDKN1A), KLF2 promoter regions to repress their transcription, sponges miR-520-3c, regulating TGFBR2 and RELA, and miR-223-3p repressing glypican 3 (GPC3), binds miR-373 promoting epidermal growth factor receptor (EGFR) expression with increased expression levels of VE-cadherin, MMP-2 and MMP-9 via activating the PI3K/Akt pathway interact with enhancer of zeste homolog 2 Polycomb repressive complex (97-100)
HOXA-AS3	colocalizes with NFκB gene promoters positively regulating activity through control of the expression of the NFκB inhibitor protein IκBα and the acetylation status at the K310 site of p65, interacts with Enhancer Of Zeste 2 (EZH2) required for H3 lysine-27 trimethylation (H3K27me3) of key osteogenic transcription factor Runx2 during MSC differentiation, sponges miR-29c promoting MEK/ERK pathway activation, poor prognosis glioma, MSC commitment, EC activation (101-104)
HOXB-AS3	regulates p53 expression by binding to DNMT1, upregulated in several cancer and leukemia (105,106)
HSPD1P1	possibly engaged in microtubule elongation (107)
ILF3-AS1	induced by nuclear transcription factor SP1, sponges miR-212 promoting SOX5 release from repression, interacts with EZH2, promoting EZH2 binding to the miR-200b/a/429 promoter and repressing miR-

	200b/a/429, represses the binding of EZH2 to the ILF3 promoter inducing euchromatin formation and ILF3 transcription activation creating an ILF3 - ILF3-AS1 positive feedback loop, sponges miR-200b/a, -429 (108-110)
JHDM1D-AS1	protects from apoptosis via inhibiting DNAJC10 that affects Bcl-2 and eIF2α phosphorylation, may be engaged in HGF and FGF1 upregulation (111,112)
KCNQ1OT1	interacts with chromatin and regulates transcription of many genes, sponges miR-140-5p releasing SOX4 from repression, and miR-217 releasing ZEB1 that in a feedback loop enhances KCNQ1OT1 transcription; targeting miR-214 promotes caspase-1 release from repression (113-119)
KRT8P12	may be linked to Huntington (120)
LBX2-AS1	may promote activation of the NOTCH pathway, frequent in RCC, angiogenesis-related (121,122)
LINC00239	promotes chemoresistance via PI3K/Akt/mTOR in AML (123)
LINC00261	a tumor suppressive ncRNA, epigenetically regulated by hypermethylation of the DNA damage response region, it downregulates Snail; targets miR-558 releasing TIMP4 from repression, suppressive via targeting miR-324-3p and Wnt signaling, also targets miR-182, miR-183, miR-153, miR-27a, and miR-96 promoting FOXO1 release, sponging miR-522-3p inhibits Wnt signaling; by miR-132-3p binding it regulates BCL2L11; it increases the methylation of the dihydropyrimidine dehydrogenase (DPYD) promoter through the recruitment of DNA methyltransferase (DNMT), which, in turn, decreases DPYD activity (124-134)
LINC00467	upregulation by STAT1, downregulated by N-Myc; epigenetically silences DKK1 by recruiting enhancer of zeste 2 polycomb repressive complex 2 subunit (EZH2) to DKK1 promoter, which allows for Wnt/β-catenin pathway activation; also recruits EZH2 to the HtrA serine peptidase 3 (HTRA3) promoter to inhibit its expression; sponges miR-9-5p promoting peroxisome proliferator-activated receptor alpha (PPARA) release (135-138)
LINC00511	AP2γ binds to the promoter region activating transcription; acts as a modular scaffold of EZH2/PRC2 complexes, coordinated their localization, and specified the histone modification pattern on the target genes, including p57 and p21; competes with miR-29b-3p releasing VEGFA andCyr61; competing with miR-765 releases laminin subunit gamma 2 (LAMC2); competing with miR-185-3p releases E2F1 protein that promotes Nanog transcription; competing with miR-15a-3p allows for Wnt/β-catenin signaling activation; competes with miR-29c releases cyclin dependent kinase 6 (CDK6) from repression; (139-146)
LINC00659	oncogenic in CoCa, growth inhibition and apoptosis, possibly via suppressing PI3K/Akt signaling (147)
LINC00662	suppresses the HIPPO-YAP1 pathway by sponging miR-497-5p; promotes stemness by interacting with Lin28; upregulates ROCK1 via sponging miR-340-5p (148-150)
LINC00665	interacts with EZH2 and regulate the phosphatidylinositol 3-kinase (PI3K)/AKT pathway; targets miR-186-5p leading to MAP4K3 activation; targets miR-98 activating the downstream AKR1B10-ERK signaling pathway (151-154)
LINC00667	associated with NSCLC progression and OvCa recurrence. Regulates vasculogenesis through miR-429 that represses ALDH1A1, associates with miR-19b-3p regulating connective tissue growth factor (CTGF) expression (155-158)
LINC00920,CALIC	associates with hnRNP-L promoting RTK AXL upregulation (CoCA metastasis) (159)
LINC00941	correlated with increased expression of TDRKH-AS1, which regulates telomere organization and EZH2-mediated epigenetic silencing of CDKN1A, CDKN1B and IL24 ; competes with miR-34a accompanied by Snail upregulation and EMT, represses SPRR5, essential in keratinocyte differentiation (160-162)
LINC00963	prevents glycolytic kinase PGK1 ubiquitination leading to AKT/mTOR signaling pathway activation; targets miR-608 which releases NACC1; binds miR-204-3p releasing fibronectin-1 (FN1) from repression; sponges miR-1193, releasing SOX4 from repression miR-608/NACC! in melanoma, fibrosis (kidney), LuCa metast asis via PGK1-Akt/mTOR (163-167)
LINC01137	suppressive in several cancer, located in a suppressive lncRNA nexus (168)
LINC01184	engaged in stress response in induced pluripotent stem cells (169)
LNCAROD	enhances DKK1 transcription via the released form with evidence that the regulatory interaction requires dissociation (170)
LINC-PINT	tumor suppressor, interacts with PRC2, regulating EGR1 transcription, proximity of co-regulated genomic loci essential; the circular form of the LINC-PINT interacts with polymerase associated factor complex (PAF1c) and inhibits the transcriptional elongation of multiple oncogenes; binds miR-208a-3p with release of JUN and MAPK pathway activation and release of programmed cell death 4 (PDCD4); targets miR-425-5p that via PTCH1 is engaged in the HH pathway; sponges miR-543 and miR-576-5p; may also downregulate lncRNA BANCR (171-178)
LOC150776	possibly engaged in ES growth and differentiation (179)
LOC93622	interacts with p12CDK2AP1, inhibits cell cycle progression (180)
LRRC37A16P	may be engaged in filipodia formation (181)
LRRC75A-AS1	highly associated with the target genes of transcription factors TP53 and ETS variant 6, may also act as a negative regulator of vascular calcification (182,183)
LSP1P5	frequently associated with chromosomal translocation in CLL (184)
MAP4K3-DT	together with other loci engaged in childhood obesity (185)
MCM3AP-AS1	targets miR-211 with KLF5release from repression associated with AGGF1 promoter, also affects the PII3K/Akt and ERK1/2 signaling pathway, important in angiogenesis; sponges miR-211-5p that affects secreted protein acidic and cysteine rich (SPARC) expression; targets miR-194-5p promoting FOXA1 expression; also targets miR-455 (186-190)
MIR4435-2HG	host gene, interacts with β-catenin preventing its degradation by the proteasome that forces EMT; is associated with differential P38/MAPK, VEGF, cell adhesion molecules and NOD-like receptor signaling pathway activation; binds to and inhibits desmoplakin (DSP) promoting WNT/β-catenin signaling and EMT; functions as a miRNA sponge of miR-125a-5p and miR-125b-5p increasing CD44 expression (191-197)
MIR4458HG	host gene (191-193)

MIR4712	host gene (191-193)
MIR600HG	host gene ; 193 one of five lncRNA predictive for PaCa progression (191-193,198)
MNX1-AS1	upregulated in many cancer; affects CDK4, cyclin D, Bax, and Bcl-2 expression; strengthens pERK1/ and pJNK expression; targets miR-34a with release of SIRT1; sponges miR-218-5p releasing downstream SEC61A1 from repression; also competes with miR-4443 and miR-218-5p that promotes COMMD8 expression; interact with miR-527 facilitating BRF2 expression; antagonizes DHX36 (199-206)
MSL3P1	a protein complex including MSL3P1. homologous to the drosophila MLS complex is responsible for histone H4 acetylation, critical for DNA damage response and double-strand break repair histone H4 acetylation (207,208)
NCBP2-AS2	represses oncogenic signaling (KRAS) in HCC, associated with increased β-catenin and glycogen synthase kinase 3β (GSK-3β) phosphorylation, engaged in damage response in ES; abundant in CAF, promotes VEGFA secretion and VEGFR signaling in EC (209-212)
NNT-AS1	engaged in the MAPK/Slug signaling pathway; competes with miR-363 for CDK6; sponges miR-142-3p with activation of the ZEB1 axis; sponging miR-424 releases E2F1 from repression; sponging miR-320a increases beta-catenin, RUNX2 and IGF-1R expression and Akt activation, also sponges miR-129-5p and miR-363 (213-219)
NOP14-AS1	strong evidence for not drawing conclusion from sense-antisense pairs on regulatory mechanims (220)
NORAD	repressive or oncogenic; sponges miR-608 releasing FOXO6; sponges miR-615-3p releasing JunB; sponges miR-590 releases SIP1; targets miR-373 releasing Wee1; upregulates TGFβ and inhibits the interaction of importin β1 with Smad3, inhibiting nuclear accumulation; competes with miR-125a-3p releasing RhoA; binds the RNA binding SAM68 (KHDRBS1); required for antagonizing Pumilio contributing to genomic stability maintenance; sponges miR-136-5p (221-231)
NUDT16P1	possibly engaged in Alzheimer disease by causing aggregates with β-amyloid (232)
OIP5-AS1	targets miR-410 releasing Wnt7b/b-catenin; sponges miR-129-5p, releasing SOX2; targets miR223 with release of CDK14; targets miR-378-3p which also releases CDK4 and CDK6; binds piR-30188 and miR-367-3p releasing CEBPA creating a feedback loop via downstream TRAF4; sponges miR-424 releasing RNA binding protein HUR (233-240)
OLMALINC	critical for oligocentrocyte maturation (241)
OTUD6B-AS1	regulates apoptosis and proliferation via cyclinD1; may be suppressive, decreases Wnt-b-catenin and EMT-related proteins may regulate apoptosis in systemic sclerosis (242,243)
PAX8-AS1	can repress or promote cancer; targets miR-17-5p suppressing PTEN, CDKN1A and ZEB4; inhibited by Myc (244-247)
PCBP1-AS1	one of the hub ncRNA in cancer; belongs to autophagy-related lncRNA with upregulation of several cancer related pathways (38,248)
PPP1R14BP3	may be engaged in modulating RNA-binding E3 ubiquitin ligases (249)
PPP1R26-AS1	oncogenic in triple- breast cancer, regulates TGFβ-Jak/STAT pathway (250)
PROX1-AS1	promotes proliferation and migration via FGFR1 activation; body mass associated, linked to type 2 diabetes (251,252)
PSMA3-AS1	forms a duplex with PSAM3 promoting PSMA3 transcription and stability, which is associated with proteasome inhibitor sensitivity (multiple myeloma associated with OS) (253).
PVT1	involved in many tumor progression, the promoters of PVT1 and MYC are closely located and compete for engagement with four intragenic enhancers in the PVT1 locus, thereby allowing the PVT1 promoter to regulate pause release of MYC transcription via promoter competition, but only from the same chromosome; associated with EZH2 the association being required for p15 and p16 repression; interacts with FOXM1 creating a positive feedback loop; sponges miR-448 with release of SERBP1, sponges miR-190a-5p and -488-3p with release of Myocyte enhancer factor 2C (MEF2C) and upregulation of JAGGED1; binds miR-149 with release of IL1β and additional inflammatory cytokines; many additional miRNA sponges being reported (254-266)
RNASEH1-AS1	binds to the DEAH box polypeptide 36 (DHX36) RNA helicase via its G-quadruplex-forming sequence and inhibits DHX36 unwinding antagonizing DHX36 in CoCa (199)
RNF216P1	interacts with RIP, inhibits TNF- & IL1-induced NFκB activation (267,268)
RPARP-AS1	one of the genes linked to autism (269)
RPL13P12	RPA-ssDNA complexes activate Rad3-related (ATR) kinase, which repairs replication forks during stress, the DNA damage response is impaired by RPA modulation (270,271)
RPL14P1	abundant in the genome, a comparison of ribosomal pseudogenes awaits functional annotation (272)
RPL18AP3	possibly RNA-binding E3 ubiquitin ligases (249)
RPL32P3	essential components of the spliceosome are RNA binding protein (RBP), most notably heterogeneous nuclear ribonucleoprotein L (HNRNPL) that directly regulate the alternative splicing of a set of RNAs and also regulate circular RNA formation via back splicing. HNRNPL aberrantly expression is abundant in cancer (273)
RPL4P4	see (272)
RPLP0P6	see (272), also engaged in ubiquitin-proteasome activity in ES (274)
RPS13P2	see (272)
RPS23P8	see (272)
RPS28P7	see (272)
RPS4XP22	see (272)
SBDSP1	associated with the ribosomopathy Swachman-Diamond syndrome, where SBDS controls the final step in cytoplasmic 60S ribosomal subunit maturation to a quality control of structural and functional integrity of the nascent particle that is distorted by SBDSP1, promotes CoCa invasion acting as hub gene in NFκB interactions (275-280)
SCAND2P	possibly engaged in alternative polyadenylation (281)
SLC25A25-AS1	tumor suppressor that mitigates ERK and p38 signaling (282)

SNHG1	small nucleolar host gene, induced by SP1; directly interacts with Polycomb Repressive Complex 2 (PRC2) and modulate the histone methylation of promoter of Kruppel like factor 2 (KLF2) and Cyclin dependent kinase inhibitor 2B (CDKN2B) in the nucleus; transfer of miR-21 into the nucleus promotes SNHG1 expression that promotes Akt activation; promotes cell proliferation by regulating p53; interferes with multiple miRNA, e.g. competes miR-195-5p releasing PDCD4, targets miR-154-5p and -376b-3p with release of FOXP2; targets miR-101-3p with ROCK1 release from repression; targeting miR-497 releases IGF1R from repression; represses miR-140 with downstream ADAM10 release; targets miR-195 releasing BCL2L2; sponges miR-154-5p releasing cyclinD2 (CCND2); sponges miR-302, -372, -373, -520 which leads to TGFBR2/SMAD3 and RAB11A/Wnt/β-catenin pathways activation; targeting miR-199a and miR-18a promotes angiogenesis partly via release of HIF1α and VEGF signaling (283-293)
SNHG10	SNHG10 acts upstream of its homolog SCARNA13, it sponges miR-150-5p and interacts with RPL4 mRNA to increase c-Myb; which enhances SNHG10 and SCARNA13 by regulating promoter activity creating a feedback loop; the small nucleolar SNHG10 also is enriched in CD47+ EV, but small nuclear RNA are not enriched (294,295)
SNHG12	similar to SNHG1, sponges a large range of miRNA, e.g. miR-195 contribution to Wnt/β-catenin activation; sponges miR-133b; sponges miR-199a activating sirtuin 1(SIRT1) that leads to AMPK activation; targets miR-103-3p that targets FOXP1; targets miR-320a with release of Myeloid cell leukemia 1 (MCL1); associates with and stabilizes HuR; SNHG12-miR-195-SOX5 works in a feedback loop; SNHG12-miR-195-5p promotes Notch2-Notch signaling pathway activation (296-303)
SNHG15	expression regulated by Myc; stabilizes Slug by preventing degradation in the proteasome; may be involved in NFκB signaling activation; targets several miRNA, beside others miR-338-3p that regulates FKBP prolyl isomerase 1A (FKBP1A) as well as FOS and RAB14; interacts with and regulates AIF and via AIF BAG3 and ERBB3; upregulated YAP1 by sponging miR-200a-3p; regulating CDK14 protein via sponging miR-486; sponges miR-211-3p with upregulation of EMT genes; targets miR-153 with release of VEGFA and Cdc42 promoting EC proliferation (304-313)
SNHG16	regulated by the Wnt signaling pathway; binds Ago with 27 AGO/miRNA target sites; sponges beside others miR1301 promoting BCL9 expression; regulates miR-98-5p; regulates the miR-15a/16 cluster with upregulation of inflammatory genes; targets miR-205 with upregulation of ZEB1; also targets the 3'UTR of Stearoyl-CoA Desaturase (SCD) suggesting a role in lipid metabolism. (314-321)
SNHG17	associated with EZH2 required for epigenetic repression of cyclin-dependent protein kinase inhibitors p15 and p57epigenetic silencing p57 (322-324)
SNHG19	negatively correlated with Alzheimer, synaptic vesicle cycle and endocytosis overpresented (325)
SNHG3	repressing KLF2 and p21 via recruiting enhancer of EZH2 to the promoter; targets miR-384 with WEE1 release promoting migration/invasion; targets miR-326 enhancing SMAD3 and ZEB1, targets miRNA-151a-3p releasing RAB22 A; related to energy metabolism by regulating miRNAs and EIF4AIII with target sites on PKM, PDHB, IDH2, and UQCRH; sponges miR-182-5p upregulating c-Myc; (326-331)
SNHG5	methioninase (METase) promotes SNHG5 upregulation that competes with miR-20a; sponges miR-26a and associates with ROCK1and SOX2; sponges miR-377 releasing Casp1; sponges miR-182-5p releasing cMyc; stabilizes 121 target transcripts by blocking their degradation by STAU1; acts via the miR-32/KLF4 axis; prevents MTA2 translocation from the cytoplasm into the nucleus, significantly increased acetylation of histone H3 and p53 might affect trapping MTA2 in the cytosol interfering with nucleosome remodeling and the histone deacetylation complex (331-339)
SNHG6	activates TGF-β/Smad signaling pathway by targeting UPF1 and ZEB1 regulation; repressed p21 transcription through recruiting EZH2 to the p21 promoter, yet can also suppress EZH2 via JNK activation; promoted expression of MAT2A by suppressing direct binding of miR-1297, suppressing production of the universal methyl donor S-adenosylmethionine (SAMe) impacts global DNA methylation levels; sponges miR-1001 with activation of Wnt/β-catenin; targets miR-26a-5p activating the MAPK6 pathway; sponging miR-26a-5p regulates E2F7; sponges miR-101-3p increasing ZEB1 at the posttranscriptional level and silences at the transcriptional level p27 by recruiting EZH2 to the promoter (340-348)
SNHG7	targets miR-34a with upregulation of Notch1, BCL-2, CDK6, and SMAD4; sponges miR-503with release of Cyclin D1; targets miR-5095 with release of CTNNB1; promotes increase of Fas apoptotic inhibitory molecule2 (FAIM2) (349-353)
SNHG8	sustains SC proliferation by promoting the transcription of ribosomal proteins; targets miR-542-3p with release of CCND1/CDK6 and Caspase-3 activation; targets miR-152 releasing c-MET; targets mi R-149 releasing phosphatase Mg2+/Mn2+ dependent 1F (PPM1F) from repression (354-357)
SNHG9	targets miR-199a-5p promoting Wnt2 axis activation; cooperates with additional lncRNA (358,359)
SVIL-AS1	cooperates with addition lncRNA in fibroblast like cells promoting rheumatoid arthritis (360)
THUMPD3-AS1	forms with additional lncRNA and 61 miRNA a network a network in regulating cancer progression (361)
TNRC6C-AS1	inhibits STK4 by promoting methylation and reducing MST1 and LATS1/2 proteins with decrease of YAP1 phosphorylation; targets miR-129-5p with upregulation of UNC5B (362,363)
TRIM52-AS1	tumor suppressor in RCC (364)
TRPM2-AS	ELK1-induced overexpression, targets miR-195 promoting high-mobility group AT-hook 1 (HMGA1) upregulation; sponges miR-140-3p releasing PYCR1; activates the p53-p66shc pathway; regulates phagosomal acidification essential for bacterial killing by macrophages; functions as a transducer converting oxidative stress into Ca(2+) signaling, critical in ROS promoted diseases and NK degranulation (365-370)
VPS9D1-AS1	cancer promoting activity, upregulating c-Myc by competing miR-184 (371)
WAC-AS1	germline specific, binds P-Element induced wimpy testis (PIWI) proteins, the interacting RNA are ncRNA suggested to be also important in cancer (372,373)
WASH3P	Proteins of the Wiskott-Aldrich syndrome protein (WASP) are nucleation-promoting factors for the Arp2/3 complex, driving the generation of branched actin filaments engaged in lamellipodia/filopodia formation, endocytosis, phagocytosis and the generation of cargo-laden vesicles; also engaged in promoting actin dynamics at the centrosome; in the nucleus they serve as molecular platforms for the assembly of epigenetic and transcriptional machinery (374-377)

ZEB1-AS1	competes with miR-200b releasing Fascin-1 (FSCN1); binds and stabilizes IL11 strengthening STAT3 signaling; binds and recruits histone methyltransferase MLL1 to the promoter region of ZEB1, inducing H3K4me3 modification with activating ZEB1 transcription; sponging miR-200s contributes to ZEB1 expression; suppresses p15 expression; supports MMP2, MMP9, N-cadherin, and Integrin-β1 and decreases E-cadherin expression (378-384)
ZFAS1	sponges miR-135a; via competing miRNA associated with translation, rRNA processing, intra-Golgi vesicle-mediated transport, ribosome, and ubiquitin-mediated proteolysis; targets miR-200b promoting Wnt/β-catenin signaling; competes miR/10a releasing SKA1; competes with miR-150 releasing Myb and Sp1; targets miR-150-5p releasing Sp1; sponges miR-329 and -484; binds and inhibits SERCA2a (sarcoplasmic reticulum Ca^{2+}-ATPase 2a); interacts with EZH2 and LSD1/CoREST to repress KLF2 and NKD2 transcription; sponging miR-150 releases ZEB1, MMP14 and MMP16 from repression (385-401)
ZNF670-ZNF695	example of a nc conjoined gene, conjoined genes regulating genomic evolution (402,403)
ZNF702P	ncRNA with N6-methyladenosine enriched in 3'UTR near stop codon. VIRMA & METTL3 core components of mRNA methylation near stop codon (281)
ZNF767P	may regulate RNA splicing, HNRNPL important component of RNA binding protein (RBP), relevant in ProstateCa for androgen receptor (273)

*, *: red: upregulated in wt-TEX treated Tspan8kd tumor cells, green: downregulated in wt-TEX treated Tspan8kd tumor cells

References (Table S9G-9I)

1. Yu S, Yang D, Ye Y, Liu P, Chen Z, Lei T, Pu J, Liu L, Wang Z. Long noncoding RNA actin filament-associated protein 1 antisense RNA 1 promotes malignant phenotype through binding with lysine-specific demethylase 1 and repressing HMG box-containing protein 1 in non-small-cell lung cancer. Cancer Sci. 2019 Jul;110(7):2211-2225. doi: 10.1111/cas.14039.
2. Wu XB, Feng X, Chang QM, Zhang CW, Wang ZF, Liu J, Hu ZQ, Liu JZ, Wu WD, Zhang ZP, Liu XQ. Cross-talk among AFAP1-AS1, ACVR1 and microRNA-384 regulates the stemness of pancreatic cancer cells and tumorigenicity in nude mice. J Exp Clin Cancer Res. 2019 Feb 28;38(1):107. doi: 10.1186/s13046-019-1051-0.
3. Li R, Liu S, Li Y, Tang Q, Xie Y, Zhai R. Long noncoding RNA AFAP1-AS1 enhances cell proliferation and invasion in osteosarcoma through regulating miR-4695-5p/TCF4-β-catenin signaling. Mol Med Rep. 2018 Aug;18(2):1616-1622. doi: 10.3892/mmr.2018.9131.
4. Ji D, Zhong X, Jiang X, Leng K, Xu Y, Li Z, Huang L, Li J, Cui Y. The role of long non-coding RNA AFAP1-AS1 in human malignant tumors. Pathol Res Pract. 2018 Oct;214(10):1524-1531. doi: 10.1016/j.prp.2018.08.014.
5. Yin D, Lu X, Su J, He X, De W, Yang J, Li W, Han L, Zhang E. Long noncoding RNA AFAP1-AS1 predicts a poor prognosis and regulates non-small cell lung cancer cell proliferation by epigenetically repressing p21 expression. Mol Cancer. 2018 May 24;17(1):92. doi: 10.1186/s12943-018-0836-7.
6. Tang XD, Zhang DD, Jia L, Ji W, Zhao YS. lncRNA AFAP1-AS1 Promotes Migration and Invasion of Non-Small Cell Lung Cancer via Up-Regulating IRF7 and the RIG-I-Like Receptor Signaling Pathway. Cell Physiol Biochem. 2018;50(1):179-195. doi: 10.1159/000493967.
7. Chen G, Peng L, Zhu Z, Du C, Shen Z, Zang R, Su Y, Xia Y, Tang W. LncRNA AFAP1-AS Functions as a Competing Endogenous RNA to Regulate RAP1B Expression by sponging miR-181a in the HSCR. Int J Med Sci. 2017 Sep 3;14(10):1022-1030. doi: 10.7150/ijms.18392.
8. Zhao Y, Chu Y, Sun J, Song R, Li Y, Xu F. LncRNA GAS8-AS inhibits colorectal cancer (CRC) cell proliferation by downregulating lncRNA AFAP1-AS1. Gene. 2019 Aug 20;710:140-144. doi: 10.1016/j.gene.2019.05.040.
9. He J, Wu K, Guo C, Zhou JK, Pu W, Deng Y, Zuo Y, Zhao Y, Liu L, Wei YQ, Peng Y. Long non-coding RNA AFAP1-AS1 plays an oncogenic role in promoting cell migration in non-small cell lung cancer. Cell Mol Life Sci. 2018 Dec;75(24):4667-4681. doi: 10.1007/s00018-018-2923-8.
10. Bo H, Gong Z, Zhang W, Li X, Zeng Y, Liao Q, Chen P, Shi L, Lian Y, Jing Y, Tang K, Li Z, Zhou Y, Zhou M, Xiang B, Li X, Yang J, Xiong W, Li G, Zeng Z. Upregulated long non-coding RNA AFAP1-AS1 expression is associated with progression and poor prognosis of nasopharyngeal carcinoma. Oncotarget. 2015 Aug 21;6(24):20404-18.
11. Kremmidiotis G, Gardner AE, Settasatian C, Savoia A, Sutherland GR, Callen DF. Molecular and functional analyses of the human and mouse genes encoding AFG3L1, a mitochondrial metalloprotease homologous to the human spastic paraplegia protein. Genomics. 2001 Aug;76(1-3):58-65.
12. Koppen M, Bonn F, Ehses S, Langer T. Autocatalytic processing of m-AAA protease subunits in mitochondria. Mol Biol Cell. 2009 Oct;20(19):4216-24. doi: 10.1091/mbc.E09-03-0218.
13. Luo Y, Xuan Z, Zhu X, Zhan P, Wang Z. Long non-coding RNAs RP5-821D11.7, APCDD1L-AS1 and RP11-277P12.9 were associated with the prognosis of lung squamous cell carcinoma. Mol Med Rep. 2018 May;17(5):7238-7248. doi: 10.3892/mmr.2018.8770.
14. Wauters L, Versées W, Kortholt A. Roco Proteins: GTPases with a Baroque Structure and Mechanism. Int J Mol Sci. 2019 Jan 3;20(1). pii: E147. doi: 10.3390/ijms20010147.
15. Tomkins JE, Dihanich S, Beilina A, Ferrari R, Ilacqua N, Cookson MR, Lewis PA, Manzoni C. Comparative Protein Interaction Network Analysis Identifies Shared and Distinct Functions for the Human ROCO Proteins. Proteomics. 2018 May;18(10):e1700444. doi: 10.1002/pmic.201700444.
16. Liu H, Li J, Koirala P, Ding X, Chen B, Wang Y, Wang Z, Wang C, Zhang X, Mo YY. Long non-coding RNAs as prognostic markers in human breast cancer. Oncotarget. 2016 Apr 12;7(15):20584-96. doi: 10.18632/oncotarget.7828.
17. Zhang Q, Chao TC, Patil VS, Qin Y, Tiwari SK, Chiou J, Dobin A, Tsai CM, Li Z, Dang J, Gupta S, Urdahl K, Nizet V, Gingeras TR, Gaulton KJ, Rana TM. The long noncoding RNA ROCKI regulates inflammatory gene expression. EMBO J. 2019 Apr 15;38(8). pii: e100041. doi: 10.15252/embj.2018100041.
18. Qian Z, Zhang Z, Wang Y. T cell receptor signaling pathway and cytokine-cytokine receptor interaction affect the rehabilitation process after respiratory syncytial virus infection. PeerJ. 2019 Jun 12;7:e7089. doi: 10.7717/peerj.7089.
19. Gong X, Wei W, Chen L, Xia Z, Yu C. Comprehensive analysis of long non-coding RNA expression profiles in hepatitis B virus-related hepatocellular carcinoma. Oncotarget. 2016 Jul 5;7(27):42422-42430. doi: 10.18632/oncotarget.9880.
20. Mao X, Qin X, Li L, Zhou J, Zhou M, Li X, Xu Y, Yuan L, Liu QN, Xing H. A 15-long non-coding RNA signature to improve prognosis prediction of cervical squamous cell carcinoma. Gynecol Oncol. 2018 Apr;149(1):181-187. doi: 10.1016/j.ygyno.2017.12.011.
21. Butland SL, Sanders SS, Schmidt ME, Riechers SP, Lin DT, Martin DD, Vaid K, Graham RK, Singaraja RR, Wanker EE, Conibear E, Hayden MR. The palmitoyl acyltransferase HIP14 shares a high proportion of interactors with huntingtin: implications for a role in the pathogenesis of Huntington's disease. Hum Mol Genet. 2014 Aug 1;23(15):4142-60. doi: 10.1093/hmg/ddu137.

22. Garnai SJ, Huyghe JR, Reed DM, Scott KM, Liebmann JM, Boehnke M, Richards JE, Ritch R, Pawar H. Congenital cataracts: de novo gene conversion event in CRYBB2. Mol Vis. 2014 Nov 6;20:1579-93. eCollection 2014.
23. Piri N, Kwong JM, Caprioli J. Crystallins in retinal ganglion cell survival and regeneration. Mol Neurobiol. 2013 Dec;48(3):819-28. doi: 10.1007/s12035-013-8470-2.
24. Luo X, He S, Hu Y, Liu J, Chen X. Sp1-induced LncRNA CTBP1-AS2 is a novel regulator in cardiomyocyte hypertrophy by interacting with FUS to stabilize TLR4. Cardiovasc Pathol. 2019 Sep - Oct;42:21-29. doi: 10.1016/j.carpath.2019.04.005.
25. Ma B, Liao T, Wen D, Dong C, Zhou L, Yang S, Wang Y, Ji Q. Long intergenic non-coding RNA 271 is predictive of a poorer prognosis of papillary thyroid cancer. Sci Rep. 2016 Nov 11;6:36973. doi: 10.1038/srep36973.
26. Li M, Wang Q, Xue F, Wu Y. lncRNA-*CYTOR* Works as an Oncogene Through the *CYTOR*/miR-3679-5p/*MACC1* Axis in Colorectal Cancer. DNA Cell Biol. 2019 Jun;38(6):572-582. doi: 10.1089/dna.2018.4548.
27. Liu D, Gao M, Wu K, Zhu D, Yang Y, Zhao S. LINC00152 facilitates tumorigenesis in esophageal squamous cell carcinoma via miR-153-3p/FYN axis. Biomed Pharmacother. 2019 Apr;112:108654. doi: 10.1016/j.biopha.2019.108654.
28. Zheng L, Hu N, Zhou X. TCF3-activated LINC00152 exerts oncogenic role in osteosarcoma through regulating miR-1182/CDK14 axis. Pathol Res Pract. 2019 Feb;215(2):373-380. doi: 10.1016/j.prp.2018.12.031.
29. Liang J, Wei X, Liu Z, Cao D, Tang Y, Zou Z, Zhou C, Lu Y. Long noncoding RNA CYTOR in cancer: A TCGA data review. Clin Chim Acta. 2018 Aug;483:227-233. doi: 10.1016/j.cca.2018.05.010.
30. Zhang J, Yin M, Huang J, Lv Z, Liang S, Miao X, Huang F, Zhao Y. Long noncoding RNA LINC00152 as a novel predictor of lymph node metastasis and survival in human cancer: a systematic review and meta-analysis. Clin Chim Acta. 2018 Aug;483:25-32. doi: 10.1016/j.cca.2018.03.034.
31. Wang X, Yu H, Sun W, Kong J, Zhang L, Tang J, Wang J, Xu E, Lai M, Zhang H. The long non-coding RNA CYTOR drives colorectal cancer progression by interacting with NCL and Sam68. Mol Cancer. 2018 Jul 31;17(1):110. doi: 10.1186/s12943-018-0860-7.
32. Yue B, Liu C, Sun H, Liu M, Song C, Cui R, Qiu S, Zhong M. A Positive Feed-Forward Loop between LncRNA-CYTOR and Wnt/β-Catenin Signaling Promotes Metastasis of Colon Cancer. Mol Ther. 2018 May 2;26(5):1287-1298. doi: 10.1016/j.ymthe.2018.02.024.
33. Bao J, Chen X, Hou Y, Kang G, Li Q, Xu Y. LncRNA DBH-AS1 facilitates the tumorigenesis of hepatocellular carcinoma by targeting miR-138 via FAK/Src/ERK pathway. Biomed Pharmacother. 2018 Nov;107:824-833. doi: 10.1016/j.biopha.2018.08.079.
34. Liu ZB, Wang JA, Lv RQ. Downregulation of long non-coding RNA DBH-AS1 inhibits osteosarcoma progression by PI3K-AKT signaling pathways and indicates good prognosis. Eur Rev Med Pharmacol Sci. 2019 Feb;23(4):1418-1427. doi: 10.26355/eurrev_201902_17098.
35. Huang JL, Ren TY, Cao SW, Zheng SH, Hu XM, Hu YW, Lin L, Chen J, Zheng L, Wang Q. HBx-related long non-coding RNA DBH-AS1 promotes cell proliferation and survival by activating MAPK signaling in hepatocellular carcinoma. Oncotarget. 2015 Oct 20;6(32):33791-804. doi: 10.18632/oncotarget.5667.
36. Yang Y, *et al*. Enhancer RNA-driven looping enhances the transcription of the long noncoding RNA DHRS4-AS1, a controller of the DHRS4 gene cluster. Sci Rep, 2016 Feb 11.
37. Li Q, *et al*. AS1DHRS4, a head-to-head natural antisense transcript, silences the DHRS4 gene cluster in cis and trans. Proc Natl Acad Sci U S A, 2012 Aug 28.
38. Luan F, Chen W, Chen M, Yan J, Chen H, Yu H, Liu T, Mo L. An autophagy-related long non-coding RNA signature for glioma. FEBS Open Bio. 2019 Mar 5;9(4):653-667. doi: 10.1002/2211-5463.12601.
39. Liu Y, Li J, Ma Z, Zhang J, Wang Y, Yu Z, Lin X, Xu Z, Su Q, An L, Zhou Y, Ma X, Yang Y, Wang F, Chen Q, Zhang Y, Wang J, Zheng H, Shi A, Yu S, Zhang J, Zhao W, Chen L. Oncogenic functions of protein kinase D2 and D3 in regulating multiple cancer-related pathways in breast cancer. Cancer Med. 2019 Feb;8(2):729-741. doi: 10.1002/cam4.1938.
40. Polev DE, Karnaukhova IK, Krukovskaya LL, Kozlov AP. ELFN1-AS1: a novel primate gene with possible microRNA function expressed predominantly in human tumors. Biomed Res Int. 2014;2014:398097. doi: 10.1155/2014/398097.
41. Liao M, Liao W, Xu N, Li B, Liu F, Zhang S, Wang Y, Wang S, Zhu Y, Chen D, Xie W, Jiang Y, Cao L, Yang BB, Zhang Y. LncRNA EPB41L4A-AS1 regulates glycolysis and glutaminolysis by mediating nucleolar translocation of HDAC2. EBioMedicine. 2019 Mar;41:200-213. doi: 10.1016/j.ebiom.2019.01.035.
42. Lu S, Dong W, Zhao P, Liu Z. lncRNA FAM83H-AS1 is associated with the prognosis of colorectal carcinoma and promotes cell proliferation by targeting the Notch signaling pathway. Oncol Lett. 2018 Feb;15(2):1861-1868. doi: 10.3892/ol.2017.7520.
43. Zhang J, Feng S, Su W, Bai S, Xiao L, Wang L, Thomas DG, Lin J, Reddy RM, Carrott PW, Lynch WR, Chang AC, Beer DG, Guo YM, Chen G. Overexpression of FAM83H-AS1 indicates poor patient survival and knockdown impairs cell proliferation and invasion via MET/EGFR signaling in lung cancer. Sci Rep. 2017 Feb 15;7:42819. doi: 10.1038/srep42819.
44. Yang F, Lv SX, Lv L, Liu YH, Dong SY, Yao ZH, Dai XX, Zhang XH, Wang OC. Identification of lncRNA *FAM83H-AS1* as a novel prognostic marker in luminal subtype breast cancer. Onco Targets Ther. 2016 Nov 15;9:7039-7045.
45. Dou Q, Xu Y, Zhu Y, Hu Y, Yan Y, Yan H. LncRNA FAM83H-AS1 contributes to the radioresistance, proliferation, and metastasis in ovarian cancer through stabilizing HuR protein. Eur J Pharmacol. 2019 Jun 5;852:134-141. doi: 10.1016/j.ejphar.2019.03.002.
46. Yu DJ, Li YH, Zhong M. LncRNA FBXL19-AS1 promotes proliferation and metastasis via regulating epithelial-mesenchymal transition in non-small cell lung cancer. Eur Rev Med Pharmacol Sci. 2019 Jun;23(11):4800-4806. doi: 10.26355/eurrev_201906_18065.
47. Shen B, Yuan Y, Zhang Y, Yu S, Peng W, Huang X, Feng J. Long non-coding RNA FBXL19-AS1 plays oncogenic role in colorectal cancer by sponging miR-203. Biochem Biophys Res Commun. 2017 Jun 17;488(1):67-73. doi: 10.1016/j.bbrc.2017.05.008.
48. Jiang Q, Cheng L, Ma D, Zhao Y. FBXL19-AS1 exerts oncogenic function by sponging miR-431-5p to regulate RAF1 expression in lung cancer. Biosci Rep. 2019 Jan 25;39(1). pii: BSR20181804. doi: 10.1042/BSR20181804.
49. Ding Z, Ye P, Yang X, Cai H. LncRNA FBXL19-AS1 promotes breast cancer cells proliferation and invasion via acting as a molecular sponge to miR-718. Biosci Rep. 2019 Apr 2;39(4). pii: BSR20182018. doi: 10.1042/BSR20182018.
50. Zhang Y, Xiao X, Zhou W, Hu J, Zhou D. LIN28A-stabilized FBXL19-AS1 promotes breast cancer migration, invasion and EMT by regulating WDR66. In Vitro Cell Dev Biol Anim. 2019 Jun;55(6):426-435. doi: 10.1007/s11626-019-00361-4.
51. Pan R, He Z, Ruan W, Li S, Chen H, Chen Z, Liu F, Tian X, Nie Y. lncRNA FBXL19-AS1 regulates osteosarcoma cell proliferation, migration and invasion by sponging miR-346. Onco Targets Ther. 2018 Nov 29;11:8409-8420. doi: 10.2147/OTT.S160963.
52. Liu Z, Shao Y, Tan L, Shi H, Chen S, Guo J. Clinical significance of the low expression of FER1L4 in gastric cancer patients. Tumour Biol. 2014 Oct;35(10):9613-7. doi: 10.1007/s13277-014-2259-4.
53. Sun X, Zheng G, Li C, Liu C. Long non-coding RNA Fer-1-like family member 4 suppresses hepatocellular carcinoma cell proliferation by regulating PTEN in vitro and in vivo. Mol Med Rep. 2019 Jan;19(1):685-692. doi: 10.3892/mmr.2018.9629.

54. Fei D, Zhang X, Liu J, Tan L, Xing J, Zhao D, Zhang Y. Long Noncoding RNA FER1L4 Suppresses Tumorigenesis by Regulating the Expression of PTEN Targeting miR-18a-5p in Osteosarcoma. Cell Physiol Biochem. 2018;51(3):1364-1375. doi: 10.1159/000495554.
55. Wu J, Huang J, Wang W, Xu J, Yin M, Cheng N, Yin J. Long non-coding RNA Fer-1-like protein 4 acts as a tumor suppressor via miR-106a-5p and predicts good prognosis in hepatocellular carcinoma. Cancer Biomark. 2017 Jul 19;20(1):55-65. doi: 10.3233/CBM-170090.
56. Yue B, Sun B, Liu C, Zhao S, Zhang D, Yu F, Yan D. Long non-coding RNA Fer-1-like protein 4 suppresses oncogenesis and exhibits prognostic value by associating with miR-106a-5p in colon cancer. Cancer Sci. 2015 Oct;106(10):1323-32. doi: 10.1111/cas.12759.
57. Li D, Jiang X, Zhang X, Cao G, Wang D, Chen Z.Long noncoding RNA FGD5-AS1 promotes colorectal cancer cell proliferation, migration, and invasion through upregulating CDCA7 via sponging miR-302e. In Vitro Cell Dev Biol Anim, 2019 Sep. PMID 31332696
58. Chen H, Lan Z, Li Q, Li Y.Abnormal expression of long noncoding RNA FGD5-AS1 affects the development of periodontitis through regulating miR-142-3p/SOCS6/NF-κB pathway. Artif Cells Nanomed Biotechnol, 2019 Dec.
59. Izuogu OG, Alhasan AA, Mellough C, Collin J, Gallon R, Hyslop J, Mastrorosa FK, Ehrmann I, Lako M, Elliott DJ, Santibanez-Koref M, Jackson MS. Analysis of human ES cell differentiation establishes that the dominant isoforms of the lncRNAs RMST and FIRRE are circular. BMC Genomics. 2018 Apr 20;19(1):276. doi: 10.1186/s12864-018-4660-7.
60. Lu Y, Liu X, Xie M, Liu M, Ye M, Li M, Chen XM, Li X, Zhou R. The NF-κB-Responsive Long Noncoding RNA FIRRE Regulates Posttranscriptional Regulation of Inflammatory Gene Expression through Interacting with hnRNPU. J Immunol. 2017 Nov 15;199(10):3571-3582. doi: 10.4049/jimmunol.1700091.
61. Yang F, Deng X, Ma W, Berletch JB, Rabaia N, Wei G, Moore JM, Filippova GN, Xu J, Liu Y, Noble WS, Shendure J, Disteche CM. The lncRNA Firre anchors the inactive X chromosome to the nucleolus by binding CTCF and maintains H3K27me3 methylation. Genome Biol. 2015 Mar 12;16:52. doi: 10.1186/s13059-015-0618-0.
62. Hacisuleyman E, Shukla CJ, Weiner CL, Rinn JL. Function and evolution of local repeats in the Firre locus. Nat Commun. 2016 Mar 24;7:11021. doi: 10.1038/ncomms11021.
63. Shi X, Cui Z, Liu X, Wu S, Wu Y, Fang F, Zhao H. LncRNA **FIRRE** is activated by MYC and promotes the development of diffuse large B-cell lymphoma via Wnt/β-catenin signaling pathway. Biochem Biophys Res Commun. 2019 Mar 19;510(4):594-600. doi: 10.1016/j.bbrc.2019.01.105.
64. Wang Y, Cao L, Wang Q, Huang J, Xu S. LncRNA FOXD2-AS1 induces chondrocyte proliferation through sponging miR-27a-3p in osteoarthritis. Artif Cells Nanomed Biotechnol. 2019 Dec;47(1):1241-1247. doi: 10.1080/21691401.2019.1596940.
65. Hu Q, Tai S, Wang J. Oncogenicity of lncRNA FOXD2-AS1 and its molecular mechanisms in human cancers. Pathol Res Pract. 2019 May;215(5):843-848. doi: 10.1016/j.prp.2019.01.033.
66. Jiang M, Qiu N, Xia H, Liang H, Li H, Ao X. Long non-coding RNA FOXD2-AS1/miR-150-5p/PFN2 axis regulates breast cancer malignancy and tumorigenesis. Int J Oncol. 2019 Mar;54(3):1043-1052. doi: 10.3892/ijo.2019.4671.
67. Dong H, Cao W, Xue J. Long noncoding FOXD2-AS1 is activated by CREB1 and promotes cell proliferation and metastasis in glioma by sponging miR-185 through targeting AKT1. Biochem Biophys Res Commun. 2019 Jan 22;508(4):1074-1081. doi: 10.1016/j.bbrc.2018.12.050.
68. Chang Y, Zhang J, Zhou C, Qiu G, Wang G, Wang S, Chang X, Li X, Fan L. Long non-coding RNA FOXD2-AS1 plays an oncogenic role in hepatocellular carcinoma by targeting miR-206. Oncol Rep. 2018 Dec;40(6):3625-3634. doi: 10.3892/or.2018.6752.
69. An Q, Zhou L, Xu N. Long noncoding RNA FOXD2-AS1 accelerates the gemcitabine-resistance of bladder cancer by sponging miR-143. Biomed Pharmacother. 2018 Jul;103:415-420. doi: 10.1016/j.biopha.2018.03.138.
70. Xu TP, Wang WY, Ma P, Shuai Y, Zhao K, Wang YF, Li W, Xia R, Chen WM, Zhang EB, Shu YQ. Upregulation of the long noncoding RNA FOXD2-AS1 promotes carcinogenesis by epigenetically silencing EphB3 through EZH2 and LSD1, and predicts poor prognosis in gastric cancer. Oncogene. 2018 Sep;37(36):5020-5036. doi: 10.1038/s41388-018-0308-y.
71. Zhu Y, Qiao L, Zhou Y, Ma N, Wang C, Zhou J. Long non-coding RNA FOXD2-AS1 contributes to colorectal cancer proliferation through its interaction with microRNA-185-5p. Cancer Sci. 2018 Jul;109(7):2235-2242. doi: 10.1111/cas.13632.
72. Chen G, Sun W, Hua X, Zeng W, Yang L. Long non-coding RNA FOXD2-AS1 aggravates nasopharyngeal carcinoma carcinogenesis by modulating miR-363-5p/S100A1 pathway. Gene. 2018 Mar 1;645:76-84. doi: 10.1016/j.gene.2017.12.026.
73. Yang X, Duan B, Zhou X. Long non-coding RNA FOXD2-AS1 functions as a tumor promoter in colorectal cancer by regulating EMT and Notch signaling pathway. Eur Rev Med Pharmacol Sci. 2017 Aug;21(16):3586-3591.
74. Rong L, Zhao R, Lu J. Highly expressed long non-coding RNA FOXD2-AS1 promotes non-small cell lung cancer progression via Wnt/β-catenin signaling. Biochem Biophys Res Commun. 2017 Mar 11;484(3):586-591. doi: 10.1016/j.bbrc.2017.01.141.
75. Oh JH, Yang JO, Hahn Y, Kim MR, Byun SS, Jeon YJ, Kim JM, Song KS, Noh SM, Kim S, Yoo HS, Kim YS, Kim NS. Transcriptome analysis of human gastric cancer. Mamm Genome. 2005 Dec;16(12):942-54.
76. Xie L, Gazin C, Park SM, Zhu LJ, Debily MA, Kittler EL, Zapp ML, Lapointe D, Gobeil S, Virbasius CM, Green MR. A synthetic interaction screen identifies factors selectively required for proliferation and TERT transcription in p53-deficient human cancer cells. PLoS Genet. 2012;8(12):e1003151. doi: 10.1371/journal.pgen.1003151.
77. Gu P, Chen X, Xie R, Han J, Xie W, Wang B, Dong W, Chen C, Yang M, Jiang J, Chen Z, Huang J, Lin T. lncRNA HOXD-AS1 Regulates Proliferation and Chemo-Resistance of Castration-Resistant Prostate Cancer via Recruiting WDR5. Mol Ther. 2017 Aug 2;25(8):1959-1973. doi: 10.1016/j.ymthe.2017.04.016.
78. Zhang Y, Dun Y, Zhou S, Huang XH. LncRNA HOXD-AS1 promotes epithelial ovarian cancer cells proliferation and invasion by targeting miR-133a-3p and activating Wnt/β-catenin signaling pathway. Biomed Pharmacother. 2017 Dec;96:1216-1221. doi: 10.1016/j.biopha.2017.11.096.
79. Chen Y, Zhao F, Cui D, Jiang R, Chen J, Huang Q, Shi J. HOXD-AS1/miR-130a sponge regulates glioma development by targeting E2F8. Int J Cancer. 2018 Jun 1;142(11):2313-2322. doi: 10.1002/ijc.31262.
80. Xia H, Jing H, Li Y, Lv X. Long noncoding RNA HOXD-AS1 promotes non-small cell lung cancer migration and invasion through regulating miR-133b/MMP9 axis. Biomed Pharmacother. 2018 Oct;106:156-162. doi: 10.1016/j.biopha.2018.06.073.
81. Gu W, Zhang E, Song L, Tu L, Wang Z, Tian F, Aikenmu K, Chu G, Zhao J. Long noncoding RNA HOXD-AS1 aggravates osteosarcoma carcinogenesis through epigenetically inhibiting p57 via EZH2. Biomed Pharmacother. 2018 Oct;106:890-895. doi: 10.1016/j.biopha.2018.06.173.
82. Yang MH, Zhao L, Wang L, Ou-Yang W, Hu SS, Li WL, Ai ML, Wang YQ, Han Y, Li TT, Ding YQ, Wang S. Nuclear lncRNA HOXD-AS1 suppresses colorectal carcinoma growth and metastasis via inhibiting HOXD3-induced integrin β3 transcriptional activating and MAPK/AKT signalling. Mol Cancer. 2019 Mar 1;18(1):31. doi: 10.1186/s12943-019-0955-9.
83. Dong S, Wang R, Wang H, Ding Q, Zhou X, Wang J, Zhang K, Long Y, Lu S, Hong T, Ren H, Wong K, Sheng X, Wang Y, Zeng Y. HOXD-AS1 promotes the epithelial to mesenchymal transition of ovarian cancer cells by regulating miR-186-5p and PIK3R3. J Exp Clin Cancer Res. 2019 Mar 1;38(1):110. doi: 10.1186/s13046-019-1103-5.

84. Huttlin EL, Bruckner RJ, Paulo JA, Cannon JR, Ting L, Baltier K, Colby G, Gebreab F, Gygi MP, Parzen H, Szpyt J, Tam S, Zarraga G, Pontano-Vaites L, Swarup S, White AE, Schweppe DK, Rad R, Erickson BK, Obar RA, Guruharsha KG, Li K, Artavanis-Tsakonas S, Gygi SP, Harper JW. Architecture of the human interactome defines protein communities and disease networks. Nature. 2017 May 25;545(7655):505-509. doi: 10.1038/nature22366.
85. Xiao Y, Yan X, Yang Y, Ma X. Downregulation of long noncoding RNA HOTAIRM1 variant 1 contributes to osteoarthritis via regulating miR-125b/BMPR2 axis and activating JNK/MAPK/ERK pathway. Biomed Pharmacother. 2019 Jan;109:1569-1577. doi: 10.1016/j.biopha.2018.10.181.
86. Li Q, Dong C, Cui J, Wang Y, Hong X. Over-expressed lncRNA HOTAIRM1 promotes tumor growth and invasion through up-regulating HOXA1 and sequestering G9a/EZH2/Dnmts away from the HOXA1 gene in glioblastoma multiforme. J Exp Clin Cancer Res. 2018 Oct 30;37(1):265. doi: 10.1186/s13046-018-0941-x.
87. Tian X, Ma J, Wang T, Tian J, Zhang Y, Mao L, Xu H, Wang S. Long Non-Coding RNA HOXA Transcript Antisense RNA Myeloid-Specific 1-HOXA1 Axis Downregulates the Immunosuppressive Activity of Myeloid-Derived Suppressor Cells in Lung Cancer. Front Immunol. 2018 Mar 12;9:473. doi: 10.3389/fimmu.2018.00473.
88. Wang XQ, Dostie J. Reciprocal regulation of chromatin state and architecture by HOTAIRM1 contributes to temporal collinear HOXA gene activation. Nucleic Acids Res. 2017 Feb 17;45(3):1091-1104. doi: 10.1093/nar/gkw966.
89. Peng F, Shi X, Meng Y, Dong B, Xu G, Hou T, Shi Y, Liu T. Long non-coding RNA HOTTIP is upregulated in renal cell carcinoma and regulates cell growth and apoptosis by epigenetically silencing of LATS2. Biomed Pharmacother. 2018 Sep;105:1133-1140. doi: 10.1016/j.biopha.2018.06.081.
90. Wang Q, Wu G, Zhang Z, Tang Q, Zheng W, Chen X, Chen F, Li Q, Che X. Long non-coding RNA HOTTIP promotes renal cell carcinoma progression through the regulation of the miR-615/IGF-2 pathway. Int J Oncol. 2018 Nov;53(5):2278-2288. doi: 10.3892/ijo.2018.4539.
91. Shen M, Li M, Liu J. Long Noncoding RNA HOTTIP Promotes Nasopharyngeal Cancer Cell Proliferation, Migration, and Invasion by Inhibiting miR-4301. Med Sci Monit. 2019 Jan 27;25:778-785. doi: 10.12659/MSM.912728.
92. Su Y, Lu J, Chen X, Liang C, Luo P, Qin C, Zhang J. Long non-coding RNA HOTTIP affects renal cell carcinoma progression by regulating autophagy via the PI3K/Akt/Atg13 signaling pathway. J Cancer Res Clin Oncol. 2019 Mar;145(3):573-588. doi: 10.1007/s00432-018-2808-0.
93. Xu C, He T, Li Z, Liu H, Ding B. Regulation of HOXA11-AS/miR-214-3p/EZH2 axis on the growth, migration and invasion of glioma cells. Biomed Pharmacother. 2017 Nov;95:1504-1513. doi: 10.1016/j.biopha.2017.08.097.
94. Yang JX, Liu B, Yang BY, Meng Q. Long non-coding RNA homeobox (HOX) A11-AS promotes malignant progression of glioma by targeting miR-124-3p. Neoplasma. 2018;65(4):505-514. doi: 10.4149/neo_2018_170705N462.
95. Nuermaimaiti N, Liu J, Liang X, Jiao Y, Zhang D, Liu L, Meng X, Guan Y. Effect of lncRNA HOXA11-AS1 on adipocyte differentiation in human adipose-derived stem cells. Biochem Biophys Res Commun. 2018 Jan 8;495(2):1878-1884. doi: 10.1016/j.bbrc.2017.12.006.
96. Zhao X, Li X, Zhou L, Ni J, Yan W, Ma R, Wu J, Feng J, Chen P. LncRNA HOXA11-AS drives cisplatin resistance of human LUAD cells via modulating miR-454-3p/Stat3. Cancer Sci. 2018 Oct;109(10):3068-3079. doi: 10.1111/cas.13764.
97. Gao Y, Yu H, Liu Y, Liu X, Zheng J, Ma J, Gong W, Chen J, Zhao L, Tian Y, Xue Y. Long Non-Coding RNA HOXA-AS2 Regulates Malignant Glioma Behaviors and Vasculogenic Mimicry Formation via the MiR-373/EGFR Axis. Cell Physiol Biochem. 2018;45(1):131-147. doi: 10.1159/000486253.
98. Zhang Y, Xu J, Zhang S, An J, Zhang J, Huang J, Jin Y. HOXA-AS2 Promotes Proliferation and Induces Epithelial-Mesenchymal Transition via the miR-520c-3p/GPC3 Axis in Hepatocellular Carcinoma. Cell Physiol Biochem. 2018;50(6):2124-2138. doi: 10.1159/000495056.
99. Dong X, Fang Z, Yu M, Zhang L, Xiao R, Li X, Pan G, Liu J. Knockdown of Long Noncoding RNA HOXA-AS2 Suppresses Chemoresistance of Acute Myeloid Leukemia via the miR-520c-3p/S100A4 Axis. Cell Physiol Biochem. 2018;51(2):886-896. doi: 10.1159/000495387.
100. Zhao Q, Zhao S, Li J, Zhang H, Qian C, Wang H, Liu J, Zhao Y. TCF7L2 activated HOXA-AS2 decreased the glucocorticoid sensitivity in acute lymphoblastic leukemia through regulating HOXA3/EGFR/Ras/Raf/MEK/ERK pathway. Biomed Pharmacother. 2019 Jan;109:1640-1649. doi: 10.1016/j.biopha.2018.10.046.
101. Wu F, Zhang C, Cai J, Yang F, Liang T, Yan X, Wang H, Wang W, Chen J, Jiang T. Upregulation of long noncoding RNA HOXA-AS3 promotes tumor progression and predicts poor prognosis in glioma. Oncotarget. 2017 May 24;8(32):53110-53123. doi: 10.18632/oncotarget.18162.
102. Tong Y, Wang M, Dai Y, Bao D, Zhang J, Pan H. LncRNA *HOXA-AS3* Sponges *miR-29c* to Facilitate Cell Proliferation, Metastasis, and EMT Process and Activate the MEK/ERK Signaling Pathway in Hepatocellular Carcinoma. Hum Gene Ther Clin Dev. 2019 Sep;30(3):129-141. doi: 10.1089/humc.2018.266.
103. Zhu X, Chen D, Liu Y, Yu J, Qiao L, Lin S, Chen D, Zhong G, Lu X, Wang Y, Wen J, Zhong X, Jiang Y. Long Noncoding RNA HOXA-AS3 Integrates NF-κB Signaling To Regulate Endothelium Inflammation. Mol Cell Biol. 2019 Sep 11;39(19). pii: e00139-19. doi: 10.1128/MCB.00139-19.
104. Zhu XX, Yan YW, Chen D, Ai CZ, Lu X, Xu SS, Jiang S, Zhong GS, Chen DB, Jiang YZ. Long non-coding RNA HoxA-AS3 interacts with EZH2 to regulate lineage commitment of mesenchymal stem cells. Oncotarget. 2016 Sep 27;7(39):63561-63570. doi: 10.18632/oncotarget.11538.
105. Huang HH, Chen FY, Chou WC, Hou HA, Ko BS, Lin CT, Tang JL, Li CC, Yao M, Tsay W, Hsu SC, Wu SJ, Chen CY, Huang SY, Tseng MH, Tien HF, Chen RH. Long non-coding RNA HOXB-AS3 promotes myeloid cell proliferation and its higher expression is an adverse prognostic marker in patients with acute myeloid leukemia and myelodysplastic syndrome. BMC Cancer. 2019 Jun 24;19(1):617. doi: 10.1186/s12885-019-5822-y.
106. Zhang XM, Chen H, Zhou B, Zhang QY, Liao Y, Wang JS, Wang ZH. lncRNA **HOXB-AS3** promotes hepatoma by inhibiting p53 expression. Eur Rev Med Pharmacol Sci. 2018 Oct;22(20):6784-6792. doi: 10.26355/eurrev_201810_16145.
107. Mukherjee K, Conway de Macario E, Macario AJ, Brocchieri L. Chaperonin genes on the rise: new divergent classes and intense duplication in human and other vertebrate genomes. BMC Evol Biol. 2010 Mar 1;10:64. doi: 10.1186/1471-2148-10-64.
108. Chen X, Liu S, Zhao X, Ma X, Gao G, Yu L, Yan D, Dong H, Sun W. Long noncoding RNA ILF3-AS1 promotes cell proliferation, migration, and invasion via negatively regulating miR-200b/a/429 in melanoma. Biosci Rep. 2017 Nov 6;37(6). pii: BSR20171031. doi: 10.1042/BSR20171031
109. Hu XH, Dai J, Shang HL, Zhao ZX, Hao YD. SP1-mediated upregulation of lncRNA ILF3-AS1 functions a ceRNA for miR-212 to contribute to osteosarcoma progression via modulation of SOX5. Biochem Biophys Res Commun. 2019 Apr 9;511(3):510-517. doi: 10.1016/j.bbrc.2019.02.110.
110. Gao G, Li W, Liu S, Han D, Yao X, Jin J, Han D, Sun W, Chen X. The positive feedback loop between ILF3 and lncRNA ILF3-AS1 promotes melanoma proliferation, migration, and invasion. Cancer Manag Res. 2018 Dec 11;10:6791-6802. doi: 10.2147/CMAR.S186777.

111. Shi B, Shao B, Yang C, Guo Y, Fu X, Gan N. Upregulation of JHDM1D-AS1 protects PDLSCs from H_2O_2-induced apoptosis by decreasing DNAJC10 via phosphorylation of eIF2α. Biochimie. 2019 Oct;165:48-56. doi: 10.1016/j.biochi.2019.06.018.
112. Kondo A, Nonaka A, Shimamura T, Yamamoto S, Yoshida T, Kodama T, Aburatani H, Osawa T. Long Noncoding RNA JHDM1D-AS1 Promotes Tumor Growth by Regulating Angiogenesis in Response to Nutrient Starvation. Mol Cell Biol. 2017 Aug 28;37(18). pii: e00125-17. doi: 10.1128/MCB.00125-17.
113. Feng W, Wang C, Liang C, Yang H, Chen D, Yu X, Zhao W, Geng D, Li S, Chen Z, Sun M. The Dysregulated Expression of KCNQ1OT1 and Its Interaction with Downstream Factors miR-145/CCNE2 in Breast Cancer Cells. Cell Physiol Biochem. 2018;49(2):432-446. doi: 10.1159/000492978.
114. Chen B, Ma J, Li C, Wang Y. Long noncoding RNA KCNQ1OT1 promotes proliferation and epithelial-mesenchymal transition by regulation of SMAD4 expression in lens epithelial cells. Mol Med Rep. 2018 Jul;18(1):16-24. doi: 10.3892/mmr.2018.8987.
115. Hu H, Yang L, Li L, Zeng C. Long non-coding RNA KCNQ1OT1 modulates oxaliplatin resistance in hepatocellular carcinoma through miR-7-5p/ ABCC1 axis. Biochem Biophys Res Commun. 2018 Sep 18;503(4):2400-2406. doi: 10.1016/j.bbrc.2018.06.168.
116. Wang CG, Liao Z, Xiao H, Liu H, Hu YH, Liao QD, Zhong D. LncRNA KCNQ1OT1 promoted BMP2 expression to regulate osteogenic differentiation by sponging miRNA-214. Exp Mol Pathol. 2019 Apr;107:77-84. doi: 10.1016/j.yexmp.2019.01.012.
117. Sun H, Li Y, Kong H, Dai S, Qian H. Dysregulation of KCNQ1OT1 promotes cholangiocarcinoma progression via miR-140-5p/SOX4 axis. Arch Biochem Biophys. 2018 Nov 15;658:7-15. doi: 10.1016/j.abb.2018.09.019.
118. Bian Y, Gao G, Zhang Q, Qian H, Yu L, Yao N, Qian J, Liu B, Qian X. KCNQ1OT1/miR-217/ZEB1 feedback loop facilitates cell migration and epithelial-mesenchymal transition in colorectal cancer. Cancer Biol Ther. 2019;20(6):886-896. doi: 10.1080/15384047.2019.1579959.
119. Jin X, Jin H, Shi Y, Guo Y, Zhang H. Long Non-Coding RNA KCNQ1OT1 Promotes Cataractogenesis via miR-214 and Activation of the Caspase-1 Pathway. Cell Physiol Biochem. 2017;42(1):295-305. doi: 10.1159/000477330.
120. Martin DD, Ladha S, Ehrnhoefer DE, Hayden MR. Autophagy in Huntington disease and huntingtin in autophagy. Trends Neurosci. 2015 Jan;38(1):26-35. doi: 10.1016/j.tins.2014.09.003.
121. Qin C, Chen J, Li J, Ju X, Zhang S, Cao Q, Han Z, Li P, Shao P, Wang M, Zhang Z, Gu M, Zhang W, Yin C. Variants in angiogenesis-related genes and the risk of clear cell renal cell carcinoma. Mutagenesis. 2014 Nov;29(6):419-25. doi: 10.1093/mutage/geu046.
122. Tang LX, Su SF, Wan Q, He P, Xhang Y, Cheng XM. Novel long non-coding RNA LBX2-AS1 indicates poor prognosis and promotes cell proliferation and metastasis through Notch signaling in non-small cell lung cancer. Eur Rev Med Pharmacol Sci. 2019 Sep;23(17):7419-7429. doi: 10.26355/eurrev_201909_18851.
123. Yang Y, Dai W, Sun Y, Zhao Z. Long non-coding RNA linc00239 promotes malignant behaviors and chemoresistance against doxorubicin partially via activation of the PI3K/Akt/mTOR pathway in acute myeloid leukaemia cells. Oncol Rep. 2019 Apr;41(4):2311-2320. doi: 10.3892/or.2019.6991.
124. Wang ZK, Yang L, Wu LL, Mao H, Zhou YH, Zhang PF, Dai GH. Long non-coding RNA LINC00261 sensitizes human colon cancer cells to cisplatin therapy. Braz J Med Biol Res. 2017 Dec 11;51(2):e6793. doi: 10.1590/1414-431X20176793.
125. Zhang HF, Li W, Han YD. LINC00261 suppresses cell proliferation, invasion and Notch signaling pathway in hepatocellular carcinoma. Cancer Biomark. 2018 Feb 14;21(3):575-582. doi: 10.3233/CBM-170471.
126. Fang Q, Sang L, Du S. Long noncoding RNA LINC00261 regulates endometrial carcinoma progression by modulating miRNA/FOXO1 expression. Cell Biochem Funct. 2018 Aug;36(6):323-330. doi: 10.1002/cbf.3352.
127. Swarr DT, Herriges M, Li S, Morley M, Fernandes S, Sridharan A, Zhou S, Garcia BA, Stewart K, Morrisey EE. The long noncoding RNA Falcor regulates Foxa2 expression to maintain lung epithelial homeostasis and promote regeneration. Genes Dev. 2019 Jun 1;33(11-12):656-668. doi: 10.1101/gad.320523.118.
128. Liao J, Dong LP. Linc00261 suppresses growth and metastasis of non-small cell lung cancer via repressing epithelial-mesenchymal transition. Eur Rev Med Pharmacol Sci. 2019 May;23(9):3829-3837. doi: 10.26355/eurrev_201905_17810.
129. Cheng D, Jiang S, Chen J, Li J, Ao L, Zhang Y. Upregulated long noncoding RNA Linc00261 in pre-eclampsia and its effect on trophoblast invasion and migration via regulating miR-558/TIMP4 signaling pathway. J Cell Biochem. 2019 Aug;120(8):13243-13253. doi: 10.1002/jcb.28598.
130. Yan D, Liu W, Liu Y, Luo M. LINC00261 suppresses human colon cancer progression via sponging miR-324-3p and inactivating the Wnt/β-catenin pathway. J Cell Physiol. 2019 Dec;234(12):22648-22656. doi: 10.1002/jcp.28831.
131. Shi J, Ma H, Wang H, Zhu W, Jiang S, Dou R, Yan B. Overexpression of LINC00261 inhibits non-small cell lung cancer cells progression by interacting with miR-522-3p and suppressing Wnt signaling. J Cell Biochem. 2019 Oct;120(10):18378-18387. doi: 10.1002/jcb.29149.
132. Lin K, Jiang H, Zhuang SS, Qin YS, Qiu GD, She YQ, Zheng JT, Chen C, Fang L, Zhang SY. Long noncoding RNA LINC00261 induces chemosensitization to 5-fluorouracil by mediating methylation-dependent repression of DPYD in human esophageal cancer. FASEB J. 2019 Feb;33(2):1972-1988. doi: 10.1096/fj.201800759R.
133. Shahabi S, Kumaran V, Castillo J, Cong Z, Nandagopal G, Mullen DJ, Alvarado A, Correa MR, Saizan A, Goel R, Bhat A, Lynch SK, Zhou B, Borok Z, Marconett CN. *LINC00261* Is an Epigenetically Regulated Tumor Suppressor Essential for Activation of the DNA Damage Response. Cancer Res. 2019 Jun 15;79(12):3050-3062. doi: 10.1158/0008-5472.CAN-18-2034.
134. Wang H, Sha L, Huang L, Yang S, Zhou Q, Luo X, Shi B. LINC00261 functions as a competing endogenous RNA to regulate BCL2L11 expression by sponging miR-132-3p in endometriosis. Am J Transl Res. 2019 Apr 15;11(4):2269-2279.
135. Atmadibrata B, Liu PY, Sokolowski N, Zhang L, Wong M, Tee AE, Marshall GM, Liu T. The novel long noncoding RNA linc00467 promotes cell survival but is down-regulated by N-Myc. PLoS One. 2014 Feb 19;9(2):e88112. doi: 10.1371/journal.pone.0088112.
136. Cai K, Li T, Guo L, Guo H, Zhu W, Yan L, Li F. Long non-coding RNA LINC00467 regulates hepatocellular carcinoma progression by modulating miR-9-5p/PPARA expression. Open Biol. 2019 Sep 27;9(9):190074. doi: 10.1098/rsob.190074.
137. Yang J, Liu Y, Mai X, Lu S, Jin L, Tai X. STAT1-induced upregulation of LINC00467 promotes the proliferation migration of lung adenocarcinoma cells by epigenetically silencing DKK1 to activate Wnt/β-catenin signaling pathway. Biochem Biophys Res Commun. 2019 Jun 18;514(1):118-126. doi: 10.1016/j.bbrc.2019.04.107.
138. Wang X, Liu H, Shen K, Pan X, Wei Y, Lv T, Song Y. Long intergenic non-coding RNA 00467 promotes lung adenocarcinoma proliferation, migration and invasion by binding with EZH2 and repressing HTRA3 expression. Mol Med Rep. 2019 Jul;20(1):640-654. doi: 10.3892/mmr.2019.10292.
139. Sun CC, Li SJ, Li G, Hua RX, Zhou XH, Li DJ. Long Intergenic Noncoding RNA 00511 Acts as an Oncogene in Non-small-cell Lung Cancer by Binding to EZH2 and Suppressing p57. Mol Ther Nucleic Acids. 2016 Nov 15;5(11):e385. doi: 10.1038/mtna.2016.94.
140. Zhao X, Liu Y, Li Z, Zheng S, Wang Z, Li W, Bi Z, Li L, Jiang Y, Luo Y, Lin Q, Fu Z, Rufu C. Linc00511 acts as a competing endogenous RNA to regulate VEGFA expression through sponging hsa-miR-29b-3p in pancreatic ductal adenocarcinoma. J Cell Mol Med. 2018 Jan;22(1):655-667. doi: 10.1111/jcmm.13351.

141. Ding J, Yang C, Yang S. LINC00511 interacts with miR-765 and modulates tongue squamous cell carcinoma progression by targeting LAMC2. J Oral Pathol Med. 2018 May;47(5):468-476. doi: 10.1111/jop.12677
142. Li J, Li Y, Meng F, Fu L, Kong C. Knockdown of long non-coding RNA linc00511 suppresses proliferation and promotes apoptosis of bladder cancer cells via suppressing Wnt/β-catenin signaling pathway. Biosci Rep. 2018 Aug 29;38(4). pii: BSR20171701. doi: 10.1042/BSR20171701.
143. Lu G, Li Y, Ma Y, Lu J, Chen Y, Jiang Q, Qin Q, Zhao L, Huang Q, Luo Z, Huang S, Wei Z. Long noncoding RNA LINC00511 contributes to breast cancer tumourigenesis and stemness by inducing the miR-185-3p/E2F1/Nanog axis. J Exp Clin Cancer Res. 2018 Nov 27;37(1):289. doi: 10.1186/s13046-018-0945-6.
144. Wang J, Tian Y, Zheng H, Ding Y, Wang X. An integrated analysis reveals the oncogenic function of lncRNA LINC00511 in human ovarian cancer. Cancer Med. 2019 Jun;8(6):3026-3035. doi: 10.1002/cam4.2171.
145. Zhang H, Zhao B, Wang X, Zhang F, Yu W. LINC00511 knockdown enhances paclitaxel cytotoxicity in breast cancer via regulating miR-29c/CDK6 axis. Life Sci. 2019 Jul 1;228:135-144. doi: 10.1016/j.lfs.2019.04.063.
146. Quan X, Zhao M, Yang X, Zhu Y, Tian X. AP2γ mediated downregulation of lncRNA LINC00511 as a ceRNA suppresses trophoblast invasion by regulating miR-29b-3p/Cyr61 axis. Biomed Pharmacother. 2019 Sep 19;120:109269. doi: 10.1016/j.biopha.2019.109269.
147. Tsai KW, Lo YH, Liu H, Yeh CY, Chen YZ, Hsu CW, Chen WS, Wang JH. Linc00659, a long noncoding RNA, acts as novel oncogene in regulating cancer cell growth in colorectal cancer. Mol Cancer. 2018 Mar 10;17(1):72. doi: 10.1186/s12943-018-0821-1.
148. Gong W, Su Y, Liu Y, Sun P, Wang X. Long non-coding RNA Linc00662 promotes cell invasion and contributes to cancer stem cell-like phenotypes in lung cancer cells. J Biochem. 2018 Dec 1;164(6):461-469. doi: 10.1093/jb/mvy078.
149. Liu Z, Yao Y, Huang S, Li L, Jiang B, Guo H, Lei W, Xiong J, Deng J. LINC00662 promotes gastric cancer cell growth by modulating the Hippo-YAP1 pathway. Biochem Biophys Res Commun. 2018 Nov 2;505(3):843-849. doi: 10.1016/j.bbrc.2018.09.191.
150. Liu Y, Gao X, Tian X. High expression of long intergenic non-coding RNA LINC00662 contributes to malignant growth of acute myeloid leukemia cells by upregulating ROCK1 via sponging microRNA-340-5p. Eur J Pharmacol. 2019 Sep 15;859:172535. doi: 10.1016/j.ejphar.2019.172535.
151. Wen DY, Lin P, Pang YY, Chen G, He Y, Dang YW, Yang H. Expression of the Long Intergenic Non-Protein Coding RNA 665 (LINC00665) Gene and the Cell Cycle in Hepatocellular Carcinoma Using The Cancer Genome Atlas, the Gene Expression Omnibus, and Quantitative Real-Time Polymerase Chain Reaction. Med Sci Monit. 2018 May 5;24:2786-2808. doi: 10.12659/MSM.907389.
152. Shan Y, Li P. Long Intergenic Non-Protein Coding RNA 665 Regulates Viability, Apoptosis, and Autophagy via the MiR-186-5p/MAP4K3 Axis in Hepatocellular Carcinoma. Yonsei Med J. 2019 Sep;60(9):842-853. doi: 10.3349/ymj.2019.60.9.842.
153. Liu X, Lu X, Zhen F, Jin S, Yu T, Zhu Q, Wang W, Xu K, Yao J, Guo R. LINC00665 Induces Acquired Resistance to Gefitinib through Recruiting EZH2 and Activating PI3K/AKT Pathway in NSCLC. Mol Ther Nucleic Acids. 2019 Jun 7;16:155-161. doi: 10.1016/j.omtn.2019.02.010.
154. Cong Z, Diao Y, Xu Y, Li X, Jiang Z, Shao C, Ji S, Shen Y, De W, Qiang Y. Long non-coding RNA linc00665 promotes lung adenocarcinoma progression and functions as ceRNA to regulate AKR1B10-ERK signaling by sponging miR-98. Cell Death Dis. 2019 Jan 28;10(2):84. doi: 10.1038/s41419-019-1361-3.
155. Zhou W, Liu T, Saren G, Liao L, Fang W, Zhao H. Comprehensive analysis of differentially expressed long non-coding RNAs in non-small cell lung cancer. Oncol Lett. 2019 Aug;18(2):1145-1156. doi: 10.3892/ol.2019.10414.
156. Chen Y, Bi F, An Y, Yang Q. Identification of pathological grade and prognosis-associated lncRNA for ovarian cancer. J Cell Biochem. 2019 Sep;120(9):14444-14454. doi: 10.1002/jcb.28704.
157. Wang D, Zheng J, Liu X, Xue Y, Liu L, Ma J, He Q, Li Z, Cai H, Liu Y. Knockdown of USF1 Inhibits the Vasculogenic Mimicry of Glioma Cells via Stimulating SNHG16/miR-212-3p and linc00667/miR-429 Axis. Mol Ther Nucleic Acids. 2019 Mar 1;14:465-482. doi: 10.1016/j.omtn.2018.12.017.
158. Chen W, Zhou ZQ, Ren YQ, Zhang L, Sun LN, Man YL, Wang ZK. Effects of long non-coding RNA LINC00667 on renal tubular epithelial cell proliferation, apoptosis and renal fibrosis via the miR-19b-3p/LINC00667/CTGF signaling pathway in chronic renal failure. Cell Signal. 2019 Feb;54:102-114. doi: 10.1016/j.cellsig.2018.10.016.
159. Kawasaki Y, Miyamoto M, Oda T, Matsumura K, Negishi L, Nakato R, Suda S, Yokota N, Shirahige K, Akiyama T. The novel lncRNA CALIC upregulates AXL to promote colon cancer metastasis. EMBO Rep. 2019 Aug;20(8):e47052. doi: 10.15252/embr.201847052.
160. Ziegler C, Graf J, Faderl S, Schedlbauer J, Strieder N, Förstl B, Spang R, Bruckmann A, Merkl R, Hombach S, Kretz M. The long non-coding RNA LINC00941 and SPRR5 are novel regulators of human epidermal homeostasis. EMBO Rep. 2019 Feb;20(2). pii: e46612. doi: 10.15252/embr.201846612.
161. Luo C, Tao Y, Zhang Y, Zhu Y, Minyao DN, Haleem M, Dong C, Zhang L, Zhang X, Zhao J, Liao Q. Regulatory network analysis of high expressed long non-coding RNA LINC00941 in gastric cancer. Gene. 2018 Jul 1;662:103-109. doi: 10.1016/j.gene.2018.04.023.
162. Wang L, Zhao H, Xu Y, Li J, Deng C, Deng Y, Bai J, Li X, Xiao Y, Zhang Y. Systematic identification of lincRNA-based prognostic biomarkers by integrating lincRNA expression and copy number variation in lung adenocarcinoma. Int J Cancer. 2019 Apr 1;144(7):1723-1734. doi: 10.1002/ijc.31865.
163. Yu T, Zhao Y, Hu Z, Li J, Chu D, Zhang J, Li Z, Chen B, Zhang X, Pan H, Li S, Lin H, Liu L, Yan M, He X, Yao M. MetaLnc9 Facilitates Lung Cancer Metastasis via a PGK1-Activated AKT/mTOR Pathway. Cancer Res. 2017 Nov 1;77(21):5782-5794. doi: 10.1158/0008-5472.CAN-17-0671.
164. Chen W, Zhang L, Zhou ZQ, Ren YQ, Sun LN, Man YL, Ma ZW, Wang ZK. Effects of Long Non-Coding RNA LINC00963 on Renal Interstitial Fibrosis and Oxidative Stress of Rats with Chronic Renal Failure via the Foxo Signaling Pathway. Cell Physiol Biochem. 2018;46(2):815-828. doi: 10.1159/000488739.
165. Jiao H, Jiang S, Wang H, Li Y, Zhang W. Upregulation of LINC00963 facilitates melanoma progression through miR-608/NACC1 pathway and predicts poor prognosis. Biochem Biophys Res Commun. 2018 Sep 26;504(1):34-39. doi: 10.1016/j.bbrc.2018.08.115
166. Zhou Y, Yin L, Li H, Liu LH, Xiao T. The LncRNA LINC00963 facilitates osteosarcoma proliferation and invasion by suppressing miR-204-3p/FN1 axis. Cancer Biol Ther. 2019;20(8):1141-1148. doi: 10.1080/15384047.2019.1598766.
167. Wang J, Li C, Xu L, Yang C, Zhang X. MiR-1193 was sponged by LINC00963 and inhibited cutaneous squamous cell carcinoma progression by targeting SOX4. Pathol Res Pract. 2019 Oct;215(10):152600. doi: 10.1016/j.prp.2019.152600.
168. Sakurai K, Reon BJ, Anaya J, Dutta A. The lncRNA DRAIC/PCAT29 Locus Constitutes a Tumor-Suppressive Nexus. Mol Cancer Res. 2015 May;13(5):828-38. doi: 10.1158/1541-7786.MCR-15-0016-T.
169. Tani H, Onuma Y, Ito Y, Torimura M. Long non-coding RNAs as surrogate indicators for chemical stress responses in human-induced pluripotent stem cells. PLoS One. 2014 Aug 29;9(8):e106282. doi: 10.1371/journal.pone.0106282.

170. Ntini E, Louloupi A, Liz J, Muino JM, Marsico A, Ørom UAV. Long ncRNA A-ROD activates its target gene DKK1 at its release from chromatin. Nat Commun. 2018 Apr 24;9(1):1636. doi: 10.1038/s41467-018-04100-3.
171. Marín-Béjar O, Marchese FP, Athie A, Sánchez Y, González J, Segura V, Huang L, Moreno I, Navarro A, Monzó M, García-Foncillas J, Rinn JL, Guo S, Huarte M. Pint lincRNA connects the p53 pathway with epigenetic silencing by the Polycomb repressive complex 2. Genome Biol. 2013;14(9):R104.
172. Zhang M, Zhao K, Xu X, Yang Y, Yan S, Wei P, Liu H, Xu J, Xiao F, Zhou H, Yang X, Huang N, Liu J, He K, Xie K, Zhang G, Huang S, Zhang N. A peptide encoded by circular form of LINC-PINT suppresses oncogenic transcriptional elongation in glioblastoma. Nat Commun. 2018 Oct 26;9(1):4475. doi: 10.1038/s41467-018-06862-2.
173. Liu W. LncRNA LINC-PINT Inhibits Cancer Cell Proliferation, Invasion, and Migration in Osteosarcoma by Downregulating miRNA-21. Cancer Biother Radiopharm. 2019 May;34(4):258-263. doi: 10.1089/cbr.2018.2684.
174. Zhang L, Hu J, Li J, Yang Q, Hao M, Bu L. Long noncoding RNA LINC-PINT inhibits non-small cell lung cancer progression through sponging miR-218-5p/PDCD4. Artif Cells Nanomed Biotechnol. 2019 Dec;47(1):1595-1602. doi: 10.1080/21691401.2019.1605371.
175. Zhu J, Gu H, Lv X, Yuan C, Ni P, Liu F. LINC-PINT Activates the Mitogen-Activated Protein Kinase Pathway to Promote Acute Myocardial Infarction by Regulating miR-208a-3p. Circ J. 2018 Oct 25;82(11):2783-2792. doi: 10.1253/circj.CJ-18-0396.
176. Huang Q, Zhang D, Diao Q, Lin M. lncRNA LINC-PINT is downregulated in melanoma and regulates cell proliferation by downregulating lncRNA BANCR. Oncol Lett. 2019 Sep;18(3):2917-2922. doi: 10.3892/ol.2019.10631.
177. Yuan Z, Xiu C, Liu D, Zhou G, Yang H, Pei R, Ding C, Cui X, Sun J, Song K. Long noncoding RNA LINC-PINT regulates laryngeal carcinoma cell stemness and chemoresistance through miR-425-5p/PTCH1/SHH axis. J Cell Physiol. 2019 Dec;234(12):23111-23122. doi: 10.1002/jcp.28874.
178. Zhang L, Chen J, Wang L, Chen L, Du Z, Zhu L, Cui M, Zhang M, Song L. Linc-PINT acted as a tumor suppressor by sponging miR-543 and miR-576-5p in esophageal cancer. J Cell Biochem. 2019 Aug 28. doi: 10.1002/jcb.28699. [Epub ahead of print]
179. Brandenberger R, Wei H, Zhang S, Lei S, Murage J, Fisk GJ, Li Y, Xu C, Fang R, Guegler K, Rao MS, Mandalam R, Lebkowski J, Stanton LW. Transcriptome characterization elucidates signaling networks that control human ES cell growth and differentiation. Nat Biotechnol. 2004 Jun;22(6):707-16.
180. Liu L, Yang X, Ni Q, Xiao Z, Zhao Y, Han J, Sun M, Chen B. Interaction between p12CDK2AP1 and a novel unnamed protein product inhibits cell proliferation by regulating the cell cycle. Mol Med Rep. 2014 Jan;9(1):156-62. doi: 10.3892/mmr.2013.1801.
181. Giannuzzi G, Siswara P, Malig M, Marques-Bonet T; NISC Comparative Sequencing Program, Mullikin JC, Ventura M, Eichler EE. Evolutionary dynamism of the primate LRRC37 gene family. Genome Res. 2013 Jan;23(1):46-59. doi: 10.1101/gr.138842.112.
182. Wang F, Tian X, Zhou J, Wang G, Yu W, Li Z, Fan Z, Zhang W, Liang A. A three-lncRNA signature for prognosis prediction of acute myeloid leukemia in patients. Mol Med Rep. 2018 Aug;18(2):1473-1484. doi: 10.3892/mmr.2018.9139.
183. Jeong G, Kwon DH, Shin S, Choe N, Ryu J, Lim YH, Kim J, Park WJ, Kook H, Kim YK. Long noncoding RNAs in vascular smooth muscle cells regulate vascular calcification. Sci Rep. 2019 Apr 10;9(1):5848. doi: 10.1038/s41598-019-42283-x.
184. May W, Korenberg JR, Chen XN, Lunsford L, Wood WJ, Thompson A, Wall R, Denny CT. Human lymphocyte-specific pp52 gene is a member of a highly conserved dispersed family. Genomics. 1993 Mar;15(3):515-20.
185. Comuzzie AG, Cole SA, Laston SL, Voruganti VS, Haack K, Gibbs RA, Butte NF. Novel genetic loci identified for the pathophysiology of childhood obesity in the Hispanic population. PLoS One. 2012;7(12):e51954. doi: 10.1371/journal.pone.0051954.
186. Cao Y, Wang P, Ning S, Xiao W, Xiao B, Li X. Identification of prognostic biomarkers in glioblastoma using a long non-coding RNA-mediated, competitive endogenous RNA network. Oncotarget. 2016 Jul 5;7(27):41737-41747. doi: 10.18632/oncotarget.9569.
187. Wang Y, Yang L, Chen T, Liu X, Guo Y, Zhu Q, Tong X, Yang W, Xu Q, Huang D, Tu K. A novel lncRNA MCM3AP-AS1 promotes the growth of hepatocellular carcinoma by targeting miR-194-5p/FOXA1 axis. Mol Cancer. 2019 Feb 19;18(1):28. doi: 10.1186/s12943-019-0957-7.
188. Zhang H, Luo C, Zhang G. LncRNA *MCM3AP-AS1* Regulates Epidermal Growth Factor Receptor and Autophagy to Promote Hepatocellular Carcinoma Metastasis by Interacting with *miR-455*. DNA Cell Biol. 2019 Aug;38(8):857-864. doi: 10.1089/dna.2019.4770.
189. Yang C, Zheng J, Xue Y, Yu H, Liu X, Ma J, Liu L, Wang P, Li Z, Cai H, Liu Y. The Effect of MCM3AP-AS1/miR-211/KLF5/AGGF1 Axis Regulating Glioblastoma Angiogenesis. Front Mol Neurosci. 2018 Jan 9;10:437. doi: 10.3389/fnmol.2017.00437
190. Liang M, Jia J, Chen L, Wei B, Guan Q, Ding Z, Yu J, Pang R, He G. LncRNA MCM3AP-AS1 promotes proliferation and invasion through regulating miR-211-5p/SPARC axis in papillary thyroid cancer. Endocrine. 2019 Aug;65(2):318-326. doi: 10.1007/s12020-019-01939-4.
191. Wang Y, Zhou J, Xu YJ, Hu HB. Long non-coding RNA LINC00968 acts as oncogene in NSCLC by activating the Wnt signaling pathway. J Cell Physiol. 2018 Apr;233(4):3397-3406. doi: 10.1002/jcp.26186.
192. Li X, Ren Y, Zuo T. Long noncoding RNA LINC00978 promotes cell proliferation and invasion in non-small cell lung cancer by inhibiting miR-6754-5p. Mol Med Rep. 2018 Nov;18(5):4725-4732. doi: 10.3892/mmr.2018.9463.
193. Bu JY, Lv WZ, Liao YF, Xiao XY, Lv BJ. Long non-coding RNA LINC00978 promotes cell proliferation and tumorigenesis via regulating microRNA-497/NTRK3 axis in gastric cancer. Int J Biol Macromol. 2019 Feb 15;123:1106-1114. doi: 10.1016/j.ijbiomac.2018.11.162.
194. Qian H, Chen L, Huang J, Wang X, Ma S, Cui F, Luo L, Ling L, Luo K, Zheng G. The lncRNA MIR4435-2HG promotes lung cancer progression by activating β-catenin signalling. J Mol Med (Berl). 2018 Aug;96(8):753-764. doi: 10.1007/s00109-018-1654-5.
195. Ouyang W, Ren L, Liu G, Chi X, Wei H. LncRNA MIR4435-2HG predicts poor prognosis in patients with colorectal cancer. PeerJ. 2019 Apr 2;7:e6683. doi: 10.7717/peerj.6683.
196. Wang H, Wu M, Lu Y, He K, Cai X, Yu X, Lu J, Teng L. LncRNA MIR4435-2HG targets desmoplakin and promotes growth and metastasis of gastric cancer by activating Wnt/β-catenin signaling. Aging (Albany NY). 2019 Sep 4;11(17):6657-6673. doi: 10.18632/aging.102164.
197. Li Z, Tan H, Zhao W, Xu Y, Zhang Z, Wang M, Zhou X. Integrative analysis of DNA methylation and gene expression profiles identifies MIR4435-2HG as an oncogenic lncRNA for glioma progression. Gene. 2019 Oct 5;715:144012. doi: 10.1016/j.gene.2019.144012.
198. Song J, Xu Q, Zhang H, Yin X, Zhu C, Zhao K, Zhu J. Five key lncRNAs considered as prognostic targets for predicting pancreatic ductal adenocarcinoma. J Cell Biochem. 2018 Jun;119(6):4559-4569. doi: 10.1002/jcb.26598.
199. Matsumura K, Kawasaki Y, Miyamoto M, Kamoshida Y, Nakamura J, Negishi L, Suda S, Akiyama T. The novel G-quadruplex-containing long non-coding RNA GSEC antagonizes DHX36 and modulates colon cancer cell migration. Oncogene. 2017 Mar 2;36(9):1191-1199. doi: 10.1038/onc.2016.282.

200. Lv Y, Li H, Li F, Liu P, Zhao X. Long Noncoding RNA MNX1-AS1 Knockdown Inhibits Cell Proliferation and Migration in Ovarian Cancer. Cancer Biother Radiopharm. 2017 Apr;32(3):91-99. doi: 10.1089/cbr.2017.2178.
201. Liu X, Yang Q, Yan J, Zhang X, Zheng M. LncRNA MNX1-AS1 promotes the progression of cervical cancer through activating MAPK pathway. J Cell Biochem. 2019 Mar;120(3):4268-4277. doi: 10.1002/jcb.27712.
202. Chu J, Li H, Xing Y, Jia J, Sheng J, Yang L, Sun K, Qu Y, Zhang Y, Yin H, Wan J, He F. LncRNA MNX1-AS1 promotes progression of esophageal squamous cell carcinoma by regulating miR-34a/SIRT1 axis. Biomed Pharmacother. 2019 Aug;116:109029. doi: 10.1016/j.biopha.2019.109029.
203. Ye Y, Gu B, Wang Y, Shen S, Huang W. E2F1-mediated MNX1-AS1-miR-218-5p-SEC61A1 feedback loop contributes to the progression of colon adenocarcinoma. J Cell Biochem. 2019 Apr;120(4):6145-6153. doi: 10.1002/jcb.27902.
204. Gao Y, Xu Y, Wang J, Yang X, Wen L, Feng J. lncRNA MNX1-AS1 Promotes Glioblastoma Progression Through Inhibition of miR-4443. Oncol Res. 2019 Feb 21;27(3):341-347. doi: 10.3727/096504018X15228909735079.
205. Ji D, Wang Y, Sun B, Yang J, Luo X. Long non-coding RNA MNX1-AS1 promotes hepatocellular carcinoma proliferation and invasion through targeting miR-218-5p/COMMD8 axis. Biochem Biophys Res Commun. 2019 Jun 4;513(3):669-674. doi: 10.1016/j.bbrc.2019.04.012.
206. Liu H, Han L, Liu Z, Gao N. Long noncoding RNA MNX1-AS1 contributes to lung cancer progression through the miR-527/BRF2 pathway. J Cell Physiol. 2019 Aug;234(8):13843-13850. doi: 10.1002/jcp.28064.
207. Hein MY, Hubner NC, Poser I, Cox J, Nagaraj N, Toyoda Y, Gak IA, Weisswange I, Mansfeld J, Buchholz F, Hyman AA, Mann M. A human interactome in three quantitative dimensions organized by stoichiometries and abundances. Cell. 2015 Oct 22;163(3):712-23. doi: 10.1016/j.cell.2015.09.053.
208. Sharma GG, So S, Gupta A, Kumar R, Cayrou C, Avvakumov N, Bhadra U, Pandita RK, Porteus MH, Chen DJ, Cote J, Pandita TK. MOF and histone H4 acetylation at lysine 16 are critical for DNA damage response and double-strand break repair. Mol Cell Biol. 2010 Jul;30(14):3582-95. doi: 10.1128/MCB.01476-09.
209. Neganova I, Vilella F, Atkinson SP, Lloret M, Passos JF, von Zglinicki T, O'Connor JE, Burks D, Jones R, Armstrong L, Lako M. An important role for CDK2 in G1 to S checkpoint activation and DNA damage response in human embryonic stem cells. Stem Cells. 2011 Apr;29(4):651-9. doi: 10.1002/stem.620.
210. Kugeratski FG, Atkinson SJ, Neilson LJ, Lilla S, Knight JRP, Serneels J, Juin A, Ismail S, Bryant DM, Markert EK, Machesky LM, Mazzone M, Sansom OJ, Zanivan S. Hypoxic cancer-associated fibroblasts increase NCBP2-AS2/HIAR to promote endothelial sprouting through enhanced VEGF signaling. Sci Signal. 2019 Feb 5;12(567). pii: eaan8247. doi: 10.1126/scisignal.aan8247.
211. Xu W, Deng B, Lin P, Liu C, Li B, Huang Q, Zhou H, Yang J, Qu L. Ribosome profiling analysis identified a KRAS-interacting microprotein that represses oncogenic signaling in hepatocellular carcinoma cells. Sci China Life Sci. 2019 Jun 24. doi: 10.1007/s11427-019-9580-5. [Epub ahead of print]
212. Zhou D, Xie M, He B, Gao Y, Yu Q, He B, Chen Q. Microarray data re-annotation reveals specific lncRNAs and their potential functions in non-small cell lung cancer subtypes. Mol Med Rep. 2017 Oct;16(4):5129-5136. doi: 10.3892/mmr.2017.7244.
213. Lu YB, Jiang Q, Yang MY, Zhou JX, Zhang Q. Long noncoding RNA NNT-AS1 promotes hepatocellular carcinoma progression and metastasis through miR-363/CDK6 axis. Oncotarget. 2017 Sep 28;8(51):88804-88814. doi: 10.18632/oncotarget.21321.
214. Hua F, Liu S, Zhu L, Ma N, Jiang S, Yang J. Highly expressed long non-coding RNA NNT-AS1 promotes cell proliferation and invasion through Wnt/β-catenin signaling pathway in cervical cancer. Biomed Pharmacother. 2017 Aug;92:1128-1134. doi: 10.1016/j.biopha.2017.03.057.
215. Li Y, Lv M, Song Z, Lou Z, Wang R, Zhuang M. Long non-coding RNA NNT-AS1 affects progression of breast cancer through miR-142-3p/ZEB1 axis. Biomed Pharmacother. 2018 Jul;103:939-946. doi: 10.1016/j.biopha.2018.04.087.
216. Shen Q, Jiang Y. LncRNA NNT-AS1 promotes the proliferation, and invasion of lung cancer cells via regulating miR-129-5p expression. Biomed Pharmacother. 2018 Sep;105:176-181. doi: 10.1016/j.biopha.2018.05.123.
217. Cai Y, Dong ZY, Wang JY. LncRNA NNT-AS1 is a major mediator of cisplatin chemoresistance in non-small cell lung cancer through MAPK/Slug pathway. Eur Rev Med Pharmacol Sci. 2018 Aug;22(15):4879-4887. doi: 10.26355/eurrev_201808_15624.
218. Chen B, Zhao Q, Guan L, Lv H, Bie L, Huang J, Chen XB. Long non-coding RNA NNT-AS1 sponges miR-424/E2F1 to promote the tumorigenesis and cell cycle progression of gastric cancer. J Cell Mol Med. 2018 Oct;22(10):4751-4759. doi: 10.1111/jcmm.13726.
219. Li C, Zhang S, Qiu T, Wang Y, Ricketts DM, Qi C. Upregulation of long non-coding RNA NNT-AS1 promotes osteosarcoma progression by inhibiting the tumor suppressive miR-320a. Cancer Biol Ther. 2019;20(4):413-422. doi: 10.1080/15384047.2018.1538612.
220. Goyal A, Fiškin E, Gutschner T, Polycarpou-Schwarz M, Groß M, Neugebauer J, Gandhi M, Caudron-Herger M, Benes V, Diederichs S. A cautionary tale of sense-antisense gene pairs: independent regulation despite inverse correlation of expression. Nucleic Acids Res. 2017 Dec 1;45(21):12496-12508. doi: 10.1093/nar/gkx952.
221. Lee S, Kopp F, Chang TC, Sataluri A, Chen B, Sivakumar S, Yu H, Xie Y, Mendell JT. Noncoding RNA NORAD Regulates Genomic Stability by Sequestering PUMILIO Proteins. Cell. 2016 Jan 14;164(1-2):69-80. doi: 10.1016/j.cell.2015.12.017.
222. Bao MH, Li GY, Huang XS, Tang L, Dong LP, Li JM. Long Noncoding RNA LINC00657 Acting as a miR-590-3p Sponge to Facilitate Low Concentration Oxidized Low-Density Lipoprotein-Induced Angiogenesis. Mol Pharmacol. 2018 Apr;93(4):368-375. doi: 10.1124/mol.117.110650.
223. Kawasaki N, Miwa T, Hokari S, Sakurai T, Ohmori K, Miyauchi K, Miyazono K, Koinuma D. Long noncoding RNA NORAD regulates transforming growth factor-β signaling and epithelial-to-mesenchymal transition-like phenotype. Cancer Sci. 2018 Jul;109(7):2211-2220. doi: 10.1111/cas.13626.
224. Sur S, Sasaki R, Devhare P, Steele R, Ray R, Ray RB. Association between MicroRNA-373 and Long Noncoding RNA NORAD in Hepatitis C Virus-Infected Hepatocytes Impairs Wee1 Expression for Growth Promotion. J Virol. 2018 Sep 26;92(20). pii: e01215-18. doi: 10.1128/JVI.01215-18.
225. Huo H, Tian J, Wang R, Li Y, Qu C, Wang N. Long non-coding RNA NORAD upregulate SIP1 expression to promote cell proliferation and invasion in cervical cancer. Biomed Pharmacother. 2018 Oct;106:1454-1460. doi: 10.1016/j.biopha.2018.07.101.
226. Miao Z, Guo X, Tian L. The long noncoding RNA NORAD promotes the growth of gastric cancer cells by sponging miR-608. Gene. 2019 Mar 1;687:116-124. doi: 10.1016/j.gene.2018.11.052.
227. Gao W, Weng T, Wang L, Shi B, Meng W, Wang X, Wu Y, Jin L, Fei L. Long non-coding RNA NORAD promotes cell proliferation and glycolysis in non-small cell lung cancer by acting as a sponge for miR-136-5p. Mol Med Rep. 2019 Jun;19(6):5397-5405. doi: 10.3892/mmr.2019.10210.
228. Sun Y, Wang J, Pan S, Yang T, Sun X, Wang Y, Shi X, Zhao X, Guo J, Zhang X. LINC00657 played oncogenic roles in esophageal squamous cell carcinoma by targeting miR-615-3p and JunB. Biomed Pharmacother. 2018 Dec;108:316-324. doi: 10.1016/j.biopha.2018.09.003. Epub 2018 Sep 15.
229. Tichon A, Perry RB, Stojic L, Ulitsky I. SAM68 is required for regulation of Pumilio by the NORAD long noncoding RNA. Genes Dev. 2018 Jan 1;32(1):70-78. doi: 10.1101/gad.309138.117.

230. Li H, Wang X, Wen C, Huo Z, Wang W, Zhan Q, Cheng D, Chen H, Deng X, Peng C, Shen B. Long noncoding RNA NORAD, a novel competing endogenous RNA, enhances the hypoxia-induced epithelial-mesenchymal transition to promote metastasis in pancreatic cancer. Mol Cancer. 2017 Nov 9;16(1):169. doi: 10.1186/s12943-017-0738-0.
231. Tichon A, Gil N, Lubelsky Y, Havkin Solomon T, Lemze D, Itzkovitz S, Stern-Ginossar N, Ulitsky I. A conserved abundant cytoplasmic long noncoding RNA modulates repression by Pumilio proteins in human cells. Nat Commun. 2016 Jul 13;7:12209. doi: 10.1038/ncomms12209.
232. Oláh J, Vincze O, Virók D, Simon D, Bozsó Z, Tõkési N, Horváth I, Hlavanda E, Kovács J, Magyar A, Szũcs M, Orosz F, Penke B, Ovádi J. Interactions of pathological hallmark proteins: tubulin polymerization promoting protein/p25, beta-amyloid, and alpha-synuclein. J Biol Chem. 2011 Sep 30;286(39):34088-100. doi: 10.1074/jbc.M111.243907.
233. Kim J, Abdelmohsen K, Yang X, De S, Grammatikakis I, Noh JH, Gorospe M. LncRNA OIP5-AS1/cyrano sponges RNA-binding protein HuR. Nucleic Acids Res. 2016 Mar 18;44(5):2378-92. doi: 10.1093/nar/gkw017.
234. Smith KN, Starmer J, Miller SC, Sethupathy P, Magnuson T. Long Noncoding RNA Moderates MicroRNA Activity to Maintain Self-Renewal in Embryonic Stem Cells. Stem Cell Reports. 2017 Jul 11;9(1):108-121. doi: 10.1016/j.stemcr.2017.05.005.
235. Liu X, Zheng J, Xue Y, Yu H, Gong W, Wang P, Li Z, Liu Y. PIWIL3/OIP5-AS1/miR-367-3p/CEBPA feedback loop regulates the biological behavior of glioma cells. Theranostics. 2018 Jan 1;8(4):1084-1105. doi: 10.7150/thno.21740.
236. Deng J, Deng H, Liu C, Liang Y, Wang S. Long non-coding RNA OIP5-AS1 functions as an oncogene in lung adenocarcinoma through targeting miR-448/Bcl-2. Biomed Pharmacother. 2018 Feb;98:102-110. doi: 10.1016/j.biopha.2017.12.031.
237. Wang M, Sun X, Yang Y, Jiao W. Long non-coding RNA OIP5-AS1 promotes proliferation of lung cancer cells and leads to poor prognosis by targeting miR-378a-3p. Thorac Cancer. 2018 Aug;9(8):939-949. doi: 10.1111/1759-7714.12767.
238. Dai J, Xu L, Hu X, Han G, Jiang H, Sun H, Zhu G, Tang X. Long noncoding RNA OIP5-AS1 accelerates CDK14 expression to promote osteosarcoma tumorigenesis via targeting miR-223. Biomed Pharmacother. 2018 Oct;106:1441-1447. doi: 10.1016/j.biopha.2018.07.109.
239. Sun WL, Kang T, Wang YY, Sun JP, Li C, Liu HJ, Yang Y, Jiao BH. Long noncoding RNA OIP5-AS1 targets Wnt-7b to affect glioma progression via modulation of miR-410. Biosci Rep. 2019 Jan 3;39(1). pii: BSR20180395. doi: 10.1042/BSR20180395.
240. Zeng H, Wang J, Chen T, Zhang K, Chen J, Wang L, Li H, Tuluhong D, Li J, Wang S. Downregulation of long non-coding RNA Opa interacting protein 5-antisense RNA 1 inhibits breast cancer progression by targeting sex-determining region Y-box 2 by microRNA-129-5p upregulation. Cancer Sci. 2019 Jan;110(1):289-302. doi: 10.1111/cas.13879.
241. Mills JD, Kavanagh T, Kim WS, Chen BJ, Waters PD, Halliday GM, Janitz M. High expression of long intervening non-coding RNA OLMALINC in the human cortical white matter is associated with regulation of oligodendrocyte maturation. Mol Brain. 2015 Jan 10;8:2. doi: 10.1186/s13041-014-0091-9.
242. Takata M, Pachera E, Frank-Bertoncelj M, Kozlova A, Jüngel A, Whitfield ML, Assassi S, Calcagni M, de Vries-Bouwstra J, Huizinga TW, Kurreeman F, Kania G, Distler O. OTUD6B-AS1 Might Be a Novel Regulator of Apoptosis in Systemic Sclerosis. Front Immunol. 2019 May 17;10:1100. doi: 10.3389/fimmu.2019.01100.
243. Wang G, Zhang ZJ, Jian WG, Liu PH, Xue W, Wang TD, Meng YY, Yuan C, Li HM, Yu YP, Liu ZX, Wu Q, Zhang DM, Zhang C. Novel long noncoding RNA OTUD6B-AS1 indicates poor prognosis and inhibits clear cell renal cell carcinoma proliferation via the Wnt/β-catenin signaling pathway. Mol Cancer. 2019 Jan 22;18(1):15. doi: 10.1186/s12943-019-0942-1.
244. Zhang Y, Li F, Chen J. MYC promotes the development of papillary thyroid carcinoma by inhibiting the expression of lncRNA PAX8-AS1:28. Oncol Rep. 2019 Apr;41(4):2511-2517. doi: 10.3892/or.2019.6996.
245. Han J, Zhou W, Jia M, Wen J, Jiang J, Shi J, Zhang K, Ma H, Liu J, Ren J, Dai M, Hu Z, Hang D, Li N, Shen H. Expression quantitative trait loci in long non-coding RNA PAX8-AS1 are associated with decreased risk of cervical cancer. Mol Genet Genomics. 2016 Aug;291(4):1743-8. doi: 10.1007/s00438-016-1217-9.
246. Bahari G, Hashemi M, Naderi M, Sadeghi-Bojd S, Taheri M. Long non-coding RNA PAX8-AS1 polymorphisms increase the risk of childhood acute lymphoblastic leukemia. Biomed Rep. 2018 Feb;8(2):184-190. doi: 10.3892/br.2017.1028.
247. Yu X, Cao Y, Tang L, Yang Y, Chen F, Xia J. Baicalein inhibits breast cancer growth via activating a novel isoform of the long noncoding RNA PAX8-AS1-N. J Cell Biochem. 2018 Aug;119(8):6842-6856. doi: 10.1002/jcb.26881.
248. Mo XB, Wu LF, Zhu XW, Xia W, Wang L, He P, Bing PF, Lu X, Zhang YH, Deng FY, Lei SF. Identification and evaluation of lncRNA and mRNA integrative modules in human peripheral blood mononuclear cells. Epigenomics. 2017 Jul;9(7):943-954. doi: 10.2217/epi-2016-0178.
249. Choudhury NR, Heikel G, Trubitsyna M, Kubik P, Nowak JS, Webb S, Granneman S, Spanos C, Rappsilber J, Castello A, Michlewski G. RNA-binding activity of TRIM25 is mediated by its PRY/SPRY domain and is required for ubiquitination. BMC Biol. 2017 Nov 8;15(1):105. doi: 10.1186/s12915-017-0444-9.
250. Xu S, Kong D, Chen Q, Ping Y, Pang D. Oncogenic long noncoding RNA landscape in breast cancer. Mol Cancer. 2017 Jul 24;16(1):129. doi: 10.1186/s12943-017-0696-6.
251. DIAbetes Genetics Replication And Meta-analysis (DIAGRAM) Consortium; Asian Genetic Epidemiology Network Type 2 Diabetes (AGEN-T2D) Consortium; South Asian Type 2 Diabetes (SAT2D) Consortium; Mexican American Type 2 Diabetes (MAT2D) Consortium; Type 2 Diabetes Genetic Exploration by Nex-generation sequencing in muylti-Ethnic Samples (T2D-GENES) Consortium, Mahajan A, Go MJ, Zhang W, Below JE, Gaulton KJ, Ferreira T, et al. Genome-wide trans-ancestry meta-analysis provides insight into the genetic architecture of type 2 diabetes susceptibility. Nat Genet. 2014 Mar;46(3):234-44. doi: 10.1038/ng.2897.
252. Jiang W, Meng K, Yang T. Long non-coding RNA PROX1-AS1 promotes the proliferation and migration in gastric cancer by epigenetically activating FGFR1. Panminerva Med. 2019 Jul 29. doi: 10.23736/S0031-0808.19.03709-1.
253. Xu H, Han H, Song S, Yi N, Qian C, Qiu Y, Zhou W, Hong Y, Zhuang W, Li Z, Li B, Zhuang W. Exosome-Transmitted *PSMA3* and *PSMA3-AS1* Promote Proteasome Inhibitor Resistance in Multiple Myeloma. Clin Cancer Res. 2019 Mar 15;25(6):1923-1935. doi: 10.1158/1078-0432.CCR-18-2363.
254. Xiao M, Feng Y, Liu C, Zhang Z. Prognostic values of long noncoding RNA PVT1 in various carcinomas: An updated systematic review and meta-analysis. Cell Prolif. 2018 Dec;51(6):e12519. doi: 10.1111/cpr.12519.
255. He F, Song Z, Chen H, Chen Z, Yang P, Li W, Yang Z, Zhang T, Wang F, Wei J, Wei F, Wang Q, Cao J. Long noncoding RNA PVT1-214 promotes proliferation and invasion of colorectal cancer by stabilizing Lin28 and interacting with miR-128. Oncogene. 2019 Jan;38(2):164-179. doi: 10.1038/s41388-018-0432-8.
256. Alessio E, Buson L, Chemello F, Peggion C, Grespi F, Martini P, Massimino ML, Pacchioni B, Millino C, Romualdi C, Bertoli A, Scorrano L, Lanfranchi G, Cagnin S. Single cell analysis reveals the involvement of the long non-coding RNA Pvt1 in the modulation of muscle atrophy and mitochondrial network. Nucleic Acids Res. 2019 Feb 28;47(4):1653-1670. doi: 10.1093/nar/gkz007.
257. Sun ZY, Jian YK, Zhu HY, Li B. lncRNAPVT1 targets miR-152 to enhance chemoresistance of osteosarcoma to gemcitabine through activating c-MET/PI3K/AKT pathway. Pathol Res Pract. 2019 Mar;215(3):555-563. doi: 10.1016/j.prp.2018.12.013.

258. Qin S, Zhao Y, Lim G, Lin H, Zhang X, Zhang X. Circular RNA PVT1 acts as a competing endogenous RNA for miR-497 in promoting non-small cell lung cancer progression. Biomed Pharmacother. 2019 Mar;111:244-250. doi: 10.1016/j.biopha.2018.12.007.
259. Chen J, Yu Y, Li H, Hu Q, Chen X, He Y, Xue C, Ren F, Ren Z, Li J, Liu L, Duan Z, Cui G, Sun R. Long non-coding RNA PVT1 promotes tumor progression by regulating the miR-143/HK2 axis in gallbladder cancer. Mol Cancer. 2019 Mar 2;18(1):33. doi: 10.1186/s12943-019-0947-9.
260. Yang L, Peng X, Jin H, Liu J. Long non-coding RNA PVT1 promotes autophagy as ceRNA to target ATG3 by sponging Gene. 2019 May 20;697:94-102. doi: 10.1016/j.gene.2019.02.036.microRNA-365 in hepatocellular carcinoma.
261. Xu MD, Wang Y, Weng W, Wei P, Qi P, Zhang Q, Tan C, Ni SJ, Dong L, Yang Y, Lin W, Xu Q, Huang D, Huang Z, Ma Y, Zhang W, Sheng W, Du X. A Positive Feedback Loop of lncRNA-*PVT1* and FOXM1 Facilitates Gastric Cancer Growth and Invasion. Clin Cancer Res. 2017 Apr 15;23(8):2071-2080. doi: 10.1158/1078-0432.CCR-16-0742.
262. Zhao L, Kong H, Sun H, Chen Z, Chen B, Zhou M. LncRNA-PVT1 promotes pancreatic cancer cells proliferation and migration through acting as a molecular sponge to regulate miR-448. J Cell Physiol. 2018 May;233(5):4044-4055. doi: 10.1002/jcp.26072.
263. Kong R, Zhang EB, Yin DD, You LH, Xu TP, Chen WM, Xia R, Wan L, Sun M, Wang ZX, De W, Zhang ZH. Long noncoding RNA PVT1 indicates a poor prognosis of gastric cancer and promotes cell proliferation through epigenetically regulating p15 and p16. Mol Cancer. 2015 Apr 12;14:82. doi: 10.1186/s12943-015-0355-8.
264. Xue W, Chen J, Liu X, Gong W, Zheng J, Guo X, Liu Y, Liu L, Ma J, Wang P, Li Z, Xue Y. PVT1 regulates the malignant behaviors of human glioma cells by targeting miR-190a-5p and miR-488-3p. Biochim Biophys Acta Mol Basis Dis. 2018 May;1864(5 Pt A):1783-1794. doi: 10.1016/j.bbadis.2018.02.022.
265. Zhao Y, Zhao J, Guo X, She J, Liu Y. Long non-coding RNA PVT1, a molecular sponge for miR-149, contributes aberrant metabolic dysfunction and inflammation in IL-1β-simulated osteoarthritic chondrocytes. Biosci Rep. 2018 Sep 28;38(5). pii: BSR20180576. doi: 10.1042/BSR20180576.
266. Cho SW, Xu J, Sun R, Mumbach MR, Carter AC, Chen YG, Yost KE, Kim J, He J, Nevins SA, Chin SF, Caldas C, Liu SJ, Horlbeck MA, Lim DA, Weissman JS, Curtis C, Chang HY. Promoter of lncRNA Gene PVT1 Is a Tumor-Suppressor DNA Boundary Element. Cell. 2018 May 31;173(6):1398-1412.e22. doi: 10.1016/j.cell.2018.03.068.
267. Chen D, Li Z, Yang Q, Zhang J, Zhai Z, Shu HB. Identification of a nuclear protein that promotes NF-kappaB activation. Biochem Biophys Res Commun. 2003 Oct 24;310(3):720-4.
268. Covarrubias S, Robinson EK, Shapleigh B, Vollmers A, Katzman S, Hanley N, Fong N, McManus MT, Carpenter S. CRISPR/Cas-based screening of long non-coding RNAs (lncRNAs) in macrophages with an NF-κB reporter. J Biol Chem. 2017 Dec 22;292(51):20911-20920. doi: 10.1074/jbc.M117.799155.
269. Jones RM, Cadby G, Melton PE, Abraham LJ, Whitehouse AJ, Moses EK. Genome-wide association study of autistic-like traits in a general population study of young adults. Front Hum Neurosci. 2013 Oct 11;7:658. doi: 10.3389/fnhum.2013.00658.
270. Maréchal A, Li JM, Ji XY, Wu CS, Yazinski SA, Nguyen HD, Liu S, Jiménez AE, Jin J, Zou L. PRP19 transforms into a sensor of RPA-ssDNA after DNA damage and drives ATR activation via a ubiquitin-mediated circuitry. Mol Cell. 2014 Jan 23;53(2):235-246. doi: 10.1016/j.molcel.2013.11.002
271. Maréchal A, Zou L. RPA-coated single-stranded DNA as a platform for post-translational modifications in the DNA damage response. Cell Res. 2015 Jan;25(1):9-23. doi: 10.1038/cr.2014.147.
272. Balasubramanian S, Zheng D, Liu YJ, Fang G, Frankish A, Carriero N, Robilotto R, Cayting P, Gerstein M. Comparative analysis of processed ribosomal protein pseudogenes in four mammalian genomes. Genome Biol. 2009;10(1):R2. doi: 10.1186/gb-2009-10-1-r2.
273. Fei T, Chen Y, Xiao T, Li W, Cato L, Zhang P, Cotter MB, Bowden M, Lis RT, Zhao SG, Wu Q, Feng FY, Loda M, He HH, Liu XS, Brown M. Genome-wide CRISPR screen identifies HNRNPL as a prostate cancer dependency regulating RNA splicing. Proc Natl Acad Sci U S A. 2017 Jun 27;114(26):E5207-E5215. doi: 10.1073/pnas.1617467114.
274. Saez I, Koyuncu S, Gutierrez-Garcia R, Dieterich C, Vilchez D. Insights into the ubiquitin-proteasome system of human embryonic stem cells. Sci Rep. 2018 Mar 6;8(1):4092. doi: 10.1038/s41598-018-22384-9.
275. Shi D, Liang L, Zheng H, Cai G, Li X, Xu Y, Cai S. Silencing of long non-coding RNA SBDSP1 suppresses tumor growth and invasion in colorectal cancer. Biomed Pharmacother. 2017 Jan;85:355-361. doi: 10.1016/j.biopha.2016.11.036.
276. Banks CA, Boanca G, Lee ZT, Eubanks CG, Hattem GL, Peak A, Weems LE, Conkright JJ, Florens L, Washburn MP. TNIP2 is a Hub Protein in the NF-κB Network with Both Protein and RNA Mediated Interactions. Mol Cell Proteomics. 2016 Nov;15(11):3435-3449.
277. Boocock GR, Morrison JA, Popovic M, Richards N, Ellis L, Durie PR, Rommens JM. Mutations in SBDS are associated with Shwachman-Diamond syndrome. Nat Genet. 2003 Jan;33(1):97-101.
278. Liu Y, Liu F, Cao Y, Xu H, Wu Y, Wu S, Liu D, Zhao Y, Songyang Z, Ma W. Shwachman-Diamond Syndrome Protein SBDS Maintains Human Telomeres by Regulating Telomerase Recruitment. Cell Rep. 2018 Feb 13;22(7):1849-1860. doi: 10.1016/j.celrep.2018.01.057.
279. Warren AJ. Molecular basis of the human ribosomopathy Shwachman-Diamond syndrome. Adv Biol Regul. 2018 Jan;67:109-127. doi: 10.1016/j.jbior.2017.09.002.
280. Bezzerri V, Cipolli M. Shwachman-Diamond Syndrome: Molecular Mechanisms and Current Perspectives. Mol Diagn Ther. 2019 Apr;23(2):281-290. doi: 10.1007/s40291-018-0368-2.
281. Yue Y, Liu J, Cui X, Cao J, Luo G, Zhang Z, Cheng T, Gao M, Shu X, Ma H, Wang F, Wang X, Shen B, Wang Y, Feng X, He C, Liu J. VIRMA mediates preferential m_6A mRNA methylation in 3'UTR and near stop codon and associates with alternative polyadenylation. Cell Discov. 2018 Feb 27;4:10. doi: 10.1038/s41421-018-0019-0.
282. Li Y, Huang S, Li Y, Zhang W, He K, Zhao M, Lin H, Li D, Zhang H, Zheng Z, Huang C. Decreased expression of LncRNA SLC25A25-AS1 promotes proliferation, chemoresistance, and EMT in colorectal cancer cells. Tumour Biol. 2016 Oct;37(10):14205-14215
283. Guo W, Huang J, Lei P, Guo L, Li X. LncRNA SNHG1 promoted HGC-27 cell growth and migration via the miR-140/ADAM10 axis. Int J Biol Macromol. 2019 Feb 1;122:817-823. doi: 10.1016/j.ijbiomac.2018.10.214.
284. Li H, Xue Y, Ma J, Shao L, Wang D, Zheng J, Liu X, Yang C, He Q, Ruan X, Li Z, Liu Y. SNHG1 promotes malignant biological behaviors of glioma cells via microRNA-154-5p/miR-376b-3p- FOXP2- KDM5B participating positive feedback loop. J Exp Clin Cancer Res. 2019 Feb 6;38(1):59. doi: 10.1186/s13046-019-1063-9.
285. Deng R, Zhang J, Chen J. lncRNA SNHG1 negatively regulates miRNA-101-3p to enhance the expression of ROCK1 and promote cell proliferation, migration and invasion in osteosarcoma. Int J Mol Med. 2019 Mar;43(3):1157-1166. doi: 10.3892/ijmm.2018.4039.
286. Li W, Dong X, He C, Tan G, Li Z, Zhai B, Feng J, Jiang X, Liu C, Jiang H, Sun X. LncRNA SNHG1 contributes to sorafenib resistance by activating the Akt pathway and is positively regulated by miR-21 in hepatocellular carcinoma cells. J Exp Clin Cancer Res. 2019 May 3;38(1):183. doi: 10.1186/s13046-019-1177-0.

287. Thin KZ, Tu JC, Raveendran S. Long non-coding SNHG1 in cancer. Clin Chim Acta. 2019 Jul;494:38-47. doi: 10.1016/j.cca.2019.03.002.
288. Huang D, Wei Y, Zhu J, Wang F. Long non-coding RNA SNHG1 functions as a competitive endogenous RNA to regulate PDCD4 expression by sponging miR-195-5p in hepatocellular carcinoma. Gene. 2019 Sep 25;714:143994. doi: 10.1016/j.gene.2019.143994.
289. Li Z, Lu Q, Zhu D, Han Y, Zhou X, Ren T. Lnc-SNHG1 may promote the progression of non-small cell lung cancer by acting as a sponge of miR-497. Biochem Biophys Res Commun. 2018 Nov 30;506(3):632-640. doi: 10.1016/j.bbrc.2018.10.086.
290. Xu M, Chen X, Lin K, Zeng K, Liu X, Pan B, Xu X, Xu T, Hu X, Sun L, He B, Pan Y, Sun H, Wang S. The long noncoding RNA SNHG1 regulates colorectal cancer cell growth through interactions with EZH2 and miR-154-5p. Mol Cancer. 2018 Sep 28;17(1):141. doi: 10.1186/s12943-018-0894-x.
291. Wang H, Wang G, Gao Y, Zhao C, Li X, Zhang F, Jiang C, Wu B. Lnc-SNHG1 Activates the TGFBR2/SMAD3 and RAB11A/Wnt/β-Catenin Pathway by Sponging MiR-302/372/373/520 in Invasive Pituitary Tumors. Cell Physiol Biochem. 2018;48(3):1291-1303. doi: 10.1159/000492089.
292. Wang Z, Wang R, Wang K, Liu X. Upregulated long noncoding RNA Snhg1 promotes the angiogenesis of brain microvascular endothelial cells after oxygen-glucose deprivation treatment by targeting miR-199a. Can J Physiol Pharmacol. 2018 Sep;96(9):909-915. doi: 10.1139/cjpp-2018-0107.
293. Zhang L, Luo X, Chen F, Yuan W, Xiao X, Zhang X, Dong Y, Zhang Y, Liu Y. LncRNA SNHG1 regulates cerebrovascular pathologies as a competing endogenous RNA through HIF-1α/VEGF signaling in ischemic stroke. J Cell Biochem. 2018 Jul;119(7):5460-5472. doi: 10.1002/jcb.26705.
294. Kaur S, Elkahloun AG, Arakelyan A, Young L, Myers TG, Otaizo-Carrasquero F, Wu W, Margolis L, Roberts DD. CD63, MHC class 1, and CD47 identify subsets of extracellular vesicles containing distinct populations of noncoding RNAs. Sci Rep. 2018 Feb 7;8(1):2577. doi: 10.1038/s41598-018-20936-7.
295. Lan T, Yuan K, Yan X, Xu L, Liao H, Hao X, Wang J, Liu H, Chen X, Xie K, Li J, Liao M, Huang J, Zeng Y, Wu H. LncRNA SNHG10 Facilitates Hepatocarcinogenesis and Metastasis by Modulating Its Homolog SCARNA13 via a Positive Feedback Loop. Cancer Res. 2019 Jul 1;79(13):3220-3234. doi: 10.1158/0008-5472.CAN-18-4044.
296. Sun Y, Liu J, Chu L, Yang W, Liu H, Li C, Yang J. Long noncoding RNA SNHG12 facilitates the tumorigenesis of glioma through miR-101-3p/FOXP1 axis. Gene. 2018 Nov 15;676:315-321. doi: 10.1016/j.gene.2018.08.034.
297. Lei W, Wang ZL, Feng HJ, Lin XD, Li CZ, Fan D. Long non-coding RNA SNHG12promotes the proliferation and migration of glioma cells by binding to HuR. Int J Oncol. 2018 Sep;53(3):1374-1384. doi: 10.3892/ijo.2018.4478.
298. Zhou S, Yu L, Xiong M, Dai G. LncRNA SNHG12 promotes tumorigenesis and metastasis in osteosarcoma by upregulating Notch2 by sponging miR-195-5p. Biochem Biophys Res Commun. 2018 Jan 8;495(2):1822-1832. doi: 10.1016/j.bbrc.2017.12.047.
299. Song J, Wu X, Ma R, Miao L, Xiong L, Zhao W. Long noncoding RNA SNHG12 promotes cell proliferation and activates Wnt/β-catenin signaling in prostate cancer through sponging microRNA-195. J Cell Biochem. 2019 Aug;120(8):13066-13075. doi: 10.1002/jcb.28578.
300. Cheng G, Song Z, Liu Y, Xiao H, Ruan H, Cao Q, Wang K, Xiao W, Xiong Z, Liu D, Chen K, Zhang X. Long noncoding RNA SNHG12 indicates the prognosis of prostate cancer and accelerates tumorigenesis via sponging miR-133b. J Cell Physiol. 2019 Jul 2. doi: 10.1002/jcp.29039. [Epub ahead of print]
301. Yin WL, Yin WG, Huang BS, Wu LX. LncRNA SNHG12 inhibits miR-199a to upregulate SIRT1 to attenuate cerebral ischemia/reperfusion injury through activating AMPK signaling pathway. Neurosci Lett. 2019 Jan 18;690:188-195. doi: 10.1016/j.neulet.2018.08.026.
302. Zhou B, Li L, Li Y, Sun H, Zeng C. Long noncoding RNA SNHG12 mediates doxorubicin resistance of osteosarcoma via miR-320a/MCL1 axis. Biomed Pharmacother. 2018 Oct;106:850-857. doi: 10.1016/j.biopha.2018.07.003.
303. Liu X, Zheng J, Xue Y, Qu C, Chen J, Wang Z, Li Z, Zhang L, Liu Y. Inhibition of TDP43-Mediated SNHG12-miR-195-SOX5 Feedback Loop Impeded Malignant Biological Behaviors of Glioma Cells. Mol Ther Nucleic Acids. 2018 Mar 2;10:142-158. doi: 10.1016/j.omtn.2017.12.001.
304. Ma Z, Huang H, Wang J, Zhou Y, Pu F, Zhao Q, Peng P, Hui B, Ji H, Wang K. Long non-coding RNA SNHG15 inhibits P15 and KLF2 expression to promote pancreatic cancer proliferation through EZH2-mediated H3K27me3. Oncotarget. 2017 Aug 18;8(48):84153-84167. doi: 10.18632/oncotarget.20359.
305. Du Y, Kong C, Zhu Y, Yu M, Li Z, Bi J, Li Z, Liu X, Zhang Z, Yu X. Knockdown of SNHG15 suppresses renal cell carcinoma proliferation and EMT by regulating the NF-κB signaling pathway. Int J Oncol. 2018 Jul;53(1):384-394. doi: 10.3892/ijo.2018.4395.
306. Jiang H, Li T, Qu Y, Wang X, Li B, Song J, Sun X, Tang Y, Wan J, Yu Y, Zhan J, Zhang H. Long non-coding RNA SNHG15 interacts with and stabilizes transcription factor Slug and promotes colon cancer progression. Cancer Lett. 2018 Jul 1;425:78-87. doi: 10.1016/j.canlet.2018.03.038.
307. Saeinasab M, Bahrami AR, González J, Marchese FP, Martinez D, Mowla SJ, Matin MM, Huarte M. SNHG15 is a bifunctional MYC-regulated noncoding locus encoding a lncRNA that promotes cell proliferation, invasion and drug resistance in colorectal cancer by interacting with AIF. J Exp Clin Cancer Res. 2019 Apr 24;38(1):172. doi: 10.1186/s13046-019-1169-0.
308. Zhang Y, Zhang D, Lv J, Wang S, Zhang Q. LncRNA SNHG15 acts as an oncogene in prostate cancer by regulating miR-338-3p/FKBP1A axis. Gene. 2019 Jul 15;705:44-50. doi: 10.1016/j.gene.2019.04.033.
309. Li M, Bian Z, Jin G, Zhang J, Yao S, Feng Y, Wang X, Yin Y, Fei B, You Q, Huang Z. LncRNA-SNHG15 enhances cell proliferation in colorectal cancer by inhibiting miR-338-3p. Cancer Med. 2019 May;8(5):2404-2413. doi: 10.1002/cam4.2105.
310. Wu DM, Wang S, Wen X, Han XR, Wang YJ, Shen M, Fan SH, Zhang ZF, Shan Q, Li MQ, Hu B, Lu J, Chen GQ, Zheng YL. LncRNA SNHG15 acts as a ceRNA to regulate YAP1-Hippo signaling pathway by sponging miR-200a-3p in papillary thyroid carcinoma. Cell Death Dis. 2018 Sep 20;9(10):947. doi: 10.1038/s41419-018-0975-1.
311. Jin B, Jin H, Wu HB, Xu JJ, Li B. Long non-coding RNA SNHG15 promotes CDK14 expression via miR-486 to accelerate non-small cell lung cancer cells progression and metastasis. J Cell Physiol. 2018 Sep;233(9):7164-7172. doi: 10.1002/jcp.26543.
312. Kong Q, Qiu M. Long noncoding RNA SNHG15 promotes human breast cancer proliferation, migration and invasion by sponging miR-211-3p. Biochem Biophys Res Commun. 2018 Jan 8;495(2):1594-1600. doi: 10.1016/j.bbrc.2017.12.013.
313. Ma Y, Xue Y, Liu X, Qu C, Cai H, Wang P, Li Z, Li Z, Liu Y. SNHG15 affects the growth of glioma microvascular endothelial cells by negatively regulating miR-153. Oncol Rep. 2017 Nov;38(5):3265-3277. doi: 10.3892/or.2017.5985.
314. Zhu H, Zeng Y, Zhou CC, Ye W. SNHG16/miR-216-5p/ZEB1 signal pathway contributes to the tumorigenesis of cervical cancer cells. Arch Biochem Biophys. 2018 Jan 1;637:1-8. doi: 10.1016/j.abb.2017.11.003.
315. Li W, Xu W, Song JS, Wu T, Wang WX. LncRNA SNHG16 promotes cell proliferation through miR-302a-3p/FGF19 axis in hepatocellular carcinoma. Neoplasma. 2019 May 23;66(3):397-404. doi: 10.4149/neo_2018_180720N504.
316. Wang X, Hu K, Chao Y, Wang L. LncRNA SNHG16 promotes proliferation, migration and invasion of osteosarcoma cells by targeting miR-1301/BCL9 axis. Biomed Pharmacother. 2019 Jun;114:108798. doi: 10.1016/j.biopha.2019.108798.

317. Liu S, Zhang W, Liu K, Liu Y. LncRNA SNHG16 promotes tumor growth of pancreatic cancer by targeting miR-218-5p. Biomed Pharmacother. 2019 Jun;114:108862. doi: 10.1016/j.biopha.2019.108862.
318. Liao S, Xing S, Ma Y. LncRNA SNHG16 sponges miR-98-5p to regulate cellular processes in osteosarcoma. Cancer Chemother Pharmacol. 2019 Jun;83(6):1065-1074. doi: 10.1007/s00280-019-03822-5.
319. Wang W, Lou C, Gao J, Zhang X, Du Y. LncRNA SNHG16 reverses the effects of miR-15a/16 on LPS-induced inflammatory pathway. Biomed Pharmacother. 2018 Oct;106:1661-1667. doi: 10.1016/j.biopha.2018.07.105.
320. Zhu C, Cheng D, Qiu X, Zhuang M, Liu Z. Long Noncoding RNA SNHG16 Promotes Cell Proliferation by Sponging MicroRNA-205 and Upregulating ZEB1 Expression in Osteosarcoma. Cell Physiol Biochem. 2018;51(1):429-440. doi: 10.1159/000495239.
321. Christensen LL, True K, Hamilton MP, Nielsen MM, Damas ND, Damgaard CK, Ongen H, Dermitzakis E, Bramsen JB, Pedersen JS, Lund AH, Vang S, Stribolt K, Madsen MR, Laurberg S, McGuire SE, Ørntoft TF, Andersen CL. SNHG16 is regulated by the Wnt pathway in colorectal cancer and affects genes involved in lipid metabolism. Mol Oncol. 2016 Oct;10(8):1266-82. doi: 10.1016/j.molonc.2016.06.003.
322. Ma Z, Gu S, Song M, Yan C, Hui B, Ji H, Wang J, Zhang J, Wang K, Zhao Q. Long non-coding RNA SNHG17 is an unfavourable prognostic factor and promotes cell proliferation by epigenetically silencing P57 in colorectal cancer. Mol Biosyst. 2017 Oct 24;13(11):2350-2361. doi: 10.1039/c7mb00280g.
323. Zhang G, Xu Y, Wang S, Gong Z, Zou C, Zhang H, Ma G, Zhang W, Jiang P. LncRNA SNHG17 promotes gastric cancer progression by epigenetically silencing of p15 and p57. J Cell Physiol. 2019 Apr;234(4):5163-5174. doi: 10.1002/jcp.27320.
324. Liu JX, Li W, Li JT, Liu F, Zhou L. Screening key long non-coding RNAs in early-stage colon adenocarcinoma by RNA-sequencing. Epigenomics. 2018 Sep;10(9):1215-1228. doi: 10.2217/epi-2017-0155.
325. Cao M, Li H, Zhao J, Cui J, Hu G. Identification of age- and gender-associated long noncoding RNAs in the human brain with Alzheimer's disease. Neurobiol Aging. 2019 Sep;81:116-126. doi: 10.1016/j.neurobiolaging.2019.05.023.
326. Zheng S, Jiang F, Ge D, Tang J, Chen H, Yang J, Yao Y, Yan J, Qiu J, Yin Z, Ni Y, Zhao L, Chen X, Li H, Yang L. LncRNA SNHG3/miRNA-151a-3p/RAB22A axis regulates invasion and migration of osteosarcoma. Biomed Pharmacother. 2019 Apr;112:108695. doi: 10.1016/j.biopha.2019.108695.
327. Fei F, He Y, He S, He Z, Wang Y, Wu G, Li M. LncRNA SNHG3 enhances the malignant progress of glioma through silencing KLF2 and p21. Biosci Rep. 2018 Sep 7;38(5). pii: BSR20180420. doi: 10.1042/BSR20180420.
328. Wang L, Su K, Wu H, Li J, Song D. LncRNA SNHG3 regulates laryngeal carcinoma proliferation and migration by modulating the miR-384/WEE1 axis. Life Sci. 2019 Sep 1;232:116597. doi: 10.1016/j.lfs.2019.116597.
329. Zhao Q, Wu C, Wang J, Li X, Fan Y, Gao S, Wang K. LncRNA SNHG3 Promotes Hepatocellular Tumorigenesis by Targeting miR-326. Tohoku J Exp Med. 2019 Sep;249(1):43-56. doi: 10.1620/tjem.249.43.
330. Li N, Zhan X, Zhan X. The lncRNA SNHG3 regulates energy metabolism of ovarian cancer by an analysis of mitochondrial proteomes. Gynecol Oncol. 2018 Aug;150(2):343-354. doi: 10.1016/j.ygyno.2018.06.013.
331. Huang W, Tian Y, Dong S, Cha Y, Li J, Guo X, Yuan X. The long non-coding RNA SNHG3 functions as a competing endogenous RNA to promote malignant development of colorectal cancer. Oncol Rep. 2017 Sep;38(3):1402-1410. doi: 10.3892/or.2017.5837.
332. Zhao L, Guo H, Zhou B, Feng J, Li Y, Han T, Liu L, Li L, Zhang S, Liu Y, Shi J, Zheng D. Long non-coding RNA SNHG5 suppresses gastric cancer progression by trapping MTA2 in the cytosol. Oncogene. 2016 Nov 3;35(44):5770-5780. doi: 10.1038/onc.2016.110.
333. Damas ND, Marcatti M, Côme C, Christensen LL, Nielsen MM, Baumgartner R, Gylling HM, Maglieri G, Rundsten CF, Seemann SE, Rapin N, Thézenas S, Vang S, Ørntoft T, Andersen CL, Pedersen JS, Lund AH. SNHG5 promotes colorectal cancer cell survival by counteracting STAU1-mediated mRNA destabilization. Nat Commun. 2016 Dec 22;7:13875. doi: 10.1038/ncomms13875.
334. Zhao L, Han T, Li Y, Sun J, Zhang S, Liu Y, Shan B, Zheng D, Shi J. The lncRNA SNHG5/miR-32 axis regulates gastric cancer cell proliferation and migration by targeting KLF4. FASEB J. 2017 Mar;31(3):893-903. doi: 10.1096/fj.201600994R.
335. He B, Bai Y, Kang W, Zhang X, Jiang X. LncRNA SNHG5 regulates imatinib resistance in chronic myeloid leukemia via acting as a CeRNA against MiR-205-5p. Am J Cancer Res. 2017 Aug 1;7(8):1704-1713.
336. Shen H, Wang Y, Shi W, Sun G, Hong L, Zhang Y. LncRNA SNHG5/miR-26a/SOX2 signal axis enhances proliferation of chondrocyte in osteoarthritis. Acta Biochim Biophys Sin (Shanghai). 2018 Feb 1;50(2):191-198. doi: 10.1093/abbs/gmx141.
337. Wang Z, Pan L, Yu H, Wang Y. The long non-coding RNA SNHG5 regulates gefitinib resistance in lung adenocarcinoma cells by targetting *miR-377*/CASP1 axis. Biosci Rep. 2018 Aug 29;38(4). pii: BSR20180400. doi: 10.1042/BSR20180400.
338. Wang Z, Wang Z, Liu J, Yang H. Long non-coding RNA SNHG5 sponges miR-26a to promote the tumorigenesis of osteosarcoma by targeting ROCK1. Biomed Pharmacother. 2018 Nov;107:598-605. doi: 10.1016/j.biopha.2018.08.025.
339. Xin L, Zhou LQ, Liu L, Yuan YW, Zhang HT, Zeng F. METase promotes cell autophagy via promoting SNHG5 and suppressing miR-20a in gastric cancer. Int J Biol Macromol. 2019 Feb 1;122:1046-1052. doi: 10.1016/j.ijbiomac.2018.09.051.
340. Guo T, Wang H, Liu P, Xiao Y, Wu P, Wang Y, Chen B, Zhao Q, Liu Z, Liu Q. SNHG6 Acts as a Genome-Wide Hypomethylation Trigger via Coupling of miR-1297-Mediated S-Adenosylmethionine-Dependent Positive Feedback Loops. Cancer Res. 2018 Jul 15;78(14):3849-3864. doi: 10.1158/0008-5472.CAN-17-3833.
341. Li Y, Li D, Zhao M, Huang S, Zhang Q, Lin H, Wang W, Li K, Li Z, Huang W, Che Y, Huang C. Long noncoding RNA SNHG6 regulates p21 expression via activation of the JNK pathway and regulation of EZH2 in gastric cancer cells. Life Sci. 2018 Sep 1;208:295-304. doi: 10.1016/j.lfs.2018.07.032.
342. Liang R, Xiao G, Wang M, Li X, Li Y, Hui Z, Sun X, Qin S, Zhang B, Du N, Liu D, Ren H. SNHG6 functions as a competing endogenous RNA to regulate E2F7 expression by sponging miR-26a-5p in lung adenocarcinoma. Biomed Pharmacother. 2018 Nov;107:1434-1446. doi: 10.1016/j.biopha.2018.08.099.
343. Wang X, Lai Q, He J, Li Q, Ding J, Lan Z, Gu C, Yan Q, Fang Y, Zhao X, Liu S. LncRNA SNHG6 promotes proliferation, invasion and migration in colorectal cancer cells by activating TGF-β/Smad signaling pathway via targeting UPF1 and inducing EMT via regulation of ZEB1. Int J Med Sci. 2019 Jan 1;16(1):51-59. doi: 10.7150/ijms.27359.
344. Lv P, Qiu X, Gu Y, Yang X, Xu X, Yang Y. Long non-coding RNA SNHG6 enhances cell proliferation, migration and invasion by regulating miR-26a-5p/MAPK6 in breast cancer. Biomed Pharmacother. 2019 Feb;110:294-301. doi: 10.1016/j.biopha.2018.11.016.
345. Jiang Y, Zhuang J, Lin Y, Wang X, Chen J, Han F. Long noncoding RNA SNHG6 contributes to ventricular septal defect formation via negative regulation of miR-101 and activation of Wnt/β-catenin pathway. Pharmazie. 2019 Jan 1;74(1):23-28. doi: 10.1691/ph.2019.8736.
346. Li Z, Qiu R, Qiu X, Tian T. SNHG6 Promotes Tumor Growth via Repression of P21 in Colorectal Cancer. Cell Physiol Biochem. 2018;49(2):463-478. doi: 10.1159/000492986.
347. Meng Q, Yang BY, Liu B, Yang JX, Sun Y. Long non-coding RNA SNHG6 promotes glioma tumorigenesis by sponging miR-101-3p. Int J Biol Markers. 2018 May;33(2):148-155. doi: 10.1177/1724600817747524.

348. Yan K, Tian J, Shi W, Xia H, Zhu Y. LncRNA SNHG6 is Associated with Poor Prognosis of Gastric Cancer and Promotes Cell Proliferation and EMT through Epigenetically Silencing p27 and Sponging miR-101-3p. Cell Physiol Biochem. 2017;42(3):999-1012. doi: 10.1159/000478682.
349. Ren J, Yang Y, Xue J, Xi Z, Hu L, Pan SJ, Sun Q. Long noncoding RNA SNHG7 promotes the progression and growth of glioblastoma via inhibition of miR-5095. Biochem Biophys Res Commun. 2018 Feb 5;496(2):712-718. doi: 10.1016/j.bbrc.2018.01.109.
350. Qi H, Wen B, Wu Q, Cheng W, Lou J, Wei J, Huang J, Yao X, Weng G. Long noncoding RNA SNHG7 accelerates prostate cancer proliferation and cycle progression through cyclin D1 by sponging miR-503. Biomed Pharmacother. 2018 Jun;102:326-332. doi: 10.1016/j.biopha.2018.03.011.
351. Chen Y, Peng Y, Xu Z, Ge B, Xiang X, Zhang T, Gao L, Shi H, Wang C, Huang J. Knockdown of lncRNA SNHG7 inhibited cell proliferation and migration in bladder cancer through activating Wnt/β-catenin pathway. Pathol Res Pract. 2019 Feb;215(2):302-307. doi: 10.1016/j.prp.2018.11.015.
352. Deng Y, Zhao F, Zhang Z, Sun F, Wang M. Long Noncoding RNA SNHG7 Promotes the Tumor Growth and Epithelial-to-Mesenchymal Transition via Regulation of miR-34a Signals in Osteosarcoma. Cancer Biother Radiopharm. 2018 Nov;33(9):365-372. doi: 10.1089/cbr.2018.2503.
353. She K, Huang J, Zhou H, Huang T, Chen G, He J. lncRNA-SNHG7 promotes the proliferation, migration and invasion and inhibits apoptosis of lung cancer cells by enhancing the FAIM2 expression. Oncol Rep. 2016 Nov;36(5):2673-2680. doi: 10.3892/or.2016.5105.
354. Dong J, Teng F, Guo W, Yang J, Ding G, Fu Z. lncRNA SNHG8 Promotes the Tumorigenesis and Metastasis by Sponging miR-149-5p and Predicts Tumor Recurrence in Hepatocellular Carcinoma. Cell Physiol Biochem. 2018;51(5):2262-2274. doi: 10.1159/000495871.
355. Chen C, Zhang Z, Li J, Sun Y. SNHG8 is identified as a key regulator in non-small-cell lung cancer progression sponging to miR-542-3p by targeting CCND1/CDK6. Onco Targets Ther. 2018 Sep 20;11:6081-6090. doi: 10.2147/OTT.S170482.
356. Chen Z, Bu N, Qiao X, Zuo Z, Shu Y, Liu Z, Qian Z, Chen J, Hou Y. Forkhead Box M1 Transcriptionally Regulates the Expression of Long Noncoding RNAs Snhg8 and Gm26917 to Promote Proliferation and Survival of Muscle Satellite Cells. Stem Cells. 2018 Jul;36(7):1097-1108. doi: 10.1002/stem.2824.
357. Yang CH, Zhang XY, Zhou LN, Wan Y, Song LL, Gu WL, Liu R, Ma YN, Meng HR, Tian YL, Zhang Y. LncRNA SNHG8 participates in the development of endometrial carcinoma through regulating c-MET expression by miR-152. Eur Rev Med Pharmacol Sci. 2018 Mar;22(6):1629-1637. doi: 10.26355/eurrev_201803_14698.
358. Zhang B, Li C, Sun Z. Long non-coding RNA LINC00346, LINC00578, LINC00673, LINC00671, LINC00261, and SNHG9 are novel prognostic markers for pancreatic cancer. Am J Transl Res. 2018 Aug 15;10(8):2648-2658.
359. Zhang H, Qin D, Jiang Z, Zhang J. SNHG9/miR-199a-5p/Wnt2 Axis Regulates Cell Growth and Aerobic Glycolysis in Glioblastoma. J Neuropathol Exp Neurol. 2019 Oct 1;78(10):939-948. doi: 10.1093/jnen/nlz078.
360. Zhang Y, Xu YZ, Sun N, Liu JH, Chen FF, Guan XL, Li A, Wang F, Zhao QF, Wang HY, Song SS, Yu W, Zhao JN, Li XJ. Long noncoding RNA expression profile in fibroblast-like synoviocytes from patients with rheumatoid arthritis. Arthritis Res Ther. 2016 Oct 6;18(1):227.
361. Li DS, Ainiwaer JL, Sheyhiding I, Zhang Z, Zhang LW. Identification of key long non-coding RNAs as competing endogenous RNAs for miRNA-mRNA in lung adenocarcinoma. Eur Rev Med Pharmacol Sci. 2016 Jun;20(11):2285-95.
362. Hou S, Lin Q, Guan F, Lin C. LncRNA TNRC6C-AS1 regulates UNC5B in thyroid cancer to influence cell proliferation, migration, and invasion as a competing endogenous RNA of miR-129-5p. J Cell Biochem. 2018 Nov;119(10):8304-8316. doi: 10.1002/jcb.26868.
363. Yang LX, Wu J, Guo ML, Zhang Y, Ma SG. Suppression of long non-coding RNA TNRC6C-AS1 protects against thyroid carcinoma through DNA demethylation of STK4 via the Hippo signalling pathway. Cell Prolif. 2019 May;52(3):e12564. doi: 10.1111/cpr.12564.
364. Liu Z, Yan HY, Xia SY, Zhang C, Xiu YC. Downregulation of long non-coding RNA TRIM52-AS1 functions as a tumor suppressor in renal cell carcinoma. Mol Med Rep. 2016 Apr;13(4):3206-12. doi: 10.3892/mmr.2016.4908.
365. Ma LY, Xie XW, Ma L, Pang JL, Xiong XM, Zheng HD, Shen XL, Wen ZG, Wang HY. Downregulated long non-coding RNA TRPM2-AS inhibits cisplatin resistance of non-small cell lung cancer cells via activation of p53- p66shc pathway. Eur Rev Med Pharmacol Sci. 2017 Jun;21(11):2626-2634.
366. Huang B, Chang C, Wang BL, Li H. ELK1-induced upregulation of lncRNA TRPM2-AS promotes tumor progression in gastric cancer by regulating miR-195/ HMGA1 axis. J Cell Biochem. 2019 Oct;120(10):16921-16933. doi: 10.1002/jcb.28951.
367. Sun T, Song Y, Yu H, Luo X. Identification of lncRNA TRPM2-AS/miR-140-3p/PYCR1 axis's proliferates and anti-apoptotic effect on breast cancer using co-expression network analysis. Cancer Biol Ther. 2019;20(6):760-773. doi: 10.1080/15384047.2018.1564563.
368. Di A, Kiya T, Gong H, Gao X, Malik AB. Role of the phagosomal redox-sensitive TRP channel TRPM2 in regulating bactericidal activity of macrophages. J Cell Sci. 2017 Feb 15;130(4):735-744. doi: 10.1242/jcs.196014.
369. Bao L, Chen SJ, Conrad K, Keefer K, Abraham T, Lee JP, Wang J, Zhang XQ, Hirschler-Laszkiewicz I, Wang HG, Dovat S, Gans B, Madesh M, Cheung JY, Miller BA. Depletion of the Human Ion Channel TRPM2 in Neuroblastoma Demonstrates Its Key Role in Cell Survival through Modulation of Mitochondrial Reactive Oxygen Species and Bioenergetics. J Biol Chem. 2016 Nov 18;291(47):24449-24464.
370. Rah SY, Kwak JY, Chung YJ, Kim UH. ADP-ribose/TRPM2-mediated Ca2+ signaling is essential for cytolytic degranulation and antitumor activity of natural killer cells. Sci Rep. 2015 Mar 25;5:9482. doi: 10.1038/srep09482.
371. Wang J, Yang X, Li R, Wang L, Gu Y, Zhao Y, Huang KH, Cheng T, Yuan Y, Gao S. Long non-coding RNA MYU promotes prostate cancer proliferation by mediating the miR-184/c-Myc axis. Oncol Rep. 2018 Nov;40(5):2814-2825. doi: 10.3892/or.2018.6661.
372. Girard A, Sachidanandam R, Hannon GJ, Carmell MA. A germline-specific class of small RNAs binds mammalian Piwi proteins. Nature. 2006 Jul 13;442(7099):199-202.
373. Han YN, Li Y, Xia SQ, Zhang YY, Zheng JH, Li W. PIWI Proteins and PIWI-Interacting RNA: Emerging Roles in Cancer. Cell Physiol Biochem. 2017;44(1):1-20. doi: 10.1159/000484541.
374. Duleh SN, Welch MD. WASH and the Arp2/3 complex regulate endosome shape and trafficking. Cytoskeleton (Hoboken). 2010 Mar;67(3):193-206. doi: 10.1002/cm.20437.
375. Alekhina O, Burstein E, Billadeau DD. Cellular functions of WASP family proteins at a glance. J Cell Sci. 2017 Jul 15;130(14):2235-2241. doi: 10.1242/jcs.199570.
376. Simonetti B, Cullen PJ. Actin-dependent endosomal receptor recycling. Curr Opin Cell Biol. 2019 Feb;56:22-33. doi: 10.1016/j.ceb.2018.08.006.

377. Farina F, Ramkumar N, Brown L, Samandar Eweis D, Anstatt J, Waring T, Bithell J, Scita G, Thery M, Blanchoin L, Zech T, Baum B. Local actin nucleation tunes centrosomal microtubule nucleation during passage through mitosis. EMBO J. 2019 Jun 3;38(11). pii: e99843. doi: 10.15252/embj.201899843.
378. Liu C, Lin J. Long noncoding RNA ZEB1-AS1 acts as an oncogene in osteosarcoma by epigenetically activating ZEB1. Am J Transl Res. 2016 Oct 15;8(10):4095-4105.
379. Gong H, Wen H, Zhu X, Lian Y, Yang X, Qian Z, Zhu J. High expression of long non-coding RNA ZEB1-AS1 promotes colorectal cancer cell proliferation partially by suppressing p15 expression. Tumour Biol. 2017 Jun;39(6):1010428317705336. doi: 10.1177/1010428317705336.
380. Su W, Xu M, Chen X, Chen N, Gong J, Nie L, Li L, Li X, Zhang M, Zhou Q. Long noncoding RNA ZEB1-AS1 epigenetically regulates the expressions of ZEB1 and downstream molecules in prostate cancer. Mol Cancer. 2017 Aug 23;16(1):142. doi: 10.1186/s12943-017-0711-y.
381. Chen C, Feng Y, Wang X. LncRNA ZEB1-AS1 expression in cancer prognosis: Review and meta-analysis. Clin Chim Acta. 2018 Sep;484:265-271. doi: 10.1016/j.cca.2018.06.007.
382. Gao R, Zhang N, Yang J, Zhu Y, Zhang Z, Wang J, Xu X, Li Z, Liu X, Li Z, Li J, Kong C, Bi J.Long non-coding RNA ZEB1-AS1 regulates miR-200b/FSCN1 signaling and enhances migration and invasion induced by TGF-β1 in bladder cancer cells. J Exp Clin Cancer Res. 2019 Mar 1;38(1):111. doi: 10.1186/s13046-019-1102-6.
383. Wang Q, Du X, Yang M, Xiao S, Cao J, Song J, Wang L. LncRNA ZEB1-AS1 contributes to STAT3 activation by associating with IL-11 in B-lymphoblastic leukemia. Biotechnol Lett. 2017 Dec;39(12):1801-1810. doi: 10.1007/s10529-017-2421-3
384. Liu C, Pan C, Cai Y, Wang H. Interplay Between Long Noncoding RNA ZEB1-AS1 and miR-200s Regulates Osteosarcoma Cell Proliferation and Migration. J Cell Biochem. 2017 Aug;118(8):2250-2260. doi: 10.1002/jcb.25879.
385. Xia B, Hou Y, Chen H, Yang S, Liu T, Lin M, Lou G. Long non-coding RNA ZFAS1 interacts with miR-150-5p to regulate Sp1 expression and ovarian cancer cell malignancy. Oncotarget. 2017 Mar 21;8(12):19534-19546. doi: 10.18632/oncotarget.14663.
386. Fang C, Zan J, Yue B, Liu C, He C, Yan D. Long non-coding ribonucleic acid zinc finger antisense 1 promotes the progression of colonic cancer by modulating ZEB1 expression. J Gastroenterol Hepatol. 2017 Jun;32(6):1204-1211. doi: 10.1111/jgh.13646.
387. Nie F, Yu X, Huang M, Wang Y, Xie M, Ma H, Wang Z, De W, Sun M. Long noncoding RNA ZFAS1 promotes gastric cancer cells proliferation by epigenetically repressing KLF2 and NKD2 expression. Oncotarget. 2017 Jun 13;8(24):38227-38238. doi: 10.18632/oncotarget.9611.
388. Liu G, Wang L, Han H, Li Y, Lu S, Li T, Cheng C. LncRNA ZFAS1 promotes growth and metastasis by regulating BMI1 and ZEB2 in osteosarcoma. Am J Cancer Res. 2017 Jul 1;7(7):1450-1462.
389. Wu T, Wu D, Wu Q, Zou B, Huang X, Cheng X, Wu Y, Hong K, Li P, Yang R, Li Y, Cheng Y. Knockdown of Long Non-Coding RNA-ZFAS1 Protects Cardiomyocytes Against Acute Myocardial Infarction Via Anti-Apoptosis by Regulating miR-150/CRP. J Cell Biochem. 2017 Oct;118(10):3281-3289. doi: 10.1002/jcb.25979.
390. Xie S, Ge Q, Wang X, Sun X, Kang Y. Long non-coding RNA ZFAS1 sponges miR-484 to promote cell proliferation and invasion in colorectal cancer. Cell Cycle. 2018;17(2):154-161. doi: 10.1080/15384101.2017.1407895.
391. Xu W, He L, Li Y, Tan Y, Zhang F, Xu H. Silencing of lncRNA ZFAS1 inhibits malignancies by blocking Wnt/β-catenin signaling in gastric cancer cells. Biosci Biotechnol Biochem. 2018 Mar;82(3):456-465. doi: 10.1080/09168451.2018.1431518.
392. Ye D, Jian W, Feng J, Liao X. Role of long noncoding RNA ZFAS1 in proliferation, apoptosis and migration of chondrocytes in osteoarthritis. Biomed Pharmacother. 2018 Aug;104:825-831. doi: 10.1016/j.biopha.2018.04.124.
393. He A, He S, Li X, Zhou L. ZFAS1: A novel vital oncogenic lncRNA in multiple human cancers. Cell Prolif. 2019 Jan;52(1):e12513. doi: 10.1111/cpr.12513.
394. Gan S, Ma P, Ma J, Wang W, Han H, Chen L, Li X, Wu F, Sun H. Knockdown of ZFAS1 suppresses the progression of acute myeloid leukemia by regulating microRNA-150/Sp1 and microRNA-150/Myb pathways. Eur J Pharmacol. 2019 Feb 5;844:38-48. doi: 10.1016/j.ejphar.2018.11.036.
395. Dong D, Mu Z, Wei N, Sun M, Wang W, Xin N, Shao Y, Zhao C. Long non-coding RNA ZFAS1 promotes proliferation and metastasis of clear cell renal cell carcinoma via targeting miR-10a/SKA1 pathway. Biomed Pharmacother. 2019 Mar;111:917-925. doi: 10.1016/j.biopha.2018.12.143.
396. Wang M, Ji YQ, Song ZB, Ma XX, Zou YY, Li XS. Knockdown of lncRNA ZFAS1 inhibits progression of nasopharyngeal carcinoma by sponging miR-135a. Neoplasma. 2019 Jun 29;2019. pii: 181213N963. doi: 10.4149/neo_2018_181213N963.
397. Zhang F, Li Y, Xu W, He L, Tan Y, Xu H. Long non-coding RNA ZFAS1 regulates the malignant progression of gastric cancer via the microRNA-200b-3p/Wnt1 axis. Biosci Biotechnol Biochem. 2019 Jul;83(7):1289-1299. doi: 10.1080/09168451.2019.1606697.
398. Han CG, Huang Y, Qin L. Long Non-Coding RNA ZFAS1 as a Novel Potential Biomarker for Predicting the Prognosis of Thyroid Cancer. Med Sci Monit. 2019 Apr 23;25:2984-2992. doi: 10.12659/MSM.912921.
399. Wang JS, Liu QH, Cheng XH, Zhang WY, Jin YC. The long noncoding RNA ZFAS1 facilitates bladder cancer tumorigenesis by sponging miR-329. Biomed Pharmacother. 2018 Jul;103:174-181. doi: 10.1016/j.biopha.2018.04.031.
400. Zhang Y, Jiao L, Sun L, Li Y, Gao Y, Xu C, Shao Y, Li M, Li C, Lu Y, Pan Z, Xuan L, Zhang Y, Li Q, Yang R, Zhuang Y, Zhang Y, Yang B. LncRNA *ZFAS1* as a SERCA2a Inhibitor to Cause Intracellular Ca_{2+} Overload and Contractile Dysfunction in a Mouse Model of Myocardial Infarction. Circ Res. 2018 May 11;122(10):1354-1368. doi: 10.1161/CIRCRESAHA.117.312117.
401. Li T, Xie J, Shen C, Cheng D, Shi Y, Wu Z, Deng X, Chen H, Shen B, Peng C, Li H, Zhan Q, Zhu Z. Amplification of Long Noncoding RNA ZFAS1 Promotes Metastasis in Hepatocellular Carcinoma. Cancer Res. 2015 Aug 1;75(15):3181-91. doi: 10.1158/0008-5472.CAN-14-3721.
402. Prakash T, Sharma VK, Adati N, Ozawa R, Kumar N, Nishida Y, Fujikake T, Takeda T, Taylor TD. Expression of conjoined genes: another mechanism for gene regulation in eukaryotes. PLoS One. 2010 Oct 12;5(10):e13284. doi: 10.1371/journal.pone.0013284.
403. Funnell T, Tasaki S, Oloumi A, Araki S, Kong E, Yap D, Nakayama Y, Hughes CS, Cheng SG, Tozaki H, Iwatani M, Sasaki S, Ohashi T, Miyazaki T, Morishita N, Morishita D, Ogasawara-Shimizu M, Ohori M, Nakao S, Karashima M, Sano M, Murai A, Nomura T, Uchiyama N, Kawamoto T, Hara R, Nakanishi O, Shumansky K, Rosner J, Wan A, McKinney S, Morin GB, Nakanishi A, Shah S, Toyoshiba H, Aparicio S. CLK-dependent exon recognition and conjoined gene formation revealed with a novel small molecule inhibitor. Nat Commun. 2017 Feb 23;8(1):7. doi: 10.1038/s41467-016-0008-7.

Table S10
Alphabetic list of full names of symbols for protein coding genes

**Symbol**	**Full name**
Aamdc	adipogenesis associated, Mth938 domain containing
Aars	alanyl-tRNA synthetase
Aatf	apoptosis antagonizing transcription factor
Abca16	ATP-binding cassette, subfamily A (ABC1), member 16
Abhd13	abhydrolase domain containing 13
ABL1	ABL proto-oncogene 1, non-receptor tyrosine kinase
Abt1	activator of basal transcription 1
Acaa2	acetyl-CoA acyltransferase 2
Acat2	acetyl-Coenzyme A acetyltransferase 3
Acpl2	acid phosphatase-like 2
Acsl1	acyl-CoA synthetase long-chain family member 1
Acsl4	acyl-CoA synthetase long-chain family member 4
Acss2	acyl-CoA synthetase short-chain family member 2
Acvr1	activin A receptor type 2
Acy3	aspartoacylase (aminocyclase) 3
Adam10, 15, 9	a disintegrin and metallopeptidase domain 10, 15 (metargidin), 9
Adamts1, 15, 2, 7	ADAM metallopeptidase with thrombospondin type 1 motif, 1, 15, 2, 7
Adap1	ArfGAP with dual PH domains 1
Adarb1	adenosine deaminase, RNA-specific, B1
Adcy9	adenylate cyclase 9
Add3	adducin 3 (gamma)
Adgrb1	adhesion GPCR B1
Adhfe1	alcohol dehydrogenase, iron containing, 1
Adm	adrenomedullin
Adora2b	adenosine A2B receptor
Adrbk1	adrenergic, beta, receptor kinase 1
Aen	apoptosis enhancing nuclease
Agfg2	ArfGAP with FG repeats 2
Agps	alkylglycerone phosphate synthase
Agrn	Agrin
Agtr1a	angiotensin II receptor, type 1a
Ahdc1	AT hook, DNA binding motif, containing 1
Ahi1	Abelson helper integration site 1
Aig1	androgen-induced 1
Akap13, 17b, 2	A kinase (PRKA) anchor protein 13, 17B, 2
Akna	AT-hook transcription factor
Akr1c13, c15, c19, e2	aldo-keto reductase family 1, member C13, member C-like 1, member C19, member E2
Akt	Akt serine/treonine kinase 1
Alcam	activated leukocyte cell adhesion molecule
Aldh3a1	aldehyde dehydrogenase 3 family, member A1
Alix/PDCD6IP	programmed cell death 6 interacting protein
Alk	ALK receptor tyrosine kinase
Alkbh1	alkB, alkylation repair homolog, histone H2A dioygenase
Alkbh3	alkB homolog 3 alpha-ketoglutarate-dependent dioxygenaseprovided
Ammecr1l	AMME chromosomal region gene 1-like
Angel1	angel homolog 1
Angptl4	angiopoietin-like 4
Ankrd1, 28, 37, 50	ankyrin repeat domain 1, 28, 37, 50
Anks3, 6	ankyrin repeat and sterile alpha motif domain containing 3, 6
Anpep	alanyl (membrane) aminopeptidase
Antxr2	Antxr cell adhesion molecule 2
Anxa1, 3, 6	annexin A1, A3, A6
Aoc3	amine oxidase, copper containing 3
Aox1	aldehyde oxidase 1
Ap2a2, s1	adaptor-related protein complex 2, alpha 2 subunit, sigma 1 subunit
Ap3s1	adaptor-related protein complex 3, sigma 1 subunit
Apex1	APEX nuclease (multifunctional DNA repair enzyme) 1
Apol9a	apolipoprotein L 9a
Aqp1	aquaporin 1
Arc	activity-regulated cytoskeleton-associated protein
Areg	amphiregulin
Arf4	ADP-ribosylation factor 4
Arg1	arginase, liver
Arglu1	arginine and glutamate rich 1
Arhgap11a, 18, 22, 28	Rho GTPase activating protein 11A, 18, 22, 28
Arhgef2	rho/rac guanine nucleotide exchange factor (GEF) 2
Arid3a	AT rich interactive domain 3A (Bright like)
Arl15	ADP-ribosylation factor-like 15
Arl2bp	ADP-ribosylation factor-like 2 binding protein
Arl8b	ADP-ribosylation factor-like 8B
Arnt2	aryl hydrocarbon receptor nuclear translocator 2
Arpc1a, 5, 5l	actin related protein 2/3 complex, subunit 1A, subunit 5, subunit 5-like
Arsi	arylsulfatase family, member I

Table S10 continued

**Symbol**	**Full name**
Artn	artemin
Asf1b	ASF1 anti-silencing function 1 homolog B
Asl	argininosuccinate lyase
Asns	asparagine synthetase
Aspm	asp (abnormal spindle) homolog, microcephaly associated
Aspn	asporin
Atf3, 4, 6	activating transcription factor 3, 4 (tax-responsive enhancer element B67), 6
Atg12, 16l2	ATG12 autophagy related 12 homolog, 16-like 2
Atp2c1	ATPase, Ca++ transporting, type 2C, member 1
Atp5j2	ATP synthase, H+ transporting, mitochondrial Fo complex, subunit F2
Atp6v0b, 1a, 1c1, 1d	ATPase, H+ transporting, lysosomal V0 subunit B, V1 subunit A, V1 subunit C1, V1 subunit D
Atp8b2	Atpase, class I, type 8B, member 2
Aurka	aurora kinase A
Azin1	antizyme inhibitor 1
Bard1	BRCA1 associated RING domain 1
Bax	Bcl2 associated X, apoptosis regulator
Bbc3	Bcl-2 binding component 3
Bcap29	B-cell receptor-associated protein 29
Bcar1	breast cancer anti-estrogen resistance 1
Bcat1	branched chain amino acid transaminase 1, cytosolic
Bcl11a	B-cell CLL/lymphoma 11A (zinc finger protein)
Bcl2l1	Bcl2-like 1
Bdh2	3-hydroxybutyrate dehydrogenase, type 2
Bf	complement factor B
Bhlhe40	basic helix-loop-helix family, member e40
Bhmt	betaine-homocysteine S-methyltransferase
Bicc1	bicaudal C homolog 1
Birc5	baculoviral IAP repeat-containing 5
Bloc1s1	biogenesis of lysosomal organelles complex-1, subunit 1
Bmp2, 4	bone morphogenetic protein 2, 4
Bmper	BMP-binding endothelial regulator
Bnip3, 3l	BCL2/adenovirus E1B interacting protein 3, 3-like
Bod1	biorientation of chromosomes in cell division 1
Bola3	bolA homolog 3 (E. coli)
Bop1	block of proliferation 1
Borcs7, 8	BLOC-1 related complex subunit 7, 8
Brip1	BRCA1 interacting protein C-terminal helicase 1
Bst1	bone marrow stromal cell antigen 1
Btd	biotinidase
Btg1, 2, 3	B-cell translocation gene 1, 2, 3
Bub1	budding uninhibited by benzimidazoles 1 homolog, 1 homolog beta
C1orf189 homolog	RGD1564171
C1qtnf6	C1q and tumor necrosis factor related protein 6
C1r, 3, 4b	complement component 1, r subcomponent, 3, 4B
Cables1	Cdk5 and Abl enzyme substrate 1
Cacybp	calcyclin binding protein
Calhm2	calcium homeostasis modulator 2
Calml4	calmodulin-like 4
Car11	carbonic anhydrase 11
Cars	cysteinyl-tRNA synthetase
Casp12, 4, 7	caspase 12, 4, 7
Casq1	calsequestrin 1 (fast-twitch, skeletal muscle)
Catsper2	cation channel, sperm associated 2
Cav1	caveolin 1, caveolae protein
Cby3	chibby homolog 3
Ccar1	cell division cycle and apoptosis regulator 1
Ccdc101, 112, 167	coiled-coil domain containing 101, 112, 167
Ccdc28b, 69, 99	coiled-coil domain containing 28B, 69, 99
Ccl19, 2, 20, 27	chemokine (C-C motif) ligand 19, 2 (Mcp1), 20 (Mip3a), 27
Ccna2, b1, b2, e1, l1	cyclin A2, B1, B2, C, E1, L1
Ccnd1	cyclin D1
Ccnyl1	cyclin Y-like 1
Ccrn4l	CCR4 carbon catabolite repression 4-like
Ccser2	granule cell antiserum positive 14
Cct8	chaperonin containing Tcp1, subunit 8 (theta)
Cd1d1	CD1d1 molecule
Cd24	CD24 molecule
Cd302	CD302 molecule
Cd36	CD36 molecule (thrombospondin receptor)
Cd3eap	CD3e molecule, epsilon associated protein
CD44	CD44 molecule
Cd48	CD48 molecule
Cd54	CD54, ICAM1 (Intercellular adhesion molecule 1)
Cd62L	CD62L, Selectin L
Cd68	CD68 molecule

Table S10 continued

**Symbol**	**Full name**
Cd74	CD74 molecule, major histocompatibility complex, class II invariant chain
Cd81	CD81 molecule
Cd86	CD86 molecule
Cda	cytidine deaminase
Cdc20	cell division cycle 20 homolog
Cdc42bpg	CDC42 binding protein kinase gamma (DMPK-like)
Cdc42ep5	CDC42 effector protein (Rho GTPase binding) 5
Cdc42se2	CDC42 small effector 2
Cdc45	cell division cycle 45 homolog
Cdca3	cell division cycle associated 3
Cdh 5, 16	cadherin 5, 16
Cdk2	cyclin dependent kinase 2
Cdkn1a, 2c, 2d	cyclin-dependent kinase inhibitor 1A, 2C (p18, inhibits CDK4), 2D
Cdkn2aip	CDKN2A interacting protein
Cdpf1	similar to 2210021J22Rik protein
Ceacam10	carcinoembryonic antigen-related cell adhesion molecule 10
Cebpd, g	CCAAT/enhancer binding protein (C/EBP), delta, gamma
Cemip2	cell migration inducing hyaluronidase 2
Cenpf	centromere protein F, J, Q, T, W
Cep250, 55	centrosomal protein 250, 55
Cept1	choline/ethanolamine phosphotransferase 1
Cers1	ceramide synthase 1
Ces1a, 1d, 5a	carboxylesterase 1A, 1D, 5A
Cfap100	cilia and flagella associated protein 100
Cfap100	cilia and flagella associated protein 100
Cfb, h	complement factor B, H
Cfl1	cofilin
Chaf1b	chromatin assembly factor 1, subunit B (p60)
Chchd6	coiled-coil-helix-coiled-coil-helix domain containing 6
Chd2	chromodomain helicase DNA binding protein 2
Chka	choline kinase alpha
Chm	choroideremia (Rab escort protein 1)
Ciart	hypothetical gene supported by NM_017187
Cisd1, 2	CDGSH iron sulfur domain 1, 2
Ciz1	CDKN1A interacting zinc finger protein 1
Ckap4	cytoskeleton-associated protein 4
Cklf	chemokine-like factor
Clec11a	C-type lectin domain family 11, member A
Clic2	chloride intracellular channel 2
Clk1	CDC-like kinase 1
Clmp	CXADR-like membrane protein
Clpp	ClpP caseinolytic peptidase, ATP-dependent, proteolytic subunit homolog (E. coli)
Clrn2	clarin 2
Clu	clusterin
Cnfn	cornifelin
Cnn3	calponin 3, acidic
Cntf	Ciliary neurotrophic factor
Cog8	component of oligomeric golgi complex 8
Col12a1,14a1,1a1,1a2	collagen, type XII, alpha 1, type XIV, alpha 1, type I, alpha 1, 2
Col3a1,4, 5a1,6a1,6a2	collagen, type III, alpha 1, type V, alpha 1, type VI, alpha 1, alpha 2
Commd1	copper metabolism (Murr1) domain containing 1
Comtd1	catechol-O-methyltransferase domain containing 1
Cops7a	COP9 constitutive photomorphogenic homolog subunit 7A
Coq8b	aarF domain containing kinase 4
Cotl1	coactosin-like 1
Cox6a2,b1,b2, 7a2,b	cytochrome c oxidase subunit VIA2, VIB1, VIB2, subunit VIIA2, B
Cp	ceruloplasmin
Cpeb1, 4	cytoplasmic polyadenylation element binding protein 1, 4
Cplx2	complexin 2
Cpne5	copine V
Cr1l	complement component (3b/4b) receptor 1-like
Creb3l1	cAMP responsive element binding protein 3-like 1
Crim1	cysteine rich transmembrane BMP regulator 1 (chordin like)
Crip2	cysteine-rich protein 2
Crtap	cartilage associated protein
Cry1	cryptochrome 1 (photolyase-like)
Cryab, l1	crystallin, alpha B, lambda 1
Csda	cold shock domain protein A
Csf11	colony stimulating factor 1 receptor
Csf2	Granulocyte-macrophage colony stimulating factor (Gmcsf)
Csgalnact1	chondroitin sulfate N-acetylgalactosaminyltransferase 1
Csrnp1	cysteine-serine-rich nuclear protein 1
Csrp2	cysteine and glycine-rich protein 2
Cst6	cystatin E/M
Ctf1	cardiotrophin 1

Table S10 continued

Symbol	Full name
Cth	cystathionase (cystathionine gamma-lyase)
Ctnnal1	catenin (cadherin associated protein), alpha-like 1
Ctps1	CTP synthase
Ctsh, k	cathepsin H, K
Ctu1	cytosolic thiouridylase subunit 1 homolog
Ctxn1, 3	cortexin 1, 3
Cul2	cullin 2
Cuta	cutA divalent cation tolerance homolog
Cx3cl1	chemokine (C-X3-C motif) ligand 1
Cx3cr1	chemokine (C-X3-C motif) receptor 1
Cxcl1, 2, 3, 5, 6	chemokine (C-X-C motif) ligand 1 (CINC-1), 2 (CINC-2a), 3 (CINC-3), 5, 6 (LIX)
Cxcl10, 12, 13	chemokine (C-X-C motif) ligand 10, 12, 13
Cxcr2, 4, 5, 7	chemokine (C-X-C motif) receptor 2, 4, 5, 7
Cyb5r3, basc3	cytochrome b5 reductase 3 b, ascorbate dependent 3
Cyhr1	cysteine and histidine rich 1
Cyp26b1, 3a9	cytochrome P450, family 26, subfamily b, polypeptide 1, family 3, subfamily a, polypeptide 9
Cyp4a8, 51	cytochrome P450, family 4, subfamily a, polypeptide 8, family 51
Cyr61	cysteine-rich, angiogenic inducer, 61
Cyth3	cytohesin 3
Da1-10	Da1-10-like
Daam1	dishevelled associated activator of morphogenesis 1
Dag	diaglycerol
Dars	aspartyl-tRNA synthetase
Dbp	D site of albumin promoter (albumin D-box) binding protein
Dbt	dihydrolipoamide branched chain transacylase E2
Dcaf6	DDB1 and CUL4 associated factor 6
Dclk1	doublecortin-like kinase 1
Dcn	decorin
Dctpp1	dCTP pyrophosphatase 1
Dcun1d1	DCN1, defective in cullin neddylation 1, domain containing 1
Ddah1	dimethylarginine dimethylaminohydrolase 1
Ddit3	DNA-damage inducible transcript 3
Ddx46, 50, 52, 59	DEAD (Asp-Glu-Ala-Asp) box polypeptide 46, 50, 52, 59
Depp1	DEPP1, autophagy regulator
Derl3	Der1-like domain family, member 3
Dexi	dexamethasone-induced transcript
Dgka	diacylglycerol kinase, alpha
Dhodh	dihydroorotate dehydrogenase (quinone)
Dhrs1	dehydrogenase/reductase (SDR family) member 1
Dhx58	DEXH (Asp-Glu-X-His) box polypeptide 58
Dhx9	DEAH (Asp-Glu-Ala-His) box polypeptide 9
Diras1	DIRAS family, GTP-binding RAS-like 1
Dlgap5	discs, large homolog-associated protein 5
Dmtf1	cyclin D binding myb-like transcription factor 1
Dnaja3	DnaJ (Hsp40) homolog, subfamily A, member 3
Dnajc10, 2, 5, 6	DnaJ (Hsp40) homolog, subfamily C, member 10, 2, 5, 6
Dnase1l1	deoxyribonuclease 1-like 1
Dohh	deoxyhypusine hydroxylase/monooxygenase
Dok5	docking protein 5
Dpep1, 2	dipeptidase 1 (renal), 2
Dph5	DPH5 homolog
Dpp8	dipeptidylpeptidase 8
Dqx1	DEAQ box RNA-dependent ATPase 1
Dse	dermatan sulfate epimerase
Dsel	dermatan sulfate epimerase-like
Dstn	destrin
Duox1	dual oxidase 1
Dus2l	dihydrouridine synthase 2-like, SMM1 homolog
Dusp1, 22, 5, 6, 8	dual specificity phosphatase 1, 22, 5, 6, 8
Dut	deoxyuridine triphosphatase
Dync2li1	dynein cytoplasmic 2 light intermediate chain 1
E2f1	E2F transcription factor 1
Ech1	enoyl CoA hydratase 1, peroxisomal
Eci2	enoyl-Coenzyme A delta isomerase 2
Edem2	ER degradation enhancer, mannosidase alpha-like 2
Edn1	endothelin 1
Eef1a2	eukaryotic translation elongation factor 1 alpha 2
Eef2kmt	family with sequence similarity 86, member A
Eefsec	eukaryotic elongation factor, selenocysteine-tRNA-specific
Eepd1	endonuclease/exonuclease/phosphatase family domain containing 1
Efna1, 2	ephrin A1, 2
Egf	epidermal growth factor
Egfr	epidermal growth factor receptor, ERBB1
Egr1, 2	early growth response 1, 2
Ehd2	EH-domain containing 2

Table S10 continued

Symbol	Full name
Eif1, 1ax, 1b	eukaryotic translation initiation factor 1, 1A, X-linked, 1B
Eif2ak2, 2b3, 2s2	eukaryotic translation initiation factor 2-alpha kinase 2, 2B subunit 3, 2 subunit 2 beta
Eif3e, 3j, 4e, 4e3	eukaryotic translation initiation factor 3, subunit E, subunit J, 4E, 4E family member 3
Eif4ebp1	eukaryotic translation initiation factor 4E binding protein 1
Elmsan1	similar to transcriptional regulating protein 132
Elf1	E74 like ETS transcription factor 1
Eln	elastin
Elovl6	ELOVL family member 6, elongation of long chain fatty acids
Emilin1	elastin microfibril interfacer 1
Emp2	epithelial membrane protein 2
Emx1, 2	empty spiracles homeobox 1, 2
Enc1	ectodermal-neural cortex 1
Eno2	enolase 2, gamma, neuronal
Epha1, 2, 3	Eph receptor A1, A2, A3
Ephb1, 3, 4, 6	Eph receptor B1, B3, B4, B6
Epn3	epsin 3
Eprs	glutamyl-prolyl-tRNA synthetase
Eps8	epidermal growth factor receptor pathway substrate 8
Epyc	epiphycan
Eral1	Era (G-protein)-like 1
Erbb2, 3	erb-b2 receptor tyrosine kinase 2, 3
Ereg	epiregulin
ERK1,2	mitogen activated protein kinase 1, 3 (Mapk1, 3)
Ermard	ER membrane-associated RNA degradation
Ero1l	ERO1-like
Erp29	endoplasmic reticulum protein 29
Errfi1	ERBB receptor feedback inhibitor 1
Espl1	extra spindle pole bodies homolog 1
Esrra	estrogen related receptor, alpha
Etfa	electron-transfer-flavoprotein, alpha polypeptide
Ets2	v-ets erythroblastosis virus E26 oncogene homolog 2
Etv1, 3	ets variant transcription factor 1, 3
Eva1c	eva-1 homolog C
Exoc3l4	exocyst complex component 3-like 4
Extl2	exostoses (multiple)-like 2
Ezr	ezrin
F3	coagulation factor III (thromboplastin, tissue factor)
Faap20	FA core complex associated protein 20
Fabp3, 5	fatty acid binding protein 3 (muscle and heart), 5 (epidermal)
Faf1	Fas (TNFRSF6) associated factor 1
Fah	fumarylacetoacetate hydrolase
Fahd1	fumarylacetoacetate hydrolase domain containing 1
Fam102b, 110a, 120c	family with sequence similarity 102 member B, 110 member A, 120C
Fam129a, 131b, 13a	family with sequence similarity 129 member A, 131 member B, 13 member A
Fam162a, 180a, 195b	family with sequence similarity 162 member A, 180 member A, 195 member B
Fam227b, 229b, 25a	family with sequence similarity 227 member B, 229 member B, 25 member A
Fam43a,50a,53a,57b	family with sequence similarity 43 member A, 50 member A, 53 member A, 57 member B
Fam65b, 71f1, 83d	family with sequence similarity 65 member B, 71 member F1, 83 member D
Farsb	phenylalanyl-tRNA synthetase, beta subunit
Faslg	Tumor necrosis factor ligand superfamily member 6, CD95L
Fasn	fatty acid synthase
Fat4	FAT tumor suppressor homolog 4
Faxdc2	fatty acid hydroxylase domain containing 2
Fbln2, 5	fibulin 2, 5
Fbxl4	F-box and leucine-rich repeat protein 4
Fbxo21, 23, 4	F-box protein 21, only protein 23, protein 4
Fbxw9	F-box and WD repeat domain containing 9
Fcgbpl1	Fc fragment of IgG binding protein-like 1
Fdps	farnesyl diphosphate synthase
Fem1b	fem-1 homolog b
FGFR1, 3, 4	Fibroblast growth factor receptor 1, 3, 4
Fggy	FGGY carbohydrate kinase domain containing
Fhl1	four and a half LIM domains 1
Fibin	fin bud initiation factor homolog (zebrafish)
Fkbp5, 7	FK506 binding protein 5, 7
Fkrp	fukutin related protein
Flad1	flavin adenine dinucleotide synthetase
Flt3	fms-related tyrosine kinase 3
Fmo3	flavin containing monooxygenase 3
Fn	fibronectin
Fos	FBJ osteosarcoma oncogene
Fosl1	fos-like antigen 1
Foxe1, f1, k1, m1, s1	forkhead box E1 (thyroid transcription factor 2), F1, K1, M1, S1
Foxo3, 4	forkhead box O3, O4
Fst	follistatin

Table S10 continued

**Symbol**	**Full name**
Fut8	fucosyltransferase 8 (alpha (1,6) fucosyltransferase)
Fxyd1	FXYD domain-containing ion transport regulator 1
Fzd1, 6	frizzled family receptor 1, 6
Gadd45a, b, g	growth arrest and DNA-damage-inducible, alpha, beta, gamma
Gal3st4	galactose-3-O-sulfotransferase 4
Gale	UDP-galactose-4-epimerase
Galnt11, 16, 2	UDP-N-acetyl-alpha-D-galactosamine:polypeptide N-acetylgalactosaminyltransferase 11, 16, 2
Gart	phosphoribosylglycinamide formyltransferase
Gas5, 6	growth arrest specific 5, 6
Gbp5	guanylate binding protein 5
Gcat	glycine C-acetyltransferase
Gchfr	GTP cyclohydrolase I feedback regulator
Gcsh	glycine cleavage system protein H (aminomethyl carrier)
Gdf15	growth differentiation factor 15
Gemin4	gem (nuclear organelle) associated protein 4
Gfra2	GDNF family receptor alpha 2
Gga1	golgi associated, gamma adaptin ear containing, ARF binding protein 1
Ggh	gamma-glutamyl hydrolase (conjugase, folylpolygammaglutamyl hydrolase)
Ghitm	growth hormone inducible transmembrane protein
Gins1, 4	GINS complex subunit 1 (Psf1 homolog), subunit 4 (Sld5 homolog)
Gja4	gap junction protein, alpha 4
Gli2	GLI family zinc finger 2
Glipr1	GLI pathogenesis-related 1
Glrx1	glutaredoxin 1
Gls2	glutaminase 2 (liver, mitochondrial)
Glt8d1, 2	glycosyltransferase 8 domain containing 1, 2
Gltp	glycolipid transfer protein
Glul	glutamate-ammonia ligase
GM3	ganglioside 3
Gmeb1	glucocorticoid modulatory element binding protein 1
Gmfb	glia maturation factor, beta
Gmnn	geminin
Gna14	guanine nucleotide binding protein, alpha 14
Gng5	guanine nucleotide binding protein (G protein), gamma 5
Gnl3	guanine nucleotide binding protein-like 3 (nucleolar)
Gns	glucosamine (N-acetyl)-6-sulfatase
Got1	glutamic-oxaloacetic transaminase 1, soluble (aspartate aminotransferase 1)
Gpatch11	coiled-coil domain containing 75
Gpcpd1	glycerophosphocholine phosphodiesterase GDE1 homolog
Gpnmb	glycoprotein (transmembrane) nmb
Gpr162, 88, 89	G protein-coupled receptor 162, 88, 89
Gprasp2	G protein-coupled receptor associated sorting protein 2
Gprc5b	G protein-coupled receptor, family C, group 5, member B
Gpsm1, 2	G-protein signaling modulator 1, 2
Gpx1, 7	glutathione peroxidase 1, 7
Gramd1b	GRAM domain containing 1B
Grem1	gremlin 1
Grin2d	glutamate receptor, ionotropic, N-methyl D-aspartate 2D
Grm6	glutamate metabotropic receptor 6
Gsn	gelsolin
Gsta3, 4, 5, 6	glutathione S-transferase A3, A4, A5, A6
Gstm1, 2, 3, 5	glutathione S-transferase mu 1, 2, 7, 5
Gtf2e1	general transcription factor IIE, polypeptide 1 (alpha subunit)
Gtpbp2	GTP binding protein 2
Gtse1	G-2 and S-phase expressed 1
Guf1	GUF1 GTPase homolog
Gusb	glucuronidase, beta
Gzmc	granzyme C
H1f0	H1 histone family, member 0
H2afj, x	H2A histone family, member J, member X
H2ai, 2ail1	similar to histone 1, H2ai, H2ai-like
H2h3c2, 3f3b	histone cluster 2 H3c2, H3 family 3B
Hadh	hydroxyacyl-CoA dehydrogenase
Hadhb	hydroxyacyl-CoA dehydrogenase/3-ketoacyl-CoA thiolase/enoyl-CoA hydratase, beta subunit
Hapln3	hyaluronan and proteoglycan link protein 3
Hars2	histidyl-tRNA synthetase 2-like
Has1	hyaluronan synthase 1
Haus8	HAUS augmin-like complex, subunit 8
Havcr1, 2	hepatitis A virus cellular receptor 1, 2 (Tim1, 3)
Hax1	HCLS1 associated protein X-1
Hbegf	heparin-binding EGF-like growth factor
Hbs1l	Hbs1-like
Hdc	histidine decarboxylase
Hebp1	heme binding protein 1
Herpud1	homocysteine-inducible, endoplasmic reticulum stress-inducible, ubiquitin-like domain member 1

Table S10 continued

**Symbol**	**Full name**
Herpud2	HERPUD family member 2
Hes7	hairy and enhancer of split 7
Hexb	hexosaminidase B
Hexim1	hexamethylene bis-acetamide inducible 1
Hid1	similar to CG8841-PA
Hif1a	hypoxia-inducible factor 1, alpha subunit (basic helix-loop-helix transcription factor)
Higd1a	HIG1 hypoxia inducible domain family, member 1A
Hip1r	huntingtin interacting protein 1 related
Hist1h1a, b, c, d	histone cluster 1, H1a, H1b, H1 VAR.1, H1d
Hist1h2af, i, il, k, n	histone cluster 1, H2af, H2ai, H2ai-like, H2ak, H2an
Hist1h2bd, h, k, l, m	histone cluster 1, H2bm, H2bh, H2bc, H2bl, H2bm
Hist1h4b	histone cluster 1, H4b
Hist2h2ac, be	histone cluster 2, H2ac, H2be
Hist2h3c, 3c2, h4	histone cluster 2, H3c, H3c2, H4
Hist3h3, 4h4	similar to histone cluster 1 H2ai, cluster 4H4
Hk2	hexokinase 2
Hmga1, b3	high mobility group AT-hook 1, box 3
Hmgcr	3-hydroxy-3-methylglutaryl-CoA reductase
Hmgcs1	3-hydroxy-3-methylglutaryl-CoA synthase 1 (soluble)
Hmx1	H6 family homeobox 1
hnRNP	heterogeneous nuclear ribonucleoprotein
Homer2	homer homolog 2 (Drosophila)
Hopx	HOP homeobox
Hp	haptoglobin
Hpn	hepsin
Hps4	Hermansky-Pudlak syndrome 4 homolog (human)
Hrasls	HRAS-like suppressor
Hs6st1	heparan sulfate 6-O-sulfotransferase 1
Hsbp1	heat shock factor binding protein 1
Hsd17b8	hydroxysteroid (17-beta) dehydrogenase 8
Hsdl2	hydroxysteroid dehydrogenase like 2
Hsp70	heat shock protein70
Hspa1b, 5, 8, 9	heat shock 70kD protein 1B, 5, 8, 9
Htatip2	HIV-1 tat interactive protein 2, homolog (human)
Hyal2	hyaluronoglucosaminidase 2
Hyou1	hypoxia up-regulated 1
Iars	isoleucyl-tRNA synthetase
Icam1, 5	intercellular adhesion molecule 1, 5
Id1, 2, 4	inhibitor of DNA binding 1, 2, 4
Idi1	isopentenyl-diphosphate delta isomerase 1
Ids	iduronate 2-sulfatase
Ier2, 3, 5, 5l	immediate early response 2, 3, 5, 5-like
Ifi27l2b	interferon, alpha-inducible protein 27 like 2B
Ifi35	interferon-induced protein 35
Ifit2, 3	interferon-induced protein with tetratricopeptide repeats 2, 3
Ifitm1	interferon induced transmembrane protein 1
Ifng	Interferon gamma
Ifngr2	interferon gamma receptor 2
Ifrd1	interferon-related developmental regulator 1
Ift88	intraflagellar transport 88 homolog (Chlamydomonas)
Igf1r, 2r	insulin-like growth factor 1, 2 receptor
Igf2bp2	insulin-like growth factor 2 mRNA binding protein 2
Igfbp6	insulin-like growth factor binding protein 6
Il11ra1, 13ra2	interleukin 11 receptor alpha chain 1, 13 receptor alpha 2
Il13, 16, 23a, 34	Interleukin 13, 16, 23 alpha subunit p19, 34
Il1a, 1b, f10, 2, 4, 6, 10	Interleukin 1 alpha, 1 beta, 1 family member 10, 2, 4, 6, 10
Il1r2, rl1, r6	interleukin 1 receptor, type II, receptor-like 1, 6 receptor
Il1rn	interleukin 1 receptor antagonist
Ilf3	interleukin enhancer binding factor 3
Incenp	inner centromere protein
Inip	similar to HSPC043 protein
Insig1	insulin induced gene 1
Insr	insulin receptor
Ip6k1	inositol hexakisphosphate kinase 1
Ipo5	importin 5
Iqgap1	IQ motif containing GTPase activating protein 1
Irak3	interleukin-1 receptor-associated kinase 3
Irf7	interferon regulatory factor 7
Irs1	insulin receptor substrate 1
Irx2	iroquois homeobox 2
Isg15	ISG15 ubiquitin-like modifier
Isg20	interferon stimulated exonuclease gene 20
Isx	similar to Retinal homeobox protein Rx (DRx1) (DRx)
Isy1	ISY1 splicing factor homolog
Isyna1	inositol-3-phosphate synthase 1

Table S10 continued

Symbol	Full name
Itga1, 10, 8	integrin, alpha 1, 10, 8
Itgb1, 5, bl1	integrin, beta 1, 5, beta-like 1
Itm2b, 2c	integral membrane protein 2B, 2C
Itpkc	inositol-trisphosphate 3-kinase C
Izumo4	IZUMO family member 4
Jade1	PHD finger protein 17
Jag1	jagged 1
Jnk	mitogen-activated protein kinase 8 (Mapk8)
Jpt1	Jupiter microtubule associated homolog 1
Jsrp1	junctional sarcoplasmic reticulum protein 1
Junb	jun B proto-oncogene
Jup	junction plakoglobin
Kazald1	Kazal-type serine peptidase inhibitor domain 1
Kcmf1	potassium channel modulatory factor 1
Kcne2, k2	potassium voltage-gated channel, Isk-related family member 2, subfamily K member 2
Kcp	kielin/chordin-like protein
Kctd13	potassium channel tetramerisation domain containing 13
Kdelc2, r3	KDEL (Lys-Asp-Glu-Leu) containing 2, endoplasmic reticulum protein retention receptor 3
Kdm6b	lysine (K)-specific demethylase 6B
Keap1	kelch like ECH associated protein 1
Kif11, 18a, 20a, 22, 23	kinesin family member 11, 18a, 20a, 22, 23
Kifc1, c2, c3	kinesin family member C1, C2, C3
Kit	KIT proto-oncogene receptor tyrosine kinase
Kitlg	KIT ligand
Klc1	kinesin light chain 1
Klf10, 2, 4, 9	Kruppel-like factor 10, 2 (lung), 4 (gut), 9
Klhl21, 36	kelch-like 21, 36
Kntc1	kinetochore associated 1
Kpna1, a2	karyopherin alpha 1, alpha 2
Kprp	keratinocyte proline-rich protein
Kras	Kras proto-oncogene GTPase
Krtap16-5, 21-2	keratin associated protein 16-5, 21-2
Lamc2	laminin, gamma 2
Laptm4a, 4b	lysosomal protein transmembrane 4 alpha, 4 beta
Lars	leucyl-tRNA synthetase
Lbh	limb bud and heart development
Lbp	lipopolysaccharide binding protein
Lc17	myosin, light polypeptide 6, alkali, smooth muscle and non-muscle-like
Lcat	lecithin cholesterol acyltransferase
Lck	LCK proto-oncogene, Src family tyrosine kinase
Lcn2	lipocalin 2
Ldlr	low density lipoprotein receptor
Lep	Leptin
Leprel4	leprecan-like 4
Letm1	leucine zipper-EF-hand containing transmembrane protein 1
Lgals1, 5, 9	lectin, galactoside-binding, soluble, 1, 5, 9
Lgmn	legumain
Lhfpl2	lipoma HMGIC fusion partner-like 2
Lims2	LIM and senescent cell antigen like domains 2
Lmbrd1	LMBR1 domain containing 1
Lnp	limb and neural patterns
Lnx1	ligand of numb-protein X 1
LOC100125364	hypothetical protein LOC100125364
LOC100359930	Cyp2s1 protein-like
LOC100360880	FBJ osteosarcoma oncogene B
LOC100361571	keratin associated protein 1-3-like
LOC103691261	similar to Sperm flagellar protein 1
LOC257650	hippyragranin
LOC301748	similar to RIKEN cDNA 1700001E04
LOC360713	similar to pleckstrin homology-like domain, family B, member 2
LOC361914	similar to solute carrier family 7 (cationic amino acid transporter, y+ system), member 12
LOC498154	hypothetical protein LOC498154
LOC498675	hypothetical LOC498675
LOC499219	hypothetical protein LOC499219
LOC499843	LRRGT00091
LOC500028	hypothetical protein LOC500028
LOC500035	hypothetical protein LOC500035
LOC500227	hypothetical gene supported by BC079424
LOC500846	hypothetical protein LOC500846
LOC500959	triosephosphate isomerase
LOC678708	similar to histone 1, H2ai
LOC680322	similar to Histone H2A type 1
LOC680578	similar to C56C10.7a
LOC681382	hypothetical protein LOC681382
LOC684998	hypothetical protein LOC684998

Table S10 continued

Symbol	Full name
LOC688459	hypothetical protein LOC688459
LOC689065	hypothetical protein LOC689065
LOC689412	similar to CG4025-PA
LOC689574	hypothetical protein LOC689574
LOC689959	hypothetical protein LOC689959
LOC690386	hypothetical protein LOC690386
LOC690415	hypothetical protein LOC690415
Lonp1	lon peptidase 1, mitochondrial
Lox	lysyl oxidase
Loxl1, 2	lysyl oxidase-like 1, 2
Lpar1, 6	lysophosphatidic acid receptor 1, 6
Lrif1	ligand dependent nuclear receptor interacting factor 1
Lrp11	low density lipoprotein receptor-related protein 11
Lrrc8b	leucine rich repeat containing 8 family, member B
Lsm14a	LSM14A, SCD6 homolog A
Ltbp3	latent transforming growth factor beta binding protein 3
Luc7l	LUC7-like
Lum	lumican
Lurap1l	leucine rich adaptor protein 1-like
Lvrn	laeverin
Ly6g5c	lymphocyte antigen 6 complex, locus G5C
Lypla2	lysophospholipase 2
Lyrm9	LYR motif containing 9
Lysmd2	LysM, putative peptidoglycan-binding, domain containing 2
Lztfl1	leucine zipper transcription factor-like 1
Mafk	v-maf musculoaponeurotic fibrosarcoma oncogene homolog K
Mag	myelin-associated glycoprotein
Maged2	melanoma antigen, family D, 2
Manba	mannosidase, beta A, lysosomal
Map2k1, 3k6	mitogen activated protein kinase kinase 1, 6
Map6, 7	microtubule-associated protein 6, 7
Mapk1, 9, 14	mitogen-activated protein kinase-1, 9, 14 (p38)
Mapkapk3	mitogen-activated protein kinase-activated protein kinase 3
Mapre2	microtubule-associated protein, RP/EB family, member 2
March2	membrane-associated ring finger (C3HC4) 2
Marcksl1	MARCKS-like 1
Mars	methionine-tRNA synthetase
Mat2b	methionine adenosyltransferase II, beta
Mbtps1	membrane-bound transcription factor peptidase, site 1
Mccrip2	family with sequence similarity 195, member A
Mcl1	myeloid cell leukemia sequence 1
Mcm3, 4, 5, 6	minichromosome maintenance complex component 3,4, 5, 6
Mcoln1	mucolipin 1
Mcub	coiled-coil domain containing 109B
Mdm2	Mdm2 p53 binding protein homolog (mouse)
MDR1a	ATP-binding cassette, sub-family B (MDR/TAP), member 1A
Me1	malic enzyme 1, NADP(+)-dependent, cytosolic
Mecp2	methyl CpG binding protein 2
Med11, 18, 23	mediator complex subunit 11, 18, 23
Medag	mesenteric estrogen-dependent adipogenesis
Medag	mesenteric estrogen-dependent adipogenesis
Mef2b	myocyte enhancer factor 2B
Melk	maternal embryonic leucine zipper kinase
Meox2	mesenchyme homeobox 2
Mesdc2	mesoderm development candidate 2
Met	MET proto-oncogene, receptor tyrosine kinase
Metrnl	meteorin, glial cell differentiation regulator-like
Mettl11a, 22, 23, 3, 7b	methyltransferase like 11A, 22, 23, 3, 7b
Mex3b	mex3 homolog B (C. elegans)
Mfge8	milk fat globe-EGF factor 8
Mgarp	mitochondria-localized glutamic acid-rich protein
Mgat1	mannosyl (alpha-1,3-)-glycoprotein beta-1,2-N-acetylglucosaminyltransferase
MGC105649	hypothetical LOC302884
Mgea5	meningioma expressed antigen 5 (hyaluronidase)
Mgst3	microsomal glutathione S-transferase 3
Micos13	mitochondrial contact site and cristae organizing system subunit 13
Micu1	mitochondrial calcium uptake 1
Mid1ip1	MID1 interacting protein 1 (gastrulation specific G12 homolog (zebrafish))
Mif4gd	MIF4G domain containing
Mis18a	MIS18 kinetochore protein homolog A (S. pombe)
Mlf1ip	myeloid leukemia factor 1 interacting protein
Mmaa	methylmalonic aciduria (cobalamin deficiency) cblA type
Mme	membrane metallo-endopeptidase
Mmp11, 15, 2, 28, 8, 9	matrix metallopeptidase 11, 15, 2, 28, 8 (neutrophil collagenase), 9
Mms22l	MMS22-like, DNA repair protein

Table S10 continued

**Symbol**	**Full name**
Mnt	max binding protein
Mogat2	monoacylglycerol O-acyltransferase 2
Mok	MOK protein kinase
Mon1b	MON1 homolog b (yeast)
Morf4l2	mortality factor 4 like 2
Mospd1	motile sperm domain containing 1
Mphosph6	M phase phosphoprotein 6
Mpp5	membrane protein, palmitoylated 5 (MAGUK p55 subfamily member 5)
Mpv17l2	MPV17 mitochondrial membrane protein-like 2
Mras	muscle RAS oncogene homolog
Mrc2	mannose receptor, C type 2
Mrgprf	MAS-related GPR, member F
Mri1	methylthioribose-1-phosphate isomerase homolog (S. cerevisiae)
Mrnip	MRN complex interacting protein
Mrpl11, 17, 38, 55	mitochondrial ribosomal protein L11, 17, 38, 55
Mrps12, 17, 18b, 21	mitochondrial ribosomal protein S12, 17, 18b, 21
Mrps21l	mitochondrial ribosomal protein S21-like
Mrvi1	murine retrovirus integration site 1 homolog
Ms4a7	membrane-spanning 4-domains, subfamily A, member 7
Mst1r	macrophage stimulating 1 receptor
Mt1a	metallothionein 1a
Mtbp	MDM2 binding protein
Mtch2	mitochondrial carrier 2
Mthfd1, 2	methylenetetrahydrofolate dehydrogenase 1, 2
Mtpap	mitochondrial poly(A) polymerase
Mus81	MUS81 endonuclease homolog
Mvd	mevalonate (diphospho) decarboxylase
Mx1	myxovirus (influenza virus) resistance 1
Mxd1, 4	max dimerization protein 1, 4
Mybl2	myeloblastosis oncogene-like 2
Myc	myelocytomatosis oncogene
Mydgf	myeloid-derived growth factor
Myh1, 8	myosin, heavy polypeptide 1 (skeletal muscle, adult), 8 (skeletal muscle, perinatal)
Myhc	myosin, heavy polypeptide 1, skeletal muscle, adult
Myl6	myosin, light chain 6, alkali, smooth muscle and non-muscle
Mylk	myosin light chain kinase
Mylk3	myosin light chain kinase 3
Myo1f, 5a	myosin IF, VA
Mzf1	myeloid zinc finger 1
N4bp2l1	NEDD4 binding protein 2-like 1
Naglu	N-acetyl-alpha-glucosaminidase
Nampt	nicotinamide phosphoribosyltransferase
Napsa	napsin A aspartic peptidase
Narf	nuclear prelamin A recognition factor
Nars	asparaginyl-tRNA synthetase
Nat14	N-acetyltransferase 14
Nbr1	neighbor of Brca1 gene 1
Ncaph	non-SMC condensin I complex, subunit H
Nck2	NCK adaptor protein 2
Nckipsd	NCK interacting protein with SH3 domain
Ndc80	NDC80 homolog, kinetochore complex component
Nde1	nudE nuclear distribution gene E homolog 1
Ndrg2, 4	N-myc downstream regulated gene 2, 4
Ndufa4	NADH dehydrogenase (ubiquinone) 1 alpha subcomplex, 4
Ndufaf6	NADH:ubiquinone oxidoreductase complex assembly factor 6
Nedd4	neural precursor cell expressed, developmentally down-regulated 4
Nek6	NIMA (never in mitosis gene a)-related kinase 6
Nelf	nasal embryonic LHRH factor
Nes	nestin
Neu1	sialidase 1 (lysosomal sialidase)
Nfat5	nuclear factor of activated T-cells 5
Nfe2l1, l2	nuclear factor, erythroid derived 2,-like 1, like 2
Nfil3	nuclear factor, interleukin 3 regulated
Nfkb1	nuclear factor kappa B subunit 1
Nfkbiz	nuclear factor of kappa light polypeptide gene enhancer in B-cells inhibitor, zeta
Nfu1	NFU1 iron-sulfur cluster scaffold homolog (S. cerevisiae)
Nfyb	nuclear transcription factor-Y beta
Ngdn	neuroguidin, EIF4E binding protein
NGF	nerve growth factor
Nid2	nidogen 2
Ninj1	ninjurin 1
Nkain1	Na+/K+ transporting ATPase interacting 1
Nkx6-3	NK6 homeobox 3
Nmnat3	nicotinamide nucleotide adenylyltransferase 3
Nmrk1	similar to Nicotinamide riboside kinase 1

Table S10 continued

Symbol	Full name
Nnat	neuronatin
Nol8, 14, 58	nucleolar protein 8, 14, 58
Nox1, 4	NADPH oxidase 1, 4
Npap60	nuclear pore associated protein
Npas2	neuronal PAS domain protein 2
Npc1	Cdig2 protein
Npepl1	aminopeptidase-like 1
Nphp1	nephronophthisis 1 (juvenile) homolog (human)
Nr1d1, 2f1, 2f2,	nuclear receptor subfamily 1, group D, member 1, 2, group F, member 1, 2, group F, member 2
Nr4a1, 2, 3	nuclear receptor subfamily 4, group A, member 1, 2, 3
Nras	NRAS proto-oncogene, GTPase
Nrf1, 2	nuclear respiratory factor 1, 2
Nrg1	neuregulin 1
Nrm	nurim (nuclear envelope membrane protein)
Ns5atp9	NS5A (hepatitis C virus) transactivated protein 9
Nsd1, 2	nuclear receptor binding SET domain protein 1, Wolf-Hirschhorn syndrome candidate 1
Nsun4	NOP2/Sun domain family, member 4
Ntrk1, 2	neurotrophic receptor tyrosine kinase 1, 2
Nudt1, 12, 14, 15, 6, 9	nudix (nucleoside diphosphate linked moiety X)-type motif 1, 12, 14, 15, 6, 9
Nuf2	NUF2, NDC80 kinetochore complex component, homolog
Nup54	nucleoporin 54
Nusap1	nucleolar and spindle associated protein 1
Nxn	nucleoredoxin
Nxph3	neurexophilin 3
Oas1e, l2	2\'-5\' oligoadenylate synthetase 1E, like 2
Oat	ornithine aminotransferase
Oaz2	ornithine decarboxylase antizyme 2
Obfc1	oligonucleotide/oligosaccharide-binding fold containing 1
Oct4/Pou5f1	Pou class 5 homeobox 1
Odc1	ornithine decarboxylase 1
Ogn	osteoglycin
Ogt	O-linked N-acetylglucosamine (GlcNAc) transferase
Olfml1, 2a, 3	olfactomedin-like 1, 2a, 3
Omd	osteomodulin
Optn	optineurin
Oser1	oxidative stress responsive gene
Osr2	odd-skipped related 2 (Drosophila)
Otub2	OTU domain, ubiquitin aldehyde binding 2
Otx1	orthodenticle homeobox 1
Ovol1	ovo-like 1(Drosophila)
P2ry2	purinergic receptor P2Y, G-protein coupled, 2
P3h1	prolyl 3-hydroxylase 1
Pak1ip1	PAK1 interacting protein 1
Paox	polyamine oxidase (exo-N4-amino)
Papola	poly (A) polymerase alpha
Pappa1	pregnancy-associated plasma protein A
Papss2	3\'-phosphoadenosine 5\'-phosphosulfate synthase 2
Parp1, 14	poly (ADP-ribose) polymerase family, member 1, 14
Parvb	parvin, beta
Patz1	POZ (BTB) and AT hook containing zinc finger 1
Pawr	PRKC, apoptosis, WT1, regulator
Pbk	PDZ binding kinase
Pcdh19, ac2, b19	protocadherin 19, alpha subfamily C 2, beta 19
Pcdhga7, gc5	protocadherin gamma subfamily A7, C5
Pck2	phosphoenolpyruvate carboxykinase 2 (mitochondrial)
Pcolce	procollagen C-endopeptidase enhancer
Pcp4l1	Purkinje cell protein 4-like 1
Pcsk4	proprotein convertase subtilisin/kexin type 4
Pdcd10, 6	programmed cell death 10, 6
Pdgfa	Platelet derived growth factor subunit A
Pdgfra	platelet derived growth factor receptor, alpha polypeptide
Pdgfrl	platelet-derived growth factor receptor-like
Pdk1, 2	pyruvate dehydrogenase kinase, isozyme 1, 2
Pdlim5	PDZ and LIM domain 5
Pdp1	pyruvate dehyrogenase phosphatase catalytic subunit 1
Pdrg1	p53 and DNA damage regulated 1
Pdx1	pancreatic and duodenal homeobox 1
Pdxp	pyridoxal (pyridoxine, vitamin B6) phosphatase
Pex13	peroxisomal biogenesis factor 13
Pfkm, p	phosphofructokinase muscle, platelet
Pgpep1	pyroglutamyl-peptidase I
Phf19	PHD finger protein 19
Phgdh	phosphoglycerate dehydrogenase
Phlda1	pleckstrin homology-like domain, family A, member 1
Pigp	phosphatidylinositol glycan anchor biosynthesis, class P

Table S10 continued

Symbol	Full name
Pik3r1	phosphoinositide-3-kinase, regulatory subunit 1 alpha (PI3K-p85))
Pim1, 3	pim-1 oncogene, 3
Pimreg	PICALM interacting mitotic regulator
Pip4p1	phosphatidylinositol-4,5-bisphosphate 4-phosphatase 1
Pip5k1c	phosphatidylinositol-4-phosphate 5-kinase, type I, gamma
Pitx1	paired-like homeodomain 1
Pkca	protein kinase C alpha
Pknox1	PBX/knotted 1 homeobox 1
Pkp1, 2	plakophilin 1, 2
Pla2g16, 2a, 2c	phospholipase A2, group XVI, group IIA (platelets, synovial fluid), group IIC
Plekha4, g5	pleckstrin homology domain containing, fam.A (phosphoinositide bind.) 4, fam.G (with RhoGef dom.) 5
Plk1, 2, 3, 4	polo-like kinase 1, 2, 3, 4
Pls3	plastin 3
Plscr2	phospholipid scramblase 2
Pnn	pinin, desmosome associated protein
Pnpla2	patatin-like phospholipase domain containing 2
Pnrc1, 2	proline-rich nuclear receptor coactivator 1, 2
Podnl1	podocan-like 1
Podxl2	podocalyxin-like 2
Pofut2	protein O-fucosyltransferase 2
Pold1, g2, m	polymerase (DNA directed), delta 1 catalytic subunit, gamma 2, accessory subunit, mu
Polr1a, 3gl	polymerase (RNA) I polypeptide A, III (DNA directed) polypeptide G-like
Pomgnt2	protein O-linked mannose N-acetylglucosaminyltransferase 2 (beta 1,4-)
Pon2	paraoxonase 2
Postn	periostin, osteoblast specific factor
Ppa2	pyrophosphatase (inorganic) 2
Ppap2b	phosphatidic acid phosphatase type 2B
Ppat	phosphoribosyl pyrophosphate amidotransferase
Ppih	peptidylprolyl isomerase H (cyclophilin H)
Ppp1cb	protein phosphatase 1, catalytic subunit, beta isozyme
Ppp1r14a, 15a, 2,,32	protein phosphatase 1, regul. (inhib.) sub.14A, regul. sub.15A, regul. (inhib.) sub.2, regul. sub.32
Ppp2r3b	protein phosphatase 2, regulatory subunit B", beta
Ppt1	palmitoyl-protein thioesterase 1
Prag1	pragma of Rnd2
Prdx4, 5	peroxiredoxin 4, 5
Prelp	proline/arginine-rich end leucine-rich repeat protein
Prepl	prolyl endopeptidase-like
Prkacb	protein kinase, cAMP dependent, catalytic, beta
Prkcd	protein kinase C delta
Prl	Prolactin
Prmt1, 5, 7	protein arginine methyltransferase 1, 5, 7
Proser2	proline and serine rich 2
Prpf40b, 4b	pre-mRNA processing factor 40 homolog B, 4 homolog B
Prr7, c2c	proline rich 7 (synaptic), coiled-coil 2C
Prss23	protease, serine, 23
PS	phosphatidylserine
Psap	prosaposin
Psat1	phosphoserine aminotransferase 1
Psd	pleckstrin and Sec7 domain containing
Psen2	presenilin 2
Psip1	PC4 and SFRS1 interacting protein 1
Psmb10, e4	proteasome (prosome, macropain) subunit, beta type 10, activator subunit 4
Psph	phosphoserine phosphatase
Ptcd2	pentatricopeptide repeat domain 2
Pten	phosphatase and tensin homolog
Ptges2, is	prostaglandin E synthase 2, I2 (prostacyclin) synthase
Pth2	parathyroid hormone 2
Ptms	parathymosin
Ptn	pleiotrophin
Ptp4a1	protein tyrosine phosphatase type IVA, member 1
Ptpdc1	protein tyrosine phosphatase domain containing 1
Ptpn12, 2	protein tyrosine phosphatase, non-receptor type 12, 2
Ptprf, m, s	protein tyrosine phosphatase, receptor type, F, M, S
Ptrhd1	peptidyl-tRNA hydrolase domain containing 1
Pttg1	pituitary tumor-transforming 1
Ptx3	pentraxin 3, long
Purb	purine rich element binding protein B
Pvr	PVR cell adhesion molecule (poliovirus receptor)
Pxn	paxillin
Pyroxd2	pyridine nucleotide-disulphide oxidoreductase domain 2
Qsox2	quiescin Q6 sulfhydryl oxidase 2
R3hdm1	R3H domain containing 1
Rab11fip5	similar to RAB11 family interacting protein 5 (class I) isoform 1
Rab26, 32	RAB26, 32, member RAS oncogene family
Rab3il1	RAB3A interacting protein (rabin3)-like 1

Table S10 continued

Symbol	Full name
Rabggtb	Rab geranylgeranyltransferase, beta subunit
Rad23a, 52, 54l, 9b	RAD23 homolog A, RAD52 homolog, 54 like, 9 homolog B
Rae1	RAE1 RNA export 1 homolog
Rage/Ager	advanced glycosylation end-product specific receptor
Rai12, 14	retinoic acid induced 12, 14
Rala	v-ral simian leukemia viral oncogene homolog A (ras related)
Ramp3	receptor (G protein-coupled) activity modifying protein 3
Rangrf	RAN guanine nucleotide release factor
Rap1a	RAP1A, member of RAS oncogene family
Rara	retinoic acid receptor, alpha
Rars2	arginyl-tRNA synthetase 2, mitochondrial
Rasl11b, 12	RAS-like family 11 member B, family 12
Rassf2, 5	Ras association (RalGDS/AF-6) domain family member 2, 5
Rb1	retinoblastoma 1
Rb1cc1	RB1-inducible coiled-coil 1
Rbfox1	RNA binding protein, fox-1 homolog 1
Rbm12, 28, 3, 38, x	RNA binding motif protein 12, 28, 3 (RNP1, RRM), 38, X-linked
Rbp1, 2	retinol binding protein 1 (cellular), 2 (cellular)
Rc3h2	ring finger and CCCH-type domains 2
Rcc2	regulator of chromosome condensation 2
Rcn3	reticulocalbin 3, EF-hand calcium binding domain
Rcor1	REST corepressor 1
Reck	reversion-inducing-cysteine-rich protein with kazal motifs
Rell2	RELT-like 2
Renbp	renin binding protein
Ret	ret proto-oncogene
Rfc3	replication factor C (activator 1) 3
Rftn1	raftlin lipid raft linker 1
RGD1304587	similar to RIKEN cDNA 2310033P09
RGD1304624	similar to RIKEN cDNA 2700097O09
RGD1305938	similar to expressed sequence AW549877
RGD1306227	similar to 4833420G17Rik protein
RGD1307929	similar to CG14967-PA
RGD1308117	similar to 9930012K11Rik protein
RGD1309104	similar to RIKEN cDNA 1700025G04 gene
RGD1309534	similar to RIKEN cDNA 4931406C07
RGD1311739	similar to RIKEN cDNA 1700037H04
RGD1311946	similar to RIKEN cDNA 1810055G02
RGD1560073	similar to ribosomal protein S10
RGD1560617	hypothetical gene supported by NM_053561; AF062594
RGD1561381	similar to microsomal glutathione S-transferase 3
RGD1561671	similar to RIKEN cDNA 2900010M23
RGD1562114	RGD1562114
RGD1562136	similar to D1Ertd622e protein
RGD1562378	histone H4 variant H4-v.1
RGD1563034	similar to ETS domain transcription factor ERF (Ets2 repressor factor)
RGD1563378	similar to ferritin heavy polypeptide-like 17
RGD1564664	similar to LOC387763 protein
RGD1564712	RGD1564712
RGD1565033	similar to hypothetical protein LOC284018 isoform b
RGD1565170	similar to 60S ribosomal protein L23a
RGD1566265	similar to RIKEN cDNA 2610002M06
Rgma	RGM domain family, member A
Rgs2, 3	regulator of G-protein signaling 2, 3
Rhbdf2	rhomboid 5 homolog 2
Rhob	ras homolog gene family, member B
Riok3	RIO kinase 3
Ripk3	receptor-interacting serine-threonine kinase 3
Rmrp	RNA component of mitochondrial RNA processing endoribonuclease
Rnase4	ribonuclease, RNase A family 4
Rnf141, 34	ring finger protein 141, 34
Rnpepl1	arginyl aminopeptidase (aminopeptidase B)-like 1
Romo1	reactive oxygen species modulator 1
Rp4	RGD1559532
Rpa3	replication protein A3
Rpe	ribulose-5-phosphate-3-epimerase
Rpf2	ribosome production factor 2 homolog
Rpl10l	ribosomal protein L10-like
Rragd	Ras-related GTP binding D
Rrm2	ribonucleotide reductase M2
Rrp12	ribosomal RNA processing 12 homolog
Rrp9	ribosomal RNA processing 9, small subunit (SSU) processome component, homolog (yeast)
Rsad1, 2	similar to radical S-adenosyl methionine domain containing 1, 2
Rsl24d1	ribosomal L24 domain containing 1
Rspo1	R-spondin 1

Table S10 continued

**Symbol**	**Full name**
Rsrp1	similar to chromosome 1 open reading frame 63
Rsu1	Ras suppressor protein 1
Rtkn	rhotekin
Rtl8b	retrotransposon Gag like 8B
Rtp4	receptor (chemosensory) transporter protein 4
Runx1	runt-related transcription factor 1
Rwdd1	RWD domain containing 1
S100a1, 10, 13,4	S100 calcium binding protein A1, A10, A13, A4
Sacm1l	SAC1 suppressor of actin mutations 1-like
Samd4a	sterile alpha motif domain containing 4A
Sap25	Sin3A-associated protein 25
Sapcd2	suppressor APC domain containing 2
Sars	seryl-tRNA synthetase
Scaf4	SR-related CTD-associated factor 4
Scara3, 5	scavenger receptor class A, member 3, 5
Scarf2	scavenger receptor class F, member 2
Scd1	stearoyl-Coenzyme A desaturase 1
Scfd2	sec1 family domain containing 2
Scp2	sterol carrier protein 2
Sdad1	SDA1 domain containing 1
Sdcbp2	syndecan binding protein (syntenin) 2
Sdf2, 2l1	stromal cell derived factor 2, 2-like 1
Sdr39u1	short chain dehydrogenase/reductase family 39U, member 1
Selenom, w	selenoprotein M, W 1
Sema3b	sema domain, immunoglobulin domain, short basic domain, secreted, (semaphorin) 3B
Sema5a	sema d., seven thrombospondin repeats (type 1 & 1-like), transm.d., short cytopl.d., (semaphorin) 5A
Sema6c	sema domain, transmembrane domain, cytoplasmic domain, (semaphorin) 6C
Senp8	SUMO/sentrin specific peptidase family member 8
Sepp1	selenoprotein P, plasma, 1
Sept4, 7	septin 4, 7
Serac1	serine active site containing 1
Serp1	stress-associated endoplasmic reticulum protein 1
Serpinb1a, 7, e1, f1	serpin peptidase inhibitor family B member 1A, member 7, E member 1, F member 1
Sertad3, 4	SERTA domain containing 3, 4
Sesn2, 3	sestrin 2, 3
Set	SET nuclear oncogene
Sfrp2	secreted frizzled-related protein 2
Sfxn1, 4	sideroflexin 1, 4
Sgcb	sarcoglycan, beta (dystrophin-associated glycoprotein)
Sgk1	serum/glucocorticoid regulated kinase 1
Sgk196	protein kinase-like protein SgK196
Sgpp1	sphingosine-1-phosphatase
Sh3bp2	SH3-domain binding protein 2
Shbg	sex hormone binding globulin
Shc2	SHC (Src homology 2 domain containing) transforming protein 2
Shcbp1	SHC binding and spindle associated 1
Shisa4, 5, 8	shisa homolog 4, 5, 8
Shmt1, 2	serine hydroxymethyltransferase 1 (soluble), 2 (mitochondrial)
Sik1	salt-inducible kinase 1
Sin3a	SIN3 homolog A, transcription regulator (yeast)
Sipa1	signal-induced proliferation-associated 1
Sirpa	signal-regulatory protein alpha
Sirt3	sirtuin 3
Six3	SIX homeobox 3
Ska1	spindle and kinetochore associated complex subunit 1
Skil	SKI-like oncogene
Skp1	S-phase kinase-associated protein 1
Slc12a2	solute carrier family 12 member 2 (sodium/potassium/chloride transporters)
Slc16a1, a3	solute carrier family 16, member 1, 3 (monocarboxylic acid transporter 1, 4)
Slc17a5	solute carrier family 17 (anion/sugar transporter), member 5
Slc19a1	solute carrier family 19 (folate transporter), member 1
Slc1a5	solute carrier family 1 (neutral amino acid transporter), member 5
Slc20a1, a2	solute carrier family 20 (phosphate transporter), member 1, 2
Slc22a17	solute carrier family 22, member 17
Slc25a12, 25	solute carrier family 25 (mitochondrial carrier), member 12, 25 (phosphate)
Slc25a28,30,33,37,38	solute carrier family 25, member 28, 30, 33, 37, 38
Slc2a1	solute carrier family 2 (facilitated glucose transporter), member 1
Slc30a4	solute carrier family 30 (zinc transporter), member 4
Slc33a1	solute carrier family 33 (acetyl-CoA transporter), member 1
Slc35a2, f5	solute carrier family 35 (UDP-galactose transporter) member A2, member F5
Slc38a7	solute carrier family 38, member 7
Slc3a2	solute carrier family 3 (activators of dibasic and neutral amino acid transport), member 2
Slc44a1	solute carrier family 44, member 1
Slc4a2	solute carrier family 4 (anion exchanger), member 2
Slc6a4, 6, 9	solute carrier family 6 (neurotransmitter transporter), member 4 (serotonin), 6 (taurine), 9 (glycine)

Table S10 continued

Symbol	Full name
Slc7a1	solute carrier family 7 (cationic amino acid transporter, y+ system), member 1
Slc7a5	solute carrier family 7 (amino acid transporter light chain, L system), member 5
Slfn2	schlafen 2
Slirp	SRA stem-loop interacting RNA binding protein
Slit3	slit homolog 3 (Drosophila)
Slpi	secretory leukocyte peptidase inhibitor
Smad1, 4	SMAD family member 1, 4
Smarcc2	SWI/SNF related, matrix associated, actin dependent regulator of chromatin, subfamily c, member 2
Smc4	structural maintenance of chromosomes 4
Smek2	SMEK homolog 2, suppressor of mek1 (Dictyostelium)
Smg6, 9	Smg-6 , 9 homolog, nonsense mediated mRNA decay factor
Smim29, 7	small integral membrane protein 29, 7
Smndc1	survival motor neuron domain containing 1
Snap	soluble *N*-ethylmaleimide-sensitive fusion attachment protein
Snare	soluble *N*-ethylmaleimide-sensitive fusion attachment protein (SNAP) receptor
Snhg11	small nucleolar RNA host gene 11
Snn	stannin
Snrpa	small nuclear ribonucleoprotein polypeptide A
Snx12, 14, 7, 8	sorting nexin 12, 14, 7, 8
Sobp	sine oculis-binding protein homolog (Drosophila)
Socs3	suppressor of cytokine signaling 3
Sod2, 3	superoxide dismutase 2 (mitochondrial), 3 (extracellular)
Sox2, 4	SRY (sex determining region Y)-box 2, 4
Sp2	Sp2 transcription factor
Spag5	sperm associated antigen 5
Sparc	secreted protein, acidic, cysteine-rich (osteonectin)
Spc25	SPC25, NDC80 kinetochore complex component, homolog
Spcs3	signal peptidase complex subunit 3 homolog
Specc1l	sperm antigen with calponin homology and coiled-coil domains 1-like
Spef1	sperm flagellar 1
Spint2	serine peptidase inhibitor, Kunitz type, 2
Spout1	similar to LOC495800 protein
Spp1	secreted phosphoprotein 1
Sprr1a	small proline-rich protein 1A
Spsb4	splA/ryanodine receptor domain and SOCS box containing 4
Sptbn1	spectrin, beta, non-erythrocytic 1
Sqstm1	sequestosome 1
Src	SRC proto-oncogene, non-receptor tyrosine kinase
Srebf1	sterol regulatory element binding transcription factor 1
Srfbp1	serum response factor binding protein 1
Srgap2	SLIT-ROBO Rho GTPase activating protein 2
Srgn	serglycin
Srm	spermidine synthase
Srp14, 68	signal recognition particle 14, 68
Srpx, x2	sushi-repeat-containing protein, X-linked, X-linked 2
Srrd	SRR1 domain containing
Srrt	serrate, RNA effector molecule
Srsf5, 6	serine/arginine-rich splicing factor 5, 6
Srxn1	sulfiredoxin 1
Ssc5d	scavenger receptor cysteine rich domain containing (5 domains)
Ssu72	SSU72 RNA polymerase II CTD phosphatase homolog
St14	suppression of tumorigenicity 14 (colon carcinoma)
St3gal5	ST3 beta-galactoside alpha-2,3-sialyltransferase 5
St7l	suppression of tumorigenicity 7-like
Stab1	Stabilin1
Stambpl1	similar to AMSH-family protein
Star	steroidogenic acute regulatory protein
Stard4, 9	StAR-related lipid transfer (START) domain containing 4, 9
Stat1, 2, 3	signal transducer and activator of transcription 1, 2, 3
Stbd1	starch binding domain 1
Steap3, 4	STEAP family member 3, 4 (metalloreductase)
Stk11ip	serine/threonine kinase 11 interacting protein
Ston1	stonin 1
Strbp	spermatid perinuclear RNA binding protein
Strn3	striatin, calmodulin binding protein 3
Stxbp5	syntaxin binding protein 5 (tomosyn)
Styxl1	serine/threonine/tyrosine interacting-like 1
Sub1	SUB1 homolog (S. cerevisiae)
Sult1a1	sulfotransferase family, cytosolic, 1A, phenol-preferring, member 1
Sumf2	sulfatase modifying factor 2
Suox	sulfite oxidase
Surf1, 2, 4	surfeit 1, 2, 4
Suv420h1	suppressor of variegation 4-20 homolog 1
Syt	synaptogamin
Tacc3	transforming, acidic coiled-coil containing protein 3

Table S10 continued

**Symbol**	**Full name**
Taf1d	TATA box binding protein (Tbp)-associated factor, RNA polymerase I, D
Tank	TRAF family member-associated NFKB activator
Taok3	TAO kinase 3
Tap1, 2	transporter 1, 2 ATP-binding cassette, sub-family B (MDR/TAP)
Tapbp	TAP binding protein
Tardbp	TAR DNA binding protein
Tars, 2	threonyl-tRNA synthetase, 2 (mitochondrial)
Tax1bp3	Tax1 (human T-cell leukemia virus type I) binding protein 3
Tbc1d20	TBC1 domain family, member 20
Tbccd1	TBCC domain containing 1
Tbx6, 21	T-box transcription factor 6, 21
Tcaf1	TRPM8 channel-associated factor 1
Tcea2, l8	transcription elongation factor A (SII) 2, -like 8
Tcf19	transcription factor 19
Tcn2	transcobalamin 2
Tcp11l2	t-complex 11 like 2
Tcp11x2	similar to t-complex 11 protein
Tect2	tectonic 2
Tex19	testis expressed 19
Tfap4	transcription factor AP-4
Tfpt	TCF3 (E2A) fusion partner
Tfrc	transferrin receptor
Tgfa, b1	transforming growth factor alpha, beta 1
Tgfbi	transforming growth factor, beta induced
Tgfbr2	transforming growth factor, beta receptor II
Tgm2	transglutaminase 2, C polypeptide
Thbd	thrombomodulin
Thbs2	thrombospondin 2
Timd/Tim1, 3, 4	T cell immunoglobulin and mucin domain containing 1, 3, 4
Timm8a1	translocase of inner mitochondrial membrane 8 homolog a1
Timp1	Metallopeptidase inhibitor 1
Tjap1	tight junction associated protein 1
Tjp3	tight junction protein 3
Tk1	thymidine kinase 1, soluble
Tlcd1	TLC domain containing 1
Tlr2	toll-like receptor 2
Tm4sf1	transmembrane 4 L six family member 1
Tma16	translation machinery associated 16 homolog
Tmco4	transmembrane and coiled-coil domains 4
Tmem116, 121, 123	transmembrane protein 116, 121, 123
Tmem140, 189	transmembrane protein 140, 189
Tmem173	transmembrane protein 173
Tmem192, 199	transmembrane protein 192, 199
Tmem204, 216, 229b	transmembrane protein 204, 216, 229b
Tmem250, 256, 268	transmembrane protein 250, 256, 268
Tmem45a, 47, 50b, 62	transmembrane protein 45A, 47, 50b, 62
Tnf	Tumor necrosis factor
Tnfrsf11b, 9	tumor necrosis factor receptor superfamily, member 11b, member 9
Tnip2	TNFAIP3 interacting protein 2
Tnn	tenascin N
Tnxb	tenascin XB
Tob1	transducer of ErbB-2.1
Tollip	toll interacting protein
Tom1	target of myb1 homolog (chicken)
Tonsl	tonsoku-like, DNA repair protein
Top2a	topoisomerase (DNA) II alpha
Topbp1	topoisomerase (DNA) II binding protein 1
Tp53	tumor protein p53
Tp53inp1	tumor protein p53 inducible nuclear protein 1
Tpbgl	similar to trophoblast glycoprotein
Tprn	taperin
Tpx2	TPX2, microtubule-associated, homolog
Tradd	TNFRSF1A-associated via death domain
Traf2	Tnf receptor-associated factor 2
Traf4af1	TRAF4 associated factor 1
Trak2	trafficking protein, kinesin binding 2
Trappc6b	trafficking protein particle complex 6B
Trib2, 3	tribbles homolog 2, 3
Trim25, 37, 42, 8	tripartite motif-containing 25, 37, 42, 8
Trit1	tRNA isopentenyltransferase 1
Trmt6	tRNA methyltransferase 6 homolog
Trpt1	tRNA phosphotransferase 1
Tsc22d3	TSC22 domain family, member 3
Tsen54	tRNA splicing endonuclease 54 homolog
Tsg101	tumor susceptibility 101

Table S10 continued

Symbol	Full name
Tspan2, 3, 5, 7, 8	tetraspanin 2, 3, 5, 7, 8
Tssc4	tumor suppressing subtransferable candidate 4
Ttc30b	tetratricopeptide repeat domain 30B, 39C
Ttc32	tetratricopeptide repeat domain 32
Ttyh3	tweety homolog 3 (Drosophila)
Tuba1a, b4a,b4b, 5	tubulin, alpha 1A, beta 4A class Iva, beta 4B class IVb, beta 5 class I
Txnip	thioredoxin interacting protein
Tyro3	TYRO3 protein tyrosine kinase
Tyw5	tRNA-yW synthesizing protein 5
Uap1, 1l1	UDP-N-acteylglucosamine pyrophosphorylase 1, 1-like 1
Uba7	ubiquitin-like modifier activating enzyme 7
Ubald2	family with sequence similarity 100, member B
Ube2c	ubiquitin-conjugating enzyme E2C
Ube4b	ubiquitination factor E4B
Ubl5	ubiquitin-like 5
Ubqln1	ubiquilin 1
Uchl5	ubiquitin carboxyl-terminal hydrolase L5
Uck2	uridine-cytidine kinase 2
Ufc1	ubiquitin-fold modifier conjugating enzyme 1
Unc5b	unc-5 homolog B
Upp1	uridine phosphorylase 1
Uprt	uracil phosphoribosyltransferase (FUR1) homolog (S. cerevisiae)
Uqcc2	ubiquinol-cytochrome c reductase complex assembly factor 2
Uqcr11, h	ubiquinol-cytochrome c reductase, complex III subunit XI, hinge protein
Uri1	URI1, prefoldin-like chaperone
Usp14, 18, 2, 42	ubiquitin specific peptidase 14, 18, 2, 42
Utrn	utrophin
Vamp1	vesicle-associated membrane protein 1
Vapa	VAMP (vesicle-associated membrane protein)-associated protein A
Vax2	ventral anterior homeobox 2
Vcam1	vascular cell adhesion molecule 1
Vcl	vinculin
Vegfa	vascular endothelial growth factor A
Vegfr2	kinase insert domain receptor (kdr)
Vhl	von Hippel-Lindau tumor suppressor
Vim	vimentin
Vkorc1	vitamin K epoxide reductase complex, subunit 1
Vldlr	very low density lipoprotein receptor
Vof16	ischemia related factor vof-16
Vom1r57, 61	vomeronasal 1 receptor 57, 61
Vps35	vacuolar protein sorting 35 homolog
Vsnl1	visinin-like 1
Vwa1	von Willebrand factor A domain containing 1
Vwf	von Willebrand factor
Wars	tryptophanyl-tRNA synthetase
Wdcp	WD repeat and coiled coil containing
Wdfy1	WD repeat and FYVE domain containing 1
Wdr12, 25, 35l, 37	WD repeat domain 12, 25-like, 35-like, 37
Wdr53, 70, 77, 89	WD repeat domain 53, 70, 77, 89
Wfdc1	WAP four-disulfide core domain 1
Wfdc21	WAP four-disulfide core domain 21provided
Whamm	WAS protein homolog associated with actin, golgi membranes and microtubules
Whsc1	Wolf-Hirschhorn syndrome candidate 1 (human)
Wnt5a	wingless-type MMTV integration site family, member 5A
Wwc2	WW and C2 domain containing 2
Xdh	xanthine dehydrogenase
Xpo1	exportin 1, CRM1 homolog
Xpot	exportin, tRNA (nuclear export receptor for tRNAs)
Xylb	xylulokinase homolog (H. influenzae)
Yae1d1	Yae1 domain containing 1
Yars	tyrosyl-tRNA synthetase
Yipf2	Yip1 domain family, member 2
Ylpm1	YLP motif containing 1
Ypel5	yippee-like 5 (Drosophila)
Yrdc	yrdC domain containing (E.coli)
Zak	sterile alpha motif and leucine zipper containing kinase AZK
ZAP70	zeta chain of T cell receptor associated protein kinase 70
Zbtb21	zinc finger protein 295
Zbtb38, 7b, 8b	zinc finger and BTB domain containing 38, 7B, 8B
Zbtb8os	zinc finger and BTB domain containing 8 opposite strand
Zc3h11a, 8	zinc finger CCCH-type containing 11A, 8
Zcchc2	zinc finger, CCHC domain containing 2
Zdhhc14, 16, 2	zinc finger, DHHC-type containing 14, 16, 2
Zeb2	zinc finger E-box binding homeobox 2
Zfand2a	zinc finger, AN1-type domain 2A

Table S10 continued

**Symbol**	**Full name**
Zfp207, 278	zinc finger protein 207, 278
Zfp280c	suppressor of hairy wing homolog 3
Zfp592, 775, 787, 846	zinc finger protein 592, 775, 787, 846
Zfr	zinc finger RNA binding protein
Zmynd15	zinc finger, MYND-type containing 15
Znf24	zinc finger protein 191
Znrf4	zinc and ring finger 4
Zscan2, 21	zinc finger and SCAN domain containing 2, 21

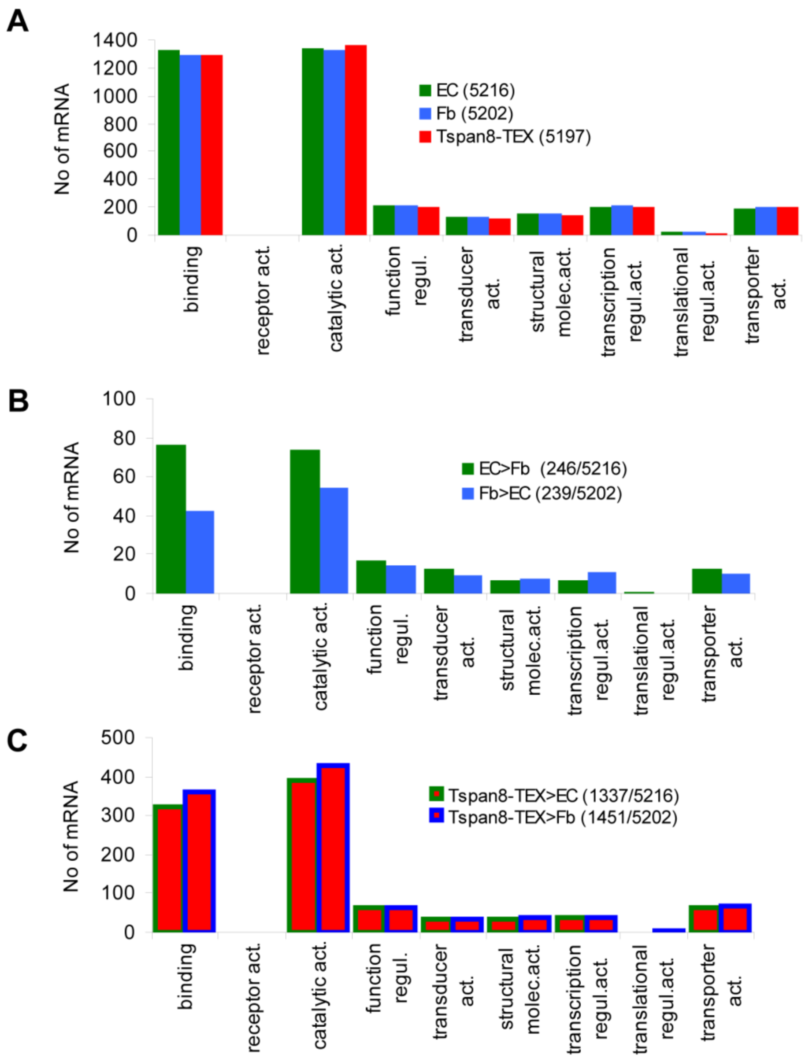

Figure S1 **Functional assignment of mRNA in endothelial cells, fibroblasts, and AS-Tspan8-TEX.** The mRNA profile of EC, Fb, and AS-Tspan8-TEX was evaluated by DS. Molecular functions (Panther pathway analysis) of genes with a signal strength ≥1000 are shown. (A) Panther tool analysis of molecular functions of 5216, 5202 and 5197 genes displayed a signal strength of >1000 in EC, Fb and AS-Tspan8-TEX, respectively; (B) Panther tool analysis of 246 mRNA in EC and 239 mRNA in Fb with an ≥2-fold increase in EC versus Fb and vice versa. (C) Panther tool analysis of Tspan8-TEX RNA that was ≥2-fold increased compared to EC and Fb.

EC, Fb, and AS-Tspan8-TEX showed a dominance of binding and catalytic activity without significant differences between the three mRNA preparations. However, EC RNA showed a slight preponderance in binding and catalytic activity and Fb in transcriptional regulator activation. No particular changes were seen in the distribution of molecular functions of AS-Tspan8-TEX versus EC or Fb mRNA.

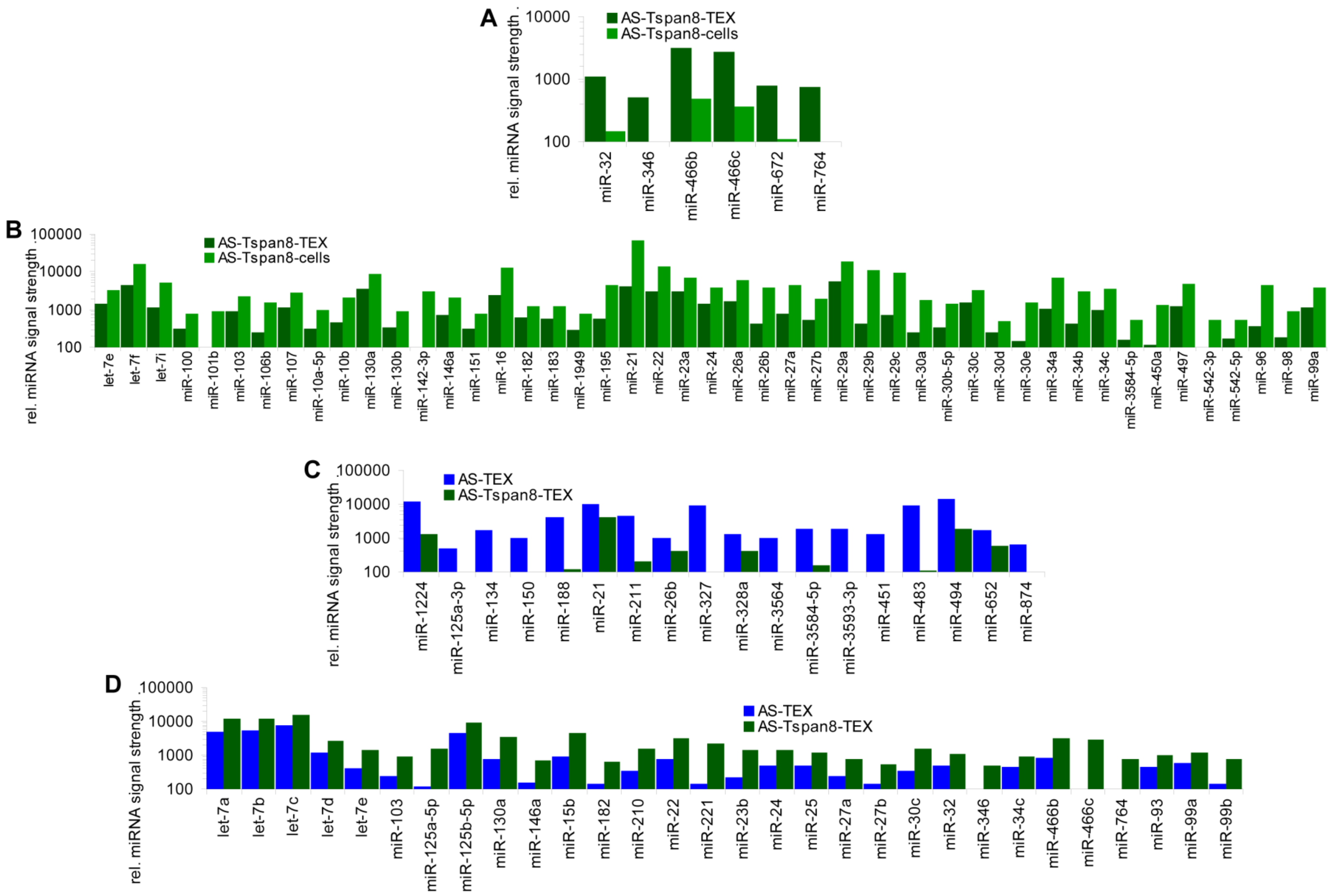
A
rel. miRNA signal strength
10000
1000
100
AS-Tspan8-TEX
AS-Tspan8-cells
miR-32
miR-346
miR-466b
miR-466c
miR-672
miR-764
B
100000
10000
1000
100
AS-Tspan8-TEX
AS-Tspan8-cells
let-7e
let-7f
let-7i
miR-100
miR-101b
miR-103
miR-106b
miR-107
miR-10a-5p
miR-10b
miR-130a
miR-130b
miR-142-3p
miR-146a
miR-151
miR-16
miR-182
miR-183
miR-1949
miR-195
miR-21
miR-22
miR-23a
miR-24
miR-26a
miR-26b
miR-27a
miR-27b
miR-29a
miR-29b
miR-29c
miR-30a
miR-30b-5p
miR-30c
miR-30d
miR-30e
miR-34a
miR-34b
miR-34c
miR-3584-5p
miR-450a
miR-497
miR-542-3p
miR-542-5p
miR-96
miR-98
miR-99a
C
100000
10000
1000
100
AS-TEX
AS-Tspan8-TEX
miR-1224
miR-125a-3p
miR-134
miR-150
miR-188
miR-21
miR-211
miR-26b
miR-327
miR-328a
miR-3564
miR-3584-5p
miR-3593-3p
miR-451
miR-483
miR-494
miR-652
miR-874
D
100000
10000
1000
100
AS-TEX
AS-Tspan8-TEX
let-7a
let-7b
let-7c
let-7d
let-7e
miR-103
miR-125a-5p
miR-125b-5p
miR-130a
miR-146a
miR-15b
miR-182
miR-210
miR-22
miR-221
miR-23b
miR-24
miR-25
miR-27a
miR-27b
miR-30c
miR-32
miR-346
miR-34c
miR-466b
miR-466c
miR-764
miR-93
miR-99a
miR-99b

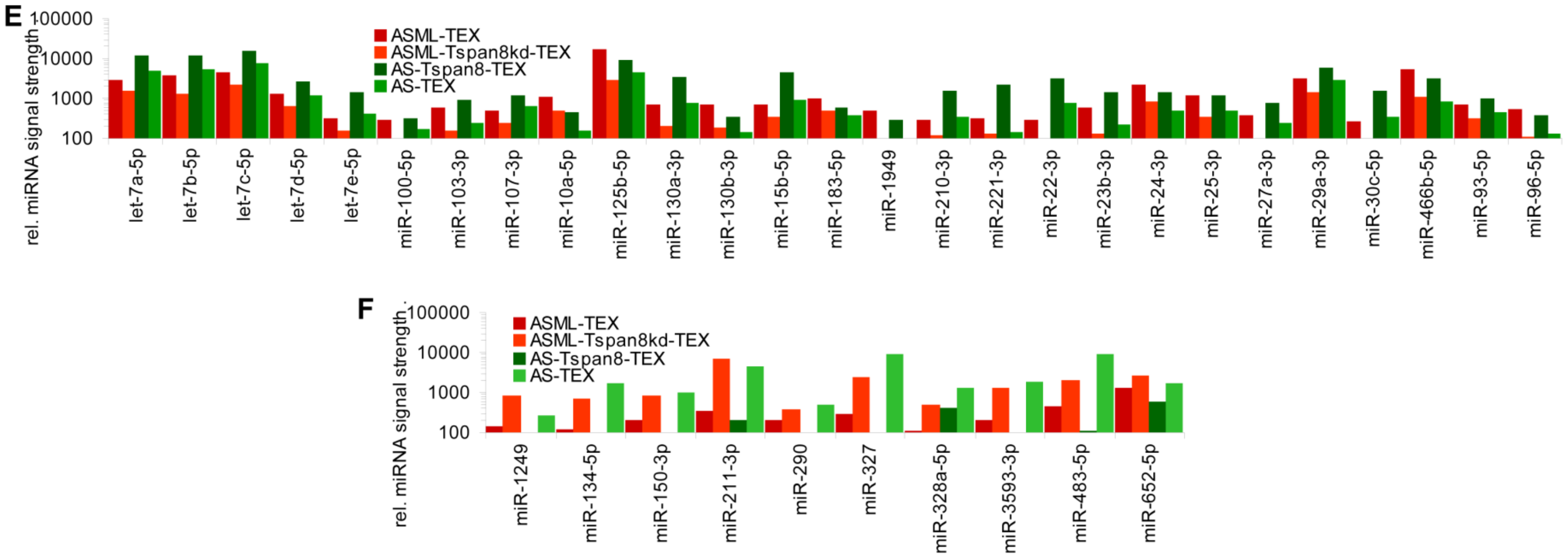

Figure S2 **Distinct miRNA recovery in AS, AS-Tspan8-, ASML- and ASML-Tspan8kd TEX and cells.** The miRNA profile was approached using Agilent miRNA arrays. Only miRNA displaying at least in one of the samples a signal strength of >200 and a ≥2-fold difference are shown. (A,B) miRNA higher in AS-Tspan8 TEX than cells and vice versa. (C,D) miRNA higher in AS-TEX than AS-Tspan8-TEX and vice versa. (E,F) miRNA higher or lower in ASML- than ASML-Tspan8kd TEX in comparison to the miRNA in AS-Tspan8- versus AS-TEX.

Unexpectedly, a higher number of miRNA are more abundant in AS-Tspan8 cells than -TEX and AS-Tspan8-TEX showed more frequently upregulated miRNA than AS-TEX. This feature was confirmed for ASML- versus ASML-Tspan8kd-TEX. The findings are in line with a minor contribution of Tspan8 to miRNA recruitment into TEX.

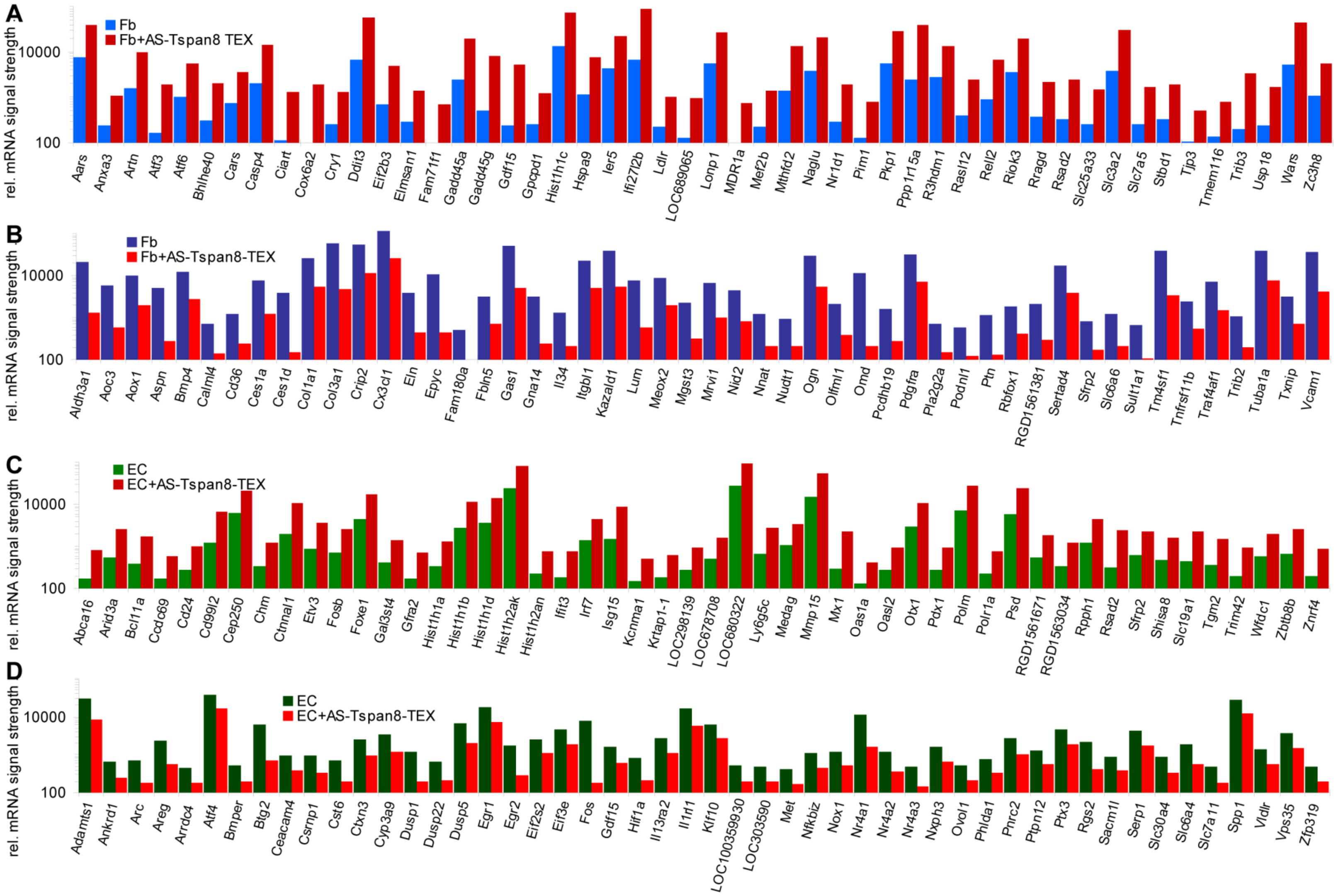
A
Fb
Fb+AS-Tspan8 TEX
rel. mRNA signal strength
10000
100
Aars
Anxa3
Artn
Atf3
Atf6
Bhlhe40
Cars
Casp4
Ciart
Cox6a2
Cry1
Ddit3
Eif2b3
Elmsan1
Fam71f1
Gadd45a
Gadd45g
Gdf15
Gpcpd1
Hist1h1c
Hspa9
Ier5
Ifi27l2b
Ldlr
LOC689065
Lonp1
MDR1a
Mef2b
Mthfd2
Naglu
Nr1d1
Pim1
Pkp1
Ppp1r15a
R3hdm1
Rasl12
Rell2
Riok3
Rragd
Rsad2
Slc25a33
Slc3a2
Slc7a5
Stbd1
Tjp3
Tmem116
Trib3
Usp18
Wars
Zc3h8
B
Fb
Fb+AS-Tspan8-TEX
rel. mRNA signal strength
10000
100
Aldh3a1
Aoc3
Aox1
Aspn
Bmp4
Calml4
Cd36
Ces1a
Ces1d
Col1a1
Col3a1
Crip2
Cx3cl1
Eln
Epyc
Fam180a
Fbln5
Gas1
Gna14
Il34
Itgbl1
Kazald1
Lum
Meox2
Mgst3
Mrvi1
Nid2
Nnat
Nudt1
Ogn
Olfml1
Omd
Pcdhb19
Pdgfra
Pla2g2a
Podnl1
Ptn
Rbfox1
RGD1561381
Sertad4
Sfrp2
Slc6a6
Sult1a1
Tm4sf1
Tnfrsf11b
Traf4af1
Trib2
Tuba1a
Txnip
Vcam1
C
EC
EC+AS-Tspan8-TEX
rel. mRNA signal strength
10000
100
Abca16
Arid3a
Bcl11a
Ccdc69
Cd24
Cd99l2
Cep250
Chm
Ctnnal1
Etv3
Fosb
Foxe1
Gal3st4
Gfra2
Hist1h1a
Hist1h1b
Hist1h1d
Hist1h2ak
Hist1h2an
Ifit3
Irf7
Isg15
Kcnma1
Krtap1-1
LOC298139
LOC678708
LOC680322
Ly6g5c
Medag
Mmp15
Mx1
Oas1a
Oasl2
Otx1
Pdx1
Polm
Polr1a
Psd
RGD1561671
RGD1563034
Rpph1
Rsad2
Sfrp2
Shisa8
Slc19a1
Tgm2
Trim42
Wfdc1
Zbtb8b
Znrf4
D
EC
EC+AS-Tspan8-TEX
rel. mRNA signal strength
10000
100
Adamts1
Ankrd1
Arc
Areg
Arrdc4
Atf4
Bmper
Btg2
Ceacam4
Csrnp1
Cst6
Ctxn3
Cyp3a9
Dusp1
Dusp22
Dusp5
Egr1
Egr2
Eif2s2
Eif3e
Fos
Gdf15
Hif1a
Il13ra2
Il1rl1
Klf10
LOC100359930
LOC303590
Met
Nfkbiz
Nox1
Nr4a1
Nr4a2
Nr4a3
Nxph3
Ovol1
Phlda1
Pnrc2
Ptpn12
Ptx3
Rgs2
Sacm1l
Serp1
Slc30a4
Slc6a4
Slc7a11
Spp1
Vldlr
Vps35
Zfp319

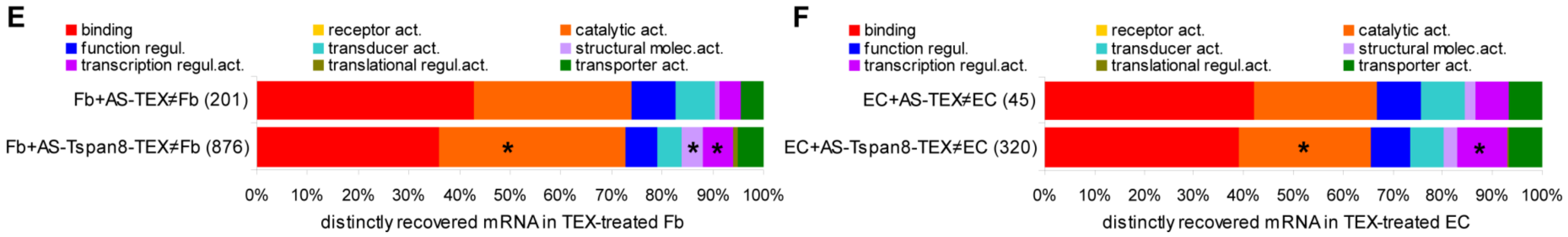

Figure S3 **Distinct mRNA recovery in fibroblasts and endothelial cells cocultured with AS-Tspan8-TEX.** Fb and EC were cocultured for 2d-3d with 30µg/ml AS-Tspan8-TEX. mRNA was isolated and subjected to DS. The 50 mRNA with the strongest change in expression are shown for (A,B) Fb mRNA and (C,D) EC mRNA. Full name of gene symbols: Table S10. (E,F) Molecular function of up- or downregulated Fb and EC mRNA after coculture with TEX. The percent of mRNA engaged in selective molecular functions is shown. Fb and EC respond more frequently with upregulation of catalytic and transcription regulators to AS-Tspan8- than AS-TEX.

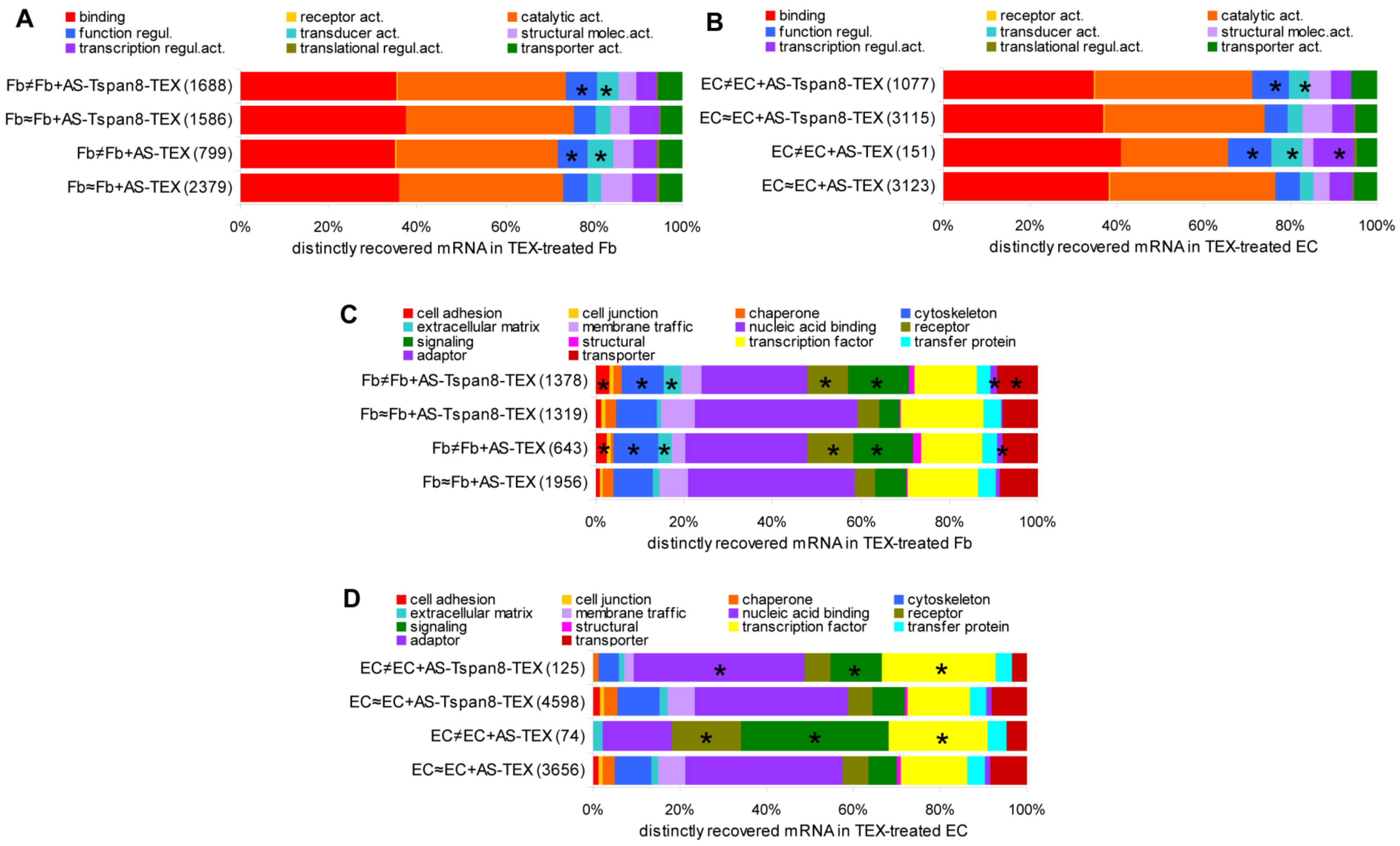

Figure S4 **Overview on molecular functions of fibroblast and endothelial cell mRNA after coculture with AS- or AS-Tspan8-TEX**. Panther pathway analysis of Fb and EC mRNA that expression remained unchanged or changed by AS- or AS-Tspan8-TEX treatment in (A,B) Fb and (C,D) EC according to molecular functions and protein classes. Molecular functions and protein classes that were expanded by AS- and AS-Tspan8-TEX treatment are indicated by a black *.

Particularly sorting according to protein classes revealed abundant changes in TEX-treated Fb and EC.

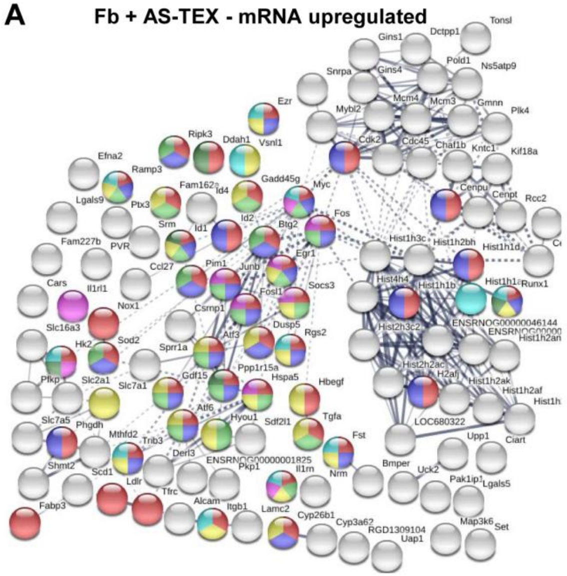

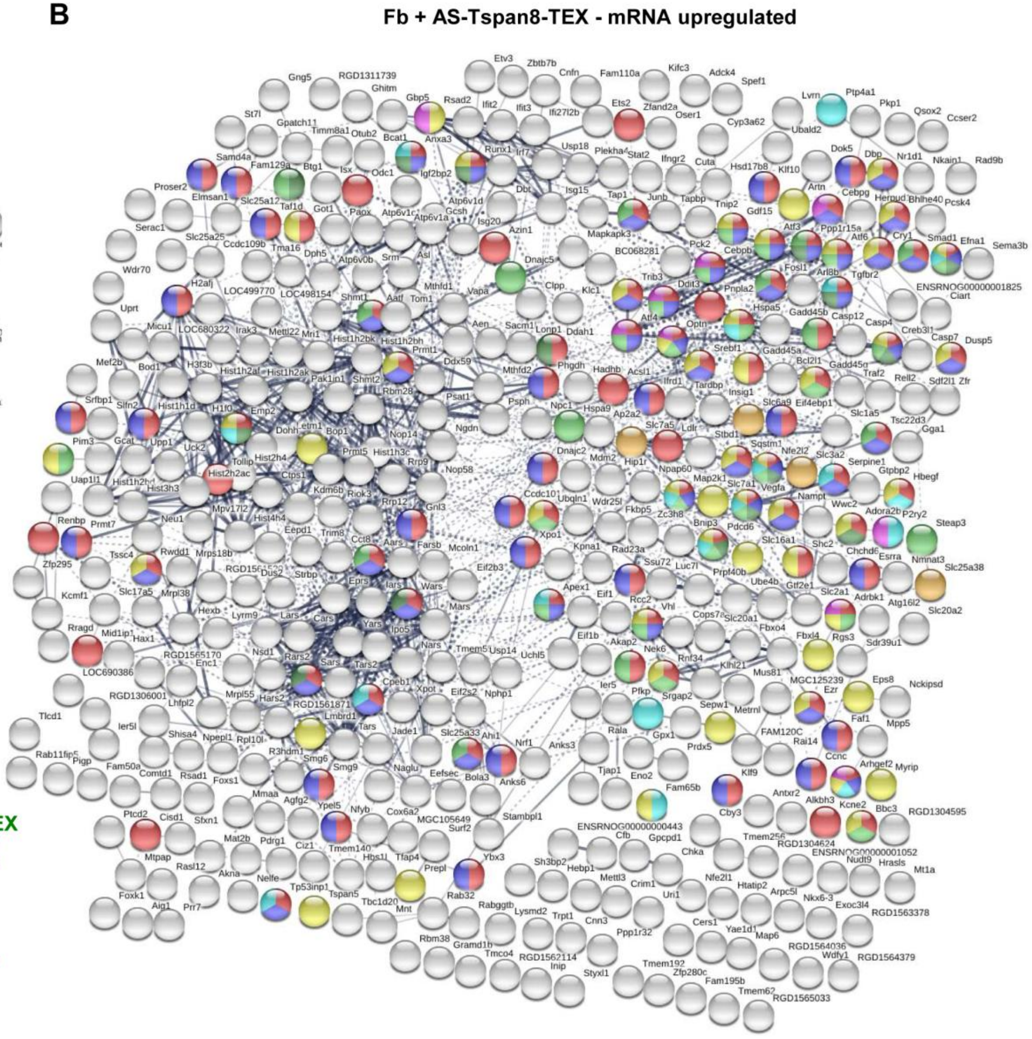

C **overrepresented edges – upregulated mRNA**

process	AS-TEX	Fb + AS-Tspan8-TEX
regulation metabolism	$7.77x10^{11}$	$5.33x10^{8}$
regulation gene express.	$2.84x10^{8}$	$5.33x10^{8}$
regulation apoptosis	$3.19x10^{8}$	0.00038
regulation signaling	$1.17x10^{5}$	0.00430
regulation cytokines	0.00080	0.00247
regulation angiogenesis	0.0063	$1.49x10^{5}$
regulation migration	0.0184	0.0041
regulation transport	0.0363	0.0279

D

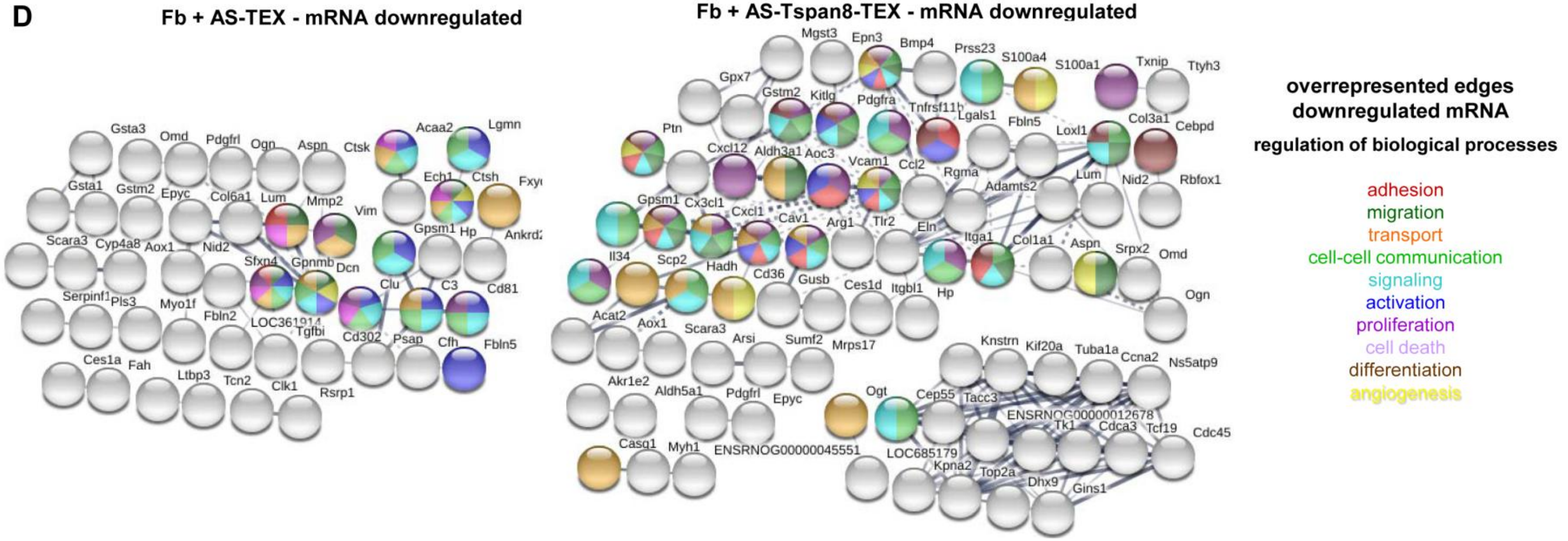
Fb + AS-TEX - mRNA downregulated
Fb + AS-Tspan8-TEX - mRNA downregulated
overrepresented edges
downregulated mRNA
regulation of biological processes
adhesion
migration
transport
cell-cell communication
signaling
activation
proliferation
cell death
differentiation
angiogenesis

E

Altered apoptosis in AS-TEX treated Fb

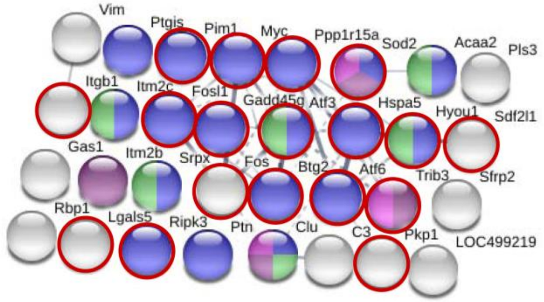

Altered apoptosis in AS-Tspan8-TEX treated Fb

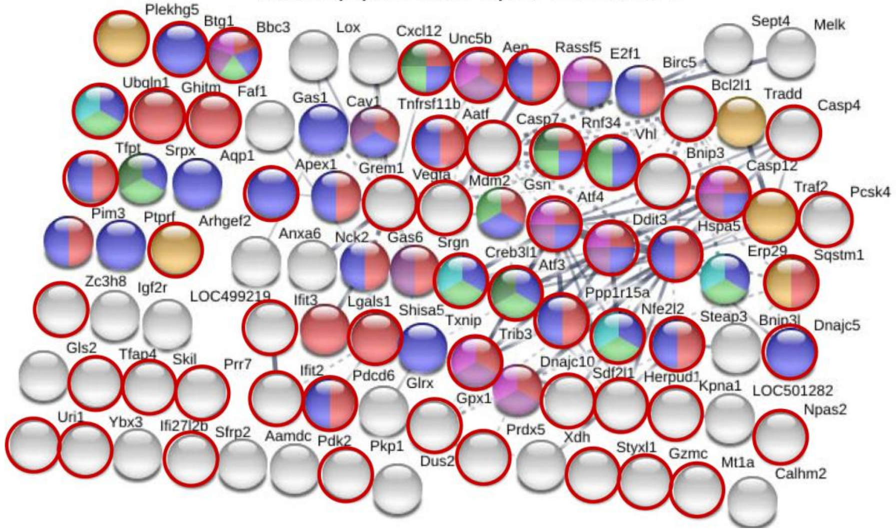

Altered apoptosis (overrepresented) in AS-and AS-Tspan8-TEX treated Fb

Apoptosis	Fb AS-TEX	AS-Tsp8-TEX
Apoptotic process	--	29
Apoptotic signalin pathway	4	10
Death receptor signaling	--	5
Intrinsic apoptosis sign. pathway	3	8
Reg. apoptotic process	18	35
Reg. apoptotic signaling pathway	6	11
Reg. extr. apopt. signal. pathway	--	4
Reg.intrins. apopt. signal. pathway	--	4

upregulated mRNA ○

F

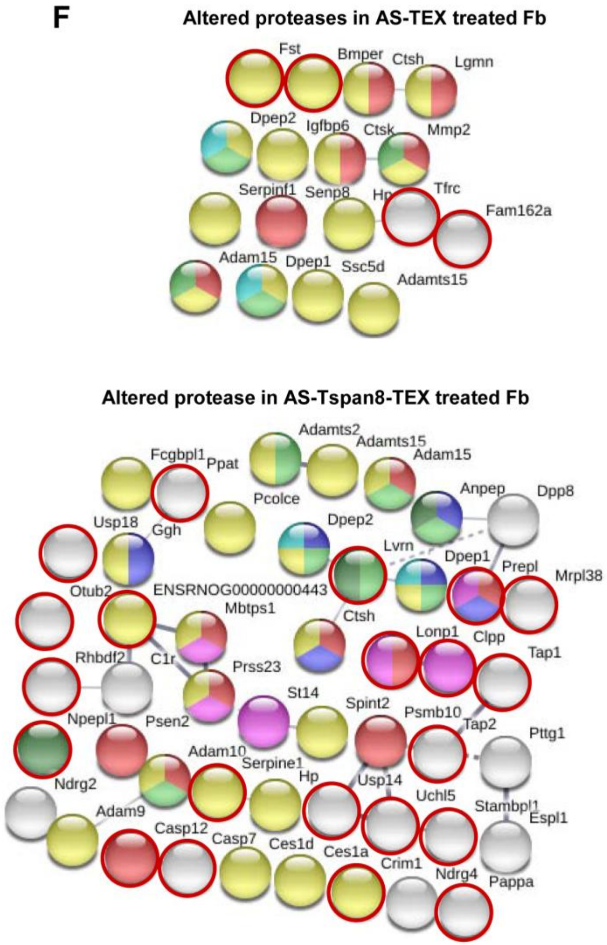

Altered protease assignment (overrepresented) in AS-and AS-Tspan8-TEX treated Fb

Proteases	Fb AS-TEX	Fb AS-Tsp8-TEX
Endopeptidase	6	10
Exopeptidase	--	6
Metalloproteinase	2	7
Serine peptidase	--	6
Aminopepidase	2	3
Dipeptidase	2	2
Signaling engaged proteases	14	20

upregulated mRNA ○

Figure S5. AS- and AS-TEX-promoted fibroblast modulation. mRNA was evaluated in AS- and AS-Tspan8-TEX-treated Fb for up- or downregulation compared to untreated Fb. Analyses included mRNA with a signal strength ≥1000 and ≥2-fold difference between untreated and TEX-treated Fb. Analysis was performed using the STRING functional protein analysis program. (A-C) mRNA upregulated in (A) AS-TEX- and (B) AS-Tspan8-TEX-treated Fb, and (C) engagement of overrepresented edges in the regulation of biological processes corresponds to Fig.4A-4C, but includes non-connected nodes; (D) mRNA downregulated in AS-TEX- and AS-Tspan8-TEX-treated Fb and color code definition of overrepresented edges according to regulation of biological processes (significance values being given in Fig.4D). (E) Altered apoptosis related mRNA in AS- and AS-Tspan8-TEX treated Fb and number of mRNA in overrepresented edges. (F) Altered protease mRNA in AS- and AS-Tspan8-TEX treated Fb and number of mRNA in overrepresented edges. (E,F) Upregulated mRNA is indicated by a red circle. Full name of gene symbols: Table S10.

AS-Tspan8-TEX also affected downregulation of proteins more strongly than AS-TEX, the only exception being cell death regulation. This is reflected by 37.5% of apoptosis regulating genes being upregulated in AS-TEX-treated, but only 18.6% in AS-Tspan8-TEX-treated Fb. There was no evidence for a strong impact of AS- and AS-Tspan8-TEX on modulating the Fb protease profile. However, exopeptidases are only upregulated after AS-Tspan8-TEX treatment. These findings suggest that AS-Tspan8-TEX promote Fb invasion mostly via signaling initiated cell adhesion, migration and cell-cell communication.

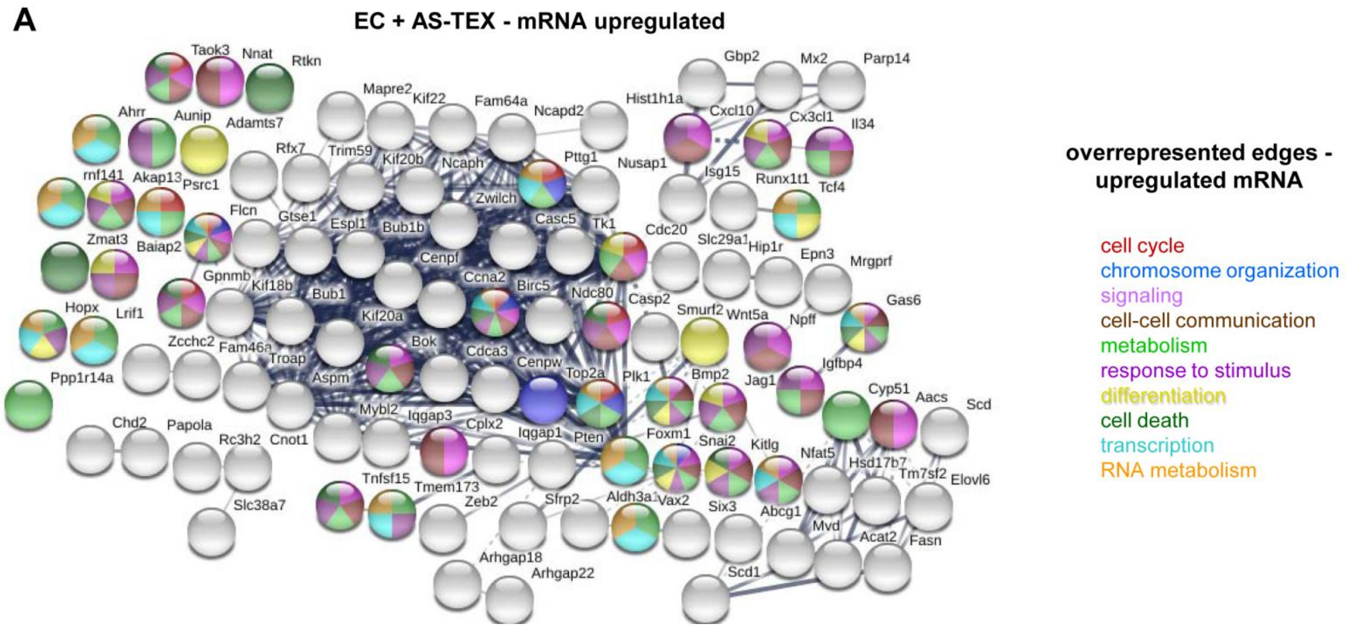
A
EC + AS-TEX - mRNA upregulated
Taok3
Nnat
Rtkn
Ahrr
Aunip
Adamts7
rnf141
Akap13
Psrc1
Zmat3
Baiap2
Flcn
Hopx
Lrif1
Ppp1r14a
Mapre2
Kif22
Fam64a
Ncapd2
Hist1h1a
Gbp2
Mx2
Parp14
Rfx7
Trim59
Kif20b
Ncaph
Pttg1
Nusap1
Cxcl10
Cx3cl1
Il34
Isg15
Runx1t1
Tcf4
Gtse1
Espl1
Bub1b
Zwilch
Casc5
Tk1
Cdc20
Slc29a1
Hip1r
Epn3
Mrgprf
Gpnmb
Kif18b
Bub1
Cenpf
Ccna2
Birc5
Ndc80
Casp2
Smurf2
Wnt5a
Npff
Gas6
Kif20a
Zcchc2
Fam46a
Troap
Aspm
Bok
Cdca3
Cenpw
Top2a
Plk1
Bmp2
Jag1
Igfbp4
Cyp51
Aacs
Scd
Chd2
Papola
Rc3h2
Cnot1
Mybl2
Iqgap3
Cplx2
Iqgap1
Pten
Foxm1
Snai2
Kitlg
Nfat5
Hsd17b7
Tm7sf2
Elovl6
Slc38a7
Tnfsf15
Tmem173
Zeb2
Sfrp2
Aldh3a1
Vax2
Six3
Abcg1
Mvd
Acat2
Fasn
Arhgap18
Arhgap22
Scd1
overrepresented edges - upregulated mRNA
cell cycle
chromosome organization
signaling
cell-cell communication
metabolism
response to stimulus
differentiation
cell death
transcription
RNA metabolism

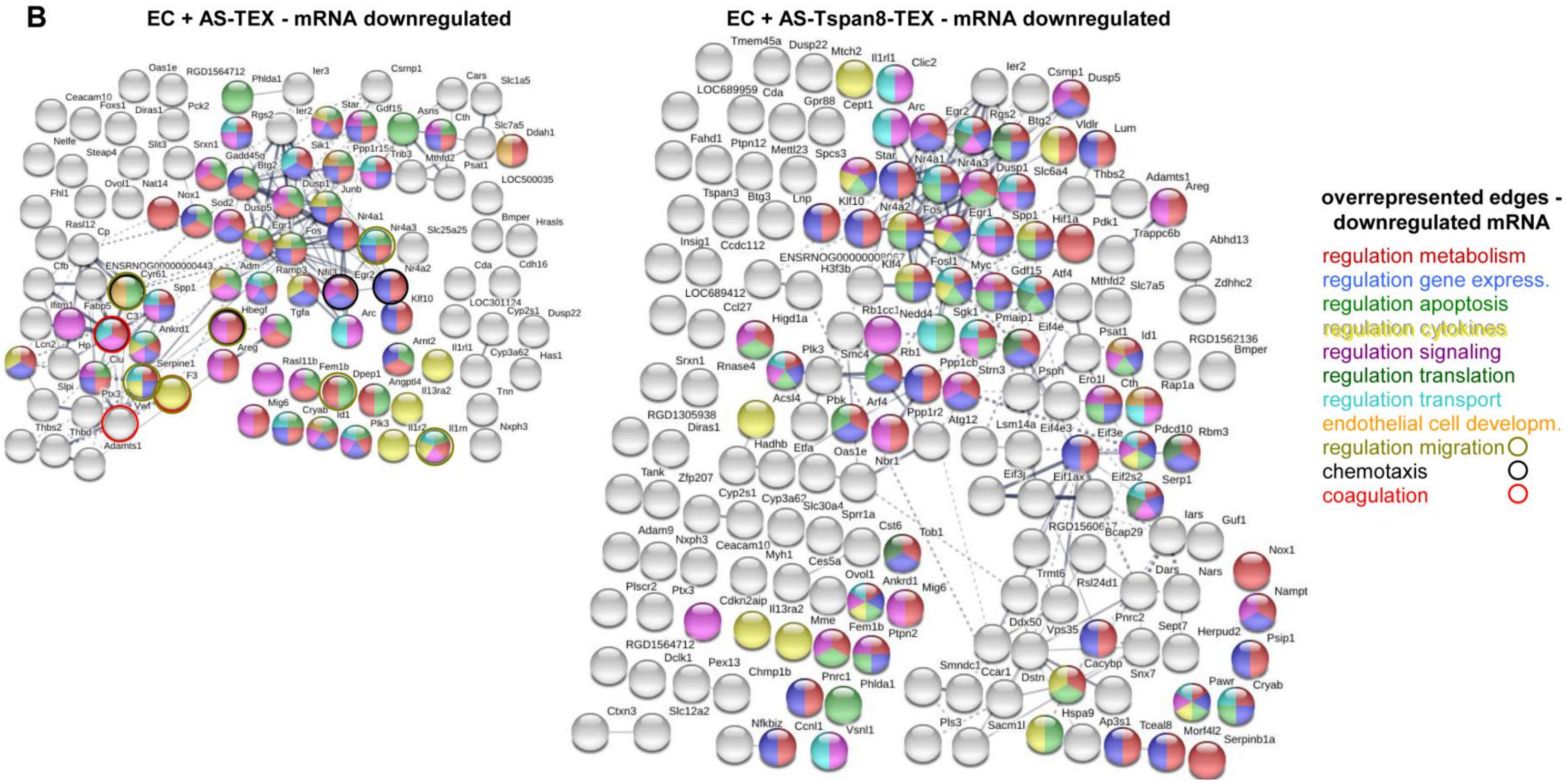

Figure S6. STRING analysis of overrepresented edges in AS- and AS-Tspan8-TEX-treated endothelial cells. STRING analysis of AS- and AS-Tspan8-TEX-treated EC was performed under less stringent conditions (signal strength ≥500, ≥1.5-fold difference). (A) Overrepresented nodes of mRNA with increased expression in AS-TEX-treated EC, overrepresented nodes are indicated by a color code; (B) Overrepresented nodes of mRNA with decreased expression in AS-TEX- and AS-Tspan8-TEX-treated EC, overrepresented nodes are indicated by a color code. . Full name of gene symbols: Table S10.

A

predicted targets of miRNA downregulated after coculture with AS-TEX or AS-Tspan8-TEX

miR-32: ADCY3, ADRB1, ANKIB1, ANKRD44, ARMC1, ATP2A2, C16orf52, CACNA1C, CALM1, CDCA7L, CEP41, DNAJBB9, EDNRB, FBXW7, FRS2, HANND2, HERPUD2, HIPK3, IRS2, ISCA1, ITPR1, MIA3, MMP16, MYO1B, NCOA1, PAPD7, PIK3R3, PLEKHB2, PRKAR1B, PRKAR2B, PRKCE, RAP1B, RNF38, RSBN1, SLC2A3, SSBP3, TMEM229A, UPG2, WASL

miR-129: ARRDC3, IGIP, PMEPA1, ITF200, CXorf21, CIB2, CBBX4, HMGB1, GPR135

miR-466b/c: ACSL4, BRINP3, CALCOCO1, CAT, CEACAM1, CNGA2, DCLK1, ELOVL6, ENPP5, GPM6A, KCNV1, KHDRBS2, MAF, MOBP, RBL2, SLC25A10, SLC2A13, TMEM38B, VSNL1

miR-500: SYYS1, GPR61, PLP1, LPIN1, DCUN1D1, PTPRD, SNX18

miR-29b: ADAMTS10, ADAMTS2, AKT2, AKT3, BRWD3, C1QTNNF6, CDC42, COL4A5, COL9A1, COL1A1, EIF4E2, ELN, FBN1, FRS2, GAB1, HBEGF, IFI30, IREB2, IRS1, MAP2K4, MAPK10, NASP, NFIA, PDGFC, PIK3R1, PIK3R3, PMP22, PTEN, RNF39, SLC30A3, STAT3, SUV420H2, SYPL2, TET3, TGFB2, TGFB3, TMEM236, TSPAN4, VEGFA, YBX3, ZBTB5

miR-34b: ADCY5, ARHGAP1, ARHGAP26, AV3, BCL2, CACNA1E, CASP2, CCND1, CCNE2, E2F3, E2F5, ERC1, FAM167A, FAM76A, FAM83H, GAB1, GMFB, GNAI2, GNAO1, HNRNPA1, IKBKE, LDHA, LIMD2, MAP2K1, MAP3K7CL, MAPK13, NAPEPLD, NCOA1, NOS1, NOTCH1, NRIP3, PACS1, PGF, PLA2G2F, PLCG1, PLN, PRKCE, PRKCQ, PRKD1, RAB43, RALGDS, RRAS, SLLC27A4, SMAD4, SMIIM15, SNX15, SRC, TP53, TTC19, VEGFA, ZDHHC16

miR-193-3p: AP2M1, ARHGEF12, ATM, BCL2L2, CALM1, CBX7, CCND1, CNOT6, DCAF7, DGCR2, E2F6, FAIM2, FHDC1, GLIPR2, GPR27, GRB7, GREM1, HOXD113, ING5, KCNJ2, KIT, KLB, KRAS, MAPK10, MMP19, MYCN, PAK4, PITX2, PLAU, PPP2R4, PPP2R5C, SIAH1, SLC39A5, SRSF2, STMN1, TAOK1, TGFB2, ZBTB5

miR-214: AURKA, CCDC152, CD247, CDC42, CTSO, CYTL1, DIS3, DNAJB9, FBXO30, GLRA3, GLUD2, IL1A, KCNH7, LUZP2, MEMO1, MYYH8, NF2, NUDT15, PAX8, PLA2G4C, PPP3CC, RBFOX1, RNF146, RPAIN, SAPCD1, SENP8, SPRYD4, ST3GAL2, TCF7L2, TCF7L2, TMEM177, TNFAIP6, TNFSF18, TNFSF8, UTP14C

miR-222: ARNT, BTG2, CASP10, CCDC64, CLVS2, DCUN1D4, FAM214A, FOS, FRS2, GAB1, GNAI2, GNAI3, IRX5, KDR, MAPK10, MEGF9, NTF3, PAK1, PIK3R1, PLCL2, PRUNE, PVRL1, RBM24, RFX7, ST8SIA1

miR-301a: ACBD5, ADAM12, BTBD3, DSEL, ESR1, FBXO28, FOXF2, HBP1, IMPDH1, IRF1, KLF7, LMLN, LONNRF1, NFIA, NPAT, PAPD4, SLAIIN1, SNAP25, SYBU, TRERF1, WDR20, ZIC5

miR-1224: ADORA1, CBX3, FANCD2, FOSB, GLYCT1, GTDC1, H3F3B, LBP, MED18, MED20, MRVI1, NIPA2, OST4, PAQR4, PDE1B, POMGNT1, PPP1R9B, PSMA4, RAB2A, RGAG4, SELK, SKP1, STX5, SYPL2, UBXNN1

miR-3584-5p: APEX1, ATXN7L1, BAZ2A, CACNG7, CCDC8, CDC45, CIR1, CIT, CYP2W1, DACT3, DAK, DBNDD2, DEFB4B, EBF2, ERF, FNDC4, GATS, IQSEC3, KIAA0247, LAS1L, LPAR5, MANSC1, MT-ND4L, NUTF2, PAX1, PEA15, PLP2, POLE3, PPP1R12B, PRR13, REXO2, RNF165, RSU1, SCAMP5, SEC14L1, SIX3, SST, TVP23C, U2AF1L4, USP46, VAMP2, ZNF784

miR-34c: ADCY5, ARHGAP199, BCL2, CACNA1E, CASP2, CCND1, E2F3, E2F5, FAM167A, FAM76A, FGD6, GAB1, GNAI2, GNAO1, HCNN3, IKBKE, LDHA, MAP2K1, MAPK13, MDM4, NAPEPLD, NCOA1, NNAV3, NOS1, NOTCH1, NTN1, PACS1, PGF, PLA2G2F, PLCG1, PLN, PRKCE, PRKCQ, PRKD1, RALGDS, RRAS, RTN4RL1, SCN2B, SMAD4, SRC, TP53, TRANK1, TTC19, VEGFA

miR-101a: ABHD17B, AEBP2, AKT3, AP3S1, APP, ASPN, CAV3, CDYL, EYA1, EZH2, FAM60A, FGFR3, FLRT3, FRS2, GAB1, GLTSCR1, GPR27, HTRA3, JAK2, KCNH7, LCOR, MAPK1, METAP1, MMORN4, MYCN, NDFIP1, NLK, NPNT, PCGF5, PHLDA1, PIEZO1, PIK3C2A, PIK3C2B, PLCG1, PTGS2, RAC1, RALBP1, RANBP9, RAP1B, SLC30A4, SMAD2, TGFA, TGFBR1, UBE2D1

miR-140: ARL4C, CCNT2, CLDN1, CORO2A, CREB3L1, EPB41L2, FGF9, GUCD1, HDGFRP3, KATNBL1, KCTD6, KLF5, LHFPL2, MAFG, MARCKS, MBNL3, MEIS2, MMD, MSMP, NFE2L2, PCDH8, PDGFRA, RNF19A, RRAGC, SEPT2, SLC16A6, SMOX, SNX2, SSR2, SSR3, STAT1, TGFB3, TMEM260, TNFSF8, TSSK2, UBAP2L, WNT11, WNT9A, ZNF131, ZNF800

miR-494: ARHGAP5, CYSTMM1, FGFR2, HMOX1, IRS1, MDM2, PGPEP1L, PIK3R3, PRRG1, PTEN, PTPN11

AS-TEX: green
AS-Tspan8-TEX: red
AS- & AS-Tspan8-TEX: violet
underlined: mRNA upregulated

B

C

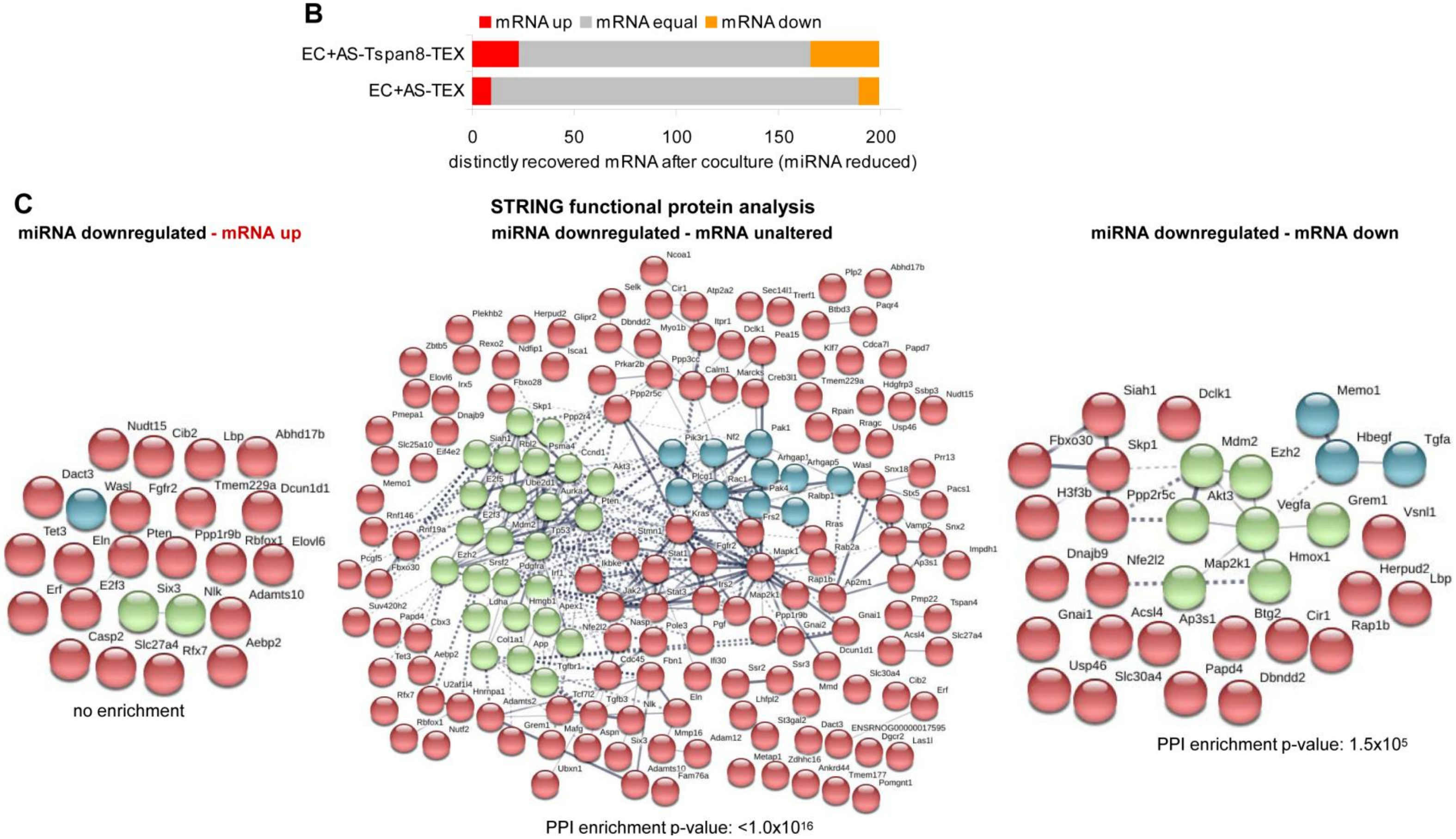

Figure S7. Recovery and functional assignment of predicted target mRNA of downregulated miRNA in TEX-treated endothelial cells. (A) Predicted and confirmed targets of downregulated miRNA after AS- or AS-Tspan8-TEX treatment were searched for by http://www.microrna.org and http://www.targetscan.org. The threshold level of mRNA was set to signal strength of ≥500 and a ≥1.5-fold increase; mRNA upregulated after AS-TEX treatment: green, after AS-Tspan8-TEX treatment: red, after AS- and AS-Tspan8-TEX treatment: violet, upregulated predicted mRNA is underlined. (B) Summary of the recovery of predicted mRNA of miRNA expressed at a lower level in EC after coculture with AS- or AS-Tspan8-TEX; mRNA upregulated after TEX treatment: red, unaltered: grey, downregulated: orange. (C) STRING functional protein network pathway analysis of mRNA targets of miRNA downregulated in TEX-treated EC. Full name of gene symbols: Table S10.
Reduced miRNA expression in TEX-treated EC is rarely accompanied by mRNA release from repression. “Suppression-released” mRNA shows no edge enrichment.

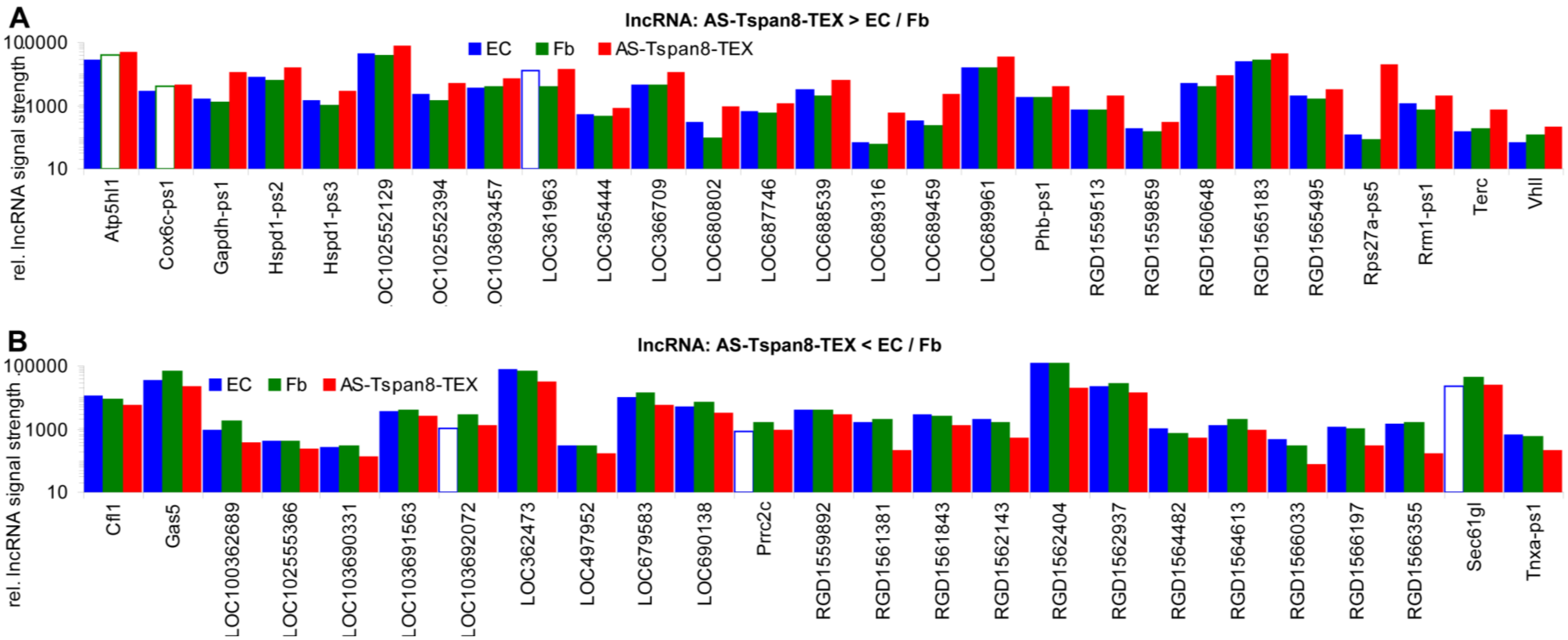

Figure S8. Pseudogenes and lncRNA in fibroblasts, endothelial cells and TEX. (A) Higher and (B) lower nc pseudogene and lncRNA recovery in AS-Tspan8-TEX compared to EC and Fb, a ≥1.5-fold difference being accepted as significant (filled bars), missing differences: empty bar. Full name of gene symbols: Table S8, S9G.
